# HDAC8 Promotes Liver Metastasis of Colorectal Cancer *via* Inhibition of IRF1 and Upregulation of SUCNR1

**DOI:** 10.1155/2022/2815187

**Published:** 2022-08-16

**Authors:** Jierong Chen, Lixue Cao, Jianhong Ma, Caifeng Yue, Dandan Zhu, Ran An, Xiaoxiao Wang, Yunmiao Guo, Bing Gu

**Affiliations:** ^1^Laboratory Medicine, Guangdong Provincial People's Hospital, Guangdong Academy of Medical Sciences, Guangzhou 510080, China; ^2^Research Center of Medical Sciences, Guangdong Provincial People's Hospital, Guangdong Academy of Medical Sciences, Guangzhou 510080, China; ^3^Department of Laboratory Medicine, Central People's Hospital of Zhanjiang, Guangdong Medical University Zhanjiang Central Hospital, Zhanjiang 524045, China; ^4^Zhanjiang Institute of Clinical Medicine, Central People's Hospital of Zhanjiang, Guangdong Medical University Zhanjiang Central Hospital, Zhanjiang 524045, China

## Abstract

Histone deacetylases (HDACs) are well-characterized for their involvement in tumor progression. Herein, the current study set out to unravel the association of HDAC8 with colorectal cancer (CRC). Bioinformatics analyses were carried out to retrieve the expression patterns of HDAC8 in CRC and the underlying mechanism. Following expression determination, the specific roles of HDAC8, IRF1, and SUCNR1 in CRC cell functions were analyzed following different interventions. Additionally, tumor formation and liver metastasis in nude mice were operated to verify the fore experiment. Bioinformatics analyses predicted the involvement of the HDAC8/IRF1/SUCNR1 axis in CRC. *In vitro* cell experiments showed that HDAC8 induced the CRC cell growth by reducing IRF1 expression. Meanwhile, IRF1 limited SUCNR1 expression by binding to its promoter. SUCNR1 triggered the growth and metastasis of CRC by inhibiting cell autophagy. HDAC8 blocked IRF1-mediated SUCNR1 inhibition and thereby inhibited autophagy, accelerating CRC cell growth. Lastly, HDAC8 facilitated the development of CRC and liver metastasis by regulating the IRF1/SUCNR1 axis *in vivo*. Taken together, our findings highlighted the critical role for the HDAC8/IRF1/SUCNR1 axis in the regulation of autophagy and the resultant liver metastasis in CRC.

## 1. Introduction

Colorectal cancer (CRC) represents one of the leading neoplasms diagnosed across the world, further accounting for the second highest cancer-related deaths globally [1]. Age, genetic, and environmental factors, especially a diet rich in red meats and low fruits and vegetables, are all known to increase the risk of developing CRC [2]. Well-established treatment modalities for CRC comprise of surgical management, adjuvant chemotherapy, and neoadjuvant therapy [3]. Adding to the plight of CRC, liver metastasis is detected in approximately 25–30% of CRC patients, with a predisposition for left-sided CRC [4]. Increasing evidences have come to light which highlight CRC tumor cell autophagy as a frequent consequence of this metastasis [5, 6]. On the other hand, suppression of autophagy exerts a diminishing effect on the migratory and invasive features of tumor cells *in vitro* and further reduces metastasis *in vivo* [7]. The abovementioned data necessitates an extensive investigation to determine the possible molecular mechanism underlying CRC cell autophagy, so as to provide biomarkers which can provide prognostic and predictive signs for patients undergoing distant metastases.

Histone deacetylases (HDACs), a class of enzymes that remove acetyl groups from an *ε*-N-acetyl lysine amino acid on a histone, are well-characterized as effective anticancer candidates against aggressive malignancies [8]. One such histone deacetylase, namely, HDAC8, is implicated in the invasion and metastasis abilities of cancer; meanwhile, selective inhibition of HDAC8 serves as a therapeutic agent in various malignancies, including breast cancer, hepatocellular carcinoma, and CRC [9–14]. Moreover, HDACs were previously suggested to inhibit the IFN-gamma-inducible expression of numerous genes, including the IRF1 gene in trophoblast cells [15]. IRF1, a nuclear transcription factor, possesses the ability to mediate the effects of interferon and exhibits antitumorigenic functions, especially in CRC [16]. Interestingly, a prior study highlighted that enforced expression of IRF1 significantly decreases the proliferation of CRC cells and enhances their apoptosis [17], such that both of the latter effects are often associated with the induction of CRC cell autophagy [18, 19]. Initial prediction results from the hTFtarget database revealed the presence of multiple binding sites of the IRF1 gene in the promoter region of the succinate receptor 1 (SUCNR1) gene. SUCNR1, also known as GPR91, is a member of the G protein-coupled receptor family, exhibiting a high potential as a drug target in human diseases, such as hypertension and diabetes [20]. Furthermore, SUCNR1 is known to stimulate intestinal inflammation and fibrosis in the context of Crohn's disease [21], while its potential role in CRC remains uncharacterized. Accordingly, the current study sought to elucidate the possible role of HDAC8/IRF1/SUCNR1 in the progression of CRC, especially from the perspective of CRC cell autophagy and liver metastasis.

## 2. Materials and Methods

### 2.1. Ethics Statement

The current study was approved by the by the Clinical Ethics Committee of Guangdong Provincial People's Hospital, and all experimental protocols were in accordance with the *Declaration of Helsinki*. Signed informed consents were obtained from all participants prior to specimen collection. Animal experimentations were ratified by the Animal Ethics Committee of Guangdong Provincial People's Hospital. Extensive efforts were made to minimize both the number and the respective suffering of the included animals.

### 2.2. Bioinformatics Analysis

Firstly, the expression patterns of HDAC8 and SUCNR1 in CRC samples included in TCGA and GTEx were retrieved from the GEPIA2 database. The potential downstream regulatory factors of HDAC8 were predicted using the ChIPBase database [22, 23]. Simultaneously, information related to transcription factors was downloaded from the Cistrome database. Afterwards, the STRING database was adopted for interaction analysis of candidate genes, and an interaction network map was constructed using the Cytoscape software (v3.7.1), with degree values counted. KEGG enrichment analysis of candidate genes was conducted utilizing the KOBAS3.0 database. A value of *p* value < 0.05 was regarded as statistically significant.

### 2.3. Clinical Sample Collection

CRC tissues and adjacent normal tissues (at least 5 cm away from tumor tissues) were collected from 58 patients with CRC (calculated mean age of 46.81 ± 8.93 years) undergoing radical surgical resection at Guangdong Provincial People's Hospital from June 2018 to June 2020. None of the included patients received local or systemic treatment prior to surgery. The collected tissues were immediately frozen in liquid nitrogen and stored at -80°C for further experimentation.

### 2.4. Cell Culture and Treatment

Four human CRC cell lines (namely, SW480, SW620, HT29, and HCT-116), human normal colorectal epithelial cell line FHC, and HEK293T cells (all procured from American Type Culture Collection, Manassas, VA) were cultured in Dulbecco's modified Eagle's medium (10569044, Gibco, Grand Island, NY) appended to 10% fetal bovine serum (FBS; 10099141, Gibco), 2 mM L-glutamine (Sigma-Aldrich, St Louis, MO), 100 U/mL penicillin, and 100 *μ*g/mL streptomycin in a 5% CO_2_ incubator at 37°C. HCT-116 cells were treated with PCI-34051 (5 *μ*M), a specific inhibitor of HDAC8, for 24 h [24].

Logarithmically growing CRC cells were seeded in a 6-well culture plate (4 × 10^5^ cells/well). Upon reaching 70-80% confluence, cell transfection was carried out using the Lipofectamine 2000 reagent (11668-019, Invitrogen Inc., Carlsbad, CA) with short hairpin RNA-negative control (sh-NC), sh-HDAC8, overexpression- (oe-) NC, oe-HDAC8, sh-HDAC8+sh-NC, sh-HDAC8+sh-IRF1, oe-IRF1, sh-SUCNR1, sh-HDAC8+oe-NC, and sh-HDAC8+oe-SUCNR1 (all sequences and plasmids were purchased from GenePharma Co., Ltd., Shanghai, China). Following transfection, the cells were cultured at 37°C with 5% CO_2_ and saturated humidity. After 6 h, the medium containing transfection fluid in the well was discarded, and the cells were allowed to culture with medium replenished with 10% FBS for 24-48 h for follow-up experimentations.

### 2.5. Reverse Transcription Quantitative Polymerase Chain Reaction (RT-qPCR)

Total RNA content was extracted using the TRIzol reagent (16096020, Thermo Fisher Scientific Inc., Waltham, MA). The obtained RNA was reverse-transcribed into cDNA utilizing Reverse Transcription kits (RR047A, Takara, Japan). RT-qPCR was carried out by means of the SYBR® Premix Ex Taq™ II kit (DRR081, Takara) on an ABI 7500 instrument (Applied Biosystems, Foster City, CA). GAPDH was utilized as a normalizer, and the fold changes were calculated utilizing the 2^-△△Ct^ method. The primer sequences are described in Supplementary Table 1.

### 2.6. Western Blot Analysis

Total protein content was extracted with the help of a radioimmunoprecipitation assay lysis buffer (P0013B, Beyotime Biotechnology, Shanghai, China) containing 1% protease inhibitor and phosphorylase inhibitor, and the obtained protein concentration was analyzed using bicinchoninic acid kits (Boster, A53226, Thermo Fisher Scientific). After undergoing electrophoresis separation, the proteins were transferred onto a polyvinylidene fluoride membrane (IPVH85R, Merck Millipore, Billerica, MA). Following blockade with 5% bovine serum albumin (BSA), the membrane was probed with primary antibodies (Abcam Inc., Cambridge, UK) against HDAC8 (ab187139, 1 : 10000), IRF1 (ab191032, 1 : 1000), SUCNR1 (ab272856, 1 : 1000), LC3 (ab128025, 1 : 1000), and GAPDH (ab181602, 1 : 2500, normalizer) overnight at 4°C. The following day, the membrane was reprobed with the horseradish peroxidase- (HRP-) labeled secondary antibody IgG (ab6721, 1 : 5000, Abcam) for 2 h. Afterwards, the immunocomplexes were visualized with an enhanced chemiluminescence reagent, and band intensities were quantified using the ImageJ 1.48u software (V1.48, National Institutes of Health, Bethesda, Maryland).

### 2.7. Dual-Luciferase Reporter Assay

SUCNR1 promoter region was cloned into the pGL3-basic luciferase reporter gene vector (GeneCreate, China) in order to construct the SUCNR1 dual-luciferase reporter gene vector and the mutant plasmid with the IRF1 binding site: pGL3-basic-SUCNR1-WT and pGL3-basic-SUCNR1-MUT. Next, the aforementioned vectors were transfected with oe-IRF1 (100 ng) and NC (100 ng) into 293T cells. After 24 h of transfection, the luciferase activity was determined with the help of a Dual-Luciferase Reporter Assay System (E1910, Promega, Madison, WI).

### 2.8. Cell Counting Kit-8 (CCK-8) Assay

Cells were seeded into 96-well plates (1 × 10^3^ cells/well) for 1-5 days. Specifically, the cells were treated for 12, 24, 48, and 96 h, whereupon 10 *μ*L of CCK-8 solution was supplemented into each well of the plate for 1 h of incubation. The optical density (OD) values were tested at 450 nm at the designated time intervals using a microplate reader. Cell viability was assessed with the help of CCK-8 kits (K1018, Apexbio) with the formula of cell viability = (OD values_sample group_ − OD values_blank group_)/(OD values_untreated group_ − OD values_blank group_) [25, 26]. The blank group was indicative of the OD value of the CCK-8 working buffer without cells.

### 2.9. Transwell Assay

A Transwell chamber (pore size of 8 mm; Corning Incorporated, Corning, NY) in 24-well plates was adopted for cell migration and invasion (utilizing chamber precoated with Matrigel) determination in accordance with previously published literature [27]. Afterwards, observation was carried out using an inverted fluorescence microscope (TE2000, Nikon, China) in 5 randomly selected fields to count the migrated and invaded cells, with the mean value obtained.

### 2.10. Transmission Electron Microscope (TEM)

CRC cells in each group were fixed with 2.5% glutaraldehyde and 2% paraformaldehyde overnight. The following day, the cells were fixed with 2% osmium tetroxide (1 h), stained with 2% uranyl acetate (1 h), dehydrated in a concentration gradient of acetone, then embedded in epoxy resin, and sliced into semithin sections. Subsequently, the semithin sections were subjected to toluidine blue staining to locate the cells. The cells were finally observed under a TEM (Hitachi, Tokyo, Japan) at 80 nm.

### 2.11. Chromatin Immunoprecipitation (ChIP)

ChIP assay was carried out with the help of a SimpleChIP® Enzymatic Chromatin IP Kit (Magnetic Beads) (#9003, Cell Signaling Technology, Beverly, MA) [28]. In brief, upon attaining 95% confluence, 3 × 10^7^ cells were cross-linked with 1% formalin at ambient temperature (10 min), and the reaction was halted with the addition of glycine. Following ultrasonic treatment, 200-300 bp chromatin fragments were produced. Next, the chromatin lysate was immunoprecipitated with 5 *μ*g of histone antibody, 5 *μ*g of IRF1 antibody, and 5 *μ*g of normal IgG at 4°C overnight and then immunoprecipitated further with 30 *μ*L of protein G magnetic beads for 2 h. The extracted immunoprecipitated DNA and SUCNR1 primers (F: TTGTCATTCAATTGCAAAACTGC; R: TCAGTCAAACCTCCCAGTCA) were utilized for RT-qPCR detection.

### 2.12. Immunofluorescence Assay

Cells were cultured on a confocal petri dish, fixed with 95% ethanol (15 min) and treated with 3% Triton X-100. Following rinsing with cold PBS, the cells were reacted with 5% BSA to block nonspecific staining and then immunostained with the specific primary antibody to LC3 (ab239416, 0.1 *μ*g/mL, Abcam) overnight in conditions void of light (4°C). The following day, the cells were incubated with fluorescent secondary antibody (ab150081, 1 : 500, Abcam) at 37°C (dark conditions, 2 h) and stained with 4′,6-diamidino-2-phenylindole for 15 min at ambient temperature, followed by observation under a confocal scanning microscope (LSM 700; Carl Zeiss, Oberkochen, Germany).

### 2.13. Flow Cytometry

The Annexin V-fluorescein isothiocyanate/propidium iodide (FITC/PI) double staining method was utilized for cell apoptosis detection. Firstly, CRC cells were seeded in a 6-well plate (2 × 10^5^ cells/well). After 48 h of transfection, the cells were rinsed with precooled PBS at 4°C, trypsinized, and centrifuged at 800 g. Next, the pellets were rinsed twice with PBS, resuspended in 500 *μ*L binding buffer, and reacted with 5 *μ*L Annexin V-FITC and 5 *μ*L PI for 15 min following the guidelines of the Annexin V-FITC Apoptosis Detection Kit *Ι* (BD Biosciences, San Jose, CA). Afterwards, cell apoptosis was tested using a flow cytometer (FACSCalibur; BD Biosciences).

### 2.14. Xenograft Tumor in Nude Mice and Liver Metastasis Model Construction

Eighty female-specific pathogen-free BALB/c nude mice (aged 4 weeks, 401, procured from Vital River Laboratory Animal Technology Co., Ltd., Beijing, China) were housed in a laboratory at 22-25°C with 60-65% humidity under a 12 h light/dark cycle and allowed *ad libitum* access to food and water. The mice were allowed to acclimatize in the aforementioned conditions for a duration of one week. Afterwards, the mice were inoculated with CRC cells pretreated with sh-NC and sh-SUCNR1 (*n* = 10 for mice upon each treatment). Approximately 5 × 10^6^ cells/0.2 mL PBS were inoculated subcutaneously into nude mice, followed by observation of tumor growth in mice. When the tumor was visible to the naked eyes, the tumor volume was assessed with digital calipers every 3 days and calculated: V = 1/2 × length × width^2^. The mice were euthanized 16 days later, whereupon the tumor tissue was removed for subsequent experimentation.

For *in vivo* liver metastasis experiments, CRC cells (5 × 10^6^ cells/0.2 mL PBS) were injected into mice *via* tail vein. After 8 weeks, the liver was excised, paraffin-embedded, and subjected to hematoxylin and eosin (HE) staining to detect tumor metastasis in the liver.

### 2.15. HE Staining

Paraffin-embedded tissue sections were treated with xylene I and xylene II (each for 10 min) and rehydrated with descending series of alcohol (absolute ethanols I and II, 95%, 90%, 80%, and 70%, 5 min for each). Following washing with distilled water, the sections were dewaxed, hydrated, stained by immersion in Harris hematoxylin for 3-8 min, treated with 1% hydrochloric acid alcohol for several seconds, and blued in 0.6% ammonia water. Following rinsing under running water, the sections were then counterstained with eosin for 1-3 min, dehydrated in ascending series of alcohol, cleared in xylene, and dried and mounted with neutral gum. Finally, the pathologic conditions were assessed using a microscope (Nikon, TE200).

### 2.16. Immunohistochemistry

Paraffin tissue sections were dewaxed, hydrated, and treated with 3% H_2_O_2_. Next, the sections were heated in 10 mM sodium citrate (pH = 6.0) for 30 min, blocked with 10% normal goat serum for 15 min, and immunolabeled with primary antibody against Ki67 (ab15580, 1 : 1000, Abcam). Following three PBS rinses, the tissue sections were reacted with secondary goat antirabbit IgG (ab6721, 1 : 5000, Abcam) for 30 min. Subsequently, the sections were treated with streptavidin-biotin complex (Vector Labs, Burlingame, CA) in a 37°C incubator (30 min), developed with DAB (P0203, Beyotime) (6 min), and then stained with hematoxylin (30 s). Afterwards, observation was carried out with an upright microscope (BX63, Olympus Optical Co., Ltd., Tokyo, Japan).

### 2.17. Statistical Analysis

All experimental data were processed using the GraphPad Prism 8 software (GraphPad Software, La Jolla, CA). Measurement data from three independent experiments were depicted as mean ± standard deviation. Data between CRC tissues and adjacent normal tissues were analyzed utilizing a paired *t*-test, while those between the other two groups were analyzed by means of an unpaired *t*-test. Analysis of data among multiple groups was processed utilizing one-way analysis of variance (ANOVA). Bonferroni-corrected repeated measure ANOVA was adopted to compare data among multiple groups at different time points. A value of *p* < 0.05 was regarded as statistically significant.

## 3. Results

### 3.1. Bioinformatics Analyses Predict that HDAC8 Participates in the Growth and Metastasis of CRC via Regulation of the IRF1/SUCNR1 Axis

**Figure 1:**
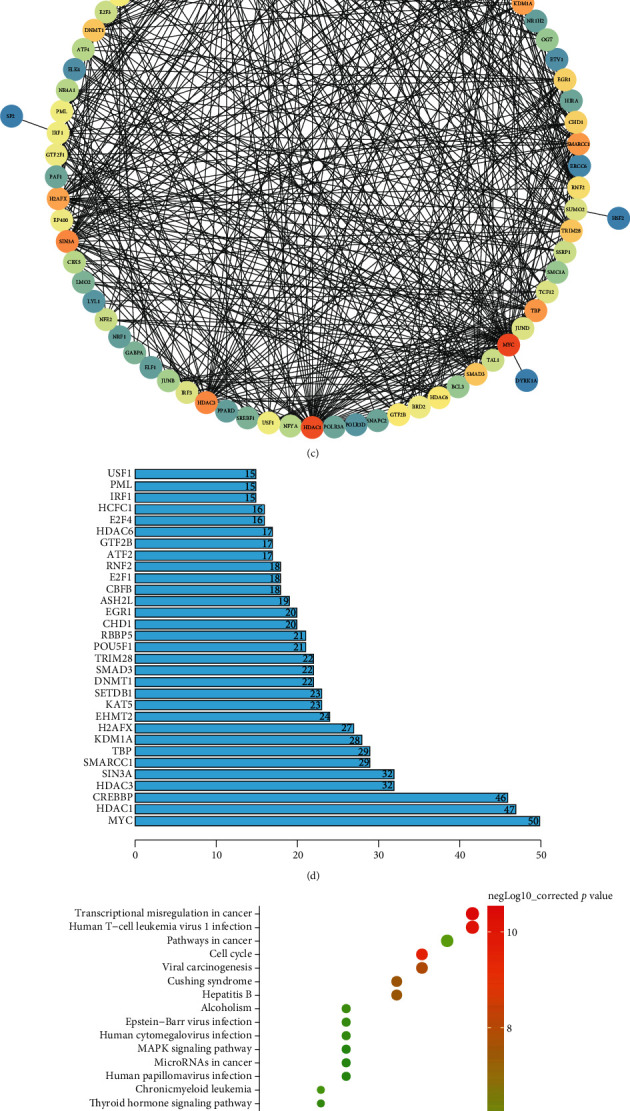
Significance of the HDAC8/IRF1/SUCNR1 axis in CRC. (a) A box plot of the differential expression of HDAC8 in the colon adenocarcinoma (COAD) and rectum adenocarcinoma (READ) samples included in TCGA and GTEx (red box plots represent tumor samples, and gray box plots represent normal samples; in COAD, there are 275 tumor samples and 349 normal samples; in READ, there are 92 tumor samples and 318 normal samples). (b) Venn diagram of HDAC8 downstream regulatory genes and transcription factors (the left is the downstream genes of HDAC8 predicted by the starBase database, the right is the transcription factor annotation, and the center represents the intersection of the two sets of data). (c) Interaction analysis of the candidate transcription factors; each circle in the figure represents a gene, and the line between circles indicates interaction between two genes; the darker color of the circle where the gene is located reflects more interaction genes, higher core degree in the interaction network, and higher degree value. (d) Statistics of degree value of core genes in the gene interaction network (the abscissa represents the degree value and the ordinate represents the gene name). (e) KEGG enrichment analysis of the candidate transcription factors (the abscissa represents the gene ratio, the ordinate represents the KEGG entry identifier, and the histogram on the right is the color scale). (f) A box plot of the differential expression of SUCNR1 in the CRC included in TCGA and GTEx (red box plots represent tumor samples, and gray box plots represent normal samples; in COAD, there are 275 tumor samples and 349 normal samples; in READ, there are 92 tumor samples and 318 normal samples). ^∗^*p* < 0.05.

There is much evidence to suggest that downregulation of the HDAC8 expression can inhibit the growth of CRC cells [14, 29], yet the specific mechanism of HDAC8 influencing the growth of CRC cells remains unknown. Therefore, we aimed to investigate the underlying mechanism of HDAC8 in CRC progression. Retrieval of HDAC8 expression patterns in CRC included in TCGA and GTEx from the GEPIA database revealed the presence of elevated HDAC8 in colon adenocarcinoma and rectum adenocarcinoma (Figure 1(a)). The downstream regulatory factors of HDAC8 were predicted using the ChIPBase database (Supplementary Table 2) and then intersected with the transcription factors obtained from the Cistrome database, with 79 candidate target genes found at the intersection (Figure 1(b)). Interaction analysis was subsequently carried out on the abovementioned 79 candidate genes, and the degree value of the core genes was counted (Figures 1(c) and 1(d)). The results of KEGG enrichment analysis revealed that these 79 candidate genes were primarily enriched in the TNF signaling pathway (Figure 1(e)). Among the 79 transcription factors, IRF1 was not only at the core in the gene interaction network but also enriched in the famous tumor pathway TNF, as detected by KEGG enrichment analysis. Existing literature further indicates that the TNF signaling pathway shares close correlation with CRC [30]. On the other hand, IRF1 is known to be capable of limiting the growth and metastasis of CRC cells [31, 32]. Accordingly, we speculated that HDAC8 may regulate IRF1 to affect CRC cell growth and metastasis.

The potential downstream regulatory genes of IRF1 were then retrieved from the hTFtarget database, with the results showing the presence of multiple binding sites between IRF1 and the promoter region of the SUCNR1 gene (Supplementary Table 3). Moreover, elevated SUCNR1 was documented in CRC samples included in TCGA and GTEx (Figure 1(f)). Altogether, these findings provided evidence suggesting that HDAC8 may participate in the growth and metastasis of CRC *via* modulation of IRF1 and SUCNR1.

### 3.2. HDAC8 Downregulates IRF1 to Promote the Growth and Metastasis of CRC

**Figure 2:**
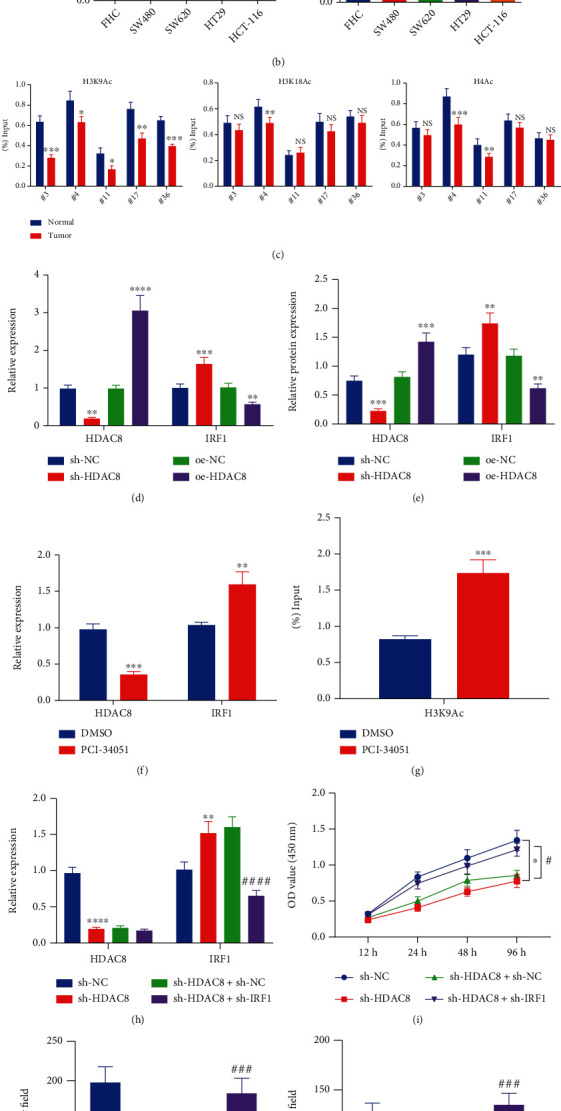
HDAC8 suppresses the expression of IRF1 and thus facilitates the growth and metastasis of CRC cells. (a) HDAC8 and IRF1 mRNA expression in CRC and adjacent normal tissues determined by RT-qPCR (*n* = 58). (b) HDAC8 and IRF1 mRNA expression in SW480, SW620, HT29, HCT-116, and FHC cell lines determined by RT-qPCR. (c) Histone acetylation levels in the IRF1 promoter region in CRC and adjacent normal tissues determined by ChIP. (d) mRNA expression of HDAC8 and IRF1 in HCT-116 cells treated with sh-HDAC8 or oe-HDAC8 determined by RT-qPCR. (e) Western blot analysis of HDAC8 and IRF1 proteins in HCT-116 cells treated with sh-HDAC8 or oe-HDAC8. (f) mRNA expression of HDAC8 and IRF1 in HCT-116 cells treated with PCI-34051 determined by RT-qPCR. (g) H3K9Ac levels in the IRF1 promoter region in HCT-116 cells treated with PCI-34051 determined by ChIP. (h) HDAC8 and IRF1 mRNA expression in HCT-116 cells treated with sh-HDAC8 or combined with sh-IRF1 determined by RT-qPCR. (i) Viability of HCT-116 cells treated with sh-HDAC8 or combined with sh-IRF1 measured by CCK-8 assay. (j) Migration of HCT-116 cells treated with sh-HDAC8 or combined with sh-IRF1 measured by Transwell assay. (k) Invasion of HCT-116 cells treated with sh-HDAC8 or combined with sh-IRF1 measured by Transwell assay. ^∗^*p* < 0.05, ^∗∗^*p* < 0.01, ^∗∗∗^*p* < 0.001, and ^∗∗∗∗^*p* < 0.0001, compared with adjacent normal tissues, FHC cells, or DMSO- or sh-NC-treated HCT-116 cells. ^#^*p* < 0.05, ^###^*p* < 0.001, and ^####^*p* < 0.0001, compared with sh-HDAC8+sh-NC-treated HCT-116 cells. The experiment was conducted three times independently.

To further validate the relationship between HDAC8 and IRF1, we subsequently assessed the expression patterns of HDAC8 and IRF1 in the CRC tissues collected from 58 patients with CRC by means of RT-qPCR. Increased HDAC8 expression levels and decreased IRF1 expression levels were detected in CRC tissues (Figure 2(a), vs. adjacent normal tissues). Similar trends were also documented in the CRC cell lines (SW480, SW620, HT29, and HCT-116) compared to the normal colorectal epithelial cells (FHC). Specifically, the HCT-116 cell line exhibited the highest HDAC8 and the lowest IRF1 expression levels (Figure 2(b)) and was thus utilized for subsequent experimentation.

Furthermore, detection of the histone acetylation levels in the IRF1 promoter region in CRC tissue depicted the presence of lower levels of H3K9Ac in CRC tissues (Figure 2(c)). In addition, previously published literature indicates that knockdown of HDAC8 significantly increases the level of acetylated histone H3K9Ac relative to other lysine sites of histones [33]. Accordingly, we tested their interaction using different treatment regimens. As expected, silencing of HDAC8 in HCT-116 cells reduced HDAC8 expression levels and elevated those of IRF1. Opposite trends were observed in the presence of HDAC8 overexpression (Figures 2(d) and 2(e)). After 24 h of treatment with HDAC8-specific inhibitor PCI-34051 [24], there was a decline in HDAC8 expression levels and an increase in those of IRF1 in response to treatment with PCI-34051 (Figure 2(f)). Moreover, ChIP results illustrated that the acetylation levels of H3K9Ac were enhanced in HCT-116 cells following PCI-34051 treatment (Figure 2(g)). Together, these findings indicated that HDAC8 reduced IRF1 expression *via* regulation of the IRF1 acetylation level in CRC cells.

Additionally, the results of RT-qPCR revealed a decline in HDAC8 expression levels and an increase in those of IRF1 in sh-HDAC8-treated HCT-116 cells. In addition, lower expression levels of IRF1 were observed in the presence of concomitant silencing of HDAC8 and IRF1 compared to individual HDAC8 silencing (Figure 2(h)). Besides, the viability, migratory, and invasive potentials of HCT-116 cells were all attenuated in the presence of sh-HDAC8 treatment, whereas opposing trends were observed following further sh-IRF1 treatment (Figures 2(i)–2(k), Supplementary Figure 1A, 1B). Altogether, these findings highlighted that HDAC8 limited IRF1 expression to promote the growth and metastasis of CRC.

### 3.3. IRF1 Binds to the SUCNR1 Promoter to Decline SUCNR1 Expression

**Figure 3:**
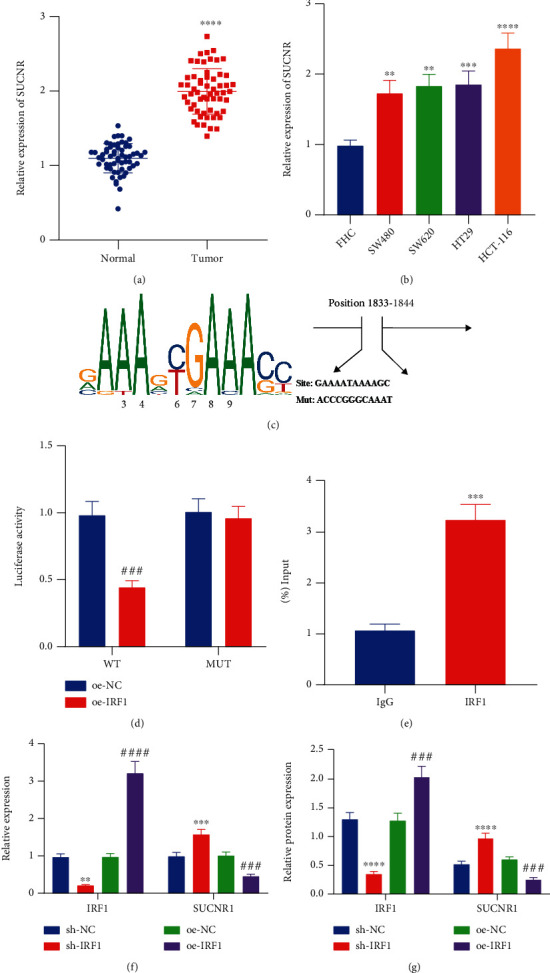
IRF1 downregulates the expression of SUCNR1 by binding to its promoter in CRC cells. (a) SUCNR1 mRNA expression in CRC and adjacent normal tissues determined by RT-qPCR (*n* = 58). (b) SUCNR1 mRNA expression in SW480, SW620, HT29, HCT-116, and FHC cell lines determined by RT-qPCR. (c) Prediction of IRF1 binding sites in the SUCNR1 promoter and mutation sequence generated by site mutation determined by ChIP. (d) Binding between IRF1 and SUCNR1 confirmed by dual-luciferase reporter assay. (e) Enrichment of IRF1 in the promoter of SUCNR1 determined by ChIP. (f) IRF1 and SUCNR1 mRNA expression in HCT-116 cells treated with sh-IRF1 or oe-IRF1 determined by RT-qPCR. (g) Western blot analysis of IRF1 and SUCNR1 proteins in HCT-116 cells treated with sh-IRF1 or oe-IRF1. ^∗∗^*p* < 0.01, ^∗∗∗^*p* < 0.001, and ^∗∗∗∗^*p* < 0.0001, compared with adjacent normal tissues, FHC cells, IgG group, or sh-NC-treated HCT-116 cells. ^###^*p* < 0.001 and ^####^*p* < 0.0001, compared with oe-NC-treated HCT-116 cells. The experiment was conducted three times independently.

Thereafter, we sought to determine the downstream regulatory factor of IRF1 in CRC. Retrieval of downstream regulatory genes of IRF1 from the hTFtarget database indicated that IRF1 may serve as a transcription factor to modulate SUCNR1 expression. Subsequent results of RT-qPCR displayed that SUCNR1 was increased in both CRC tissues (Figure 3(a), vs. adjacent normal tissues) as well as in CRC cell lines (Figure 3(b), vs. FHC cell line). The hTFtarget database further predicted the binding sites of IRF1 in the promoter region of SUCNR1 (Figure 3(c)). Accordingly, a dual-luciferase reporter assay was carried out, the results of which verified that luciferase activity of PGL3-basic-SUCNR1-WT was reduced, while that of PGL3-basic-SUCNR1-MUT remained unchanged in the presence of oe-IEF1 (Figure 3(d)). Moreover, ChIP results illustrated increased enrichment of IRF1 in the SUCNR1 promoter (Figure 3(e)). In addition, the expression levels of IRF1 were decreased, while those of SUCNR1 were increased in HCT-116 cells treated with sh-IRF1, while opposing trends were observed following oe-IRF1 treatment (Figures 3(f) and 3(g)). Taken together, these findings indicated that IRF1 could bind to the SUCNR1 promoter to reduce SUCNR1 expression.

### 3.4. SUCNR1 Stimulates the Malignant Features of CRC Cells by Inhibiting Tumor Cell Autophagy

**Figure 4:**
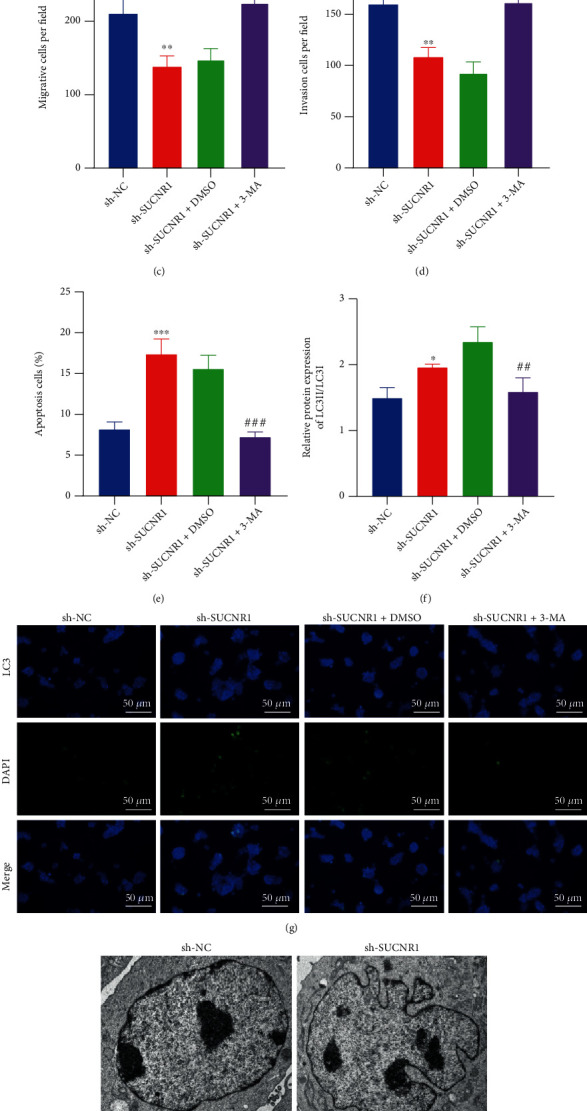
SUCNR1 promotes the migration and invasion of CRC cells by blunting tumor cell autophagy. (a) SUCNR1 mRNA expression in HCT-116 cells treated with sh-SUCNR1 determined by RT-qPCR. (b) Viability of HCT-116 cells following SUCNR1 knockdown or combined with 3-MA measured by CCK-8 assay. (c) Migration of HCT-116 cells following SUCNR1 knockdown or combined with 3-MA measured by Transwell assay. (d) Invasion of HCT-116 cells following SUCNR1 knockdown or combined with 3-MA measured by Transwell assay. (e) Flow cytometric analysis of the HCT-116 cell apoptosis following SUCNR1 knockdown or combined with 3-MA. (f) Western blot analysis of LC3-II/LC3-I ratio in HCT-116 cells following SUCNR1 knockdown or combined with 3-MA. (g) Immunofluorescence detection of the number of GFP-LC3 spots in HCT-116 cells following SUCNR1 knockdown or combined with 3-MA (scale bar = 50 *μ*m). (h) Number of autophagic vacuoles in HCT-116 cells following SUCNR1 knockdown or combined with 3-MA under a TEM. ^∗^*p* < 0.05, ^∗∗^*p* < 0.01, and ^∗∗∗^*p* < 0.001, compared with HCT-116 cells transfected with sh-NC. ^#^*p* < 0.05, ^##^*p* < 0.01, and ^###^*p* < 0.001, compared with HCT-116 cells treated with sh-SUCNR1+DMSO. The experiment was conducted three times independently.

To further elucidate the effects of SUCNR1 on CRC cell migration and invasion, we first transfected shRNA sequences against SUCNR1 in HCT-116 cells to silence SUCNR1 expression. The transfection efficiency was subsequently validated by means of RT-qPCR (Figure 4(a)). It was found that the viability, migratory, and invasive abilities of HCT-116 cells were all inhibited following sh-SUCNR1 treatment (Figures 4(b)–4(d), Supplementary Figure 1C, 1D). Flow cytometric analysis results demonstrated that there was an increase in the apoptosis of sh-SUCNR1-treated HCT-116 cells (Figure 4(e)). Overall, silencing of SUCNR1 exerted a limiting effect on the migratory and invasive potentials of CRC cells.

The abovementioned findings suggested that HDAC8 downregulated the expression of IRF1, which in turn brought about a reduction in the expression of SUCNR1. Previously published literature has further confirmed that HDAC inhibitors can inhibit the malignant features of cancer cells by inducing autophagy; meanwhile, IRF1 is also known to suppress the growth of human hepatocellular carcinoma cells by inducing autophagy [34, 35]. Accordingly, we speculated that the HDAC8/IRF1/SUCNR1 axis may be implicated in CRC growth and metastasis *via* mediation of autophagy. To ascertain the same, we administered the autophagy inhibitor 3-MA in HCT-116 cells. Subsequent results of Western blot assay showed that the ratio of LC3-II/LC3-I was increased in HCT-116 cells following sh-SUCNR1 treatment, while being reduced after further 3-MA treatment (Figure 4(f)). Immunofluorescence detection results further illustrated that knockdown of SUCNR1 augmented the number of GFP-LC3 spots; however, further treatment with 3-MA led to a reduction in the number of GFP-LC3 spots (Figure 4(g)). Meanwhile, the number of autophagic vacuoles were increased following SUCNR1 knockdown, whereas opposite trends was observed following further treatment with 3-MA, by means of TEM (Figure 4(h)). The latter findings validated that knockdown of SUCNR1 could induce CRC cell autophagy. Furthermore, enhanced HCT-116 cell viability, migratory and invasive potentials, and diminished cell apoptosis were detected in the presence of sh-SUCNR1+3-MA than sh-SUCNR1+DMSO (Figures 4(b)–4(e)). All in all, these findings indicated SUCNR1 could induce the malignant features of CRC cells via suppression of tumor cell autophagy.

### 3.5. HDAC8 Downregulates IRF1 and Upregulates SUCNR1 to Inhibit Autophagy and Promote CRC Cell Malignant Properties

**Figure 5:**
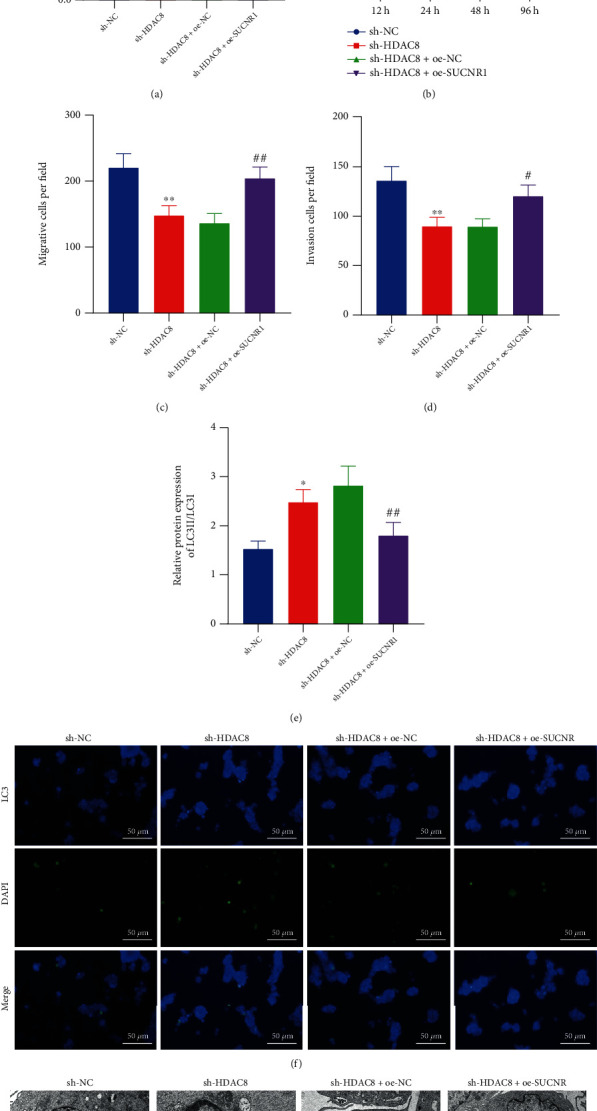
HDAC8 suppresses cell autophagy to boost the migration and invasion of CRC cells by regulating the IRF1/SUCNR1 axis. HCT-116 cells were transfected with sh-HDAC8 or combined with oe-SUCNR1. (a) SUCNR1 mRNA expression in HCT-116 cells determined by RT-qPCR. (b) Viability of HCT-116 cells measured by CCK-8 assay. (c) Migration of HCT-116 cells measured by Transwell assay. (d) Invasion of HCT-116 cells measured by Transwell assay. (e) Western blot analysis of LC3-II/LC3-I ratio in HCT-116 cells. (f) Immunofluorescence detection of the number of GFP-LC3 spots in HCT-116 cells (scale bar = 50 *μ*m). (g) Number of autophagic vacuoles in HCT-116 cells under a TEM. ^∗^*p* < 0.05 and ^∗∗^*p* < 0.01, compared with HCT-116 cells transfected with sh-NC. ^#^*p* < 0.05, ^##^*p* < 0.01, and ^####^*p* < 0.0001, compared with HCT-116 cells transfected with sh-HDAC8+oe-NC. The experiment was conducted three times independently.

Thereafter, we sought to examine the effects of HDAC8 regulating the IRF1/SUCNR1 axis on the growth and metastasis of CRC. As depicted by the results of RT-qPCR, reduced SUCNR1 levels were detected upon sh-HDAC8 treatment, while being enhanced following further overexpression of SUCNR1 (Figure 5(a)). Moreover, the viability, migration, and invasiveness of HCT-116 cells were all inhibited in response to sh-HDAC8, whereas opposing trends were observed after additional overexpression of SUCNR1 (Figures 5(b)–5(d)).

Furthermore, the results of Western blot assay demonstrated an elevation in the LC3-II/LC3-I ratio upon sh-HDAC8, whereas opposing trends were observed following both HDAC8 silencing and SUCNR1 overexpression (Figure 5(e)). As shown in Figures 5(f) and 5(g), the number of both GFP-LC3 spots and autophagic vacuoles was increased upon HDAC8 silencing, while contrary results were documented in the presence of both HDAC8 silencing and SUCNR1 overexpression. Together, these findings indicated that HDAC8 may limit autophagy and promote the growth and metastasis of CRC by modulation of the IRF1/SUCNR1 axis.

### 3.6. HDAC8 Enhances Tumorigenesis and Liver Metastasis of CRC Cells by Regulating the IRF1/SUCNR1 Axis In Vivo

**Figure 6:**
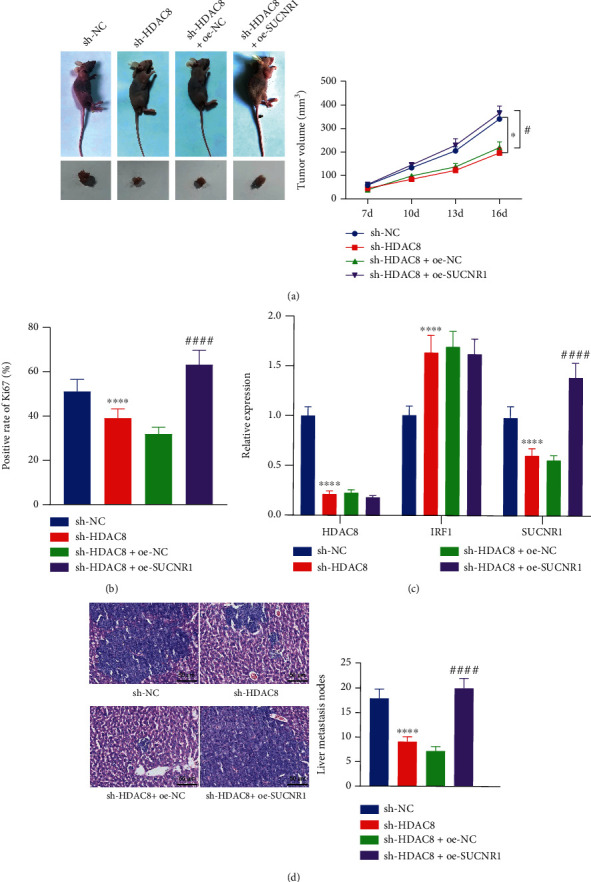
HDAC8 promotes tumorigenesis and liver metastasis of CRC cells by regulating the IRF1/SUCNR1 axis in nude mice. (a) Tumor growth of mice treated with sh-HDAC8 or combined with oe-SUCNR1. (b) Ki67 immunohistochemical staining images of tumor tissues of nude mice treated with sh-HDAC8 or combined with oe-SUCNR1 as well as the semiquantitative analysis. (c) mRNA expression of HDAC8, IRF1, and SUCNR1 in tumor tissues of mice treated with sh-HDAC8 or combined with oe-SUCNR1 determined by RT-qPCR. (d) HE staining analysis of number of liver metastases in the liver tissues of nude mice treated with sh-HDAC8 or combined with oe-SUCNR1 (scale bar = 50 *μ*m). *n* = 10 for mice upon each treatment. ^∗^*p* < 0.05 and ^∗∗∗∗^*p* < 0.0001, compared with mice treated with sh-NC. ^#^*p* < 0.05 and ^####^*p* < 0.0001, compared with mice treated with sh-HDAC8+oe-NC.

Lastly, we proceeded to elucidate the effect of HDAC8 regulating the IRF1/SUCNR1 axis on the growth and metastasis of CRC *in vivo*. As depicted in Figures 6(a) and 6(b), HDAC8 silencing led to a reduction in tumor size and Ki67 expression levels, while further overexpression of SUCNR1 brought about the opposite trends. RT-qPCR results further depicted reduced expression levels of HDAC8 and SUCNR1 and enhanced IRF1 expression levels in the tumor tissues of mice treated with sh-HDAC8, whereas SUCNR1 expression levels were enhanced, and those of HDAC8 and IRF1 did not change after further oe-SUCNR1 treatment (Figure 6(c)). Overall, these findings indicated that HDAC8 could downregulate IRF1 and then upregulate SUCNR1 to promote the development of CRC *in vivo*.

The stably transfected HCT-116 cells (5 × 10^6^ cells/0.2 mL PBS) were injected into nude mice *via* the tail vein to construct an *in vivo* liver metastasis model. After 8 weeks, the liver tissues were removed and adopted for HE staining to count the number of liver metastases. The results showed that compared with the sh-NC group, the number of liver metastases in the sh-HDAC8 group was significantly reduced; meanwhile, compared with the sh-HDAC8+oe-NC group, the number of liver metastases was significantly increased in the sh-HDAC8+oe-SUCNR1 group (Figure 6(d)). Collectively, the aforementioned findings supported that HDAC8 may reduce IRF1 and elevate SUCNR1, ultimately inducing the tumorigenesis and liver metastasis of CRC cells in nude mice.

## 4. Discussion

The hard-done work of our peers has shown that HDAC inhibition exerts potent anticancer effects against CRC and further serves as a promising therapeutic modality in CRC treatment [36]. Our obtained findings clarified that HDAC8 could potentially limit IRF1 expression, augment SUCNR1 expression, and thereby promote the growth and metastasis of CRC cells both *in vitro* and *in vivo*, thus inducing the occurrence of CRC.

Initial findings in our study demonstrated that HDAC8 was highly expressed in CRC tissues and cells, and further, HDAC8 could induce CRC by downregulation of IRF1. On the other hand, inhibition of HDAC8 was previously shown to exert a diminishing effect on the growth of HT-29 and HCT-116 cell lines, while enhancing their apoptosis [14]. In addition, a prior study documented that HDAC8 silencing brought about a marked reduction in the proliferation and colony formation of SW1116csc [29]. Meanwhile, existing evidence further indicates that HDAC is capable of negatively regulating IRF1 [37, 38]. Expanding our understanding of the latter, the current study is the first of its kind to reveal that HDAC8 downregulate IRF1 *via* regulation of IRF1 acetylation levels in CRC cells. Further in line with our data, Hong et al. illustrated that IRF1 is poorly expressed in CRC relative to normal mucosa, whereas IRF1 overexpression could suppress the malignant features of CRC cells *in vivo* and *in vitro* [32]. Similarly, repressed IGF1 by the IFN-*γ*-mediated IRF1/miR-29b feedback loop inhibits CRC cell growth and metastasis [31]. Together, the aforementioned findings and evidence indicate that targeting the HDAC8/IRF1 axis may represent a novel strategy for delaying CRC growth and metastasis.

Additional analyses in our study unveiled that IRF1 could bind to the SUCNR1 promoter and further induce the inhibition of SUCNR1 expression. Meanwhile, it is well-established that gene expression is modulated by the specific binding of protein transcription factors to the binding sites of cis-regulatory transcription factor in the promoter regions of genes [39]. In addition, cancer cells are known to deliver succinate into the cancer microenvironment and consequently trigger SUCNR1 to polarize macrophages into tumor-associated macrophages, thus stimulating cancer cell malignant behaviors, as well as augmenting cancer metastasis [40]. Herein, our findings were in accordance with the latter, such that SUCNR1 stimulated the growth and metastasis of CRC cells, which was associated with inhibition of tumor cell autophagy. Autophagy represents a fundamental process that maintains cell survival and function by degrading organelles and proteins [41]. More importantly, autophagy exerts critical influence on the development and progression of CRC by either promoting tumor growth and cell survival or inducing tumor suppression and cell death, both of which involve complex regulatory networks [42]. Besides, the upregulation of LC3-II, an autophagy-related protein, is indicative of autophagy induction [43, 44]. Interestingly, our findings revealed an enhancement in the LC3-II/LC3-I ratio in HCT-116 cells in the absence of SUCNR1, which underscores that SUCNR1 may serve as an inhibitor to suppress cell autophagy. It is also noteworthy that HDAC inhibitors are known to limit the growth of cancer cells and trigger their apoptosis by virtue of inducing autophagy [45, 46], while there is further evidence to suggest that IRF1 can inhibit the growth of hepatocellular carcinoma cells by inducing autophagy [35]. Consistently, we documented that HDAC8 inhibited autophagy and eventually promoted CRC cell malignant properties *in vitro*, along with enhanced tumorigenesis and liver metastasis of CRC cells *in vivo* by downregulating IRF1 and upregulating SUCNR1. In lieu of the same, it would be plausible to suggest that targeting the HDAC8/IRF1/SUCNR1 signaling may provide novel insights into the molecular mechanisms of CRC progression. However, the correlation between HDAC8 and SUCNR1 has not been elucidated yet, and further studies are warranted to validate this application.

## 5. Conclusion

**Figure 7:**
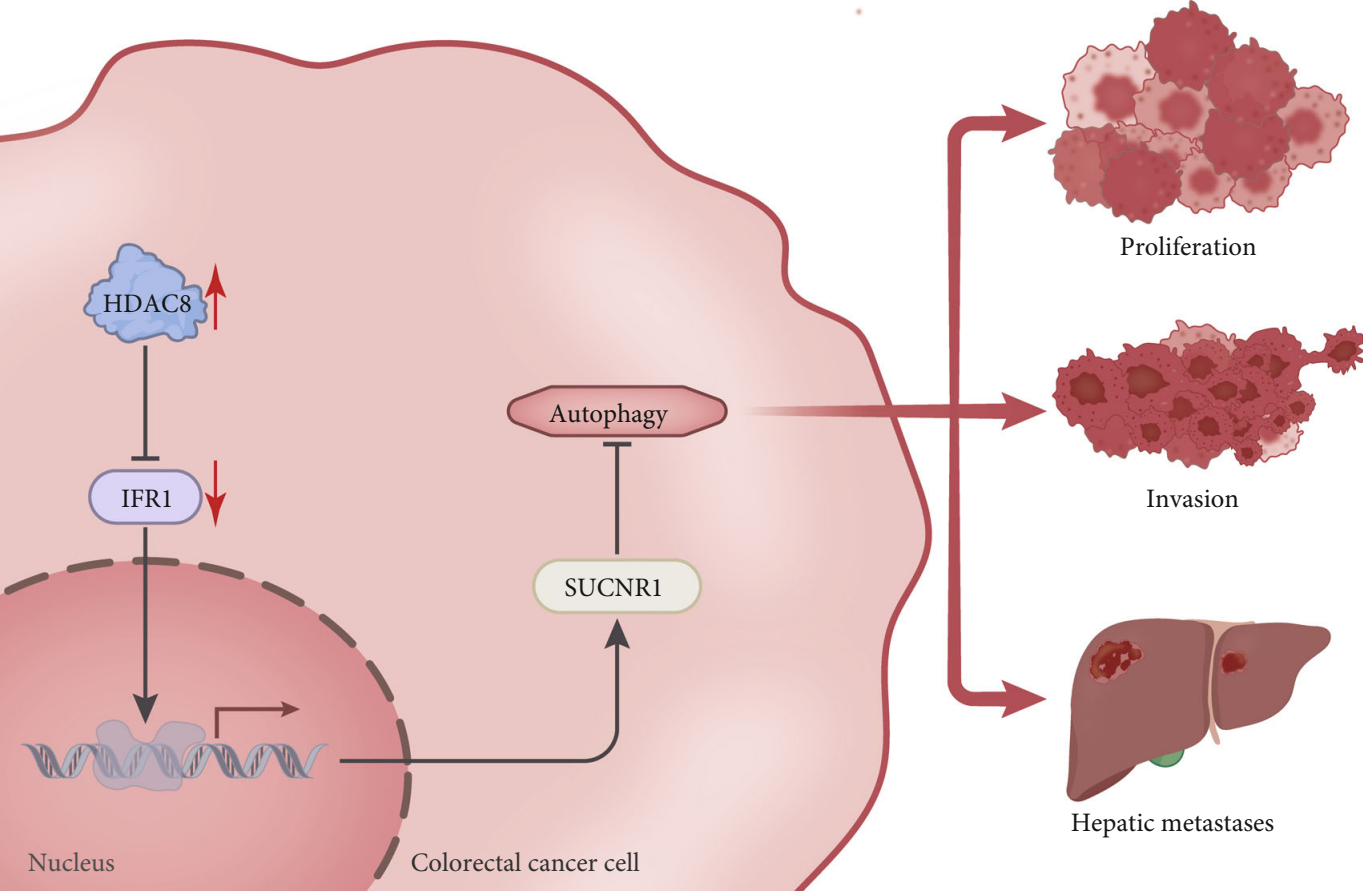
Molecular mechanism by which HDAC8 regulates the progression of CRC. Histone deacetylase HDAC8 upregulates SUCNR1 by downregulating IRF1 and consequently inhibits CRC cell autophagy, ultimately contributing to the CRC growth and liver metastasis.

In summary, the current study highlighted that HDAC8 can attenuate the IRF1-induced SUCNR1 inhibition and diminish CRC cell autophagy, thus promoting the growth and liver metastasis of CRC (Figure 7). These findings shed a light on the unrecognized roles of the HDAC8/IRF1/SUCNR1 signaling axis in the progression of CRC and offer new prognostic markers and/or effective therapeutic targets in CRC.

## Data Availability

The data underlying this article will be shared on reasonable request to the corresponding authors.

## Supplementary Materials

Supplementary 1 Table S1: primer sequences for RT-qPCR.

Supplementary 2 Table S2: the downstream regulatory factors of HDAC8 were predicted by the ChIPBase database.

Supplementary 3 Table S3: multiple binding sites between IRF1 and the promoter region of SUCNR1 gene.

Supplementary 4 Figure S1: representative images of Transwell assay.
