# Supplementary material for: HDAC8 Promotes Liver Metastasis of Colorectal Cancer via Inhibition of IRF1 and Upregulation of SUCNR1
Source: Oxid Med Cell Longev. 2022 Aug 16;2022:2815187. doi: 10.1155/2022/2815187 (PMC9400431; doi:10.1155/2022/2815187)
Supplement: Supplementary 1 — Table S1: primer sequences for RT-qPCR. [file 2815187.f1.docx]

**Supplementary Table 1** Primer sequences for RT-qPCR

| Gene | Sequences |
| --- | --- |
| HDAC8 (human) | Forward: 5’-TGGGCAGTCGCTGGT-3’ |
|  | Reverse: 5’-GTGGCTGGGCAGTCATAA-3’ |
| IRF1 (human) | Forward: 5’-AGGAGCCAGATCCCAAGACGTG-3’ |
|  | Reverse: 5’-AGCATCCGGTACACTCGCACAG-3’ |
| SUCNR1 (human) | Forward: 5’-TCAAGGGATCAAGTCTTCCAA-3’ |
|  | Reverse: 5’-CCAGCCAGTTTTTGCAAGTT-3’ |
| GAPDH (human) | Forward: 5’-AGAAGGCTGGGGCTCATTTG-3’ |
|  | Reverse: 5’-GCAGGAGGCATTGCTGATGAT-3’ |
