# Supplementary material for: HDAC8 Promotes Liver Metastasis of Colorectal Cancer via Inhibition of IRF1 and Upregulation of SUCNR1
Source: Oxid Med Cell Longev. 2022 Aug 16;2022:2815187. doi: 10.1155/2022/2815187 (PMC9400431; doi:10.1155/2022/2815187)
Supplement: Supplementary 2 — Table S2: the downstream regulatory factors of HDAC8 were predicted by the ChIPBase database. [file 2815187.f2.pdf]

##**Please cite: Zhou KR, Liu S, Sun WJ, Zheng LL, Zhou H, Yang JH, Qu LH. ChIPBase
##**Please cite: Yang JH, Li JH, Jiang S, Zhou H, Qu LH. ChIPBase: a database for d

##ChIPBase v2.0 project, hg38, All protein genes regulated by HDAC8 in sample HUMHG
##All coordiates in this table are one-based start coordinates
##About sample: cell line/tissue: K562, treatment: HDAC8, peak called: -

Gene ID	Gene Symb	Gene Chro	Gene Star	Gene End	Gene Stra	Gene Type	Relative
ENSG0000(	SCYL3		1			protein_coding	78
ENSG0000(	FUCA2		1			protein_coding	124
ENSG0000(	FUCA2		1			protein_coding	635
ENSG0000(	NFYA		1			protein_coding	-517
ENSG0000(	STPG1		1			protein_coding	-122
ENSG0000(	NIPAL3		1			protein_coding	-130
ENSG0000(	ANKIB1		1			protein_coding	406
ENSG0000(	KRIT1		1			protein_coding	-473
ENSG0000(	CFLAR		1			protein_coding	250
ENSG0000(	RBM5		1			protein_coding	-191
ENSG0000(	RBM5		1			protein_coding	308
ENSG0000(	POLDIP2		1			protein_coding	-119
ENSG0000(	POLDIP2		1			protein_coding	-420
ENSG0000(	KDM1A		1			protein_coding	-196
ENSG0000(	VPS50		1			protein_coding	-53
ENSG0000(	HSPB6		1			protein_coding	115
ENSG0000(	CDC27		1			protein_coding	351
ENSG0000(	UPF1		1			protein_coding	-251
ENSG0000(	AC004381.6		1			protein_coding	518
ENSG0000(	SPPL2B		1			protein_coding	-796
ENSG0000(	CREBBP		1			protein_coding	-405
ENSG0000(	KMT2E		1			protein_coding	-544
ENSG0000(	IBTK		1			protein_coding	-730
ENSG0000(	LAMP2		1			protein_coding	765
ENSG0000(	ITGA2B		1			protein_coding	-486
ENSG0000(	C19orf60		1			protein_coding	-177
ENSG0000(	C19orf60		1			protein_coding	-734
ENSG0000(	OSBPL7		1			protein_coding	-139
ENSG0000(	TMEM98		1			protein_coding	-351
ENSG0000(	TMEM98		1			protein_coding	795
ENSG0000(	CACNG3		1			protein_coding	443
ENSG0000(	KDM7A		1			protein_coding	-332
ENSG0000(	KDM7A		1			protein_coding	-702
ENSG0000(	ETV1		1			protein_coding	597
ENSG0000(	AGK		1			protein_coding	-27
ENSG0000(	TTC22		1			protein_coding	-325
ENSG0000(	PHTF2		1			protein_coding	-429
ENSG0000(	GGCT		1			protein_coding	-269
ENSG0000(	GGCT		1			protein_coding	758
ENSG0000(	PAF1		1			protein_coding	22
ENSG0000(	PNPLA4		1			protein_coding	-56
ENSG0000(	CDKL3		1			protein_coding	50
ENSG0000(	PRSS21		1			protein_coding	-57
ENSG0000(	MARK4		1			protein_coding	-68
ENSG0000(	CCDC124		1			protein_coding	-194
ENSG0000(	ST7L		1			protein_coding	875

ENSG0000(	RPUSD1	1	protein_coding	-351
ENSG0000(	LUC7L	1	protein_coding	27
ENSG0000(	PIGQ	1	protein_coding	-11
ENSG0000(	CRAMP1	1	protein_coding	-363
ENSG0000(	MYLIP	1	protein_coding	927
ENSG0000(	PSMB1	1	protein_coding	-571
ENSG0000(	SYN1	1	protein_coding	-283
ENSG0000(	CAMK1G	1	protein_coding	-904
ENSG0000(	NADK	1	protein_coding	-26
ENSG0000(	NADK	1	protein_coding	-819
ENSG0000(	NADK	1	protein_coding	475
ENSG0000(	NADK	1	protein_coding	995
ENSG0000(	MPND	1	protein_coding	-83
ENSG0000(	CRY1	1	protein_coding	-103
ENSG0000(	NFIX	1	protein_coding	-632
ENSG0000(	HEATR5B	1	protein_coding	-452
ENSG0000(	HEATR5B	1	protein_coding	-827
ENSG0000(	RPS20	1	protein_coding	-283
ENSG0000(	CSDE1	1	protein_coding	607
ENSG0000(	REV3L	1	protein_coding	-353
ENSG0000(	FAM76A	1	protein_coding	-350
ENSG0000(	VTA1	1	protein_coding	1
ENSG0000(	MLXIPL	1	protein_coding	865
ENSG0000(	BAZ1B	1	protein_coding	-394
ENSG0000(	RANBP9	1	protein_coding	-793
ENSG0000(	SPRTN	1	protein_coding	-791
ENSG0000(	SPRTN	1	protein_coding	877
ENSG0000(	METTL13	1	protein_coding	-538
ENSG0000(	METTL13	1	protein_coding	455
ENSG0000(	ZNF207	1	protein_coding	68
ENSG0000(	GIPR	1	protein_coding	-391
ENSG0000(	GIPR	1	protein_coding	376
ENSG0000(	NISCH	1	protein_coding	146
ENSG0000(	FUZ	1	protein_coding	-751
ENSG0000(	LRRC23	1	protein_coding	410
ENSG0000(	BTK	1	protein_coding	-169
ENSG0000(	HFE	1	protein_coding	453
ENSG0000(	SCMH1	1	protein_coding	-634
ENSG0000(	LYPLA2	1	protein_coding	-405
ENSG0000(	NME1-NME2	1	protein_coding	-173
ENSG0000(	BTBD7	1	protein_coding	888
ENSG0000(	APBA3	1	protein_coding	-151
ENSG0000(	MKS1	1	protein_coding	124
ENSG0000(	ABHD5	1	protein_coding	511
ENSG0000(	PTBP1	1	protein_coding	-119
ENSG0000(	LARS2	1	protein_coding	-63
ENSG0000(	LARS2	1	protein_coding	308
ENSG0000(	PIK3C2A	1	protein_coding	-406
ENSG0000(	PIK3C2A	1	protein_coding	-894
ENSG0000(	QPCTL	1	protein_coding	124
ENSG0000(	QPCTL	1	protein_coding	536
ENSG0000(	PPP5C	1	protein_coding	-231
ENSG0000(	ELOVL5	1	protein_coding	81
ENSG0000(	PSMC4	1	protein_coding	-209

ENSG0000(	PSMC4	1	protein_coding	-675
ENSG0000(	RWDD2A	1	protein_coding	-523
ENSG0000(	CLK1	1	protein_coding	2
ENSG0000(	POLR3B	1	protein_coding	30
ENSG0000(	POLA2	1	protein_coding	224
ENSG0000(	MDH1	1	protein_coding	208
ENSG0000(	SLC30A9	1	protein_coding	114
ENSG0000(	WAS	1	protein_coding	36
ENSG0000(	MATR3	1	protein_coding	708
ENSG0000(	XYLT2	1	protein_coding	-358
ENSG0000(	GLT8D1	1	protein_coding	-127
ENSG0000(	GLT8D1	1	protein_coding	75
ENSG0000(	RALBP1	1	protein_coding	209
ENSG0000(	NCDN	1	protein_coding	24
ENSG0000(	ALAS1	1	protein_coding	-154
ENSG0000(	GLRX2	1	protein_coding	396
ENSG0000(	DEF6	1	protein_coding	-97
ENSG0000(	DEF6	1	protein_coding	964
ENSG0000(	UBR2	1	protein_coding	35
ENSG0000(	CCDC28A	1	protein_coding	131
ENSG0000(	HSF2	1	protein_coding	362
ENSG0000(	PHF20	1	protein_coding	-256
ENSG0000(	NR1H3	1	protein_coding	770
ENSG0000(	KPNA6	1	protein_coding	77
ENSG0000(	RTEL1-TNFRSF6B	1	protein_coding	-682
ENSG0000(	RNASET2	1	protein_coding	-429
ENSG0000(	BRD9	1	protein_coding	465
ENSG0000(	SNX1	1	protein_coding	-324
ENSG0000(	SNX1	1	protein_coding	-821
ENSG0000(	BCLAF1	1	protein_coding	244
ENSG0000(	SLC39A9	1	protein_coding	553
ENSG0000(	NUP160	1	protein_coding	-319
ENSG0000(	NUP160	1	protein_coding	390
ENSG0000(	CENPQ	1	protein_coding	17
ENSG0000(	SARS	1	protein_coding	182
ENSG0000(	RANBP3	1	protein_coding	-269
ENSG0000(	TSSC1	1	protein_coding	-83
ENSG0000(	TSSC1	1	protein_coding	-842
ENSG0000(	CHPF2	1	protein_coding	-480
ENSG0000(	CHPF2	1	protein_coding	761
ENSG0000(	PEX3	1	protein_coding	-52
ENSG0000(	GABARAPL2	1	protein_coding	-565
ENSG0000(	VCL	1	protein_coding	-311
ENSG0000(	DEPDC1B	1	protein_coding	411
ENSG0000(	NSMAF	1	protein_coding	-383
ENSG0000(	RFC1	1	protein_coding	-91
ENSG0000(	USP2	1	protein_coding	-259
ENSG0000(	FBXO42	1	protein_coding	-383
ENSG0000(	FBXO42	1	protein_coding	-912
ENSG0000(	MFAP3	1	protein_coding	-262
ENSG0000(	EDC4	1	protein_coding	-957
ENSG0000(	SKIV2L2	1	protein_coding	373
ENSG0000(	PNKP	1	protein_coding	878
ENSG0000(	PQLC2	1	protein_coding	382

ENSG0000(	PQLC2	1	protein_coding	755
ENSG0000(	RTN4R	1	protein_coding	69
ENSG0000(	PSMA4	1	protein_coding	-281
ENSG0000(	THAP3	1	protein_coding	-59
ENSG0000(	FAM65C	1	protein_coding	758
ENSG0000(	TDP1	1	protein_coding	-341
ENSG0000(	LCP2	1	protein_coding	960
ENSG0000(	CUL7	1	protein_coding	316
ENSG0000(	CUL7	1	protein_coding	714
ENSG0000(	HSPA5	1	protein_coding	-540
ENSG0000(	HSPA5	1	protein_coding	55
ENSG0000(	WDR37	1	protein_coding	4
ENSG0000(	WDR37	1	protein_coding	469
ENSG0000(	TPR	1	protein_coding	116
ENSG0000(	GOPC	1	protein_coding	-199
ENSG0000(	TSPAN17	1	protein_coding	-397
ENSG0000(	NOP16	1	protein_coding	313
ENSG0000(	RRM2B	1	protein_coding	579
ENSG0000(	SNX29	1	protein_coding	-150
ENSG0000(	MRPS10	1	protein_coding	121
ENSG0000(	VPS13D	1	protein_coding	-291
ENSG0000(	ADAMTS6	1	protein_coding	-53
ENSG0000(	RFC2	1	protein_coding	-171
ENSG0000(	RFC2	1	protein_coding	383
ENSG0000(	NFE2L3	1	protein_coding	-353
ENSG0000(	MCUR1	1	protein_coding	369
ENSG0000(	LETMD1	1	protein_coding	257
ENSG0000(	LAMC3	1	protein_coding	-309
ENSG0000(	FAM160A2	1	protein_coding	-629
ENSG0000(	RAD51	1	protein_coding	-12
ENSG0000(	RAD51	1	protein_coding	-771
ENSG0000(	PIK3CB	1	protein_coding	-395
ENSG0000(	PRSS8	1	protein_coding	-156
ENSG0000(	AKR7A2	1	protein_coding	-561
ENSG0000(	AKR7A2	1	protein_coding	-934
ENSG0000(	MRTO4	1	protein_coding	131
ENSG0000(	THRAP3	1	protein_coding	-390
ENSG0000(	THRAP3	1	protein_coding	658
ENSG0000(	PHPT1	1	protein_coding	-558
ENSG0000(	SDCCAG8	1	protein_coding	-675
ENSG0000(	SDCCAG8	1	protein_coding	146
ENSG0000(	KIF1B	1	protein_coding	-389
ENSG0000(	FAM168A	1	protein_coding	-540
ENSG0000(	SZRD1	1	protein_coding	263
ENSG0000(	SZRD1	1	protein_coding	792
ENSG0000(	KCNH2	1	protein_coding	919
ENSG0000(	CUL1	1	protein_coding	-302
ENSG0000(	FAM114A2	1	protein_coding	293
ENSG0000(	USP36	1	protein_coding	398
ENSG0000(	PUM2	1	protein_coding	777
ENSG0000(	ATG5	1	protein_coding	-60
ENSG0000(	PITHD1	1	protein_coding	-370
ENSG0000(	ATP11B	1	protein_coding	-465
ENSG0000(	YIPF1	1	protein_coding	-547

ENSG0000(	NDC1	1	protein_coding	314
ENSG0000(	PARP12	1	protein_coding	679
ENSG0000(	DNAJC25	1	protein_coding	-199
ENSG0000(	PSD	1	protein_coding	-835
ENSG0000(	PSD	1	protein_coding	554
ENSG0000(	CCAR1	1	protein_coding	10
ENSG0000(	PIGV	1	protein_coding	-318
ENSG0000(	PTPRU	1	protein_coding	465
ENSG0000(	MPC1	1	protein_coding	174
ENSG0000(	MPC1	1	protein_coding	810
ENSG0000(	ACAA1	1	protein_coding	-243
ENSG0000(	BCAT1	1	protein_coding	193
ENSG0000(	NCKAP1	1	protein_coding	-143
ENSG0000(	CS	1	protein_coding	-507
ENSG0000(	CS	1	protein_coding	-914
ENSG0000(	CS	1	protein_coding	458
ENSG0000(	LTK	1	protein_coding	-653
ENSG0000(	LTK	1	protein_coding	17
ENSG0000(	VMP1	1	protein_coding	208
ENSG0000(	GLTSCR1	1	protein_coding	20
ENSG0000(	MED29	1	protein_coding	-129
ENSG0000(	HAGH	1	protein_coding	9
ENSG0000(	TM7SF3	1	protein_coding	-326
ENSG0000(	TSPAN32	1	protein_coding	-185
ENSG0000(	TSPAN32	1	protein_coding	661
ENSG0000(	TNPO3	1	protein_coding	334
ENSG0000(	BORCS8-MEF2B	1	protein_coding	-226
ENSG0000(	RFXANK	1	protein_coding	156
ENSG0000(	SUGP2	1	protein_coding	-93
ENSG0000(	ABCA7	1	protein_coding	228
ENSG0000(	DDX20	1	protein_coding	574
ENSG0000(	BTBD1	1	protein_coding	-26
ENSG0000(	BTBD1	1	protein_coding	-280
ENSG0000(	SBNO2	1	protein_coding	-164
ENSG0000(	TAF11	1	protein_coding	-98
ENSG0000(	UHRF1BP1	1	protein_coding	-90
ENSG0000(	UHRF1BP1	1	protein_coding	-503
ENSG0000(	GNAI3	1	protein_coding	-450
ENSG0000(	WDR18	1	protein_coding	-58
ENSG0000(	TBC1D22B	1	protein_coding	176
ENSG0000(	SPEN	1	protein_coding	-557
ENSG0000(	ZC3H15	1	protein_coding	-73
ENSG0000(	TMEM206	1	protein_coding	-139
ENSG0000(	TMEM206	1	protein_coding	-558
ENSG0000(	CYB5R4	1	protein_coding	-13
ENSG0000(	PRKCQ	1	protein_coding	-613
ENSG0000(	ASB1	1	protein_coding	-411
ENSG0000(	YBX1	1	protein_coding	-893
ENSG0000(	PDE4A	1	protein_coding	-25
ENSG0000(	PPP2R5A	1	protein_coding	223
ENSG0000(	PPP2R5A	1	protein_coding	978
ENSG0000(	ELAVL1	1	protein_coding	-480
ENSG0000(	KDM4A	1	protein_coding	543
ENSG0000(	NFYC	1	protein_coding	-134

ENSG0000(	NFYC	1	protein_coding	671
ENSG0000(	ASPM	1	protein_coding	-225
ENSG0000(	ASPM	1	protein_coding	445
ENSG0000(	ELOVL1	1	protein_coding	-479
ENSG0000(	ELOVL1	1	protein_coding	756
ENSG0000(	SPI1	1	protein_coding	-37
ENSG0000(	TRMT11	1	protein_coding	72
ENSG0000(	MSANTD3	1	protein_coding	-508
ENSG0000(	ZFAT	1	protein_coding	-637
ENSG0000(	MTFR1	1	protein_coding	-262
ENSG0000(	MTFR1	1	protein_coding	94
ENSG0000(	FECH	1	protein_coding	-373
ENSG0000(	IDI1	1	protein_coding	-371
ENSG0000(	IDI1	1	protein_coding	-836
ENSG0000(	SP100	1	protein_coding	35
ENSG0000(	TRAM1	1	protein_coding	-202
ENSG0000(	PHKA1	1	protein_coding	555
ENSG0000(	TNFRSF1A	1	protein_coding	162
ENSG0000(	STOML1	1	protein_coding	-843
ENSG0000(	DHX29	1	protein_coding	-410
ENSG0000(	METTL22	1	protein_coding	309
ENSG0000(	TP53BP1	1	protein_coding	-559
ENSG0000(	RRP15	1	protein_coding	-5
ENSG0000(	IDH3G	1	protein_coding	343
ENSG0000(	ROGDI	1	protein_coding	891
ENSG0000(	CBFB	1	protein_coding	-372
ENSG0000(	PLEKHH3	1	protein_coding	582
ENSG0000(	INPP5A	1	protein_coding	-698
ENSG0000(	FTSJ1	1	protein_coding	12
ENSG0000(	PRR11	1	protein_coding	-538
ENSG0000(	POLR1A	1	protein_coding	-387
ENSG0000(	LAPTM4A	1	protein_coding	268
ENSG0000(	TTC7A	1	protein_coding	340
ENSG0000(	KIF2A	1	protein_coding	-328
ENSG0000(	PSME4	1	protein_coding	924
ENSG0000(	IFT80	1	protein_coding	235
ENSG0000(	SIRT2	1	protein_coding	140
ENSG0000(	SIRT2	1	protein_coding	598
ENSG0000(	PITX1	1	protein_coding	-189
ENSG0000(	PITX1	1	protein_coding	-589
ENSG0000(	NUP133	1	protein_coding	-222
ENSG0000(	NUP133	1	protein_coding	-857
ENSG0000(	BCL3	1	protein_coding	-777
ENSG0000(	HES2	1	protein_coding	-594
ENSG0000(	IKBKAP	1	protein_coding	-580
ENSG0000(	NUCB2	1	protein_coding	237
ENSG0000(	NUCB2	1	protein_coding	725
ENSG0000(	CLTCL1	1	protein_coding	-753
ENSG0000(	DGCR2	1	protein_coding	-435
ENSG0000(	MNT	1	protein_coding	-479
ENSG0000(	MNT	1	protein_coding	-758
ENSG0000(	ST6GALNAC1	1	protein_coding	-119
ENSG0000(	ST6GALNAC1	1	protein_coding	697
ENSG0000(	GBA2	1	protein_coding	-772

ENSG0000(	PABPC1	1	protein_coding	-384
ENSG0000(	EIF2B3	1	protein_coding	825
ENSG0000(	CDC42	1	protein_coding	129
ENSG0000(	RPL31	1	protein_coding	-67
ENSG0000(	RPL31	1	protein_coding	445
ENSG0000(	WDR1	1	protein_coding	-369
ENSG0000(	SNX13	1	protein_coding	512
ENSG0000(	ING3	1	protein_coding	586
ENSG0000(	TRIP13	1	protein_coding	-283
ENSG0000(	TRIP13	1	protein_coding	-866
ENSG0000(	HLTF	1	protein_coding	-85
ENSG0000(	CYBRD1	1	protein_coding	-16
ENSG0000(	PDCD2	1	protein_coding	591
ENSG0000(	RDH11	1	protein_coding	574
ENSG0000(	PRKACA	1	protein_coding	-549
ENSG0000(	TFRC	1	protein_coding	-292
ENSG0000(	TFRC	1	protein_coding	142
ENSG0000(	SREBF1	1	protein_coding	335
ENSG0000(	SREBF1	1	protein_coding	785
ENSG0000(	AFF4	1	protein_coding	-437
ENSG0000(	SMC1A	1	protein_coding	-317
ENSG0000(	HMMR	1	protein_coding	-41
ENSG0000(	HMMR	1	protein_coding	724
ENSG0000(	P4HA2	1	protein_coding	-676
ENSG0000(	NFATC3	1	protein_coding	-120
ENSG0000(	FBXW11	1	protein_coding	-73
ENSG0000(	TMEM38A	1	protein_coding	-452
ENSG0000(	TMEM38A	1	protein_coding	-903
ENSG0000(	PDE8A	1	protein_coding	-108
ENSG0000(	PDE8A	1	protein_coding	962
ENSG0000(	NLE1	1	protein_coding	-82
ENSG0000(	MAP3K13	1	protein_coding	437
ENSG0000(	MRPS34	1	protein_coding	-357
ENSG0000(	MRPS34	1	protein_coding	850
ENSG0000(	CLNS1A	1	protein_coding	-105
ENSG0000(	CLNS1A	1	protein_coding	-597
ENSG0000(	CDHR2	1	protein_coding	-711
ENSG0000(	CDHR2	1	protein_coding	6
ENSG0000(	TSG101	1	protein_coding	343
ENSG0000(	ATP2A3	1	protein_coding	-802
ENSG0000(	LMAN1	1	protein_coding	141
ENSG0000(	HACD3	1	protein_coding	96
ENSG0000(	ENO1	1	protein_coding	613
ENSG0000(	MYDGF	1	protein_coding	81
ENSG0000(	ANO8	1	protein_coding	-28
ENSG0000(	ANO8	1	protein_coding	572
ENSG0000(	EIF4G3	1	protein_coding	-353
ENSG0000(	TMEM131	1	protein_coding	-483
ENSG0000(	ACTB	1	protein_coding	-6
ENSG0000(	MOCOS	1	protein_coding	613
ENSG0000(	PLD1	1	protein_coding	845
ENSG0000(	WDR62	1	protein_coding	-272
ENSG0000(	WDR62	1	protein_coding	-773
ENSG0000(	BCAP29	1	protein_coding	726

ENSG0000(	SART3	1	protein_coding	37
ENSG0000(	EXOSC7	1	protein_coding	334
ENSG0000(	MKRN2	1	protein_coding	3
ENSG0000(	MCM6	1	protein_coding	924
ENSG0000(	REXO2	1	protein_coding	627
ENSG0000(	RBM7	1	protein_coding	377
ENSG0000(	BAZ2A	1	protein_coding	217
ENSG0000(	PTPN23	1	protein_coding	-155
ENSG0000(	MLH1	1	protein_coding	-724
ENSG0000(	MLH1	1	protein_coding	244
ENSG0000(	KLHL20	1	protein_coding	-138
ENSG0000(	KLHL20	1	protein_coding	589
ENSG0000(	SPAG5	1	protein_coding	102
ENSG0000(	TRAF4	1	protein_coding	-704
ENSG0000(	MCAM	1	protein_coding	-136
ENSG0000(	RAP1GAP	1	protein_coding	-266
ENSG0000(	RAP1GAP	1	protein_coding	-879
ENSG0000(	MAP2K7	1	protein_coding	-237
ENSG0000(	UBE2T	1	protein_coding	192
ENSG0000(	PPP1R12B	1	protein_coding	-85
ENSG0000(	PPP1R12B	1	protein_coding	602
ENSG0000(	DNAJC10	1	protein_coding	617
ENSG0000(	SNRPA	1	protein_coding	-278
ENSG0000(	DYNC1I2	1	protein_coding	49
ENSG0000(	FAM76B	1	protein_coding	639
ENSG0000(	ACER3	1	protein_coding	-638
ENSG0000(	ACER3	1	protein_coding	770
ENSG0000(	SYNJ2	1	protein_coding	-364
ENSG0000(	SYNJ2	1	protein_coding	-704
ENSG0000(	ZCWPW1	1	protein_coding	-882
ENSG0000(	ZCWPW1	1	protein_coding	701
ENSG0000(	NRDC	1	protein_coding	-423
ENSG0000(	NRDC	1	protein_coding	351
ENSG0000(	NRDC	1	protein_coding	798
ENSG0000(	VDAC3	1	protein_coding	-57
ENSG0000(	PCM1	1	protein_coding	-95
ENSG0000(	SDF4	1	protein_coding	505
ENSG0000(	UBE2D4	1	protein_coding	209
ENSG0000(	THOC1	1	protein_coding	-411
ENSG0000(	THOC1	1	protein_coding	559
ENSG0000(	FKBP7	1	protein_coding	-317
ENSG0000(	OSBPL6	1	protein_coding	-421
ENSG0000(	MKNK1	1	protein_coding	-167
ENSG0000(	SAR1A	1	protein_coding	600
ENSG0000(	DUSP13	1	protein_coding	440
ENSG0000(	CIC	1	protein_coding	-272
ENSG0000(	KIF22	1	protein_coding	-171
ENSG0000(	KIF22	1	protein_coding	470
ENSG0000(	DDX1	1	protein_coding	437
ENSG0000(	KEAP1	1	protein_coding	864
ENSG0000(	SLC35C2	1	protein_coding	104
ENSG0000(	RIF1	1	protein_coding	-139
ENSG0000(	RIF1	1	protein_coding	240
ENSG0000(	SESN1	1	protein_coding	-662

ENSG0000(	CNOT4	1	protein_coding	-602
ENSG0000(	PSEN1	1	protein_coding	803
ENSG0000(	MOK	1	protein_coding	-37
ENSG0000(	RBL1	1	protein_coding	-22
ENSG0000(	NDC80	1	protein_coding	59
ENSG0000(	AP4E1	1	protein_coding	-80
ENSG0000(	RSBN1	1	protein_coding	152
ENSG0000(	EXD2	1	protein_coding	-103
ENSG0000(	KIAA0141	1	protein_coding	-351
ENSG0000(	PHLPP1	1	protein_coding	-425
ENSG0000(	SMARCD3	1	protein_coding	537
ENSG0000(	ME2	1	protein_coding	-437
ENSG0000(	CCNT2	1	protein_coding	-304
ENSG0000(	GEMIN5	1	protein_coding	774
ENSG0000(	NFE2L1	1	protein_coding	-555
ENSG0000(	XPO1	1	protein_coding	-605
ENSG0000(	KAT6A	1	protein_coding	-423
ENSG0000(	PLOD1	1	protein_coding	2
ENSG0000(	NUFIP1	1	protein_coding	-61
ENSG0000(	OXCT1	1	protein_coding	-85
ENSG0000(	RPS5	1	protein_coding	-451
ENSG0000(	RPS5	1	protein_coding	412
ENSG0000(	RPS5	1	protein_coding	840
ENSG0000(	YTHDC1	1	protein_coding	-190
ENSG0000(	ZMPSTE24	1	protein_coding	-253
ENSG0000(	STARD7	1	protein_coding	384
ENSG0000(	SSH1	1	protein_coding	992
ENSG0000(	EIF3I	1	protein_coding	-437
ENSG0000(	EIF3I	1	protein_coding	509
ENSG0000(	TXLNA	1	protein_coding	-48
ENSG0000(	TXLNA	1	protein_coding	-721
ENSG0000(	TXLNA	1	protein_coding	829
ENSG0000(	HADHA	1	protein_coding	-711
ENSG0000(	HADHA	1	protein_coding	497
ENSG0000(	CD59	1	protein_coding	-212
ENSG0000(	CD82	1	protein_coding	-31
ENSG0000(	AK6	1	protein_coding	236
ENSG0000(	WDR47	1	protein_coding	42
ENSG0000(	MAP3K4	1	protein_coding	912
ENSG0000(	RRN3	1	protein_coding	-65
ENSG0000(	CTTN	1	protein_coding	-256
ENSG0000(	CTTN	1	protein_coding	-589
ENSG0000(	TTC39A	1	protein_coding	-988
ENSG0000(	ORC1	1	protein_coding	469
ENSG0000(	MGST2	1	protein_coding	-134
ENSG0000(	MGST2	1	protein_coding	317
ENSG0000(	RAD54L	1	protein_coding	-544
ENSG0000(	NFX1	1	protein_coding	-39
ENSG0000(	AQP6	1	protein_coding	29
ENSG0000(	HBQ1	1	protein_coding	664
ENSG0000(	ITPKC	1	protein_coding	-627
ENSG0000(	TXLNG	1	protein_coding	204
ENSG0000(	MTMR2	1	protein_coding	911
ENSG0000(	PPP1R15A	1	protein_coding	82

ENSG0000(	HSD17B14	1	protein_coding	789
ENSG0000(	TRIP6	1	protein_coding	-84
ENSG0000(	ACHE	1	protein_coding	831
ENSG0000(	FTL	1	protein_coding	814
ENSG0000(	PSMC5	1	protein_coding	117
ENSG0000(	UIMC1	1	protein_coding	-777
ENSG0000(	LPCAT2	1	protein_coding	-140
ENSG0000(	NOP14	1	protein_coding	306
ENSG0000(	NOP14	1	protein_coding	889
ENSG0000(	ADD1	1	protein_coding	760
ENSG0000(	GMCL1	1	protein_coding	-194
ENSG0000(	KLHL42	1	protein_coding	672
ENSG0000(	AURKA	1	protein_coding	-254
ENSG0000(	ALG6	1	protein_coding	-486
ENSG0000(	ALG6	1	protein_coding	556
ENSG0000(	CNOT3	1	protein_coding	-821
ENSG0000(	GP6	1	protein_coding	-94
ENSG0000(	PTPN4	1	protein_coding	378
ENSG0000(	KHSRP	1	protein_coding	-866
ENSG0000(	DNMT3B	1	protein_coding	713
ENSG0000(	KIF9	1	protein_coding	872
ENSG0000(	ZNF343	1	protein_coding	126
ENSG0000(	CPXM1	1	protein_coding	412
ENSG0000(	MAVS	1	protein_coding	868
ENSG0000(	DYNLL1	1	protein_coding	362
ENSG0000(	SNX5	1	protein_coding	88
ENSG0000(	SNX5	1	protein_coding	255
ENSG0000(	RPL6	1	protein_coding	-678
ENSG0000(	MAPKAPK5	1	protein_coding	-130
ENSG0000(	ESF1	1	protein_coding	-18
ENSG0000(	KDM2B	1	protein_coding	-624
ENSG0000(	CFAP61	1	protein_coding	-64
ENSG0000(	GCN1	1	protein_coding	-470
ENSG0000(	ERP29	1	protein_coding	-393
ENSG0000(	FXYD5	1	protein_coding	-867
ENSG0000(	GMIP	1	protein_coding	-457
ENSG0000(	BIRC5	1	protein_coding	54
ENSG0000(	NECAP1	1	protein_coding	-56
ENSG0000(	BLVRB	1	protein_coding	-493
ENSG0000(	BLVRB	1	protein_coding	674
ENSG0000(	PAPOLA	1	protein_coding	876
ENSG0000(	PCBP4	1	protein_coding	-72
ENSG0000(	MRPS33	1	protein_coding	628
ENSG0000(	NUDC	1	protein_coding	358
ENSG0000(	ICAM1	1	protein_coding	-365
ENSG0000(	ICAM1	1	protein_coding	150
ENSG0000(	MUL1	1	protein_coding	10
ENSG0000(	MUL1	1	protein_coding	791
ENSG0000(	PDCD7	1	protein_coding	-185
ENSG0000(	FLT3LG	1	protein_coding	-170
ENSG0000(	FLT3LG	1	protein_coding	568
ENSG0000(	USP48	1	protein_coding	-15
ENSG0000(	USP48	1	protein_coding	806
ENSG0000(	OSBPL8	1	protein_coding	-345

ENSG0000(	DLD	1	protein_coding	109
ENSG0000(	DLD	1	protein_coding	725
ENSG0000(	WDR7	1	protein_coding	-228
ENSG0000(	TXNL1	1	protein_coding	486
ENSG0000(	AC013461.1	1	protein_coding	-158
ENSG0000(	CDV3	1	protein_coding	-671
ENSG0000(	ALKBH5	1	protein_coding	266
ENSG0000(	PPP2R3C	1	protein_coding	307
ENSG0000(	HAUS4	1	protein_coding	-157
ENSG0000(	SCFD1	1	protein_coding	-149
ENSG0000(	HECTD1	1	protein_coding	-581
ENSG0000(	TYRO3	1	protein_coding	962
ENSG0000(	SNAP23	1	protein_coding	-20
ENSG0000(	EZR	1	protein_coding	-505
ENSG0000(	CLSPN	1	protein_coding	3
ENSG0000(	COMT	1	protein_coding	400
ENSG0000(	HDAC6	1	protein_coding	-378
ENSG0000(	AAAS	1	protein_coding	374
ENSG0000(	CBX5	1	protein_coding	-381
ENSG0000(	CBX5	1	protein_coding	-976
ENSG0000(	DHPS	1	protein_coding	-28
ENSG0000(	PSMD5	1	protein_coding	250
ENSG0000(	SH2D3C	1	protein_coding	-396
ENSG0000(	TBC1D2	1	protein_coding	-345
ENSG0000(	WHRN	1	protein_coding	919
ENSG0000(	CYP26A1	1	protein_coding	-170
ENSG0000(	WAC	1	protein_coding	427
ENSG0000(	CREM	1	protein_coding	264
ENSG0000(	NUBP2	1	protein_coding	-122
ENSG0000(	HIVEP1	1	protein_coding	-142
ENSG0000(	FKBP5	1	protein_coding	76
ENSG0000(	MRPS18A	1	protein_coding	-118
ENSG0000(	TMEM14A	1	protein_coding	-108
ENSG0000(	EFHC1	1	protein_coding	-239
ENSG0000(	HSP90AB1	1	protein_coding	-357
ENSG0000(	ZNF184	1	protein_coding	334
ENSG0000(	SIRT1	1	protein_coding	-686
ENSG0000(	HNRNPH3	1	protein_coding	588
ENSG0000(	IFT74	1	protein_coding	328
ENSG0000(	ACOT7	1	protein_coding	340
ENSG0000(	CDC7	1	protein_coding	-429
ENSG0000(	TMED1	1	protein_coding	-354
ENSG0000(	RAB18	1	protein_coding	-170
ENSG0000(	RAB18	1	protein_coding	147
ENSG0000(	PRTFDC1	1	protein_coding	771
ENSG0000(	MZF1	1	protein_coding	828
ENSG0000(	MYO9B	1	protein_coding	-831
ENSG0000(	CATSPERG	1	protein_coding	-83
ENSG0000(	PSMD8	1	protein_coding	-314
ENSG0000(	FBXL19	1	protein_coding	-210
ENSG0000(	SETD1A	1	protein_coding	-20
ENSG0000(	SETD1A	1	protein_coding	497
ENSG0000(	BCL7C	1	protein_coding	-380
ENSG0000(	BCL7C	1	protein_coding	-950

ENSG0000(	BCL7C	1	protein_coding	337
ENSG0000(	BCL7C	1	protein_coding	662
ENSG0000(	CIRBP	1	protein_coding	31
ENSG0000(	ATP5D	1	protein_coding	-78
ENSG0000(	CBARP	1	protein_coding	-689
ENSG0000(	NDUFB7	1	protein_coding	7
ENSG0000(	TIMM13	1	protein_coding	791
ENSG0000(	POLRMT	1	protein_coding	61
ENSG0000(	IZUMO4	1	protein_coding	-311
ENSG0000(	MKNK2	1	protein_coding	-363
ENSG0000(	MKNK2	1	protein_coding	15
ENSG0000(	ARVCF	1	protein_coding	-658
ENSG0000(	TRMT2A	1	protein_coding	373
ENSG0000(	RANBP1	1	protein_coding	-947
ENSG0000(	ZDHHC8	1	protein_coding	-155
ENSG0000(	KLHL22	1	protein_coding	-565
ENSG0000(	KLHL22	1	protein_coding	780
ENSG0000(	MED15	1	protein_coding	-809
ENSG0000(	MED15	1	protein_coding	536
ENSG0000(	SNAP29	1	protein_coding	-576
ENSG0000(	CRKL	1	protein_coding	220
ENSG0000(	LZTR1	1	protein_coding	774
ENSG0000(	P2RX6	1	protein_coding	303
ENSG0000(	PPIL2	1	protein_coding	-743
ENSG0000(	PPIL2	1	protein_coding	131
ENSG0000(	YPEL1	1	protein_coding	879
ENSG0000(	MAPK1	1	protein_coding	-329
ENSG0000(	MAPK1	1	protein_coding	784
ENSG0000(	PPM1F	1	protein_coding	-262
ENSG0000(	PPM1F	1	protein_coding	789
ENSG0000(	TOP3B	1	protein_coding	359
ENSG0000(	DGCR14	1	protein_coding	219
ENSG0000(	HIRA	1	protein_coding	-3
ENSG0000(	HPS4	1	protein_coding	-435
ENSG0000(	SRRD	1	protein_coding	396
ENSG0000(	CRYBB1	1	protein_coding	-155
ENSG0000(	MICALL1	1	protein_coding	-48
ENSG0000(	POLR2F	1	protein_coding	701
ENSG0000(	DEPDC5	1	protein_coding	-629
ENSG0000(	DDX17	1	protein_coding	813
ENSG0000(	DMC1	1	protein_coding	-509
ENSG0000(	HSCB	1	protein_coding	294
ENSG0000(	CYB5R3	1	protein_coding	919
ENSG0000(	TTLL1	1	protein_coding	-792
ENSG0000(	RPL3	1	protein_coding	285
ENSG0000(	RBFOX2	1	protein_coding	462
ENSG0000(	APOL1	1	protein_coding	-129
ENSG0000(	FOXRED2	1	protein_coding	-322
ENSG0000(	EIF3D	1	protein_coding	156
ENSG0000(	TNRC6B	1	protein_coding	-523
ENSG0000(	SLC25A17	1	protein_coding	501
ENSG0000(	ST13	1	protein_coding	73
ENSG0000(	L3MBTL2	1	protein_coding	-58
ENSG0000(	L3MBTL2	1	protein_coding	155

ENSG0000(	RANGAP1	1	protein_coding	-166
ENSG0000(	DESI1	1	protein_coding	444
ENSG0000(	CERK	1	protein_coding	853
ENSG0000(	RBM23	1	protein_coding	241
ENSG0000(	POLE2	1	protein_coding	-407
ENSG0000(	NIN	1	protein_coding	-689
ENSG0000(	ERH	1	protein_coding	60
ENSG0000(	TELO2	1	protein_coding	-127
ENSG0000(	PSMB5	1	protein_coding	143
ENSG0000(	ACIN1	1	protein_coding	223
ENSG0000(	PABPN1	1	protein_coding	-484
ENSG0000(	KIAA0391	1	protein_coding	365
ENSG0000(	BRMS1L	1	protein_coding	-262
ENSG0000(	PCIF1	1	protein_coding	147
ENSG0000(	GSS	1	protein_coding	300
ENSG0000(	TRPC4AP	1	protein_coding	-325
ENSG0000(	UQCC1	1	protein_coding	153
ENSG0000(	ZMYND8	1	protein_coding	-701
ENSG0000(	ZMYND8	1	protein_coding	108
ENSG0000(	PABPC1L	1	protein_coding	864
ENSG0000(	STK4	1	protein_coding	-143
ENSG0000(	CSTF1	1	protein_coding	221
ENSG0000(	TPD52L2	1	protein_coding	-827
ENSG0000(	TPD52L2	1	protein_coding	26
ENSG0000(	CTSZ	1	protein_coding	-440
ENSG0000(	PRPF6	1	protein_coding	-108
ENSG0000(	PRPF6	1	protein_coding	-616
ENSG0000(	DIDO1	1	protein_coding	77
ENSG0000(	DIDO1	1	protein_coding	377
ENSG0000(	GID8	1	protein_coding	-243
ENSG0000(	GID8	1	protein_coding	-543
ENSG0000(	GMEB2	1	protein_coding	-396
ENSG0000(	GMEB2	1	protein_coding	912
ENSG0000(	C20orf27	1	protein_coding	194
ENSG0000(	NDUFAF5	1	protein_coding	-45
ENSG0000(	TRIB3	1	protein_coding	549
ENSG0000(	CSNK2A1	1	protein_coding	-429
ENSG0000(	CDS2	1	protein_coding	-356
ENSG0000(	SNPH	1	protein_coding	-658
ENSG0000(	TM9SF4	1	protein_coding	78
ENSG0000(	KIF3B	1	protein_coding	-345
ENSG0000(	MANBAL	1	protein_coding	-32
ENSG0000(	CDK5RAP1	1	protein_coding	41
ENSG0000(	CDK5RAP1	1	protein_coding	461
ENSG0000(	TTI1	1	protein_coding	-46
ENSG0000(	TTI1	1	protein_coding	-475
ENSG0000(	TTI1	1	protein_coding	358
ENSG0000(	E2F1	1	protein_coding	823
ENSG0000(	RPRD1B	1	protein_coding	-31
ENSG0000(	RPRD1B	1	protein_coding	-435
ENSG0000(	RPRD1B	1	protein_coding	398
ENSG0000(	PXMP4	1	protein_coding	-39
ENSG0000(	ACTR5	1	protein_coding	470
ENSG0000(	DHX35	1	protein_coding	-310

ENSG0000(	METTL4	1	protein_coding	-60
ENSG0000(	LPIN2	1	protein_coding	-318
ENSG0000(	SMCHD1	1	protein_coding	-365
ENSG0000(	CEP192	1	protein_coding	458
ENSG0000(	RNMT	1	protein_coding	8
ENSG0000(	RNF125	1	protein_coding	782
ENSG0000(	ANKRD12	1	protein_coding	-410
ENSG0000(	ANKRD12	1	protein_coding	282
ENSG0000(	TBL1X	1	protein_coding	-54
ENSG0000(	UBL4A	1	protein_coding	756
ENSG0000(	TIMP1	1	protein_coding	-501
ENSG0000(	PIN4	1	protein_coding	614
ENSG0000(	GLA	1	protein_coding	-231
ENSG0000(	CAB39L	1	protein_coding	73
ENSG0000(	ACP5	1	protein_coding	607
ENSG0000(	ACP5	1	protein_coding	855
ENSG0000(	MGRN1	1	protein_coding	-755
ENSG0000(	TRADD	1	protein_coding	117
ENSG0000(	HSF4	1	protein_coding	300
ENSG0000(	TSNAXIP1	1	protein_coding	458
ENSG0000(	NFAT5	1	protein_coding	-762
ENSG0000(	ZNF423	1	protein_coding	106
ENSG0000(	ACD	1	protein_coding	85
ENSG0000(	ACD	1	protein_coding	487
ENSG0000(	PARD6A	1	protein_coding	-215
ENSG0000(	PARD6A	1	protein_coding	-617
ENSG0000(	NME3	1	protein_coding	-575
ENSG0000(	TANGO6	1	protein_coding	-212
ENSG0000(	COG4	1	protein_coding	-648
ENSG0000(	PLA2G15	1	protein_coding	-174
ENSG0000(	PLA2G15	1	protein_coding	-405
ENSG0000(	PLA2G15	1	protein_coding	485
ENSG0000(	WDR59	1	protein_coding	-136
ENSG0000(	MON1B	1	protein_coding	372
ENSG0000(	CRISPLD2	1	protein_coding	479
ENSG0000(	ZNF500	1	protein_coding	-938
ENSG0000(	ABCC1	1	protein_coding	-327
ENSG0000(	NARFL	1	protein_coding	302
ENSG0000(	MTHFSD	1	protein_coding	530
ENSG0000(	FAM173A	1	protein_coding	72
ENSG0000(	RHBDL1	1	protein_coding	-349
ENSG0000(	RHBDL1	1	protein_coding	-641
ENSG0000(	ZNF174	1	protein_coding	-203
ENSG0000(	UBFD1	1	protein_coding	71
ENSG0000(	EARS2	1	protein_coding	299
ENSG0000(	TCEB2	1	protein_coding	708
ENSG0000(	CPPED1	1	protein_coding	446
ENSG0000(	USP31	1	protein_coding	-454
ENSG0000(	HMOX2	1	protein_coding	-660
ENSG0000(	DNAJA3	1	protein_coding	-191
ENSG0000(	DNAJA3	1	protein_coding	887
ENSG0000(	BFAR	1	protein_coding	-369
ENSG0000(	QPRT	1	protein_coding	816
ENSG0000(	STX4	1	protein_coding	160

ENSG0000(	BCKDK	1	protein_coding	-182
ENSG0000(	HERC1	1	protein_coding	-325
ENSG0000(	HERC1	1	protein_coding	-797
ENSG0000(	TRIP4	1	protein_coding	2
ENSG0000(	HOMER2	1	protein_coding	222
ENSG0000(	EHD4	1	protein_coding	-376
ENSG0000(	ZNF106	1	protein_coding	-89
ENSG0000(	CEP152	1	protein_coding	-58
ENSG0000(	CEP152	1	protein_coding	679
ENSG0000(	OCA2	1	protein_coding	330
ENSG0000(	GABPB1	1	protein_coding	82
ENSG0000(	EIF3J	1	protein_coding	747
ENSG0000(	SPG11	1	protein_coding	-79
ENSG0000(	VPS18	1	protein_coding	657
ENSG0000(	OIP5	1	protein_coding	478
ENSG0000(	SLC30A4	1	protein_coding	-492
ENSG0000(	BLOC1S6	1	protein_coding	-239
ENSG0000(	INTS9	1	protein_coding	-33
ENSG0000(	INTS9	1	protein_coding	654
ENSG0000(	RIPK2	1	protein_coding	-381
ENSG0000(	CPQ	1	protein_coding	-429
ENSG0000(	POP1	1	protein_coding	-50
ENSG0000(	GSDMD	1	protein_coding	704
ENSG0000(	TSTA3	1	protein_coding	362
ENSG0000(	SLC39A14	1	protein_coding	733
ENSG0000(	MTMR9	1	protein_coding	-120
ENSG0000(	LEPROTL1	1	protein_coding	-397
ENSG0000(	GSR	1	protein_coding	-265
ENSG0000(	KCTD9	1	protein_coding	-519
ENSG0000(	BNIP3L	1	protein_coding	133
ENSG0000(	DHDH	1	protein_coding	254
ENSG0000(	GYS1	1	protein_coding	-361
ENSG0000(	GYS1	1	protein_coding	-947
ENSG0000(	MAP4K1	1	protein_coding	-395
ENSG0000(	ECH1	1	protein_coding	431
ENSG0000(	NFKBIB	1	protein_coding	-435
ENSG0000(	NFKBIB	1	protein_coding	23
ENSG0000(	LIN7B	1	protein_coding	-273
ENSG0000(	PIH1D1	1	protein_coding	-85
ENSG0000(	PIH1D1	1	protein_coding	409
ENSG0000(	RNASEH2A	1	protein_coding	-133
ENSG0000(	SF3A2	1	protein_coding	144
ENSG0000(	LYL1	1	protein_coding	-234
ENSG0000(	TBC1D17	1	protein_coding	-529
ENSG0000(	TBC1D17	1	protein_coding	49
ENSG0000(	IL4I1	1	protein_coding	-131
ENSG0000(	IL4I1	1	protein_coding	460
ENSG0000(	TLE6	1	protein_coding	332
ENSG0000(	SGTA	1	protein_coding	-395
ENSG0000(	MED25	1	protein_coding	-151
ENSG0000(	SNAPC2	1	protein_coding	-286
ENSG0000(	SNAPC2	1	protein_coding	997
ENSG0000(	C19orf53	1	protein_coding	246
ENSG0000(	IL27RA	1	protein_coding	387

ENSG0000(	ASF1B	1	protein_coding	-586
ENSG0000(	ASF1B	1	protein_coding	114
ENSG0000(	VRK3	1	protein_coding	-95
ENSG0000(	PPP6R1	1	protein_coding	-422
ENSG0000(	ILVBL	1	protein_coding	171
ENSG0000(	FBL	1	protein_coding	-458
ENSG0000(	DYRK1B	1	protein_coding	-193
ENSG0000(	GPI	1	protein_coding	-303
ENSG0000(	AKT2	1	protein_coding	-383
ENSG0000(	CCDC94	1	protein_coding	-82
ENSG0000(	SLC1A5	1	protein_coding	126
ENSG0000(	CACTIN	1	protein_coding	-154
ENSG0000(	CCDC9	1	protein_coding	289
ENSG0000(	FZR1	1	protein_coding	-346
ENSG0000(	MRPL4	1	protein_coding	219
ENSG0000(	CD79A	1	protein_coding	760
ENSG0000(	CD79A	1	protein_coding	978
ENSG0000(	ICAM5	1	protein_coding	-524
ENSG0000(	ETFB	1	protein_coding	394
ENSG0000(	BABAM1	1	protein_coding	-270
ENSG0000(	TYK2	1	protein_coding	-388
ENSG0000(	TYK2	1	protein_coding	405
ENSG0000(	CDC37	1	protein_coding	256
ENSG0000(	ATP1A3	1	protein_coding	970
ENSG0000(	MEGF8	1	protein_coding	-444
ENSG0000(	GRWD1	1	protein_coding	-200
ENSG0000(	SYNGR4	1	protein_coding	-496
ENSG0000(	CLEC11A	1	protein_coding	436
ENSG0000(	CARD8	1	protein_coding	-703
ENSG0000(	CARD8	1	protein_coding	149
ENSG0000(	FAM83E	1	protein_coding	508
ENSG0000(	FAM83E	1	protein_coding	742
ENSG0000(	RASIP1	1	protein_coding	-313
ENSG0000(	RASIP1	1	protein_coding	516
ENSG0000(	TNPO2	1	protein_coding	588
ENSG0000(	KLF1	1	protein_coding	-22
ENSG0000(	KLF1	1	protein_coding	-786
ENSG0000(	KLF1	1	protein_coding	141
ENSG0000(	DNASE2	1	protein_coding	193
ENSG0000(	DNASE2	1	protein_coding	705
ENSG0000(	LENG1	1	protein_coding	-591
ENSG0000(	LENG1	1	protein_coding	232
ENSG0000(	PRPF31	1	protein_coding	151
ENSG0000(	TFPT	1	protein_coding	115
ENSG0000(	RPL18A	1	protein_coding	-125
ENSG0000(	RPL18A	1	protein_coding	391
ENSG0000(	ARRDC2	1	protein_coding	933
ENSG0000(	ISYNA1	1	protein_coding	-197
ENSG0000(	ISYNA1	1	protein_coding	-402
ENSG0000(	COPE	1	protein_coding	459
ENSG0000(	DDX49	1	protein_coding	-736
ENSG0000(	ARMC6	1	protein_coding	542
ENSG0000(	TMEM147	1	protein_coding	-74
ENSG0000(	KXD1	1	protein_coding	-64

ENSG0000(	SUGP1	1	protein_coding	-444
ENSG0000(	HPN	1	protein_coding	484
ENSG0000(	GSK3A	1	protein_coding	-190
ENSG0000(	SIPA1L3	1	protein_coding	-203
ENSG0000(	SMG9	1	protein_coding	-280
ENSG0000(	BET1	1	protein_coding	-45
ENSG0000(	NAMPT	1	protein_coding	285
ENSG0000(	TWISTNB	1	protein_coding	310
ENSG0000(	HBP1	1	protein_coding	-271
ENSG0000(	DUS4L	1	protein_coding	957
ENSG0000(	WDR91	1	protein_coding	274
ENSG0000(	CBLL1	1	protein_coding	324
ENSG0000(	MTPN	1	protein_coding	-89
ENSG0000(	ZC3HAV1	1	protein_coding	-255
ENSG0000(	ZC3HAV1	1	protein_coding	462
ENSG0000(	OGDH	1	protein_coding	714
ENSG0000(	H2AFV	1	protein_coding	-374
ENSG0000(	RNF32	1	protein_coding	-266
ENSG0000(	SSBP1	1	protein_coding	28
ENSG0000(	ABHD11	1	protein_coding	-635
ENSG0000(	ABHD11	1	protein_coding	295
ENSG0000(	FKBP14	1	protein_coding	75
ENSG0000(	PLEKHA8	1	protein_coding	-794
ENSG0000(	NOD1	1	protein_coding	250
ENSG0000(	GARS	1	protein_coding	137
ENSG0000(	EPHB6	1	protein_coding	401
ENSG0000(	CASP2	1	protein_coding	-279
ENSG0000(	CASP2	1	protein_coding	818
ENSG0000(	HSPB1	1	protein_coding	-227
ENSG0000(	PDAP1	1	protein_coding	-592
ENSG0000(	BUD31	1	protein_coding	781
ENSG0000(	MOSPD3	1	protein_coding	17
ENSG0000(	USP42	1	protein_coding	-369
ENSG0000(	IMPDH1	1	protein_coding	656
ENSG0000(	AGFG2	1	protein_coding	91
ENSG0000(	NRF1	1	protein_coding	-499
ENSG0000(	TMEM106B	1	protein_coding	-243
ENSG0000(	ZNF862	1	protein_coding	-96
ENSG0000(	ZNF862	1	protein_coding	551
ENSG0000(	ANKMY2	1	protein_coding	987
ENSG0000(	ACTR3C	1	protein_coding	-143
ENSG0000(	PSMA2	1	protein_coding	211
ENSG0000(	MRPL32	1	protein_coding	-187
ENSG0000(	URGCP	1	protein_coding	-235
ENSG0000(	TMEM248	1	protein_coding	-245
ENSG0000(	BCL7B	1	protein_coding	31
ENSG0000(	CLIP2	1	protein_coding	-455
ENSG0000(	TRIM14	1	protein_coding	-242
ENSG0000(	TRIM14	1	protein_coding	-501
ENSG0000(	CORO2A	1	protein_coding	-578
ENSG0000(	CORO2A	1	protein_coding	352
ENSG0000(	TGFBR1	1	protein_coding	662
ENSG0000(	DNM1	1	protein_coding	-386
ENSG0000(	AK1	1	protein_coding	-322

ENSG0000(	AK1	1	protein_coding	-715
ENSG0000(	AK1	1	protein_coding	475
ENSG0000(	KANK1	1	protein_coding	151
ENSG0000(	RGP1	1	protein_coding	714
ENSG0000(	ABCA2	1	protein_coding	13
ENSG0000(	SHB	1	protein_coding	-722
ENSG0000(	PHYH	1	protein_coding	-111
ENSG0000(	EIF3A	1	protein_coding	-355
ENSG0000(	TRDMT1	1	protein_coding	305
ENSG0000(	TRDMT1	1	protein_coding	957
ENSG0000(	DDX50	1	protein_coding	-459
ENSG0000(	MAPK8	1	protein_coding	-552
ENSG0000(	SEC23IP	1	protein_coding	-117
ENSG0000(	SEC23IP	1	protein_coding	59
ENSG0000(	MICU1	1	protein_coding	-268
ENSG0000(	MICU1	1	protein_coding	167
ENSG0000(	CCSER2	1	protein_coding	-350
ENSG0000(	TNKS2	1	protein_coding	-76
ENSG0000(	TNKS2	1	protein_coding	588
ENSG0000(	PITX3	1	protein_coding	-807
ENSG0000(	CPEB3	1	protein_coding	684
ENSG0000(	ACBD5	1	protein_coding	648
ENSG0000(	OBFC1	1	protein_coding	-482
ENSG0000(	FAM208B	1	protein_coding	-422
ENSG0000(	TFAM	1	protein_coding	513
ENSG0000(	CCDC6	1	protein_coding	-414
ENSG0000(	UBE2S	1	protein_coding	-638
ENSG0000(	RPL28	1	protein_coding	-605
ENSG0000(	PBLD	1	protein_coding	339
ENSG0000(	TBC1D12	1	protein_coding	-133
ENSG0000(	NUFIP2	1	protein_coding	-212
ENSG0000(	PSMD3	1	protein_coding	-127
ENSG0000(	CASC3	1	protein_coding	-428
ENSG0000(	RGS9	1	protein_coding	-338
ENSG0000(	MTMR4	1	protein_coding	-574
ENSG0000(	DHX40	1	protein_coding	-131
ENSG0000(	TUBD1	1	protein_coding	-745
ENSG0000(	GOSR2	1	protein_coding	-144
ENSG0000(	PNPO	1	protein_coding	-162
ENSG0000(	RPS6KB1	1	protein_coding	645
ENSG0000(	CBX1	1	protein_coding	-106
ENSG0000(	PIGL	1	protein_coding	3
ENSG0000(	CHRNE	1	protein_coding	106
ENSG0000(	NUP88	1	protein_coding	-13
ENSG0000(	SMARCD2	1	protein_coding	-198
ENSG0000(	B9D1	1	protein_coding	105
ENSG0000(	UTP6	1	protein_coding	44
ENSG0000(	C17orf75	1	protein_coding	-153
ENSG0000(	CYTH1	1	protein_coding	970
ENSG0000(	NAGLU	1	protein_coding	-500
ENSG0000(	NAGLU	1	protein_coding	-816
ENSG0000(	NAGLU	1	protein_coding	571
ENSG0000(	EZH1	1	protein_coding	674
ENSG0000(	DLX4	1	protein_coding	579

ENSG0000(	PTGES3L-AARSD1	1	protein_coding	-480
ENSG0000(	PTGES3L-AARSD1	1	protein_coding	30
ENSG0000(	MRPL27	1	protein_coding	186
ENSG0000(	HDAC5	1	protein_coding	-380
ENSG0000(	EFTUD2	1	protein_coding	122
ENSG0000(	PRKAR1A	1	protein_coding	601
ENSG0000(	DPH1	1	protein_coding	456
ENSG0000(	TNFAIP1	1	protein_coding	-34
ENSG0000(	IFT20	1	protein_coding	-78
ENSG0000(	ALDOC	1	protein_coding	61
ENSG0000(	PHF12	1	protein_coding	-597
ENSG0000(	NMU	1	protein_coding	-285
ENSG0000(	ZNF330	1	protein_coding	22
ENSG0000(	GAB1	1	protein_coding	-807
ENSG0000(	RPL34	1	protein_coding	-20
ENSG0000(	GRPEL1	1	protein_coding	75
ENSG0000(	GAR1	1	protein_coding	-197
ENSG0000(	DHX15	1	protein_coding	-121
ENSG0000(	SEPSECS	1	protein_coding	-190
ENSG0000(	FBXW7	1	protein_coding	-448
ENSG0000(	NKX3-2	1	protein_coding	-373
ENSG0000(	MFSD10	1	protein_coding	-182
ENSG0000(	MFSD10	1	protein_coding	-563
ENSG0000(	UGDH	1	protein_coding	-63
ENSG0000(	UGDH	1	protein_coding	126
ENSG0000(	UGDH	1	protein_coding	396
ENSG0000(	HTATIP2	1	protein_coding	-81
ENSG0000(	HTATIP2	1	protein_coding	591
ENSG0000(	HTATIP2	1	protein_coding	961
ENSG0000(	ZPR1	1	protein_coding	-129
ENSG0000(	MTCH2	1	protein_coding	-285
ENSG0000(	MTCH2	1	protein_coding	-964
ENSG0000(	FNBP4	1	protein_coding	-245
ENSG0000(	FNBP4	1	protein_coding	73
ENSG0000(	EHD1	1	protein_coding	-828
ENSG0000(	EHD1	1	protein_coding	238
ENSG0000(	OSBP	1	protein_coding	-432
ENSG0000(	UNC93B1	1	protein_coding	684
ENSG0000(	NRXN2	1	protein_coding	-430
ENSG0000(	CCDC86	1	protein_coding	-253
ENSG0000(	CCDC86	1	protein_coding	-672
ENSG0000(	TMEM109	1	protein_coding	336
ENSG0000(	CHORDC1	1	protein_coding	637
ENSG0000(	ANAPC15	1	protein_coding	256
ENSG0000(	PANX1	1	protein_coding	-246
ENSG0000(	APOA4	1	protein_coding	548
ENSG0000(	RNF141	1	protein_coding	-281
ENSG0000(	IL10RA	1	protein_coding	165
ENSG0000(	BIRC2	1	protein_coding	-409
ENSG0000(	UPK2	1	protein_coding	-461
ENSG0000(	NAA40	1	protein_coding	720
ENSG0000(	CARS	1	protein_coding	-315
ENSG0000(	C11orf21	1	protein_coding	392
ENSG0000(	PITPNM1	1	protein_coding	402

ENSG0000(	RPS13	1	protein_coding	147
ENSG0000(	AIP	1	protein_coding	257
ENSG0000(	NUP98	1	protein_coding	-342
ENSG0000(	NDUFS8	1	protein_coding	-374
ENSG0000(	NDUFS8	1	protein_coding	428
ENSG0000(	CHKA	1	protein_coding	-352
ENSG0000(	PSMD9	1	protein_coding	-133
ENSG0000(	P3H3	1	protein_coding	-958
ENSG0000(	PRPF40B	1	protein_coding	13
ENSG0000(	PRDM4	1	protein_coding	-129
ENSG0000(	PRDM4	1	protein_coding	805
ENSG0000(	SELPLG	1	protein_coding	113
ENSG0000(	CAPRIN2	1	protein_coding	-409
ENSG0000(	CAPRIN2	1	protein_coding	815
ENSG0000(	CSRNP2	1	protein_coding	23
ENSG0000(	IL23A	1	protein_coding	-343
ENSG0000(	IL23A	1	protein_coding	-982
ENSG0000(	ATP5B	1	protein_coding	-619
ENSG0000(	PTGES3	1	protein_coding	777
ENSG0000(	RSRC2	1	protein_coding	-393
ENSG0000(	KRT18	1	protein_coding	-573
ENSG0000(	KRT18	1	protein_coding	905
ENSG0000(	GLI1	1	protein_coding	-906
ENSG0000(	MAGOHB	1	protein_coding	27
ENSG0000(	ITFG2	1	protein_coding	-164
ENSG0000(	ITFG2	1	protein_coding	356
ENSG0000(	ARPC3	1	protein_coding	556
ENSG0000(	GPN3	1	protein_coding	-122
ENSG0000(	GPN3	1	protein_coding	763
ENSG0000(	VPS29	1	protein_coding	-241
ENSG0000(	VPS29	1	protein_coding	714
ENSG0000(	NAA25	1	protein_coding	195
ENSG0000(	OGFOD2	1	protein_coding	177
ENSG0000(	OGFOD2	1	protein_coding	635
ENSG0000(	GTF2H3	1	protein_coding	-426
ENSG0000(	EIF2B1	1	protein_coding	454
ENSG0000(	DDX55	1	protein_coding	-279
ENSG0000(	COPZ1	1	protein_coding	88
ENSG0000(	CAND1	1	protein_coding	-387
ENSG0000(	NUP107	1	protein_coding	20
ENSG0000(	CNOT2	1	protein_coding	289
ENSG0000(	CPSF6	1	protein_coding	-269
ENSG0000(	KRR1	1	protein_coding	73
ENSG0000(	COPS7A	1	protein_coding	577
ENSG0000(	CDCA3	1	protein_coding	317
ENSG0000(	CHPT1	1	protein_coding	71
ENSG0000(	USP5	1	protein_coding	-378
ENSG0000(	SUDS3	1	protein_coding	-138
ENSG0000(	C2CD5	1	protein_coding	-187
ENSG0000(	COX6A1	1	protein_coding	-413
ENSG0000(	AL021546.6	1	protein_coding	-521
ENSG0000(	RIC8B	1	protein_coding	-247
ENSG0000(	SRSF9	1	protein_coding	-418
ENSG0000(	TDP2	1	protein_coding	405

ENSG0000(	RWDD1	1	protein_coding	-142
ENSG0000(	TMEM14C	1	protein_coding	-160
ENSG0000(	PAK1IP1	1	protein_coding	218
ENSG0000(	SMIM8	1	protein_coding	440
ENSG0000(	HINT3	1	protein_coding	69
ENSG0000(	ULBP1	1	protein_coding	-70
ENSG0000(	FBXO5	1	protein_coding	-306
ENSG0000(	MTRF1L	1	protein_coding	-650
ENSG0000(	MTRF1L	1	protein_coding	474
ENSG0000(	PPARD	1	protein_coding	-247
ENSG0000(	MAPK14	1	protein_coding	-180
ENSG0000(	RHAG	1	protein_coding	91
ENSG0000(	SRSF3	1	protein_coding	-272
ENSG0000(	SOD2	1	protein_coding	271
ENSG0000(	MRPL18	1	protein_coding	448
ENSG0000(	ICK	1	protein_coding	-122
ENSG0000(	MDN1	1	protein_coding	-318
ENSG0000(	MDN1	1	protein_coding	25
ENSG0000(	SAYSD1	1	protein_coding	-174
ENSG0000(	E2F3	1	protein_coding	-703
ENSG0000(	ACOT13	1	protein_coding	-406
ENSG0000(	RPS12	1	protein_coding	-154
ENSG0000(	C6orf62	1	protein_coding	-893
ENSG0000(	GMNN	1	protein_coding	-34
ENSG0000(	SNX3	1	protein_coding	-235
ENSG0000(	SNX3	1	protein_coding	928
ENSG0000(	EPM2A	1	protein_coding	238
ENSG0000(	SLC39A7	1	protein_coding	-524
ENSG0000(	SLC39A7	1	protein_coding	370
ENSG0000(	CUTA	1	protein_coding	-460
ENSG0000(	CUTA	1	protein_coding	158
ENSG0000(	PACRG	1	protein_coding	745
ENSG0000(	TFEB	1	protein_coding	469
ENSG0000(	CCND3	1	protein_coding	-574
ENSG0000(	TBP	1	protein_coding	-389
ENSG0000(	GLTSCR1L	1	protein_coding	743
ENSG0000(	PPP2R5D	1	protein_coding	-495
ENSG0000(	PTK7	1	protein_coding	-242
ENSG0000(	CUL9	1	protein_coding	-402
ENSG0000(	CUL9	1	protein_coding	419
ENSG0000(	CUL9	1	protein_coding	772
ENSG0000(	DNPH1	1	protein_coding	-216
ENSG0000(	DNPH1	1	protein_coding	456
ENSG0000(	EXOC2	1	protein_coding	-31
ENSG0000(	EXOC2	1	protein_coding	-317
ENSG0000(	TMEM30A	1	protein_coding	-3
ENSG0000(	GMDS	1	protein_coding	-302
ENSG0000(	VEGFA	1	protein_coding	-412
ENSG0000(	PRPF4B	1	protein_coding	-208
ENSG0000(	SLC29A1	1	protein_coding	-330
ENSG0000(	FBRSL1	1	protein_coding	-662
ENSG0000(	HARS2	1	protein_coding	58
ENSG0000(	PAPD7	1	protein_coding	-344
ENSG0000(	HMGCS1	1	protein_coding	-296

ENSG0000(	BRD8	1	protein_coding	354
ENSG0000(	KIF20A	1	protein_coding	-86
ENSG0000(	MRPS30	1	protein_coding	-7
ENSG0000(	HSPA9	1	protein_coding	-426
ENSG0000(	HSPA9	1	protein_coding	183
ENSG0000(	APBB3	1	protein_coding	-13
ENSG0000(	APBB3	1	protein_coding	245
ENSG0000(	TMCO6	1	protein_coding	-275
ENSG0000(	IK	1	protein_coding	792
ENSG0000(	COL4A3BP	1	protein_coding	95
ENSG0000(	FAF2	1	protein_coding	259
ENSG0000(	FAF2	1	protein_coding	549
ENSG0000(	PCDHB15	1	protein_coding	489
ENSG0000(	ITK	1	protein_coding	-121
ENSG0000(	RNF130	1	protein_coding	-58
ENSG0000(	CNOT6	1	protein_coding	790
ENSG0000(	MSH3	1	protein_coding	788
ENSG0000(	CCNG1	1	protein_coding	488
ENSG0000(	TARS	1	protein_coding	666
ENSG0000(	BRIX1	1	protein_coding	273
ENSG0000(	RAD50	1	protein_coding	842
ENSG0000(	IL5	1	protein_coding	-22
ENSG0000(	NUP155	1	protein_coding	-516
ENSG0000(	PPP2CA	1	protein_coding	-290
ENSG0000(	SEC24A	1	protein_coding	-248
ENSG0000(	SEC24A	1	protein_coding	293
ENSG0000(	TTC33	1	protein_coding	-63
ENSG0000(	TTC33	1	protein_coding	-316
ENSG0000(	RARS	1	protein_coding	-16
ENSG0000(	H2AFY	1	protein_coding	417
ENSG0000(	TCERG1	1	protein_coding	-224
ENSG0000(	CSNK1A1	1	protein_coding	-149
ENSG0000(	HMGXB3	1	protein_coding	531
ENSG0000(	CPEB4	1	protein_coding	-346
ENSG0000(	DBN1	1	protein_coding	-540
ENSG0000(	ZNF346	1	protein_coding	715
ENSG0000(	SMC4	1	protein_coding	372
ENSG0000(	SELK	1	protein_coding	-106
ENSG0000(	ARMC8	1	protein_coding	94
ENSG0000(	PDCD10	1	protein_coding	98
ENSG0000(	PRKAR2A	1	protein_coding	-25
ENSG0000(	PRKAR2A	1	protein_coding	795
ENSG0000(	TFG	1	protein_coding	178
ENSG0000(	RPL24	1	protein_coding	668
ENSG0000(	IQCG	1	protein_coding	-592
ENSG0000(	NCBP2	1	protein_coding	-700
ENSG0000(	NCBP2	1	protein_coding	74
ENSG0000(	KLHL18	1	protein_coding	-337
ENSG0000(	KLHL18	1	protein_coding	-812
ENSG0000(	SCAP	1	protein_coding	422
ENSG0000(	SCAP	1	protein_coding	993
ENSG0000(	MRPL3	1	protein_coding	522
ENSG0000(	HEMK1	1	protein_coding	-989
ENSG0000(	CISH	1	protein_coding	924

ENSG0000(	MAPKAPK3	1	protein_coding	-612
ENSG0000(	WDR48	1	protein_coding	-14
ENSG0000(	GORASP1	1	protein_coding	656
ENSG0000(	ABHD14B	1	protein_coding	-704
ENSG0000(	ABHD14B	1	protein_coding	408
ENSG0000(	ABHD14B	1	protein_coding	790
ENSG0000(	EIF1B	1	protein_coding	-319
ENSG0000(	EIF1B	1	protein_coding	-892
ENSG0000(	ABHD14A-ACY1	1	protein_coding	-617
ENSG0000(	ABHD14A-ACY1	1	protein_coding	665
ENSG0000(	KLHL24	1	protein_coding	-77
ENSG0000(	CLCN2	1	protein_coding	453
ENSG0000(	LMAN2L	1	protein_coding	-476
ENSG0000(	NCL	1	protein_coding	849
ENSG0000(	ACTR1B	1	protein_coding	938
ENSG0000(	SF3B6	1	protein_coding	226
ENSG0000(	DNAJC27	1	protein_coding	-182
ENSG0000(	DNAJC27	1	protein_coding	865
ENSG0000(	STAM2	1	protein_coding	54
ENSG0000(	ACVR1	1	protein_coding	471
ENSG0000(	GTF3C2	1	protein_coding	498
ENSG0000(	EIF2B4	1	protein_coding	-1
ENSG0000(	PSMD14	1	protein_coding	633
ENSG0000(	SNX17	1	protein_coding	-8
ENSG0000(	PPM1G	1	protein_coding	-40
ENSG0000(	PPM1G	1	protein_coding	-411
ENSG0000(	RPS15	1	protein_coding	-24
ENSG0000(	TTC31	1	protein_coding	-352
ENSG0000(	TTC31	1	protein_coding	302
ENSG0000(	AUP1	1	protein_coding	643
ENSG0000(	RTN4	1	protein_coding	-68
ENSG0000(	RTN4	1	protein_coding	563
ENSG0000(	HTRA2	1	protein_coding	-80
ENSG0000(	UNC50	1	protein_coding	-114
ENSG0000(	IGFBP5	1	protein_coding	-813
ENSG0000(	CCT4	1	protein_coding	109
ENSG0000(	OTX1	1	protein_coding	866
ENSG0000(	CHMP3	1	protein_coding	-173
ENSG0000(	IL1R1	1	protein_coding	-136
ENSG0000(	IL1R1	1	protein_coding	812
ENSG0000(	FHL2	1	protein_coding	-442
ENSG0000(	ABCB6	1	protein_coding	118
ENSG0000(	STK16	1	protein_coding	142
ENSG0000(	ID2	1	protein_coding	743
ENSG0000(	TAF1B	1	protein_coding	-247
ENSG0000(	NOL10	1	protein_coding	35
ENSG0000(	GORASP2	1	protein_coding	30
ENSG0000(	DCAF17	1	protein_coding	-560
ENSG0000(	DCAF17	1	protein_coding	246
ENSG0000(	SLC25A12	1	protein_coding	-241
ENSG0000(	DARS	1	protein_coding	109
ENSG0000(	DARS	1	protein_coding	521
ENSG0000(	DARS	1	protein_coding	950
ENSG0000(	SRSF7	1	protein_coding	-84

ENSG0000(	SLC1A4	1	protein_coding	928
ENSG0000(	ATF2	1	protein_coding	402
ENSG0000(	THADA	1	protein_coding	-80
ENSG0000(	DNAJC16	1	protein_coding	-70
ENSG0000(	CACYBP	1	protein_coding	20
ENSG0000(	SCP2	1	protein_coding	427
ENSG0000(	SCP2	1	protein_coding	699
ENSG0000(	CEP104	1	protein_coding	-158
ENSG0000(	TCEANC2	1	protein_coding	-905
ENSG0000(	TMEM59	1	protein_coding	823
ENSG0000(	RPL22	1	protein_coding	-54
ENSG0000(	QSOX1	1	protein_coding	-715
ENSG0000(	STXBP3	1	protein_coding	-84
ENSG0000(	STXBP3	1	protein_coding	776
ENSG0000(	SRSF4	1	protein_coding	-286
ENSG0000(	SRSF4	1	protein_coding	782
ENSG0000(	MECR	1	protein_coding	-305
ENSG0000(	HDAC1	1	protein_coding	816
ENSG0000(	CAPZA1	1	protein_coding	418
ENSG0000(	CAPZA1	1	protein_coding	778
ENSG0000(	S100PBP	1	protein_coding	239
ENSG0000(	SCAMP3	1	protein_coding	333
ENSG0000(	DLGAP3	1	protein_coding	639
ENSG0000(	LAMTOR2	1	protein_coding	-313
ENSG0000(	LAMTOR2	1	protein_coding	445
ENSG0000(	MEF2D	1	protein_coding	-645
ENSG0000(	MEF2D	1	protein_coding	-928
ENSG0000(	SRM	1	protein_coding	-988
ENSG0000(	SWT1	1	protein_coding	-82
ENSG0000(	SWT1	1	protein_coding	436
ENSG0000(	LEPR	1	protein_coding	745
ENSG0000(	IVNS1ABP	1	protein_coding	-719
ENSG0000(	KIAA2013	1	protein_coding	-305
ENSG0000(	MFN2	1	protein_coding	-362
ENSG0000(	MFN2	1	protein_coding	675
ENSG0000(	SMG7	1	protein_coding	-186
ENSG0000(	TROVE2	1	protein_coding	-470
ENSG0000(	TROVE2	1	protein_coding	502
ENSG0000(	TROVE2	1	protein_coding	759
ENSG0000(	UCHL5	1	protein_coding	-100
ENSG0000(	UCHL5	1	protein_coding	157
ENSG0000(	BCAS2	1	protein_coding	-276
ENSG0000(	CTH	1	protein_coding	-290
ENSG0000(	PHTF1	1	protein_coding	-299
ENSG0000(	ZBTB17	1	protein_coding	-164
ENSG0000(	CD58	1	protein_coding	674
ENSG0000(	KIF21B	1	protein_coding	900
ENSG0000(	TMEM9	1	protein_coding	76
ENSG0000(	ADPRHL2	1	protein_coding	603
ENSG0000(	MRPS15	1	protein_coding	-472
ENSG0000(	MRPS15	1	protein_coding	878
ENSG0000(	EXOC8	1	protein_coding	-128
ENSG0000(	GNPAT	1	protein_coding	-280
ENSG0000(	TSNAX	1	protein_coding	-60

ENSG0000(	C1orf109	1	protein_coding	-18
ENSG0000(	RRAGC	1	protein_coding	-499
ENSG0000(	RRAGC	1	protein_coding	808
ENSG0000(	MTR	1	protein_coding	-361
ENSG0000(	MTR	1	protein_coding	500
ENSG0000(	BMP8B	1	protein_coding	-601
ENSG0000(	MYCL	1	protein_coding	-225
ENSG0000(	RLF	1	protein_coding	-107
ENSG0000(	KCNQ4	1	protein_coding	-655
ENSG0000(	ACADM	1	protein_coding	-262
ENSG0000(	MFAP2	1	protein_coding	-851
ENSG0000(	RPF1	1	protein_coding	78
ENSG0000(	CTBS	1	protein_coding	470
ENSG0000(	RBBP5	1	protein_coding	-293
ENSG0000(	RAB29	1	protein_coding	28
ENSG0000(	ECE1	1	protein_coding	-849
ENSG0000(	ID3	1	protein_coding	399
ENSG0000(	CD46	1	protein_coding	449
ENSG0000(	PRPF3	1	protein_coding	250
ENSG0000(	P3H1	1	protein_coding	-286
ENSG0000(	P3H1	1	protein_coding	953
ENSG0000(	SLC2A1	1	protein_coding	16
ENSG0000(	CDC20	1	protein_coding	-649
ENSG0000(	IPO13	1	protein_coding	-85
ENSG0000(	ATP6V0B	1	protein_coding	125
ENSG0000(	AKR1A1	1	protein_coding	232
ENSG0000(	AKR1A1	1	protein_coding	895
ENSG0000(	PRDX1	1	protein_coding	855
ENSG0000(	PIK3R3	1	protein_coding	-490
ENSG0000(	TSPAN1	1	protein_coding	125
ENSG0000(	BLZF1	1	protein_coding	-122
ENSG0000(	SLC19A2	1	protein_coding	-297
ENSG0000(	NSUN4	1	protein_coding	-476
ENSG0000(	NSUN4	1	protein_coding	588
ENSG0000(	TMED5	1	protein_coding	568
ENSG0000(	DR1	1	protein_coding	-90
ENSG0000(	PRRC2C	1	protein_coding	-113
ENSG0000(	DPH5	1	protein_coding	-169
ENSG0000(	PRDX6	1	protein_coding	952
ENSG0000(	DARS2	1	protein_coding	379
ENSG0000(	DIEXF	1	protein_coding	-174
ENSG0000(	SYF2	1	protein_coding	-455
ENSG0000(	SLC35A3	1	protein_coding	171
ENSG0000(	SLC35A3	1	protein_coding	583
ENSG0000(	MTFR1L	1	protein_coding	-314
ENSG0000(	NEK2	1	protein_coding	-297
ENSG0000(	RPS6KA1	1	protein_coding	598
ENSG0000(	DHDDS	1	protein_coding	50
ENSG0000(	NSL1	1	protein_coding	-82
ENSG0000(	ARID1A	1	protein_coding	-209
ENSG0000(	CENPF	1	protein_coding	-206
ENSG0000(	RPA2	1	protein_coding	-180
ENSG0000(	STX12	1	protein_coding	654
ENSG0000(	TXNDC12	1	protein_coding	-752

ENSG0000(	TXNDC12	1	protein_coding	672
ENSG0000(	ESYT2	1	protein_coding	-250
ENSG0000(	ESYT2	1	protein_coding	-797
ENSG0000(	STAG1	1	protein_coding	-764
ENSG0000(	SLC8A2	1	protein_coding	-49
ENSG0000(	KPTN	1	protein_coding	92
ENSG0000(	KPTN	1	protein_coding	645
ENSG0000(	RPS25	1	protein_coding	-554
ENSG0000(	RPS25	1	protein_coding	238
ENSG0000(	KIF14	1	protein_coding	96
ENSG0000(	TNNT2	1	protein_coding	683
ENSG0000(	DDX59	1	protein_coding	-498
ENSG0000(	USP35	1	protein_coding	-453
ENSG0000(	ANKRD13C	1	protein_coding	-211
ENSG0000(	FBXO30	1	protein_coding	573
ENSG0000(	TNFAIP3	1	protein_coding	-821
ENSG0000(	TNFAIP3	1	protein_coding	515
ENSG0000(	ZNF430	1	protein_coding	568
ENSG0000(	DCLRE1B	1	protein_coding	-420
ENSG0000(	DCLRE1B	1	protein_coding	205
ENSG0000(	CCNI	1	protein_coding	-432
ENSG0000(	HS1BP3	1	protein_coding	25
ENSG0000(	DNAH7	1	protein_coding	228
ENSG0000(	UBE2B	1	protein_coding	-181
ENSG0000(	ITGB1BP1	1	protein_coding	-756
ENSG0000(	CPSF3	1	protein_coding	736
ENSG0000(	PIGZ	1	protein_coding	-228
ENSG0000(	HEATR1	1	protein_coding	-166
ENSG0000(	FAM206A	1	protein_coding	516
ENSG0000(	WDR34	1	protein_coding	-277
ENSG0000(	CNTRL	1	protein_coding	-258
ENSG0000(	FBXW2	1	protein_coding	-455
ENSG0000(	PHF19	1	protein_coding	751
ENSG0000(	PPP6C	1	protein_coding	-65
ENSG0000(	PPP6C	1	protein_coding	-557
ENSG0000(	RBM18	1	protein_coding	-367
ENSG0000(	NR4A3	1	protein_coding	-38
ENSG0000(	NR4A3	1	protein_coding	537
ENSG0000(	DENND1A	1	protein_coding	-630
ENSG0000(	VPS4B	1	protein_coding	-160
ENSG0000(	DCAF4	1	protein_coding	798
ENSG0000(	NEK9	1	protein_coding	286
ENSG0000(	IRF2BPL	1	protein_coding	-730
ENSG0000(	NRDE2	1	protein_coding	240
ENSG0000(	COQ6	1	protein_coding	41
ENSG0000(	RHOQ	1	protein_coding	155
ENSG0000(	SUPT7L	1	protein_coding	110
ENSG0000(	TMEM214	1	protein_coding	28
ENSG0000(	TMEM214	1	protein_coding	597
ENSG0000(	FAM98A	1	protein_coding	855
ENSG0000(	TCTN3	1	protein_coding	424
ENSG0000(	PANK3	1	protein_coding	-498
ENSG0000(	RCL1	1	protein_coding	-482
ENSG0000(	NUP43	1	protein_coding	-350

ENSG0000(	PCMT1	1	protein_coding	573
ENSG0000(	CYSTM1	1	protein_coding	200
ENSG0000(	WDR55	1	protein_coding	55
ENSG0000(	MRPS14	1	protein_coding	-71
ENSG0000(	CENPL	1	protein_coding	-161
ENSG0000(	GORAB	1	protein_coding	-39
ENSG0000(	GORAB	1	protein_coding	364
ENSG0000(	TCP1	1	protein_coding	-510
ENSG0000(	SLC10A7	1	protein_coding	-50
ENSG0000(	SLC10A7	1	protein_coding	-347
ENSG0000(	SLC10A7	1	protein_coding	-729
ENSG0000(	NUDCD1	1	protein_coding	186
ENSG0000(	ENY2	1	protein_coding	-124
ENSG0000(	MASTL	1	protein_coding	-654
ENSG0000(	MASTL	1	protein_coding	761
ENSG0000(	TAF12	1	protein_coding	-83
ENSG0000(	WBP4	1	protein_coding	-6
ENSG0000(	ELF1	1	protein_coding	173
ENSG0000(	HSPH1	1	protein_coding	-102
ENSG0000(	ETF1	1	protein_coding	-382
ENSG0000(	FAM53C	1	protein_coding	-295
ENSG0000(	SIL1	1	protein_coding	-798
ENSG0000(	PAIP2	1	protein_coding	240
ENSG0000(	EGR1	1	protein_coding	-912
ENSG0000(	SERP1	1	protein_coding	73
ENSG0000(	PLS1	1	protein_coding	-278
ENSG0000(	PLS1	1	protein_coding	72
ENSG0000(	TMPO	1	protein_coding	206
ENSG0000(	SOCS2	1	protein_coding	-346
ENSG0000(	APAF1	1	protein_coding	-348
ENSG0000(	APAF1	1	protein_coding	-870
ENSG0000(	TNFRSF10B	1	protein_coding	-226
ENSG0000(	EPHX2	1	protein_coding	665
ENSG0000(	NPPB	1	protein_coding	-558
ENSG0000(	UBIAD1	1	protein_coding	379
ENSG0000(	UBIAD1	1	protein_coding	775
ENSG0000(	TNFRSF8	1	protein_coding	859
ENSG0000(	LYPLA1	1	protein_coding	774
ENSG0000(	SLC35B1	1	protein_coding	695
ENSG0000(	NCAPH	1	protein_coding	536
ENSG0000(	LRAT	1	protein_coding	850
ENSG0000(	MND1	1	protein_coding	12
ENSG0000(	CEP89	1	protein_coding	-3
ENSG0000(	ECHDC2	1	protein_coding	-443
ENSG0000(	ECHDC2	1	protein_coding	-715
ENSG0000(	RNF2	1	protein_coding	-583
ENSG0000(	TRMT1L	1	protein_coding	-443
ENSG0000(	TRMT1L	1	protein_coding	75
ENSG0000(	POPDC2	1	protein_coding	195
ENSG0000(	CAT	1	protein_coding	416
ENSG0000(	TBC1D15	1	protein_coding	30
ENSG0000(	TBC1D15	1	protein_coding	447
ENSG0000(	KHDRBS1	1	protein_coding	59
ENSG0000(	TMEM39B	1	protein_coding	422

ENSG0000(	CCRL2	1	protein_coding	-238
ENSG0000(	PDS5A	1	protein_coding	-247
ENSG0000(	LIAS	1	protein_coding	-112
ENSG0000(	LRIF1	1	protein_coding	358
ENSG0000(	CLCC1	1	protein_coding	334
ENSG0000(	GTDC1	1	protein_coding	-231
ENSG0000(	POLK	1	protein_coding	288
ENSG0000(	KIAA1191	1	protein_coding	82
ENSG0000(	ZC3H7A	1	protein_coding	-420
ENSG0000(	SERAC1	1	protein_coding	-296
ENSG0000(	ANXA11	1	protein_coding	-427
ENSG0000(	RPL5	1	protein_coding	179
ENSG0000(	TRMT13	1	protein_coding	-150
ENSG0000(	ZNF644	1	protein_coding	-271
ENSG0000(	ZNF644	1	protein_coding	159
ENSG0000(	CCDC18	1	protein_coding	242
ENSG0000(	7-Sep	1	protein_coding	-356
ENSG0000(	7-Sep	1	protein_coding	189
ENSG0000(	ARL4A	1	protein_coding	-79
ENSG0000(	SMU1	1	protein_coding	405
ENSG0000(	SLC25A51	1	protein_coding	-202
ENSG0000(	C7orf49	1	protein_coding	-4
ENSG0000(	C7orf49	1	protein_coding	825
ENSG0000(	AKR1D1	1	protein_coding	-391
ENSG0000(	ECD	1	protein_coding	321
ENSG0000(	P4HA1	1	protein_coding	154
ENSG0000(	SLC25A16	1	protein_coding	412
ENSG0000(	VPS26A	1	protein_coding	120
ENSG0000(	CDKN2C	1	protein_coding	-210
ENSG0000(	RNF11	1	protein_coding	-139
ENSG0000(	RNF11	1	protein_coding	787
ENSG0000(	ITPR2	1	protein_coding	953
ENSG0000(	DDX39A	1	protein_coding	-280
ENSG0000(	DDX39A	1	protein_coding	65
ENSG0000(	NR4A1	1	protein_coding	984
ENSG0000(	NFE2	1	protein_coding	-168
ENSG0000(	TUBA1B	1	protein_coding	935
ENSG0000(	KBTBD4	1	protein_coding	56
ENSG0000(	ATPAF1	1	protein_coding	892
ENSG0000(	STIL	1	protein_coding	284
ENSG0000(	AMD1	1	protein_coding	-439
ENSG0000(	USP45	1	protein_coding	274
ENSG0000(	METTL8	1	protein_coding	340
ENSG0000(	TTC21B	1	protein_coding	-315
ENSG0000(	NMI	1	protein_coding	97
ENSG0000(	BAZ2B	1	protein_coding	279
ENSG0000(	EXOSC9	1	protein_coding	-73
ENSG0000(	PLA2G12A	1	protein_coding	-366
ENSG0000(	B9D2	1	protein_coding	-284
ENSG0000(	CTCFL	1	protein_coding	604
ENSG0000(	NCOA3	1	protein_coding	-383
ENSG0000(	VAPB	1	protein_coding	715
ENSG0000(	ATP5E	1	protein_coding	451
ENSG0000(	SRSF6	1	protein_coding	-341

ENSG0000(	ZNFX1	1	protein_coding	-351
ENSG0000(	ZNFX1	1	protein_coding	-733
ENSG0000(	CSE1L	1	protein_coding	703
ENSG0000(	TMEM189-UBE2V1	1	protein_coding	-570
ENSG0000(	TMEM189-UBE2V1	1	protein_coding	132
ENSG0000(	SNAI1	1	protein_coding	-703
ENSG0000(	NEURL2	1	protein_coding	-60
ENSG0000(	NEURL2	1	protein_coding	135
ENSG0000(	FASTKD3	1	protein_coding	-796
ENSG0000(	STAMBP	1	protein_coding	17
ENSG0000(	ZNF576	1	protein_coding	125
ENSG0000(	SIRT5	1	protein_coding	-723
ENSG0000(	WRNIP1	1	protein_coding	-48
ENSG0000(	SNRPC	1	protein_coding	253
ENSG0000(	XPO5	1	protein_coding	-303
ENSG0000(	XPO5	1	protein_coding	526
ENSG0000(	ABCC10	1	protein_coding	638
ENSG0000(	PEX6	1	protein_coding	-260
ENSG0000(	NQO2	1	protein_coding	-475
ENSG0000(	NQO2	1	protein_coding	627
ENSG0000(	ZNF391	1	protein_coding	600
ENSG0000(	RPS10	1	protein_coding	-102
ENSG0000(	RPS10	1	protein_coding	383
ENSG0000(	RPS10	1	protein_coding	742
ENSG0000(	HIST1H2BJ	1	protein_coding	-181
ENSG0000(	TBCC	1	protein_coding	176
ENSG0000(	MAD2L1BP	1	protein_coding	352
ENSG0000(	KLHDC3	1	protein_coding	-794
ENSG0000(	MEA1	1	protein_coding	660
ENSG0000(	RIOK1	1	protein_coding	203
ENSG0000(	RPP40	1	protein_coding	-47
ENSG0000(	RPP40	1	protein_coding	-475
ENSG0000(	RPP40	1	protein_coding	505
ENSG0000(	NUP153	1	protein_coding	-263
ENSG0000(	NUP153	1	protein_coding	532
ENSG0000(	NUP153	1	protein_coding	766
ENSG0000(	EREG	1	protein_coding	-138
ENSG0000(	AHNAK	1	protein_coding	-197
ENSG0000(	EMC3	1	protein_coding	132
ENSG0000(	CNOT1	1	protein_coding	-476
ENSG0000(	LRRC29	1	protein_coding	-92
ENSG0000(	GOT2	1	protein_coding	-894
ENSG0000(	GOT2	1	protein_coding	533
ENSG0000(	ABCC4	1	protein_coding	575
ENSG0000(	C17orf53	1	protein_coding	-67
ENSG0000(	C17orf53	1	protein_coding	716
ENSG0000(	IRF1	1	protein_coding	-672
ENSG0000(	IRF1	1	protein_coding	41
ENSG0000(	RNF113A	1	protein_coding	-128
ENSG0000(	NDUFA1	1	protein_coding	469
ENSG0000(	ATP5S	1	protein_coding	-889
ENSG0000(	GRK4	1	protein_coding	-528
ENSG0000(	MRPS7	1	protein_coding	-699
ENSG0000(	NUP85	1	protein_coding	-34

ENSG0000(	SLC25A19	1	protein_coding	-123
ENSG0000(	MIF4GD	1	protein_coding	366
ENSG0000(	MIF4GD	1	protein_coding	682
ENSG0000(	TTF1	1	protein_coding	-101
ENSG0000(	GTF3C4	1	protein_coding	-321
ENSG0000(	GTF3C4	1	protein_coding	198
ENSG0000(	DDX31	1	protein_coding	169
ENSG0000(	DDX31	1	protein_coding	688
ENSG0000(	BARHL1	1	protein_coding	19
ENSG0000(	POLR1B	1	protein_coding	563
ENSG0000(	GTF2F1	1	protein_coding	-241
ENSG0000(	CLPP	1	protein_coding	-42
ENSG0000(	THOC2	1	protein_coding	-87
ENSG0000(	THOC2	1	protein_coding	813
ENSG0000(	RPL23	1	protein_coding	-63
ENSG0000(	ATG4C	1	protein_coding	-412
ENSG0000(	GPR108	1	protein_coding	901
ENSG0000(	FOSB	1	protein_coding	-442
ENSG0000(	SNRPD2	1	protein_coding	-37
ENSG0000(	SNRPD2	1	protein_coding	-449
ENSG0000(	RTN2	1	protein_coding	847
ENSG0000(	VASP	1	protein_coding	790
ENSG0000(	SYMPK	1	protein_coding	-18
ENSG0000(	SYMPK	1	protein_coding	618
ENSG0000(	SNRPB	1	protein_coding	179
ENSG0000(	AP5S1	1	protein_coding	-583
ENSG0000(	AP5S1	1	protein_coding	81
ENSG0000(	ZNF133	1	protein_coding	69
ENSG0000(	SNRPB2	1	protein_coding	21
ENSG0000(	MGME1	1	protein_coding	-20
ENSG0000(	MGME1	1	protein_coding	-187
ENSG0000(	ITPA	1	protein_coding	346
ENSG0000(	NCLN	1	protein_coding	212
ENSG0000(	HNRNPR	1	protein_coding	-711
ENSG0000(	ID1	1	protein_coding	-992
ENSG0000(	DYNLRB1	1	protein_coding	-28
ENSG0000(	MMP24-AS1	1	protein_coding	843
ENSG0000(	TMEM115	1	protein_coding	-12
ENSG0000(	PSMB2	1	protein_coding	257
ENSG0000(	UROD	1	protein_coding	-583
ENSG0000(	UROD	1	protein_coding	480
ENSG0000(	HECTD3	1	protein_coding	-234
ENSG0000(	PCID2	1	protein_coding	-1
ENSG0000(	PCID2	1	protein_coding	586
ENSG0000(	PDCD2L	1	protein_coding	131
ENSG0000(	RBM42	1	protein_coding	-69
ENSG0000(	COX6B1	1	protein_coding	81
ENSG0000(	FRMD8	1	protein_coding	-255
ENSG0000(	PRDX5	1	protein_coding	-259
ENSG0000(	PRDX5	1	protein_coding	234
ENSG0000(	BCL2L12	1	protein_coding	-49
ENSG0000(	IRF3	1	protein_coding	783
ENSG0000(	WNK4	1	protein_coding	-843
ENSG0000(	BECN1	1	protein_coding	-42

ENSG0000(	GLIS2	1	protein_coding	-433
ENSG0000(	GLIS2	1	protein_coding	-665
ENSG0000(	IFI6	1	protein_coding	175
ENSG0000(	ATG14	1	protein_coding	-214
ENSG0000(	RHOT1	1	protein_coding	-342
ENSG0000(	WDR60	1	protein_coding	-75
ENSG0000(	AIF1L	1	protein_coding	-441
ENSG0000(	NUP214	1	protein_coding	171
ENSG0000(	AVPR2	1	protein_coding	-935
ENSG0000(	MAP2K2	1	protein_coding	-172
ENSG0000(	HNRNPH2	1	protein_coding	-138
ENSG0000(	CANX	1	protein_coding	-64
ENSG0000(	CANX	1	protein_coding	99
ENSG0000(	ABHD8	1	protein_coding	252
ENSG0000(	ABHD8	1	protein_coding	954
ENSG0000(	ATP13A4	1	protein_coding	-689
ENSG0000(	ATP13A4	1	protein_coding	133
ENSG0000(	HELB	1	protein_coding	157
ENSG0000(	RAP1B	1	protein_coding	-621
ENSG0000(	LRRC61	1	protein_coding	-133
ENSG0000(	LRRC61	1	protein_coding	-616
ENSG0000(	TMEM175	1	protein_coding	463
ENSG0000(	AUNIP	1	protein_coding	331
ENSG0000(	PIN1	1	protein_coding	-425
ENSG0000(	PIN1	1	protein_coding	601
ENSG0000(	FBXL12	1	protein_coding	-14
ENSG0000(	EMC1	1	protein_coding	-117
ENSG0000(	UBR4	1	protein_coding	-249
ENSG0000(	HP1BP3	1	protein_coding	-520
ENSG0000(	SLC35E1	1	protein_coding	-538
ENSG0000(	F2RL3	1	protein_coding	-52
ENSG0000(	PKMYT1	1	protein_coding	-140
ENSG0000(	WDR24	1	protein_coding	-39
ENSG0000(	CHTF18	1	protein_coding	703
ENSG0000(	KDM4B	1	protein_coding	-622
ENSG0000(	TICAM1	1	protein_coding	781
ENSG0000(	METTL25	1	protein_coding	42
ENSG0000(	TUBA4A	1	protein_coding	322
ENSG0000(	PTPN12	1	protein_coding	-610
ENSG0000(	PEX1	1	protein_coding	-381
ENSG0000(	MTERF1	1	protein_coding	24
ENSG0000(	RBM48	1	protein_coding	140
ENSG0000(	SRD5A3	1	protein_coding	46
ENSG0000(	DGCR6L	1	protein_coding	-81
ENSG0000(	DGCR6L	1	protein_coding	-491
ENSG0000(	DGCR8	1	protein_coding	-255
ENSG0000(	SDF2L1	1	protein_coding	360
ENSG0000(	ATF4	1	protein_coding	594
ENSG0000(	MPST	1	protein_coding	600
ENSG0000(	TST	1	protein_coding	-51
ENSG0000(	RAC2	1	protein_coding	227
ENSG0000(	C22orf23	1	protein_coding	362
ENSG0000(	DOCK4	1	protein_coding	-161
ENSG0000(	ATP6V1F	1	protein_coding	-228

ENSG0000(	DNAJB9	1	protein_coding	271
ENSG0000(	CALU	1	protein_coding	-289
ENSG0000(	KLHDC10	1	protein_coding	-263
ENSG0000(	NDUFA5	1	protein_coding	140
ENSG0000(	NDUFA5	1	protein_coding	777
ENSG0000(	MTX2	1	protein_coding	595
ENSG0000(	OSGEPL1	1	protein_coding	65
ENSG0000(	HAT1	1	protein_coding	405
ENSG0000(	TWSG1	1	protein_coding	-611
ENSG0000(	INO80	1	protein_coding	-382
ENSG0000(	ICE2	1	protein_coding	731
ENSG0000(	FAM63B	1	protein_coding	-345
ENSG0000(	ARPP19	1	protein_coding	575
ENSG0000(	MBD4	1	protein_coding	-211
ENSG0000(	SUMF2	1	protein_coding	197
ENSG0000(	RPAIN	1	protein_coding	533
ENSG0000(	MPDU1	1	protein_coding	180
ENSG0000(	PHF20L1	1	protein_coding	-215
ENSG0000(	CCNT1	1	protein_coding	474
ENSG0000(	PUS7L	1	protein_coding	180
ENSG0000(	ILF3	1	protein_coding	-265
ENSG0000(	CDKN2D	1	protein_coding	-467
ENSG0000(	REEP5	1	protein_coding	390
ENSG0000(	FOXJ1	1	protein_coding	-42
ENSG0000(	RHBDF2	1	protein_coding	-54
ENSG0000(	ASH2L	1	protein_coding	395
ENSG0000(	SGO1	1	protein_coding	359
ENSG0000(	MAU2	1	protein_coding	608
ENSG0000(	CBFA2T3	1	protein_coding	-134
ENSG0000(	CBFA2T3	1	protein_coding	-996
ENSG0000(	CBFA2T3	1	protein_coding	425
ENSG0000(	ERMARD	1	protein_coding	50
ENSG0000(	PHF10	1	protein_coding	343
ENSG0000(	ECSIT	1	protein_coding	-110
ENSG0000(	PRKCSH	1	protein_coding	-108
ENSG0000(	ZSCAN10	1	protein_coding	-684
ENSG0000(	NECTIN2	1	protein_coding	-434
ENSG0000(	APOC1	1	protein_coding	894
ENSG0000(	FAM98C	1	protein_coding	81
ENSG0000(	GTPBP3	1	protein_coding	-62
ENSG0000(	GTPBP3	1	protein_coding	-662
ENSG0000(	BST2	1	protein_coding	335
ENSG0000(	SLC27A1	1	protein_coding	57
ENSG0000(	COLGALT1	1	protein_coding	604
ENSG0000(	DDA1	1	protein_coding	-235
ENSG0000(	DDA1	1	protein_coding	467
ENSG0000(	MRPL34	1	protein_coding	264
ENSG0000(	PGLS	1	protein_coding	456
ENSG0000(	LSM7	1	protein_coding	802
ENSG0000(	TULP4	1	protein_coding	773
ENSG0000(	C6orf203	1	protein_coding	105
ENSG0000(	C6orf203	1	protein_coding	496
ENSG0000(	RSPH3	1	protein_coding	224
ENSG0000(	RSPH3	1	protein_coding	996

ENSG0000(	MLLT1	1	protein_coding	980
ENSG0000(	NDUFA10	1	protein_coding	-46
ENSG0000(	UNC13A	1	protein_coding	21
ENSG0000(	SSBP4	1	protein_coding	-36
ENSG0000(	GDF15	1	protein_coding	-557
ENSG0000(	GDF15	1	protein_coding	784
ENSG0000(	LSM4	1	protein_coding	703
ENSG0000(	JUND	1	protein_coding	-818
ENSG0000(	HRC	1	protein_coding	-678
ENSG0000(	HRC	1	protein_coding	574
ENSG0000(	SAMD10	1	protein_coding	-510
ENSG0000(	HBZ	1	protein_coding	-360
ENSG0000(	MNX1	1	protein_coding	-775
ENSG0000(	CEP85	1	protein_coding	-263
ENSG0000(	CEP85	1	protein_coding	659
ENSG0000(	RBBP8NL	1	protein_coding	752
ENSG0000(	EXOSC2	1	protein_coding	312
ENSG0000(	CHMP2A	1	protein_coding	-135
ENSG0000(	TRIM28	1	protein_coding	-181
ENSG0000(	C16orf13	1	protein_coding	-182
ENSG0000(	C16orf13	1	protein_coding	133
ENSG0000(	C16orf13	1	protein_coding	721
ENSG0000(	ZC3H4	1	protein_coding	20
ENSG0000(	NPAS1	1	protein_coding	-324
ENSG0000(	MAP3K10	1	protein_coding	-611
ENSG0000(	LRRC47	1	protein_coding	-28
ENSG0000(	LRRC47	1	protein_coding	-479
ENSG0000(	SESN2	1	protein_coding	-532
ENSG0000(	SMPDL3B	1	protein_coding	47
ENSG0000(	ATPIF1	1	protein_coding	107
ENSG0000(	ATPIF1	1	protein_coding	832
ENSG0000(	MED18	1	protein_coding	-15
ENSG0000(	ZNF317	1	protein_coding	-62
ENSG0000(	ZNF317	1	protein_coding	260
ENSG0000(	PPAN	1	protein_coding	-242
ENSG0000(	DNMT1	1	protein_coding	-855
ENSG0000(	DKC1	1	protein_coding	766
ENSG0000(	NOL11	1	protein_coding	653
ENSG0000(	PPIL4	1	protein_coding	-301
ENSG0000(	PPIL4	1	protein_coding	611
ENSG0000(	LATS1	1	protein_coding	-186
ENSG0000(	ZNF428	1	protein_coding	-172
ENSG0000(	ZNF428	1	protein_coding	591
ENSG0000(	CHMP1A	1	protein_coding	-612
ENSG0000(	CHMP1A	1	protein_coding	18
ENSG0000(	SLC34A1	1	protein_coding	52
ENSG0000(	PRR7	1	protein_coding	-300
ENSG0000(	PRR7	1	protein_coding	60
ENSG0000(	CAP1	1	protein_coding	-326
ENSG0000(	HAUS8	1	protein_coding	676
ENSG0000(	HACL1	1	protein_coding	191
ENSG0000(	CAPN7	1	protein_coding	-264
ENSG0000(	CAPN7	1	protein_coding	259
ENSG0000(	RBSN	1	protein_coding	19

ENSG0000(	SLC6A6	1	protein_coding	-419
ENSG0000(	SLC6A6	1	protein_coding	729
ENSG0000(	KCNC3	1	protein_coding	-207
ENSG0000(	KCNC3	1	protein_coding	-710
ENSG0000(	KCNC3	1	protein_coding	832
ENSG0000(	NR1H2	1	protein_coding	91
ENSG0000(	PDLIM4	1	protein_coding	910
ENSG0000(	TUBG1	1	protein_coding	-583
ENSG0000(	RPL27	1	protein_coding	328
ENSG0000(	NDUFA2	1	protein_coding	-64
ENSG0000(	ANKHD1	1	protein_coding	-431
ENSG0000(	ANKHD1	1	protein_coding	394
ENSG0000(	ANKHD1	1	protein_coding	648
ENSG0000(	EXOC4	1	protein_coding	574
ENSG0000(	ACAP3	1	protein_coding	-242
ENSG0000(	ACAP3	1	protein_coding	-681
ENSG0000(	PPFIA1	1	protein_coding	-308
ENSG0000(	TMEM204	1	protein_coding	-115
ENSG0000(	TMEM204	1	protein_coding	-499
ENSG0000(	KREMEN2	1	protein_coding	-27
ENSG0000(	TRAF7	1	protein_coding	-519
ENSG0000(	PEX11B	1	protein_coding	-523
ENSG0000(	PIAS3	1	protein_coding	8
ENSG0000(	NR0B2	1	protein_coding	-307
ENSG0000(	THAP1	1	protein_coding	5
ENSG0000(	FAAP24	1	protein_coding	-214
ENSG0000(	ACTR10	1	protein_coding	-193
ENSG0000(	RFX1	1	protein_coding	-296
ENSG0000(	RFX1	1	protein_coding	330
ENSG0000(	C19orf57	1	protein_coding	487
ENSG0000(	CC2D1A	1	protein_coding	-235
ENSG0000(	RAF1	1	protein_coding	-202
ENSG0000(	NUP210	1	protein_coding	-866
ENSG0000(	NUP210	1	protein_coding	409
ENSG0000(	ENOSF1	1	protein_coding	943
ENSG0000(	CNGA4	1	protein_coding	576
ENSG0000(	RRP8	1	protein_coding	-524
ENSG0000(	PTCD3	1	protein_coding	361
ENSG0000(	RAN	1	protein_coding	-962
ENSG0000(	TBC1D14	1	protein_coding	-522
ENSG0000(	COQ3	1	protein_coding	-25
ENSG0000(	PNISR	1	protein_coding	-109
ENSG0000(	SEC61G	1	protein_coding	631
ENSG0000(	UTP3	1	protein_coding	-135
ENSG0000(	H3F3B	1	protein_coding	-450
ENSG0000(	H3F3B	1	protein_coding	313
ENSG0000(	UNK	1	protein_coding	-327
ENSG0000(	UNK	1	protein_coding	810
ENSG0000(	EIF5A	1	protein_coding	160
ENSG0000(	GPS2	1	protein_coding	770
ENSG0000(	DLG4	1	protein_coding	-18
ENSG0000(	RIDA	1	protein_coding	-5
ENSG0000(	PCBD2	1	protein_coding	-323
ENSG0000(	ERAL1	1	protein_coding	-161

ENSG0000(	ERAL1	1	protein_coding	234
ENSG0000(	NIP7	1	protein_coding	162
ENSG0000(	NIP7	1	protein_coding	585
ENSG0000(	MTSS1L	1	protein_coding	-755
ENSG0000(	PCED1A	1	protein_coding	752
ENSG0000(	PCNA	1	protein_coding	197
ENSG0000(	NXT1	1	protein_coding	-284
ENSG0000(	KIAA0907	1	protein_coding	-93
ENSG0000(	NES	1	protein_coding	-854
ENSG0000(	IGHMBP2	1	protein_coding	-23
ENSG0000(	MMACHC	1	protein_coding	-72
ENSG0000(	DPH2	1	protein_coding	148
ENSG0000(	NASP	1	protein_coding	-64
ENSG0000(	MUTYH	1	protein_coding	-212
ENSG0000(	SERINC3	1	protein_coding	-201
ENSG0000(	PPP1R3D	1	protein_coding	-322
ENSG0000(	RSG1	1	protein_coding	-125
ENSG0000(	CASP9	1	protein_coding	-208
ENSG0000(	DCTN4	1	protein_coding	132
ENSG0000(	XPO4	1	protein_coding	130
ENSG0000(	PIK3C2B	1	protein_coding	454
ENSG0000(	DSTYK	1	protein_coding	-328
ENSG0000(	CHIT1	1	protein_coding	348
ENSG0000(	GPALPP1	1	protein_coding	16
ENSG0000(	KMT5C	1	protein_coding	-311
ENSG0000(	HSPBP1	1	protein_coding	-621
ENSG0000(	MACROD1	1	protein_coding	924
ENSG0000(	LGALS12	1	protein_coding	-36
ENSG0000(	FOPNL	1	protein_coding	-598
ENSG0000(	SEC14L4	1	protein_coding	-939
ENSG0000(	ADCK2	1	protein_coding	-374
ENSG0000(	ADCK2	1	protein_coding	319
ENSG0000(	AGAP3	1	protein_coding	-246
ENSG0000(	AGAP3	1	protein_coding	-665
ENSG0000(	BTG1	1	protein_coding	92
ENSG0000(	LARS	1	protein_coding	-93
ENSG0000(	IMPA1	1	protein_coding	672
ENSG0000(	CCDC59	1	protein_coding	267
ENSG0000(	SARAF	1	protein_coding	224
ENSG0000(	SARAF	1	protein_coding	554
ENSG0000(	CCNB1	1	protein_coding	-210
ENSG0000(	CCNB1	1	protein_coding	156
ENSG0000(	IRAK2	1	protein_coding	45
ENSG0000(	THUMPD3	1	protein_coding	12
ENSG0000(	THUMPD3	1	protein_coding	553
ENSG0000(	MEIS2	1	protein_coding	31
ENSG0000(	DPH6	1	protein_coding	-93
ENSG0000(	EMC7	1	protein_coding	-46
ENSG0000(	GNAT2	1	protein_coding	-397
ENSG0000(	GNAT2	1	protein_coding	478
ENSG0000(	PRPF38B	1	protein_coding	-233
ENSG0000(	PSRC1	1	protein_coding	-582
ENSG0000(	PSRC1	1	protein_coding	386
ENSG0000(	WNT2B	1	protein_coding	-391

ENSG0000(	LAMTOR5	1	protein_coding	187
ENSG0000(	AP4B1	1	protein_coding	-144
ENSG0000(	AP4B1	1	protein_coding	481
ENSG0000(	RSAD2	1	protein_coding	581
ENSG0000(	CMPK2	1	protein_coding	249
ENSG0000(	LDHA	1	protein_coding	211
ENSG0000(	CDC73	1	protein_coding	71
ENSG0000(	TIMM17A	1	protein_coding	95
ENSG0000(	DDB2	1	protein_coding	881
ENSG0000(	ACP2	1	protein_coding	-163
ENSG0000(	STK26	1	protein_coding	661
ENSG0000(	PUM1	1	protein_coding	-102
ENSG0000(	PUM1	1	protein_coding	333
ENSG0000(	SPOCD1	1	protein_coding	-28
ENSG0000(	PHC2	1	protein_coding	775
ENSG0000(	CDCA8	1	protein_coding	-150
ENSG0000(	AGO4	1	protein_coding	915
ENSG0000(	BTF3L4	1	protein_coding	-625
ENSG0000(	BTF3L4	1	protein_coding	799
ENSG0000(	PRPF38A	1	protein_coding	-573
ENSG0000(	DSC2	1	protein_coding	128
ENSG0000(	ELP2	1	protein_coding	386
ENSG0000(	TPGS2	1	protein_coding	-248
ENSG0000(	TIMM10	1	protein_coding	298
ENSG0000(	DHX34	1	protein_coding	-91
ENSG0000(	FADS2	1	protein_coding	-266
ENSG0000(	TMEM258	1	protein_coding	89
ENSG0000(	TMEM165	1	protein_coding	895
ENSG0000(	GGACT	1	protein_coding	651
ENSG0000(	UBAC2	1	protein_coding	-129
ENSG0000(	CARS2	1	protein_coding	358
ENSG0000(	APC	1	protein_coding	75
ENSG0000(	WDR36	1	protein_coding	571
ENSG0000(	UBQLN1	1	protein_coding	-123
ENSG0000(	C9orf40	1	protein_coding	271
ENSG0000(	CTSL	1	protein_coding	979
ENSG0000(	AGTPBP1	1	protein_coding	752
ENSG0000(	PSAT1	1	protein_coding	237
ENSG0000(	HAVCR2	1	protein_coding	58
ENSG0000(	TMEM60	1	protein_coding	218
ENSG0000(	PNPLA8	1	protein_coding	-172
ENSG0000(	HILPDA	1	protein_coding	-324
ENSG0000(	RINT1	1	protein_coding	114
ENSG0000(	RINT1	1	protein_coding	479
ENSG0000(	TES	1	protein_coding	676
ENSG0000(	MTO1	1	protein_coding	263
ENSG0000(	ORC3	1	protein_coding	50
ENSG0000(	LMO2	1	protein_coding	232
ENSG0000(	DNAJC14	1	protein_coding	997
ENSG0000(	COQ10A	1	protein_coding	16
ENSG0000(	PAN2	1	protein_coding	223
ENSG0000(	PAN2	1	protein_coding	862
ENSG0000(	HNRNPA1	1	protein_coding	322
ENSG0000(	HNRNPA1	1	protein_coding	917

ENSG0000(	MIP	1	protein_coding	275
ENSG0000(	LTV1	1	protein_coding	49
ENSG0000(	CD164	1	protein_coding	-690
ENSG0000(	LACE1	1	protein_coding	217
ENSG0000(	AHI1	1	protein_coding	-927
ENSG0000(	AHI1	1	protein_coding	181
ENSG0000(	TEC	1	protein_coding	-654
ENSG0000(	CCDC142	1	protein_coding	34
ENSG0000(	CCDC142	1	protein_coding	688
ENSG0000(	MDM2	1	protein_coding	375
ENSG0000(	DYNC1LI2	1	protein_coding	-32
ENSG0000(	FBXL8	1	protein_coding	251
ENSG0000(	FHOD1	1	protein_coding	-350
ENSG0000(	ZNF670-ZNF695	1	protein_coding	8
ENSG0000(	COG2	1	protein_coding	-447
ENSG0000(	COG2	1	protein_coding	153
ENSG0000(	ABCB10	1	protein_coding	-603
ENSG0000(	GLUL	1	protein_coding	-419
ENSG0000(	DHX9	1	protein_coding	-347
ENSG0000(	CEP350	1	protein_coding	-238
ENSG0000(	NPL	1	protein_coding	791
ENSG0000(	PIGC	1	protein_coding	-20
ENSG0000(	CAB39	1	protein_coding	-325
ENSG0000(	REV1	1	protein_coding	-535
ENSG0000(	TSGA10	1	protein_coding	-23
ENSG0000(	TSGA10	1	protein_coding	-373
ENSG0000(	TMEM127	1	protein_coding	-381
ENSG0000(	TMEM127	1	protein_coding	-692
ENSG0000(	GCC2	1	protein_coding	165
ENSG0000(	C2orf49	1	protein_coding	-115
ENSG0000(	PWP1	1	protein_coding	118
ENSG0000(	KIAA1033	1	protein_coding	522
ENSG0000(	VILL	1	protein_coding	-699
ENSG0000(	MED4	1	protein_coding	832
ENSG0000(	SETDB2	1	protein_coding	-320
ENSG0000(	CHST12	1	protein_coding	-354
ENSG0000(	IL6	1	protein_coding	730
ENSG0000(	TBRG4	1	protein_coding	221
ENSG0000(	TBRG4	1	protein_coding	665
ENSG0000(	NACAD	1	protein_coding	-736
ENSG0000(	RSAD1	1	protein_coding	460
ENSG0000(	NMT1	1	protein_coding	-361
ENSG0000(	SRSF1	1	protein_coding	-15
ENSG0000(	TACO1	1	protein_coding	-75
ENSG0000(	DCAF7	1	protein_coding	-458
ENSG0000(	NDUFB5	1	protein_coding	-101
ENSG0000(	MRPL47	1	protein_coding	66
ENSG0000(	TRA2B	1	protein_coding	-175
ENSG0000(	TRA2B	1	protein_coding	158
ENSG0000(	TRA2B	1	protein_coding	921
ENSG0000(	7-Mar	1	protein_coding	231
ENSG0000(	GALNT5	1	protein_coding	99
ENSG0000(	EPRS	1	protein_coding	-92
ENSG0000(	EPRS	1	protein_coding	638

ENSG0000(	VPS45	1	protein_coding	178
ENSG0000(	KCTD3	1	protein_coding	890
ENSG0000(	RPS6KC1	1	protein_coding	-353
ENSG0000(	WDR33	1	protein_coding	-108
ENSG0000(	CCDC115	1	protein_coding	-326
ENSG0000(	SAP130	1	protein_coding	-304
ENSG0000(	IMP4	1	protein_coding	451
ENSG0000(	YME1L1	1	protein_coding	-318
ENSG0000(	CDK9	1	protein_coding	-684
ENSG0000(	CDK9	1	protein_coding	857
ENSG0000(	TXN	1	protein_coding	-645
ENSG0000(	ODF2	1	protein_coding	498
ENSG0000(	ODF2	1	protein_coding	994
ENSG0000(	SMC2	1	protein_coding	-95
ENSG0000(	SLC2A8	1	protein_coding	-479
ENSG0000(	SLC2A8	1	protein_coding	-841
ENSG0000(	CDK5RAP2	1	protein_coding	-360
ENSG0000(	PRPF4	1	protein_coding	146
ENSG0000(	ATP6V1G1	1	protein_coding	22
ENSG0000(	WDR38	1	protein_coding	-431
ENSG0000(	TSTD2	1	protein_coding	630
ENSG0000(	PSMB7	1	protein_coding	-52
ENSG0000(	TRMO	1	protein_coding	295
ENSG0000(	RABEPK	1	protein_coding	-944
ENSG0000(	RABEPK	1	protein_coding	484
ENSG0000(	XPA	1	protein_coding	601
ENSG0000(	NCBP1	1	protein_coding	-680
ENSG0000(	DSCC1	1	protein_coding	294
ENSG0000(	DERL1	1	protein_coding	433
ENSG0000(	MYC	1	protein_coding	906
ENSG0000(	POLR1E	1	protein_coding	321
ENSG0000(	PLAA	1	protein_coding	97
ENSG0000(	RNF38	1	protein_coding	846
ENSG0000(	DNAJB5	1	protein_coding	-299
ENSG0000(	GRHPR	1	protein_coding	-76
ENSG0000(	GRHPR	1	protein_coding	-673
ENSG0000(	CNPY3	1	protein_coding	-28
ENSG0000(	FOXP4	1	protein_coding	-253
ENSG0000(	PPIL1	1	protein_coding	-816
ENSG0000(	KIF13A	1	protein_coding	-980
ENSG0000(	GMPR	1	protein_coding	-978
ENSG0000(	GMPR	1	protein_coding	582
ENSG0000(	GMPR	1	protein_coding	993
ENSG0000(	YIPF3	1	protein_coding	-103
ENSG0000(	YIPF3	1	protein_coding	416
ENSG0000(	FRS3	1	protein_coding	-597
ENSG0000(	BPHL	1	protein_coding	-281
ENSG0000(	BPHL	1	protein_coding	340
ENSG0000(	UQCC2	1	protein_coding	-430
ENSG0000(	TCF19	1	protein_coding	57
ENSG0000(	TCF19	1	protein_coding	663
ENSG0000(	FLOT1	1	protein_coding	-577
ENSG0000(	FLOT1	1	protein_coding	604
ENSG0000(	MDC1	1	protein_coding	636

ENSG0000(	NRM	1	protein_coding	887
ENSG0000(	VARS2	1	protein_coding	-135
ENSG0000(	TAF8	1	protein_coding	419
ENSG0000(	FAM8A1	1	protein_coding	999
ENSG0000(	C6orf52	1	protein_coding	-115
ENSG0000(	MYO7A	1	protein_coding	-420
ENSG0000(	ARRB1	1	protein_coding	-482
ENSG0000(	ARRB1	1	protein_coding	601
ENSG0000(	ARRB1	1	protein_coding	999
ENSG0000(	RAB30	1	protein_coding	-83
ENSG0000(	RAB30	1	protein_coding	-489
ENSG0000(	RAB30	1	protein_coding	164
ENSG0000(	LRRC32	1	protein_coding	-625
ENSG0000(	NARS2	1	protein_coding	-371
ENSG0000(	RNF121	1	protein_coding	-578
ENSG0000(	MRPL15	1	protein_coding	-553
ENSG0000(	GGH	1	protein_coding	-295
ENSG0000(	TGS1	1	protein_coding	-560
ENSG0000(	DCUN1D5	1	protein_coding	-167
ENSG0000(	SLC37A4	1	protein_coding	0
ENSG0000(	SLC37A4	1	protein_coding	-197
ENSG0000(	C11orf1	1	protein_coding	165
ENSG0000(	C11orf1	1	protein_coding	727
ENSG0000(	CTDSPL2	1	protein_coding	92
ENSG0000(	CTDSPL2	1	protein_coding	605
ENSG0000(	SLTM	1	protein_coding	-315
ENSG0000(	MAPKBP1	1	protein_coding	143
ENSG0000(	NUSAP1	1	protein_coding	-550
ENSG0000(	NDUFAF1	1	protein_coding	-25
ENSG0000(	HAUS2	1	protein_coding	67
ENSG0000(	TUBGCP4	1	protein_coding	951
ENSG0000(	ITPKA	1	protein_coding	120
ENSG0000(	ZNF280D	1	protein_coding	291
ENSG0000(	ZNF280D	1	protein_coding	578
ENSG0000(	RSL24D1	1	protein_coding	140
ENSG0000(	GCHFR	1	protein_coding	117
ENSG0000(	GTF2B	1	protein_coding	430
ENSG0000(	GIPC2	1	protein_coding	-48
ENSG0000(	DBT	1	protein_coding	238
ENSG0000(	IFT172	1	protein_coding	1
ENSG0000(	KHK	1	protein_coding	-966
ENSG0000(	PNPT1	1	protein_coding	201
ENSG0000(	SULT6B1	1	protein_coding	-360
ENSG0000(	SULT6B1	1	protein_coding	204
ENSG0000(	RAB1A	1	protein_coding	-729
ENSG0000(	PREB	1	protein_coding	658
ENSG0000(	CENPO	1	protein_coding	-327
ENSG0000(	CENPO	1	protein_coding	174
ENSG0000(	FAM149B1	1	protein_coding	569
ENSG0000(	ASCC1	1	protein_coding	230
ENSG0000(	ADAMTS14	1	protein_coding	-898
ENSG0000(	ADAMTS14	1	protein_coding	925
ENSG0000(	TET1	1	protein_coding	-425
ENSG0000(	DNA2	1	protein_coding	40

ENSG0000(	SMARCAL1	1	protein_coding	28
ENSG0000(	METTL5	1	protein_coding	-117
ENSG0000(	PPIG	1	protein_coding	11
ENSG0000(	FASTKD1	1	protein_coding	356
ENSG0000(	OLA1	1	protein_coding	-496
ENSG0000(	OLA1	1	protein_coding	922
ENSG0000(	CIR1	1	protein_coding	-472
ENSG0000(	CIR1	1	protein_coding	150
ENSG0000(	ITGAV	1	protein_coding	935
ENSG0000(	DIRC2	1	protein_coding	-865
ENSG0000(	USP8	1	protein_coding	438
ENSG0000(	TMOD3	1	protein_coding	-278
ENSG0000(	CILP	1	protein_coding	-778
ENSG0000(	SEMA7A	1	protein_coding	-595
ENSG0000(	SEMA7A	1	protein_coding	212
ENSG0000(	UBL7	1	protein_coding	-310
ENSG0000(	HERC6	1	protein_coding	545
ENSG0000(	COPS4	1	protein_coding	366
ENSG0000(	SEC31A	1	protein_coding	434
ENSG0000(	FGF5	1	protein_coding	278
ENSG0000(	NUP54	1	protein_coding	30
ENSG0000(	BMP2K	1	protein_coding	-675
ENSG0000(	G3BP2	1	protein_coding	-412
ENSG0000(	USO1	1	protein_coding	345
ENSG0000(	CASP6	1	protein_coding	444
ENSG0000(	SEC24B	1	protein_coding	-168
ENSG0000(	YARS2	1	protein_coding	697
ENSG0000(	ETNK1	1	protein_coding	-227
ENSG0000(	ETNK1	1	protein_coding	676
ENSG0000(	TMEM19	1	protein_coding	109
ENSG0000(	SNRPF	1	protein_coding	-26
ENSG0000(	TDG	1	protein_coding	-242
ENSG0000(	SDSL	1	protein_coding	27
ENSG0000(	NABP2	1	protein_coding	389
ENSG0000(	BRCA2	1	protein_coding	46
ENSG0000(	LMBR1L	1	protein_coding	-631
ENSG0000(	SBNO1	1	protein_coding	-615
ENSG0000(	VPS33A	1	protein_coding	75
ENSG0000(	RHOF	1	protein_coding	462
ENSG0000(	CUL4A	1	protein_coding	-63
ENSG0000(	CUL4A	1	protein_coding	524
ENSG0000(	CDH24	1	protein_coding	-570
ENSG0000(	WDR89	1	protein_coding	304
ENSG0000(	EFCAB11	1	protein_coding	180
ENSG0000(	MFAP1	1	protein_coding	-79
ENSG0000(	TCF12	1	protein_coding	-342
ENSG0000(	TCF12	1	protein_coding	-629
ENSG0000(	SORD	1	protein_coding	419
ENSG0000(	ZSCAN29	1	protein_coding	854
ENSG0000(	BNIP2	1	protein_coding	-93
ENSG0000(	BAHD1	1	protein_coding	41
ENSG0000(	BAHD1	1	protein_coding	718
ENSG0000(	MAN2C1	1	protein_coding	137
ENSG0000(	MAN2C1	1	protein_coding	483

ENSG0000(	BBS4	1	protein_coding	531
ENSG0000(	PML	1	protein_coding	793
ENSG0000(	CCDC33	1	protein_coding	-692
ENSG0000(	CCDC33	1	protein_coding	195
ENSG0000(	FANCI	1	protein_coding	-282
ENSG0000(	ABHD2	1	protein_coding	318
ENSG0000(	FURIN	1	protein_coding	-5
ENSG0000(	FURIN	1	protein_coding	705
ENSG0000(	12-Sep	1	protein_coding	-315
ENSG0000(	C16orf58	1	protein_coding	533
ENSG0000(	PARN	1	protein_coding	283
ENSG0000(	GCSH	1	protein_coding	-353
ENSG0000(	GCSH	1	protein_coding	485
ENSG0000(	MBTPS1	1	protein_coding	-43
ENSG0000(	MBTPS1	1	protein_coding	-760
ENSG0000(	RHOT2	1	protein_coding	-83
ENSG0000(	ZSCAN32	1	protein_coding	34
ENSG0000(	RPS2	1	protein_coding	-247
ENSG0000(	RPS2	1	protein_coding	776
ENSG0000(	TCF25	1	protein_coding	-400
ENSG0000(	TCF25	1	protein_coding	-979
ENSG0000(	NCOR1	1	protein_coding	992
ENSG0000(	RANBP10	1	protein_coding	-570
ENSG0000(	PCTP	1	protein_coding	259
ENSG0000(	G6PC3	1	protein_coding	400
ENSG0000(	CLTC	1	protein_coding	-552
ENSG0000(	C17orf64	1	protein_coding	859
ENSG0000(	BCAS3	1	protein_coding	158
ENSG0000(	PTRH2	1	protein_coding	227
ENSG0000(	AFG3L2	1	protein_coding	-623
ENSG0000(	IMPA2	1	protein_coding	-699
ENSG0000(	SLC39A6	1	protein_coding	-444
ENSG0000(	C18orf8	1	protein_coding	-238
ENSG0000(	C18orf8	1	protein_coding	975
ENSG0000(	MINK1	1	protein_coding	997
ENSG0000(	CCDC40	1	protein_coding	-84
ENSG0000(	ARHGDIA	1	protein_coding	-192
ENSG0000(	ARHGDIA	1	protein_coding	-734
ENSG0000(	ANAPC11	1	protein_coding	-250
ENSG0000(	TBCD	1	protein_coding	60
ENSG0000(	FN3KRP	1	protein_coding	551
ENSG0000(	NARF	1	protein_coding	45
ENSG0000(	NARF	1	protein_coding	597
ENSG0000(	RPTOR	1	protein_coding	119
ENSG0000(	FOXK2	1	protein_coding	-520
ENSG0000(	TRIM65	1	protein_coding	443
ENSG0000(	CBX4	1	protein_coding	-537
ENSG0000(	PNMT	1	protein_coding	667
ENSG0000(	SLC39A3	1	protein_coding	728
ENSG0000(	TPGS1	1	protein_coding	-222
ENSG0000(	PRDM15	1	protein_coding	982
ENSG0000(	FEM1A	1	protein_coding	-138
ENSG0000(	VAV1	1	protein_coding	147
ENSG0000(	MVB12A	1	protein_coding	-408

ENSG0000(	DUS3L	1	protein_coding	265
ENSG0000(	CCDC97	1	protein_coding	301
ENSG0000(	TMEM91	1	protein_coding	-994
ENSG0000(	IFITM3	1	protein_coding	81
ENSG0000(	IFITM3	1	protein_coding	430
ENSG0000(	IFNAR1	1	protein_coding	260
ENSG0000(	SOD1	1	protein_coding	33
ENSG0000(	TRPM2	1	protein_coding	-655
ENSG0000(	SCYL1	1	protein_coding	-459
ENSG0000(	SCYL1	1	protein_coding	786
ENSG0000(	DOPEY2	1	protein_coding	138
ENSG0000(	URB1	1	protein_coding	-490
ENSG0000(	AKT1	1	protein_coding	-471
ENSG0000(	AKT1	1	protein_coding	-676
ENSG0000(	SAE1	1	protein_coding	459
ENSG0000(	LMTK3	1	protein_coding	-467
ENSG0000(	GEMIN7	1	protein_coding	25
ENSG0000(	RNPEPL1	1	protein_coding	-84
ENSG0000(	CAPN10	1	protein_coding	-261
ENSG0000(	ZNF473	1	protein_coding	328
ENSG0000(	FAM71E1	1	protein_coding	180
ENSG0000(	PTH2	1	protein_coding	612
ENSG0000(	CTD-2545M3.6	1	protein_coding	-968
ENSG0000(	RPL13A	1	protein_coding	-903
ENSG0000(	RPL13A	1	protein_coding	62
ENSG0000(	CTU1	1	protein_coding	-80
ENSG0000(	CTU1	1	protein_coding	611
ENSG0000(	NOSIP	1	protein_coding	-140
ENSG0000(	PEX14	1	protein_coding	174
ENSG0000(	PGD	1	protein_coding	21
ENSG0000(	ZNF593	1	protein_coding	-324
ENSG0000(	C1orf216	1	protein_coding	267
ENSG0000(	KIAA0319L	1	protein_coding	454
ENSG0000(	PLK4	1	protein_coding	111
ENSG0000(	MAP3K6	1	protein_coding	-129
ENSG0000(	GPN2	1	protein_coding	571
ENSG0000(	SYTL1	1	protein_coding	-4
ENSG0000(	SYTL1	1	protein_coding	-402
ENSG0000(	SYTL1	1	protein_coding	945
ENSG0000(	WDTC1	1	protein_coding	-664
ENSG0000(	HSPG2	1	protein_coding	-560
ENSG0000(	HSPG2	1	protein_coding	890
ENSG0000(	SERBP1	1	protein_coding	-473
ENSG0000(	TINAGL1	1	protein_coding	-117
ENSG0000(	TINAGL1	1	protein_coding	281
ENSG0000(	RPS8	1	protein_coding	199
ENSG0000(	RPS8	1	protein_coding	965
ENSG0000(	KIF2C	1	protein_coding	-51
ENSG0000(	MOB3C	1	protein_coding	-119
ENSG0000(	MTF2	1	protein_coding	-137
ENSG0000(	IGSF3	1	protein_coding	-389
ENSG0000(	STRIP1	1	protein_coding	166
ENSG0000(	PSMA5	1	protein_coding	-154
ENSG0000(	CELSR2	1	protein_coding	-859

ENSG0000(	ITGA10	1	protein_coding	-56
ENSG0000(	ALDH9A1	1	protein_coding	547
ENSG0000(	ALDH9A1	1	protein_coding	898
ENSG0000(	NME7	1	protein_coding	120
ENSG0000(	POGK	1	protein_coding	-410
ENSG0000(	MPC2	1	protein_coding	-530
ENSG0000(	MPC2	1	protein_coding	665
ENSG0000(	DCAF6	1	protein_coding	-173
ENSG0000(	DCAF6	1	protein_coding	593
ENSG0000(	MGST3	1	protein_coding	286
ENSG0000(	ADCY10	1	protein_coding	27
ENSG0000(	RFWD2	1	protein_coding	-122
ENSG0000(	PPOX	1	protein_coding	-300
ENSG0000(	PPOX	1	protein_coding	825
ENSG0000(	FCGR2A	1	protein_coding	69
ENSG0000(	PFDN2	1	protein_coding	431
ENSG0000(	USP21	1	protein_coding	-134
ENSG0000(	PRCC	1	protein_coding	-913
ENSG0000(	RRNAD1	1	protein_coding	-142
ENSG0000(	MRPL24	1	protein_coding	317
ENSG0000(	ISG20L2	1	protein_coding	500
ENSG0000(	CRABP2	1	protein_coding	-249
ENSG0000(	HDGF	1	protein_coding	-437
ENSG0000(	TOR1AIP1	1	protein_coding	232
ENSG0000(	LYPLAL1	1	protein_coding	111
ENSG0000(	ECM1	1	protein_coding	-181
ENSG0000(	ZNF687	1	protein_coding	2
ENSG0000(	TARS2	1	protein_coding	-203
ENSG0000(	TARS2	1	protein_coding	139
ENSG0000(	SETDB1	1	protein_coding	-78
ENSG0000(	ANP32E	1	protein_coding	263
ENSG0000(	ANXA9	1	protein_coding	216
ENSG0000(	ENSA	1	protein_coding	356
ENSG0000(	ARNT	1	protein_coding	-614
ENSG0000(	GABPB2	1	protein_coding	-132
ENSG0000(	DTL	1	protein_coding	-388
ENSG0000(	DYRK3	1	protein_coding	-465
ENSG0000(	EIF2D	1	protein_coding	135
ENSG0000(	INTS7	1	protein_coding	378
ENSG0000(	TP53BP2	1	protein_coding	-518
ENSG0000(	TP53BP2	1	protein_coding	979
ENSG0000(	ATP8B2	1	protein_coding	-297
ENSG0000(	ATP8B2	1	protein_coding	853
ENSG0000(	ADAM15	1	protein_coding	-317
ENSG0000(	ADAM15	1	protein_coding	159
ENSG0000(	UBAP2L	1	protein_coding	195
ENSG0000(	HAX1	1	protein_coding	-97
ENSG0000(	EFNA3	1	protein_coding	-777
ENSG0000(	C1orf43	1	protein_coding	255
ENSG0000(	INTS3	1	protein_coding	-342
ENSG0000(	PKLR	1	protein_coding	111
ENSG0000(	PKLR	1	protein_coding	574
ENSG0000(	C1orf131	1	protein_coding	261
ENSG0000(	TTC13	1	protein_coding	-215

ENSG0000(	CEP170	1	protein_coding	-10
ENSG0000(	CEP170	1	protein_coding	-831
ENSG0000(	NVL	1	protein_coding	13
ENSG0000(	SDE2	1	protein_coding	-631
ENSG0000(	SDE2	1	protein_coding	384
ENSG0000(	GUK1	1	protein_coding	543
ENSG0000(	CDC42BPA	1	protein_coding	-758
ENSG0000(	CNIH3	1	protein_coding	534
ENSG0000(	MBOAT2	1	protein_coding	-881
ENSG0000(	PYCR2	1	protein_coding	805
ENSG0000(	PPFIA4	1	protein_coding	-447
ENSG0000(	PTPN7	1	protein_coding	-78
ENSG0000(	PTPN7	1	protein_coding	224
ENSG0000(	PTPN7	1	protein_coding	659
ENSG0000(	ARL8A	1	protein_coding	-588
ENSG0000(	EML4	1	protein_coding	-434
ENSG0000(	CALM2	1	protein_coding	-425
ENSG0000(	CALM2	1	protein_coding	180
ENSG0000(	RPS27A	1	protein_coding	473
ENSG0000(	CIAO1	1	protein_coding	244
ENSG0000(	CIAO1	1	protein_coding	555
ENSG0000(	ZNF514	1	protein_coding	-157
ENSG0000(	SNRNP200	1	protein_coding	-638
ENSG0000(	NT5DC4	1	protein_coding	-115
ENSG0000(	ZC3H8	1	protein_coding	45
ENSG0000(	LIPT1	1	protein_coding	33
ENSG0000(	LIPT1	1	protein_coding	383
ENSG0000(	UBXN4	1	protein_coding	-184
ENSG0000(	POLR2D	1	protein_coding	-383
ENSG0000(	SCRN3	1	protein_coding	-164
ENSG0000(	SCRN3	1	protein_coding	458
ENSG0000(	KIAA1715	1	protein_coding	342
ENSG0000(	PHOSPHO2	1	protein_coding	-69
ENSG0000(	PHOSPHO2	1	protein_coding	670
ENSG0000(	SUMF1	1	protein_coding	-64
ENSG0000(	HES6	1	protein_coding	360
ENSG0000(	COPS7B	1	protein_coding	-51
ENSG0000(	CPNE9	1	protein_coding	676
ENSG0000(	TAMM41	1	protein_coding	-11
ENSG0000(	CTDSP1	1	protein_coding	-6
ENSG0000(	GMPPA	1	protein_coding	-464
ENSG0000(	GMPPA	1	protein_coding	-795
ENSG0000(	EAF1	1	protein_coding	-322
ENSG0000(	CSRNP1	1	protein_coding	213
ENSG0000(	GOLGA4	1	protein_coding	86
ENSG0000(	GOLGA4	1	protein_coding	577
ENSG0000(	CTDSPL	1	protein_coding	791
ENSG0000(	RP11-977G19.10	1	protein_coding	-346
ENSG0000(	EIF2A	1	protein_coding	21
ENSG0000(	TMEM44	1	protein_coding	-129
ENSG0000(	RUBCN	1	protein_coding	2
ENSG0000(	RUBCN	1	protein_coding	298
ENSG0000(	TM4SF19	1	protein_coding	189
ENSG0000(	TM4SF19	1	protein_coding	900

ENSG0000(	EIF2B5	1	protein_coding	-802
ENSG0000(	EIF2B5	1	protein_coding	237
ENSG0000(	ECE2	1	protein_coding	722
ENSG0000(	FIP1L1	1	protein_coding	562
ENSG0000(	SLC26A1	1	protein_coding	-565
ENSG0000(	SLC26A1	1	protein_coding	494
ENSG0000(	CCNA2	1	protein_coding	702
ENSG0000(	METTL14	1	protein_coding	198
ENSG0000(	USP53	1	protein_coding	343
ENSG0000(	NAF1	1	protein_coding	-33
ENSG0000(	CBR4	1	protein_coding	20
ENSG0000(	ROPN1L	1	protein_coding	-240
ENSG0000(	6-Mar	1	protein_coding	-581
ENSG0000(	RPL37	1	protein_coding	-123
ENSG0000(	RPL37	1	protein_coding	287
ENSG0000(	SKP2	1	protein_coding	-975
ENSG0000(	SSBP2	1	protein_coding	696
ENSG0000(	IQGAP2	1	protein_coding	-274
ENSG0000(	SLC30A5	1	protein_coding	141
ENSG0000(	ATG12	1	protein_coding	179
ENSG0000(	YIPF5	1	protein_coding	95
ENSG0000(	DDX46	1	protein_coding	-339
ENSG0000(	DDX46	1	protein_coding	467
ENSG0000(	FBXO38	1	protein_coding	-140
ENSG0000(	BOD1	1	protein_coding	761
ENSG0000(	CPLX2	1	protein_coding	721
ENSG0000(	TBC1D7	1	protein_coding	-221
ENSG0000(	TBC1D7	1	protein_coding	430
ENSG0000(	FARS2	1	protein_coding	-255
ENSG0000(	TRIM41	1	protein_coding	-154
ENSG0000(	TRIM41	1	protein_coding	-627
ENSG0000(	HIGD2A	1	protein_coding	-84
ENSG0000(	TNFRSF21	1	protein_coding	27
ENSG0000(	MUT	1	protein_coding	-203
ENSG0000(	CRIP3	1	protein_coding	786
ENSG0000(	RPL7L1	1	protein_coding	-175
ENSG0000(	RPL7L1	1	protein_coding	577
ENSG0000(	NFKBIE	1	protein_coding	544
ENSG0000(	RARS2	1	protein_coding	-113
ENSG0000(	SLC18B1	1	protein_coding	-562
ENSG0000(	MTFR2	1	protein_coding	87
ENSG0000(	DYNLT1	1	protein_coding	96
ENSG0000(	TMEM181	1	protein_coding	-224
ENSG0000(	ZMYM4	1	protein_coding	-453
ENSG0000(	VWDE	1	protein_coding	-89
ENSG0000(	C7orf50	1	protein_coding	883
ENSG0000(	CDCA5	1	protein_coding	-512
ENSG0000(	PURB	1	protein_coding	-531
ENSG0000(	TMEM168	1	protein_coding	-41
ENSG0000(	MEPCE	1	protein_coding	-498
ENSG0000(	AGBL3	1	protein_coding	334
ENSG0000(	LUC7L2	1	protein_coding	989
ENSG0000(	DENND2A	1	protein_coding	522
ENSG0000(	HDAC8	1	protein_coding	-54

ENSG0000(	EBP	1	protein_coding	379
ENSG0000(	OGT	1	protein_coding	-249
ENSG0000(	CCDC25	1	protein_coding	-7
ENSG0000(	HMBOX1	1	protein_coding	-118
ENSG0000(	HMBOX1	1	protein_coding	-805
ENSG0000(	PROSC	1	protein_coding	-8
ENSG0000(	GINS4	1	protein_coding	227
ENSG0000(	MRPS28	1	protein_coding	514
ENSG0000(	MTDH	1	protein_coding	415
ENSG0000(	EBAG9	1	protein_coding	495
ENSG0000(	POLR2K	1	protein_coding	-153
ENSG0000(	UTP23	1	protein_coding	-656
ENSG0000(	ZCCHC7	1	protein_coding	-549
ENSG0000(	INIP	1	protein_coding	450
ENSG0000(	STOM	1	protein_coding	-363
ENSG0000(	STOM	1	protein_coding	461
ENSG0000(	MRRF	1	protein_coding	604
ENSG0000(	ALAD	1	protein_coding	-250
ENSG0000(	WDR31	1	protein_coding	-15
ENSG0000(	SURF6	1	protein_coding	-167
ENSG0000(	SURF6	1	protein_coding	144
ENSG0000(	MED22	1	protein_coding	-213
ENSG0000(	REXO4	1	protein_coding	-80
ENSG0000(	RPL7A	1	protein_coding	131
ENSG0000(	LCN2	1	protein_coding	248
ENSG0000(	LRSAM1	1	protein_coding	-402
ENSG0000(	DPH7	1	protein_coding	-88
ENSG0000(	FAM188A	1	protein_coding	-6
ENSG0000(	RSU1	1	protein_coding	400
ENSG0000(	ST8SIA6	1	protein_coding	-588
ENSG0000(	NRBF2	1	protein_coding	-269
ENSG0000(	POLR3A	1	protein_coding	-45
ENSG0000(	DNAJB12	1	protein_coding	406
ENSG0000(	PPRC1	1	protein_coding	-309
ENSG0000(	PGAP2	1	protein_coding	411
ENSG0000(	ZNF215	1	protein_coding	106
ENSG0000(	EIF3M	1	protein_coding	-94
ENSG0000(	SSRP1	1	protein_coding	-219
ENSG0000(	C11orf49	1	protein_coding	-13
ENSG0000(	ARFGAP2	1	protein_coding	861
ENSG0000(	INTS4	1	protein_coding	-522
ENSG0000(	NPAT	1	protein_coding	-569
ENSG0000(	NPAT	1	protein_coding	71
ENSG0000(	ATM	1	protein_coding	88
ENSG0000(	ATM	1	protein_coding	728
ENSG0000(	LAMTOR1	1	protein_coding	-162
ENSG0000(	TKFC	1	protein_coding	518
ENSG0000(	TMEM138	1	protein_coding	763
ENSG0000(	ROM1	1	protein_coding	155
ENSG0000(	EML3	1	protein_coding	889
ENSG0000(	B3GAT3	1	protein_coding	-158
ENSG0000(	CHEK1	1	protein_coding	240
ENSG0000(	CHEK1	1	protein_coding	649
ENSG0000(	SIDT2	1	protein_coding	-106

ENSG0000(	C20orf144	1	protein_coding	89
ENSG0000(	DSN1	1	protein_coding	13
ENSG0000(	DSN1	1	protein_coding	502
ENSG0000(	CNBD2	1	protein_coding	-75
ENSG0000(	CNBD2	1	protein_coding	-934
ENSG0000(	FERMT3	1	protein_coding	-145
ENSG0000(	FERMT3	1	protein_coding	588
ENSG0000(	MRPL49	1	protein_coding	220
ENSG0000(	FAU	1	protein_coding	71
ENSG0000(	TBX6	1	protein_coding	-140
ENSG0000(	TBX6	1	protein_coding	-989
ENSG0000(	ALDOA	1	protein_coding	24
ENSG0000(	FAM57B	1	protein_coding	-135
ENSG0000(	HIRIP3	1	protein_coding	372
ENSG0000(	HIRIP3	1	protein_coding	675
ENSG0000(	CTF1	1	protein_coding	-696
ENSG0000(	CWC15	1	protein_coding	98
ENSG0000(	KIAA1328	1	protein_coding	359
ENSG0000(	CTAGE5	1	protein_coding	791
ENSG0000(	PDCD4	1	protein_coding	-224
ENSG0000(	SPATA4	1	protein_coding	412
ENSG0000(	CD226	1	protein_coding	-230
ENSG0000(	DIXDC1	1	protein_coding	-285
ENSG0000(	DIXDC1	1	protein_coding	-593
ENSG0000(	DLAT	1	protein_coding	-41
ENSG0000(	DLAT	1	protein_coding	421
ENSG0000(	PIH1D2	1	protein_coding	134
ENSG0000(	C11orf57	1	protein_coding	55
ENSG0000(	TIMM8B	1	protein_coding	7
ENSG0000(	TIMM8B	1	protein_coding	312
ENSG0000(	PTS	1	protein_coding	-199
ENSG0000(	UBC	1	protein_coding	-41
ENSG0000(	SLC7A11	1	protein_coding	512
ENSG0000(	ENKUR	1	protein_coding	-169
ENSG0000(	ENKUR	1	protein_coding	400
ENSG0000(	DCP1B	1	protein_coding	41
ENSG0000(	THRB	1	protein_coding	940
ENSG0000(	RAD9B	1	protein_coding	-251
ENSG0000(	RAD9B	1	protein_coding	704
ENSG0000(	DLG5	1	protein_coding	588
ENSG0000(	EIF4E	1	protein_coding	-120
ENSG0000(	TMEM18	1	protein_coding	-7
ENSG0000(	KCTD14	1	protein_coding	-149
ENSG0000(	NDUFC2	1	protein_coding	-605
ENSG0000(	NDUFC2	1	protein_coding	461
ENSG0000(	NDUFC2	1	protein_coding	820
ENSG0000(	FER	1	protein_coding	745
ENSG0000(	CDC123	1	protein_coding	37
ENSG0000(	SCLT1	1	protein_coding	122
ENSG0000(	C4orf33	1	protein_coding	169
ENSG0000(	ACAD8	1	protein_coding	39
ENSG0000(	THYN1	1	protein_coding	-163
ENSG0000(	KIN	1	protein_coding	-137
ENSG0000(	CENPU	1	protein_coding	-183

ENSG0000(	SLC25A4	1	protein_coding	-10
ENSG0000(	AMN1	1	protein_coding	-443
ENSG0000(	AMN1	1	protein_coding	816
ENSG0000(	GUF1	1	protein_coding	-29
ENSG0000(	FBXO4	1	protein_coding	-226
ENSG0000(	TMEM267	1	protein_coding	-106
ENSG0000(	TMEM267	1	protein_coding	-451
ENSG0000(	CACUL1	1	protein_coding	-61
ENSG0000(	TIAL1	1	protein_coding	-76
ENSG0000(	RABGAP1L	1	protein_coding	743
ENSG0000(	GPATCH11	1	protein_coding	344
ENSG0000(	GPATCH11	1	protein_coding	719
ENSG0000(	GEMIN6	1	protein_coding	45
ENSG0000(	PDK1	1	protein_coding	244
ENSG0000(	UHMK1	1	protein_coding	241
ENSG0000(	POC5	1	protein_coding	148
ENSG0000(	DCLRE1C	1	protein_coding	147
ENSG0000(	ZFP36L2	1	protein_coding	-349
ENSG0000(	ZFP36L2	1	protein_coding	-828
ENSG0000(	NADK2	1	protein_coding	-183
ENSG0000(	GPD1L	1	protein_coding	693
ENSG0000(	SLC30A6	1	protein_coding	-164
ENSG0000(	SAR1B	1	protein_coding	190
ENSG0000(	SAR1B	1	protein_coding	731
ENSG0000(	WDR78	1	protein_coding	-705
ENSG0000(	WDR78	1	protein_coding	197
ENSG0000(	GRM1	1	protein_coding	772
ENSG0000(	RAD17	1	protein_coding	485
ENSG0000(	MED21	1	protein_coding	71
ENSG0000(	MR1	1	protein_coding	201
ENSG0000(	SRP19	1	protein_coding	-302
ENSG0000(	SRP19	1	protein_coding	13
ENSG0000(	TXNDC11	1	protein_coding	-264
ENSG0000(	ANAPC1	1	protein_coding	423
ENSG0000(	CETN3	1	protein_coding	-583
ENSG0000(	CETN3	1	protein_coding	307
ENSG0000(	SYCP2L	1	protein_coding	54
ENSG0000(	HNRNPU	1	protein_coding	-617
ENSG0000(	RANBP2	1	protein_coding	-147
ENSG0000(	NR4A2	1	protein_coding	953
ENSG0000(	INO80C	1	protein_coding	76
ENSG0000(	INO80C	1	protein_coding	643
ENSG0000(	LPCAT1	1	protein_coding	-274
ENSG0000(	UBALD1	1	protein_coding	-267
ENSG0000(	UBALD1	1	protein_coding	-710
ENSG0000(	ING1	1	protein_coding	510
ENSG0000(	UBP1	1	protein_coding	369
ENSG0000(	RPIA	1	protein_coding	-348
ENSG0000(	ZDHHC7	1	protein_coding	673
ENSG0000(	LGI4	1	protein_coding	905
ENSG0000(	CHD1	1	protein_coding	-295
ENSG0000(	DGKE	1	protein_coding	893
ENSG0000(	HS2ST1	1	protein_coding	-491
ENSG0000(	GDPD1	1	protein_coding	464

ENSG0000(	IMPACT	1	protein_coding	49
ENSG0000(	OTULIN	1	protein_coding	-457
ENSG0000(	CC2D1B	1	protein_coding	600
ENSG0000(	TNIK	1	protein_coding	-524
ENSG0000(	BUB3	1	protein_coding	-146
ENSG0000(	CCDC173	1	protein_coding	-690
ENSG0000(	CCDC173	1	protein_coding	49
ENSG0000(	TCEB1	1	protein_coding	-269
ENSG0000(	MRPL39	1	protein_coding	-979
ENSG0000(	ATP5J	1	protein_coding	530
ENSG0000(	GABPA	1	protein_coding	574
ENSG0000(	TSEN2	1	protein_coding	-232
ENSG0000(	CCDC174	1	protein_coding	60
ENSG0000(	DPH3	1	protein_coding	-171
ENSG0000(	OXNAD1	1	protein_coding	-16
ENSG0000(	CXXC1	1	protein_coding	423
ENSG0000(	PPP4R1	1	protein_coding	-392
ENSG0000(	EME1	1	protein_coding	-191
ENSG0000(	ZNF18	1	protein_coding	-191
ENSG0000(	KLF10	1	protein_coding	404
ENSG0000(	ATP6V1C1	1	protein_coding	-820
ENSG0000(	ATP6V1C1	1	protein_coding	111
ENSG0000(	OTUD6B	1	protein_coding	194
ENSG0000(	GTF3C6	1	protein_coding	469
ENSG0000(	AGPAT5	1	protein_coding	-95
ENSG0000(	ZFYVE27	1	protein_coding	-124
ENSG0000(	USP25	1	protein_coding	-373
ENSG0000(	ZCCHC10	1	protein_coding	373
ENSG0000(	RHOC	1	protein_coding	-259
ENSG0000(	PPM1J	1	protein_coding	-884
ENSG0000(	SLC16A1	1	protein_coding	863
ENSG0000(	OXA1L	1	protein_coding	181
ENSG0000(	NUP205	1	protein_coding	266
ENSG0000(	RBM45	1	protein_coding	87
ENSG0000(	RBM45	1	protein_coding	758
ENSG0000(	PDIA4	1	protein_coding	-256
ENSG0000(	PDIA4	1	protein_coding	887
ENSG0000(	PDZD9	1	protein_coding	-58
ENSG0000(	PPARGC1B	1	protein_coding	-353
ENSG0000(	SLC26A2	1	protein_coding	-42
ENSG0000(	CARNMT1	1	protein_coding	784
ENSG0000(	FAM161B	1	protein_coding	448
ENSG0000(	ADK	1	protein_coding	-304
ENSG0000(	ADK	1	protein_coding	162
ENSG0000(	NDUFAF6	1	protein_coding	-568
ENSG0000(	C8orf37	1	protein_coding	-298
ENSG0000(	N6AMT1	1	protein_coding	-122
ENSG0000(	RWDD2B	1	protein_coding	58
ENSG0000(	USP16	1	protein_coding	365
ENSG0000(	CCT8	1	protein_coding	68
ENSG0000(	CCT8	1	protein_coding	649
ENSG0000(	SCAF4	1	protein_coding	-697
ENSG0000(	PCDH1	1	protein_coding	-30
ENSG0000(	MTERF3	1	protein_coding	682

ENSG0000(	PTDSS1	1	protein_coding	-786
ENSG0000(	SUPV3L1	1	protein_coding	-108
ENSG0000(	EEF1A1	1	protein_coding	-163
ENSG0000(	SAMD8	1	protein_coding	595
ENSG0000(	RAB11FIP1	1	protein_coding	-890
ENSG0000(	RAB11FIP1	1	protein_coding	49
ENSG0000(	BAG4	1	protein_coding	-258
ENSG0000(	TBC1D31	1	protein_coding	23
ENSG0000(	WDYHV1	1	protein_coding	786
ENSG0000(	NSMCE2	1	protein_coding	196
ENSG0000(	PRR14	1	protein_coding	-163
ENSG0000(	FBRS	1	protein_coding	-659
ENSG0000(	PHKG2	1	protein_coding	-153
ENSG0000(	SASS6	1	protein_coding	-44
ENSG0000(	GALK2	1	protein_coding	13
ENSG0000(	BUB1B	1	protein_coding	-240
ENSG0000(	BUB1B	1	protein_coding	125
ENSG0000(	EIF4A2	1	protein_coding	715
ENSG0000(	RPUSD3	1	protein_coding	842
ENSG0000(	TATDN2	1	protein_coding	216
ENSG0000(	TATDN2	1	protein_coding	987
ENSG0000(	GHRL	1	protein_coding	-179
ENSG0000(	SEC13	1	protein_coding	23
ENSG0000(	FCHO2	1	protein_coding	-374
ENSG0000(	C1orf27	1	protein_coding	-260
ENSG0000(	CPT2	1	protein_coding	58
ENSG0000(	NECAP2	1	protein_coding	-364
ENSG0000(	NECAP2	1	protein_coding	7
ENSG0000(	PAXIP1	1	protein_coding	-792
ENSG0000(	HTR5A	1	protein_coding	-197
ENSG0000(	ST3GAL2	1	protein_coding	-199
ENSG0000(	DSCR3	1	protein_coding	200
ENSG0000(	DYRK1A	1	protein_coding	21
ENSG0000(	ETS2	1	protein_coding	-131
ENSG0000(	LCA5L	1	protein_coding	-2
ENSG0000(	TMEM164	1	protein_coding	-547
ENSG0000(	C2CD2	1	protein_coding	-678
ENSG0000(	SLC38A10	1	protein_coding	965
ENSG0000(	UBN2	1	protein_coding	907
ENSG0000(	BRAF	1	protein_coding	552
ENSG0000(	CABP1	1	protein_coding	987
ENSG0000(	AP3S2	1	protein_coding	553
ENSG0000(	SPPL3	1	protein_coding	-336
ENSG0000(	FAM213B	1	protein_coding	-623
ENSG0000(	FAM213B	1	protein_coding	-878
ENSG0000(	TNFRSF14	1	protein_coding	998
ENSG0000(	SKI	1	protein_coding	453
ENSG0000(	AGAP1	1	protein_coding	-507
ENSG0000(	PAFAH2	1	protein_coding	-107
ENSG0000(	PAFAH2	1	protein_coding	879
ENSG0000(	MRPL17	1	protein_coding	26
ENSG0000(	NCK1	1	protein_coding	329
ENSG0000(	RHPN1	1	protein_coding	-929
ENSG0000(	EYA3	1	protein_coding	342

ENSG0000(	EYA3	1	protein_coding	964
ENSG0000(	FAM46B	1	protein_coding	622
ENSG0000(	RNF207	1	protein_coding	-350
ENSG0000(	HIST1H2BD	1	protein_coding	-194
ENSG0000(	HIST1H2BD	1	protein_coding	-710
ENSG0000(	HIST1H4H	1	protein_coding	523
ENSG0000(	MITD1	1	protein_coding	-131
ENSG0000(	EIF5B	1	protein_coding	-789
ENSG0000(	RIBC1	1	protein_coding	356
ENSG0000(	AHCYL2	1	protein_coding	-447
ENSG0000(	AHCYL2	1	protein_coding	951
ENSG0000(	SPATA2	1	protein_coding	-333
ENSG0000(	ZC3H18	1	protein_coding	-518
ENSG0000(	GDPD5	1	protein_coding	352
ENSG0000(	PPP1R15B	1	protein_coding	118
ENSG0000(	COPG2	1	protein_coding	814
ENSG0000(	EMSY	1	protein_coding	-241
ENSG0000(	ZSCAN12	1	protein_coding	167
ENSG0000(	TAGLN2	1	protein_coding	33
ENSG0000(	ELK4	1	protein_coding	-205
ENSG0000(	RNF166	1	protein_coding	-78
ENSG0000(	F11R	1	protein_coding	399
ENSG0000(	USF1	1	protein_coding	-221
ENSG0000(	NIT1	1	protein_coding	-405
ENSG0000(	ZNF276	1	protein_coding	-835
ENSG0000(	PINK1	1	protein_coding	-46
ENSG0000(	SLC5A11	1	protein_coding	-983
ENSG0000(	CCAR2	1	protein_coding	-43
ENSG0000(	EPB41	1	protein_coding	-659
ENSG0000(	EPB41	1	protein_coding	907
ENSG0000(	MIS18A	1	protein_coding	780
ENSG0000(	ALG8	1	protein_coding	-157
ENSG0000(	FBXW5	1	protein_coding	-63
ENSG0000(	FBXW5	1	protein_coding	-311
ENSG0000(	FBXW5	1	protein_coding	730
ENSG0000(	C21orf59	1	protein_coding	-190
ENSG0000(	C21orf59	1	protein_coding	311
ENSG0000(	SYNJ1	1	protein_coding	-155
ENSG0000(	SYNJ1	1	protein_coding	-520
ENSG0000(	PAXBP1	1	protein_coding	-288
ENSG0000(	IFNAR2	1	protein_coding	618
ENSG0000(	SON	1	protein_coding	-151
ENSG0000(	DONSON	1	protein_coding	-383
ENSG0000(	UBE2Z	1	protein_coding	-211
ENSG0000(	SNF8	1	protein_coding	-28
ENSG0000(	IGF2BP1	1	protein_coding	-926
ENSG0000(	MORC3	1	protein_coding	792
ENSG0000(	HLCS	1	protein_coding	-235
ENSG0000(	HLCS	1	protein_coding	513
ENSG0000(	ADIPOR1	1	protein_coding	-191
ENSG0000(	BTG2	1	protein_coding	-504
ENSG0000(	BTG2	1	protein_coding	839
ENSG0000(	UBR1	1	protein_coding	-88
ENSG0000(	MED8	1	protein_coding	-363

ENSG0000(	RSPRY1	1	protein_coding	763
ENSG0000(	GPBP1L1	1	protein_coding	720
ENSG0000(	TMEM69	1	protein_coding	180
ENSG0000(	ACE	1	protein_coding	-794
ENSG0000(	TEPP	1	protein_coding	13
ENSG0000(	ZYX	1	protein_coding	-978
ENSG0000(	CCDC117	1	protein_coding	20
ENSG0000(	ZNF230	1	protein_coding	-26
ENSG0000(	ZNF235	1	protein_coding	-50
ENSG0000(	DFFA	1	protein_coding	65
ENSG0000(	IQCC	1	protein_coding	-75
ENSG0000(	TMEM234	1	protein_coding	-77
ENSG0000(	TMEM234	1	protein_coding	869
ENSG0000(	BSDC1	1	protein_coding	428
ENSG0000(	SSU72	1	protein_coding	-157
ENSG0000(	UBE2J2	1	protein_coding	695
ENSG0000(	UBASH3A	1	protein_coding	388
ENSG0000(	RSPH1	1	protein_coding	-40
ENSG0000(	SLC37A1	1	protein_coding	387
ENSG0000(	WDR4	1	protein_coding	-17
ENSG0000(	WDR4	1	protein_coding	655
ENSG0000(	NDUFV3	1	protein_coding	-58
ENSG0000(	NDUFV3	1	protein_coding	-730
ENSG0000(	PKNOX1	1	protein_coding	-868
ENSG0000(	HSF2BP	1	protein_coding	-292
ENSG0000(	HSF2BP	1	protein_coding	5
ENSG0000(	RRP1B	1	protein_coding	-59
ENSG0000(	RRP1B	1	protein_coding	238
ENSG0000(	PDXK	1	protein_coding	-995
ENSG0000(	PDXK	1	protein_coding	267
ENSG0000(	PDXK	1	protein_coding	879
ENSG0000(	G6PD	1	protein_coding	991
ENSG0000(	CSTB	1	protein_coding	261
ENSG0000(	C21orf33	1	protein_coding	-176
ENSG0000(	C21orf33	1	protein_coding	141
ENSG0000(	C21orf2	1	protein_coding	-99
ENSG0000(	FAM207A	1	protein_coding	-838
ENSG0000(	RALGDS	1	protein_coding	-203
ENSG0000(	FTCD	1	protein_coding	-754
ENSG0000(	SPATC1L	1	protein_coding	-452
ENSG0000(	SPATC1L	1	protein_coding	125
ENSG0000(	LSS	1	protein_coding	665
ENSG0000(	VAV2	1	protein_coding	-656
ENSG0000(	MCM3AP	1	protein_coding	18
ENSG0000(	PRMT2	1	protein_coding	536
ENSG0000(	GPSM1	1	protein_coding	-769
ENSG0000(	TOR2A	1	protein_coding	-117
ENSG0000(	TOR2A	1	protein_coding	586
ENSG0000(	ST6GALNAC6	1	protein_coding	401
ENSG0000(	SHKBP1	1	protein_coding	767
ENSG0000(	ZER1	1	protein_coding	138
ENSG0000(	SPTBN4	1	protein_coding	93
ENSG0000(	MED27	1	protein_coding	-15
ENSG0000(	DEDD2	1	protein_coding	279

ENSG0000(	MPZL3	1	protein_coding	156
ENSG0000(	NEK8	1	protein_coding	-429
ENSG0000(	TLCD1	1	protein_coding	-283
ENSG0000(	S100A1	1	protein_coding	-131
ENSG0000(	SHC1	1	protein_coding	-516
ENSG0000(	SHC1	1	protein_coding	544
ENSG0000(	VPS11	1	protein_coding	192
ENSG0000(	UBE2Q1	1	protein_coding	-340
ENSG0000(	ANO10	1	protein_coding	971
ENSG0000(	FDPS	1	protein_coding	-2
ENSG0000(	SLC25A44	1	protein_coding	-802
ENSG0000(	SLC25A44	1	protein_coding	11
ENSG0000(	UBQLN4	1	protein_coding	-644
ENSG0000(	CYP3A7	1	protein_coding	810
ENSG0000(	TONSL	1	protein_coding	-724
ENSG0000(	PPP1R16A	1	protein_coding	979
ENSG0000(	ALKBH4	1	protein_coding	-9
ENSG0000(	RPL8	1	protein_coding	107
ENSG0000(	LRWD1	1	protein_coding	-43
ENSG0000(	FBXL13	1	protein_coding	58
ENSG0000(	MFSD12	1	protein_coding	291
ENSG0000(	YDJC	1	protein_coding	993
ENSG0000(	CCDC116	1	protein_coding	435
ENSG0000(	PCYT1A	1	protein_coding	537
ENSG0000(	PCYT1A	1	protein_coding	877
ENSG0000(	THAP8	1	protein_coding	154
ENSG0000(	THAP8	1	protein_coding	655
ENSG0000(	PRPSAP1	1	protein_coding	-589
ENSG0000(	SRSF2	1	protein_coding	90
ENSG0000(	TMEM143	1	protein_coding	339
ENSG0000(	ALDH16A1	1	protein_coding	-80
ENSG0000(	ALDH16A1	1	protein_coding	414
ENSG0000(	EMC10	1	protein_coding	174
ENSG0000(	SYCE2	1	protein_coding	8
ENSG0000(	SPC24	1	protein_coding	-136
ENSG0000(	SPC24	1	protein_coding	565
ENSG0000(	LEMD2	1	protein_coding	-82
ENSG0000(	MED11	1	protein_coding	-107
ENSG0000(	MED11	1	protein_coding	100
ENSG0000(	C16orf59	1	protein_coding	-286
ENSG0000(	CCNF	1	protein_coding	-351
ENSG0000(	CCNF	1	protein_coding	-803
ENSG0000(	NTN3	1	protein_coding	610
ENSG0000(	PAQR4	1	protein_coding	-309
ENSG0000(	CLPB	1	protein_coding	197
ENSG0000(	CLPB	1	protein_coding	764
ENSG0000(	CYB561A3	1	protein_coding	-464
ENSG0000(	LBHD1	1	protein_coding	270
ENSG0000(	TTC9C	1	protein_coding	-752
ENSG0000(	TTC9C	1	protein_coding	868
ENSG0000(	SLC25A45	1	protein_coding	696
ENSG0000(	ZFPL1	1	protein_coding	454
ENSG0000(	RPS6KA4	1	protein_coding	-989
ENSG0000(	RPS6KA4	1	protein_coding	87

ENSG0000(	RPS6KA4	1	protein_coding	277
ENSG0000(	TAL1	1	protein_coding	-442
ENSG0000(	CMPK1	1	protein_coding	-223
ENSG0000(	C1orf123	1	protein_coding	-473
ENSG0000(	C1orf123	1	protein_coding	100
ENSG0000(	MAGOH	1	protein_coding	3
ENSG0000(	FAM151A	1	protein_coding	-932
ENSG0000(	NOL9	1	protein_coding	539
ENSG0000(	GMEB1	1	protein_coding	-300
ENSG0000(	LZIC	1	protein_coding	-268
ENSG0000(	TMEM82	1	protein_coding	-582
ENSG0000(	LAPTM5	1	protein_coding	416
ENSG0000(	SDC3	1	protein_coding	-856
ENSG0000(	PEF1	1	protein_coding	-120
ENSG0000(	SYNC	1	protein_coding	-11
ENSG0000(	RBBP4	1	protein_coding	-342
ENSG0000(	KIAA1522	1	protein_coding	-807
ENSG0000(	KIAA1522	1	protein_coding	777
ENSG0000(	TSSK3	1	protein_coding	-579
ENSG0000(	UBXN10	1	protein_coding	-690
ENSG0000(	UBXN10	1	protein_coding	464
ENSG0000(	FAAP20	1	protein_coding	81
ENSG0000(	TM2D1	1	protein_coding	151
ENSG0000(	USP1	1	protein_coding	-487
ENSG0000(	FUBP1	1	protein_coding	-383
ENSG0000(	DNAJB4	1	protein_coding	319
ENSG0000(	ATXN7L2	1	protein_coding	820
ENSG0000(	AGL	1	protein_coding	-591
ENSG0000(	AGL	1	protein_coding	273
ENSG0000(	EXTL2	1	protein_coding	-755
ENSG0000(	SLC30A7	1	protein_coding	678
ENSG0000(	ZNF496	1	protein_coding	-660
ENSG0000(	TRIM58	1	protein_coding	-177
ENSG0000(	FCRLB	1	protein_coding	582
ENSG0000(	C1orf74	1	protein_coding	-330
ENSG0000(	FLVCR1	1	protein_coding	-337
ENSG0000(	RBM15	1	protein_coding	-317
ENSG0000(	RBM15	1	protein_coding	558
ENSG0000(	AXDND1	1	protein_coding	322
ENSG0000(	IER5	1	protein_coding	-96
ENSG0000(	BPNT1	1	protein_coding	526
ENSG0000(	BROX	1	protein_coding	-662
ENSG0000(	BROX	1	protein_coding	460
ENSG0000(	TFB2M	1	protein_coding	-67
ENSG0000(	CNST	1	protein_coding	-52
ENSG0000(	B3GALNT2	1	protein_coding	-436
ENSG0000(	CAPN2	1	protein_coding	-600
ENSG0000(	MRPL55	1	protein_coding	71
ENSG0000(	MRPL55	1	protein_coding	469
ENSG0000(	PUS10	1	protein_coding	797
ENSG0000(	PEX13	1	protein_coding	238
ENSG0000(	TYW5	1	protein_coding	-99
ENSG0000(	C2orf47	1	protein_coding	519
ENSG0000(	ARL5A	1	protein_coding	-317

ENSG0000(	CLHC1	1	protein_coding	188
ENSG0000(	DUSP19	1	protein_coding	-130
ENSG0000(	DUSP19	1	protein_coding	378
ENSG0000(	VSNL1	1	protein_coding	-661
ENSG0000(	H3F3A	1	protein_coding	-389
ENSG0000(	BBS5	1	protein_coding	518
ENSG0000(	PDLIM5	1	protein_coding	758
ENSG0000(	RPRD2	1	protein_coding	74
ENSG0000(	ANKRD23	1	protein_coding	-236
ENSG0000(	LYSMD1	1	protein_coding	-7
ENSG0000(	TMOD4	1	protein_coding	614
ENSG0000(	ERCC3	1	protein_coding	600
ENSG0000(	IWS1	1	protein_coding	-361
ENSG0000(	S100A11	1	protein_coding	76
ENSG0000(	CCNYL1	1	protein_coding	-490
ENSG0000(	GPR155	1	protein_coding	800
ENSG0000(	PYGO2	1	protein_coding	-224
ENSG0000(	LENEP	1	protein_coding	-389
ENSG0000(	LENEP	1	protein_coding	577
ENSG0000(	EOGT	1	protein_coding	243
ENSG0000(	NAXE	1	protein_coding	-310
ENSG0000(	TMEM79	1	protein_coding	113
ENSG0000(	GLB1L	1	protein_coding	-118
ENSG0000(	STT3B	1	protein_coding	-738
ENSG0000(	CHCHD4	1	protein_coding	-755
ENSG0000(	CHCHD4	1	protein_coding	728
ENSG0000(	SERPINI1	1	protein_coding	-401
ENSG0000(	RPL22L1	1	protein_coding	130
ENSG0000(	RPL22L1	1	protein_coding	479
ENSG0000(	COX18	1	protein_coding	-184
ENSG0000(	COX18	1	protein_coding	379
ENSG0000(	TIPARP	1	protein_coding	857
ENSG0000(	CCNL1	1	protein_coding	311
ENSG0000(	RPL9	1	protein_coding	61
ENSG0000(	SMIM14	1	protein_coding	-101
ENSG0000(	RPP14	1	protein_coding	18
ENSG0000(	IL17RC	1	protein_coding	-347
ENSG0000(	CRELD1	1	protein_coding	-500
ENSG0000(	CRELD1	1	protein_coding	-857
ENSG0000(	CRELD1	1	protein_coding	409
ENSG0000(	TTC14	1	protein_coding	-29
ENSG0000(	CXCL3	1	protein_coding	-730
ENSG0000(	RCHY1	1	protein_coding	12
ENSG0000(	RCHY1	1	protein_coding	587
ENSG0000(	ZNF513	1	protein_coding	-347
ENSG0000(	SLC4A1AP	1	protein_coding	260
ENSG0000(	ZDHHC3	1	protein_coding	-301
ENSG0000(	ZDHHC3	1	protein_coding	611
ENSG0000(	ELP6	1	protein_coding	98
ENSG0000(	SMIM12	1	protein_coding	-105
ENSG0000(	ZMYM6	1	protein_coding	260
ENSG0000(	MEAF6	1	protein_coding	-275
ENSG0000(	POLR2H	1	protein_coding	-519
ENSG0000(	TMEM41A	1	protein_coding	-323

ENSG0000(	TMEM41A	1	protein_coding	309
ENSG0000(	RPN1	1	protein_coding	346
ENSG0000(	IFT122	1	protein_coding	355
ENSG0000(	RFC4	1	protein_coding	471
ENSG0000(	RFC4	1	protein_coding	736
ENSG0000(	UBXN7	1	protein_coding	-368
ENSG0000(	PIGX	1	protein_coding	-447
ENSG0000(	PIGX	1	protein_coding	591
ENSG0000(	S100P	1	protein_coding	-425
ENSG0000(	S100P	1	protein_coding	385
ENSG0000(	ABLIM2	1	protein_coding	-345
ENSG0000(	C1orf50	1	protein_coding	102
ENSG0000(	ZNF691	1	protein_coding	-226
ENSG0000(	ZNF691	1	protein_coding	619
ENSG0000(	H2AFZ	1	protein_coding	115
ENSG0000(	SLC9B1	1	protein_coding	-54
ENSG0000(	APEH	1	protein_coding	-727
ENSG0000(	HSPA4L	1	protein_coding	782
ENSG0000(	MFSD8	1	protein_coding	-38
ENSG0000(	MFSD8	1	protein_coding	726
ENSG0000(	ABHD18	1	protein_coding	-36
ENSG0000(	ABHD18	1	protein_coding	728
ENSG0000(	MAD2L1	1	protein_coding	146
ENSG0000(	NAA15	1	protein_coding	-146
ENSG0000(	ARFIP1	1	protein_coding	206
ENSG0000(	ANAPC10	1	protein_coding	-352
ENSG0000(	ANAPC10	1	protein_coding	1
ENSG0000(	ANAPC10	1	protein_coding	284
ENSG0000(	ABCE1	1	protein_coding	326
ENSG0000(	ABCE1	1	protein_coding	609
ENSG0000(	ABCE1	1	protein_coding	962
ENSG0000(	LMBRD2	1	protein_coding	948
ENSG0000(	ADGRV1	1	protein_coding	760
ENSG0000(	PRRC1	1	protein_coding	-241
ENSG0000(	ARSK	1	protein_coding	282
ENSG0000(	TIGD6	1	protein_coding	591
ENSG0000(	CAGE1	1	protein_coding	45
ENSG0000(	CASP3	1	protein_coding	-545
ENSG0000(	PRIMPOL	1	protein_coding	442
ENSG0000(	RICTOR	1	protein_coding	955
ENSG0000(	ANKRA2	1	protein_coding	-135
ENSG0000(	UBLCP1	1	protein_coding	-135
ENSG0000(	UTP15	1	protein_coding	379
ENSG0000(	8-Sep	1	protein_coding	-80
ENSG0000(	CITED2	1	protein_coding	-22
ENSG0000(	CREBRF	1	protein_coding	-420
ENSG0000(	GALNT10	1	protein_coding	-901
ENSG0000(	SAP30L	1	protein_coding	-765
ENSG0000(	RPS14	1	protein_coding	645
ENSG0000(	COG5	1	protein_coding	74
ENSG0000(	RELL2	1	protein_coding	-337
ENSG0000(	KCNK5	1	protein_coding	709
ENSG0000(	ZNF12	1	protein_coding	-402
ENSG0000(	USP49	1	protein_coding	-381

ENSG0000(	SPIDR	1	protein_coding	-172
ENSG0000(	GPR146	1	protein_coding	-757
ENSG0000(	GPER1	1	protein_coding	180
ENSG0000(	CDK5	1	protein_coding	-467
ENSG0000(	CDK5	1	protein_coding	469
ENSG0000(	SLC4A2	1	protein_coding	-191
ENSG0000(	SLC4A2	1	protein_coding	-891
ENSG0000(	SLC4A2	1	protein_coding	852
ENSG0000(	FASTK	1	protein_coding	-973
ENSG0000(	FASTK	1	protein_coding	727
ENSG0000(	TMUB1	1	protein_coding	-321
ENSG0000(	KIAA0196	1	protein_coding	-34
ENSG0000(	NUDT2	1	protein_coding	-311
ENSG0000(	TMEM65	1	protein_coding	-264
ENSG0000(	TMEM65	1	protein_coding	-514
ENSG0000(	LETM2	1	protein_coding	125
ENSG0000(	KIAA1958	1	protein_coding	-386
ENSG0000(	STRBP	1	protein_coding	-720
ENSG0000(	GAPVD1	1	protein_coding	-146
ENSG0000(	GAPVD1	1	protein_coding	768
ENSG0000(	AQP3	1	protein_coding	-477
ENSG0000(	VCP	1	protein_coding	384
ENSG0000(	STOML2	1	protein_coding	-93
ENSG0000(	INTS6L	1	protein_coding	22
ENSG0000(	INPPL1	1	protein_coding	-600
ENSG0000(	INPPL1	1	protein_coding	-969
ENSG0000(	REEP3	1	protein_coding	-481
ENSG0000(	NUDT5	1	protein_coding	143
ENSG0000(	ATP5C1	1	protein_coding	-10
ENSG0000(	VSTM4	1	protein_coding	500
ENSG0000(	VDAC2	1	protein_coding	-70
ENSG0000(	VDAC2	1	protein_coding	575
ENSG0000(	PDZD8	1	protein_coding	-347
ENSG0000(	GHITM	1	protein_coding	-369
ENSG0000(	GHITM	1	protein_coding	625
ENSG0000(	FAM69B	1	protein_coding	928
ENSG0000(	ZMYND19	1	protein_coding	938
ENSG0000(	DDX21	1	protein_coding	63
ENSG0000(	TMEM55B	1	protein_coding	-409
ENSG0000(	METTL3	1	protein_coding	-79
ENSG0000(	FRAT1	1	protein_coding	-506
ENSG0000(	LARGE2	1	protein_coding	-57
ENSG0000(	SLC39A13	1	protein_coding	406
ENSG0000(	PTER	1	protein_coding	191
ENSG0000(	CEP295	1	protein_coding	594
ENSG0000(	R3HCC1L	1	protein_coding	-223
ENSG0000(	CEP57	1	protein_coding	-194
ENSG0000(	SPRED1	1	protein_coding	-475
ENSG0000(	IKBIP	1	protein_coding	321
ENSG0000(	IKBIP	1	protein_coding	843
ENSG0000(	RPUSD2	1	protein_coding	-25
ENSG0000(	BRD7	1	protein_coding	979
ENSG0000(	CKB	1	protein_coding	-793
ENSG0000(	TRMT61A	1	protein_coding	452

ENSG0000(	BTRC	1	protein_coding	-199
ENSG0000(	POLL	1	protein_coding	990
ENSG0000(	API5	1	protein_coding	716
ENSG0000(	NOLC1	1	protein_coding	52
ENSG0000(	ALKBH3	1	protein_coding	-53
ENSG0000(	COPS2	1	protein_coding	-7
ENSG0000(	SGPL1	1	protein_coding	731
ENSG0000(	ARIH1	1	protein_coding	-103
ENSG0000(	COX11	1	protein_coding	-700
ENSG0000(	STXBP4	1	protein_coding	759
ENSG0000(	CUL5	1	protein_coding	257
ENSG0000(	BORCS7	1	protein_coding	-156
ENSG0000(	C2	1	protein_coding	26
ENSG0000(	TMEM100	1	protein_coding	-42
ENSG0000(	ANAPC16	1	protein_coding	-95
ENSG0000(	ANAPC16	1	protein_coding	905
ENSG0000(	APBB1	1	protein_coding	-91
ENSG0000(	NUDT13	1	protein_coding	88
ENSG0000(	TRIM44	1	protein_coding	347
ENSG0000(	ILK	1	protein_coding	437
ENSG0000(	TAF10	1	protein_coding	-86
ENSG0000(	USP54	1	protein_coding	-424
ENSG0000(	KIAA0355	1	protein_coding	-421
ENSG0000(	CENPN	1	protein_coding	780
ENSG0000(	C16orf46	1	protein_coding	42
ENSG0000(	TMEM41B	1	protein_coding	-210
ENSG0000(	LEO1	1	protein_coding	56
ENSG0000(	ZNF143	1	protein_coding	174
ENSG0000(	WEE1	1	protein_coding	-554
ENSG0000(	WEE1	1	protein_coding	-877
ENSG0000(	MAPK7	1	protein_coding	357
ENSG0000(	ZNF3	1	protein_coding	289
ENSG0000(	ZNF3	1	protein_coding	735
ENSG0000(	TK2	1	protein_coding	749
ENSG0000(	TMEM135	1	protein_coding	-303
ENSG0000(	IQCD	1	protein_coding	465
ENSG0000(	FAM96B	1	protein_coding	-199
ENSG0000(	BLCAP	1	protein_coding	-107
ENSG0000(	BLCAP	1	protein_coding	929
ENSG0000(	COG1	1	protein_coding	623
ENSG0000(	B2M	1	protein_coding	127
ENSG0000(	ZNF592	1	protein_coding	-696
ENSG0000(	AP1G1	1	protein_coding	-213
ENSG0000(	AP1G1	1	protein_coding	213
ENSG0000(	AP1G1	1	protein_coding	543
ENSG0000(	PPIB	1	protein_coding	-413
ENSG0000(	FAM96A	1	protein_coding	220
ENSG0000(	FAM96A	1	protein_coding	717
ENSG0000(	KIAA0101	1	protein_coding	-62
ENSG0000(	MESP1	1	protein_coding	508
ENSG0000(	CLPX	1	protein_coding	-407
ENSG0000(	ATP23	1	protein_coding	457
ENSG0000(	STX3	1	protein_coding	-904
ENSG0000(	MRPL16	1	protein_coding	70

ENSG0000(	TSC22D4	1	protein_coding	187
ENSG0000(	SMAD3	1	protein_coding	554
ENSG0000(	MAP1A	1	protein_coding	330
ENSG0000(	RCCD1	1	protein_coding	171
ENSG0000(	DUSP18	1	protein_coding	-158
ENSG0000(	TTC16	1	protein_coding	-218
ENSG0000(	PIP5KL1	1	protein_coding	-396
ENSG0000(	PIP5KL1	1	protein_coding	-898
ENSG0000(	GOLGA2	1	protein_coding	-215
ENSG0000(	GOLGA2	1	protein_coding	-891
ENSG0000(	GOLGA2	1	protein_coding	641
ENSG0000(	TRUB2	1	protein_coding	-92
ENSG0000(	COQ4	1	protein_coding	299
ENSG0000(	SLC27A4	1	protein_coding	849
ENSG0000(	DOLPP1	1	protein_coding	-575
ENSG0000(	CCDC103	1	protein_coding	399
ENSG0000(	ENDOG	1	protein_coding	-207
ENSG0000(	SP2	1	protein_coding	-874
ENSG0000(	COQ7	1	protein_coding	-110
ENSG0000(	FBXO22	1	protein_coding	-2
ENSG0000(	FBXO22	1	protein_coding	719
ENSG0000(	TBC1D16	1	protein_coding	-699
ENSG0000(	ART5	1	protein_coding	-415
ENSG0000(	STIM1	1	protein_coding	-61
ENSG0000(	RRM1	1	protein_coding	248
ENSG0000(	FN3K	1	protein_coding	414
ENSG0000(	ZNF23	1	protein_coding	833
ENSG0000(	IRGQ	1	protein_coding	-381
ENSG0000(	ZNF226	1	protein_coding	-167
ENSG0000(	GNG8	1	protein_coding	853
ENSG0000(	LPO	1	protein_coding	934
ENSG0000(	GPX4	1	protein_coding	-217
ENSG0000(	MIDN	1	protein_coding	-440
ENSG0000(	MIDN	1	protein_coding	571
ENSG0000(	MVD	1	protein_coding	188
ENSG0000(	ANKRD11	1	protein_coding	-259
ENSG0000(	SPATA33	1	protein_coding	26
ENSG0000(	SPATA33	1	protein_coding	656
ENSG0000(	PROCA1	1	protein_coding	-766
ENSG0000(	RPL13	1	protein_coding	-85
ENSG0000(	CACNB3	1	protein_coding	941
ENSG0000(	DHRS13	1	protein_coding	797
ENSG0000(	RAB4B	1	protein_coding	-413
ENSG0000(	RAB4B	1	protein_coding	763
ENSG0000(	PROSER3	1	protein_coding	-178
ENSG0000(	LENG8	1	protein_coding	223
ENSG0000(	LENG8	1	protein_coding	873
ENSG0000(	TMEM145	1	protein_coding	713
ENSG0000(	ZNF526	1	protein_coding	-409
ENSG0000(	TRAPPC9	1	protein_coding	56
ENSG0000(	TRAPPC9	1	protein_coding	802
ENSG0000(	ZNF146	1	protein_coding	-273
ENSG0000(	ZNF146	1	protein_coding	252
ENSG0000(	ZNF146	1	protein_coding	795

ENSG0000(	YIF1B	1	protein_coding	7
ENSG0000(	CHAF1A	1	protein_coding	812
ENSG0000(	MFSD3	1	protein_coding	-832
ENSG0000(	WDR81	1	protein_coding	21
ENSG0000(	WDR81	1	protein_coding	816
ENSG0000(	HSD11B1L	1	protein_coding	191
ENSG0000(	C19orf48	1	protein_coding	212
ENSG0000(	CTD-255008.5	1	protein_coding	-434
ENSG0000(	CD320	1	protein_coding	-190
ENSG0000(	CD320	1	protein_coding	427
ENSG0000(	SPRYD3	1	protein_coding	698
ENSG0000(	NDUFV1	1	protein_coding	-68
ENSG0000(	CDK2AP2	1	protein_coding	-14
ENSG0000(	CD300A	1	protein_coding	-404
ENSG0000(	SRP68	1	protein_coding	-62
ENSG0000(	SRP68	1	protein_coding	-347
ENSG0000(	TMC8	1	protein_coding	-286
ENSG0000(	TMEM68	1	protein_coding	826
ENSG0000(	FAM234A	1	protein_coding	856
ENSG0000(	ZNF598	1	protein_coding	-325
ENSG0000(	E4F1	1	protein_coding	110
ENSG0000(	ECI1	1	protein_coding	93
ENSG0000(	ABCA3	1	protein_coding	-223
ENSG0000(	SRRM2	1	protein_coding	-568
ENSG0000(	DDB1	1	protein_coding	70
ENSG0000(	RAB3IL1	1	protein_coding	200
ENSG0000(	RAB3IL1	1	protein_coding	969
ENSG0000(	BEST1	1	protein_coding	342
ENSG0000(	SLC3A2	1	protein_coding	33
ENSG0000(	ATG16L2	1	protein_coding	-416
ENSG0000(	ATG16L2	1	protein_coding	298
ENSG0000(	C2CD3	1	protein_coding	-102
ENSG0000(	TTC21A	1	protein_coding	4
ENSG0000(	RPSA	1	protein_coding	41
ENSG0000(	FADD	1	protein_coding	380
ENSG0000(	FADD	1	protein_coding	908
ENSG0000(	PBK	1	protein_coding	45
ENSG0000(	COPS6	1	protein_coding	-290
ENSG0000(	PAFAH1B2	1	protein_coding	-211
ENSG0000(	SETD5	1	protein_coding	522
ENSG0000(	HIST3H3	1	protein_coding	551
ENSG0000(	THAP9	1	protein_coding	49
ENSG0000(	DDIT4	1	protein_coding	-243
ENSG0000(	DDIT4	1	protein_coding	-966
ENSG0000(	DDIT4	1	protein_coding	915
ENSG0000(	GNG4	1	protein_coding	-606
ENSG0000(	IRF2BP2	1	protein_coding	-992
ENSG0000(	COA6	1	protein_coding	149
ENSG0000(	THAP11	1	protein_coding	-17
ENSG0000(	MMADHC	1	protein_coding	41
ENSG0000(	HIST1H1E	1	protein_coding	-122
ENSG0000(	PCMTD1	1	protein_coding	-188
ENSG0000(	IRF2	1	protein_coding	-533
ENSG0000(	ARF4	1	protein_coding	360

ENSG0000(	MLKL	1	protein_coding	465
ENSG0000(	COG7	1	protein_coding	-136
ENSG0000(	TXNDC2	1	protein_coding	-261
ENSG0000(	TXNDC2	1	protein_coding	458
ENSG0000(	REEP4	1	protein_coding	240
ENSG0000(	REEP4	1	protein_coding	728
ENSG0000(	ATXN2L	1	protein_coding	-566
ENSG0000(	POLR3D	1	protein_coding	-328
ENSG0000(	FEN1	1	protein_coding	77
ENSG0000(	SDPR	1	protein_coding	688
ENSG0000(	SERINC2	1	protein_coding	687
ENSG0000(	TMEM223	1	protein_coding	364
ENSG0000(	TMUB2	1	protein_coding	100
ENSG0000(	NDUFS5	1	protein_coding	-508
ENSG0000(	NDUFS5	1	protein_coding	266
ENSG0000(	TMEM208	1	protein_coding	38
ENSG0000(	AHCYL1	1	protein_coding	-122
ENSG0000(	GSTM4	1	protein_coding	-154
ENSG0000(	ABHD15	1	protein_coding	-716
ENSG0000(	ZBTB5	1	protein_coding	-830
ENSG0000(	ADAL	1	protein_coding	27
ENSG0000(	LCMT2	1	protein_coding	-95
ENSG0000(	SNTB2	1	protein_coding	-594
ENSG0000(	DDX19A	1	protein_coding	-3
ENSG0000(	FEM1B	1	protein_coding	-100
ENSG0000(	ATP5I	1	protein_coding	-93
ENSG0000(	HSPBAP1	1	protein_coding	-105
ENSG0000(	CSNK1G1	1	protein_coding	57
ENSG0000(	ATF5	1	protein_coding	-842
ENSG0000(	ATF5	1	protein_coding	378
ENSG0000(	ATF5	1	protein_coding	969
ENSG0000(	UBE2V2	1	protein_coding	133
ENSG0000(	PCSK9	1	protein_coding	-985
ENSG0000(	XPO6	1	protein_coding	-394
ENSG0000(	CD2BP2	1	protein_coding	-324
ENSG0000(	TBC1D10B	1	protein_coding	-698
ENSG0000(	THBS3	1	protein_coding	663
ENSG0000(	SLC50A1	1	protein_coding	303
ENSG0000(	EFNA1	1	protein_coding	-297
ENSG0000(	GPRIN1	1	protein_coding	747
ENSG0000(	MRPL1	1	protein_coding	301
ENSG0000(	PGM2	1	protein_coding	-430
ENSG0000(	SLC33A1	1	protein_coding	-226
ENSG0000(	SLC33A1	1	protein_coding	360
ENSG0000(	CRADD	1	protein_coding	-166
ENSG0000(	SIN3A	1	protein_coding	-217
ENSG0000(	SDC2	1	protein_coding	832
ENSG0000(	PCBP1	1	protein_coding	809
ENSG0000(	DTWD2	1	protein_coding	-263
ENSG0000(	DTWD2	1	protein_coding	336
ENSG0000(	CLIC3	1	protein_coding	-191
ENSG0000(	INO80E	1	protein_coding	68
ENSG0000(	INO80E	1	protein_coding	468
ENSG0000(	INO80E	1	protein_coding	771

ENSG0000(	DFFB	1	protein_coding	106
ENSG0000(	NFU1	1	protein_coding	-148
ENSG0000(	NFU1	1	protein_coding	79
ENSG0000(	C15orf40	1	protein_coding	-317
ENSG0000(	C15orf40	1	protein_coding	632
ENSG0000(	FAM103A1	1	protein_coding	-275
ENSG0000(	LRRC45	1	protein_coding	411
ENSG0000(	STRA13	1	protein_coding	395
ENSG0000(	ASPSCR1	1	protein_coding	-42
ENSG0000(	CNBP	1	protein_coding	52
ENSG0000(	DUS1L	1	protein_coding	34
ENSG0000(	ZNF32	1	protein_coding	-424
ENSG0000(	LIMS1	1	protein_coding	-521
ENSG0000(	UGP2	1	protein_coding	-370
ENSG0000(	HNRNPF	1	protein_coding	-303
ENSG0000(	HNRNPF	1	protein_coding	177
ENSG0000(	BTD	1	protein_coding	321
ENSG0000(	TRIM56	1	protein_coding	-308
ENSG0000(	OTUD3	1	protein_coding	-334
ENSG0000(	GUSB	1	protein_coding	11
ENSG0000(	ZNF764	1	protein_coding	-780
ENSG0000(	ZNF764	1	protein_coding	267
ENSG0000(	ZNF747	1	protein_coding	-507
ENSG0000(	ZNF747	1	protein_coding	594
ENSG0000(	ZNF768	1	protein_coding	-174
ENSG0000(	SF3B5	1	protein_coding	-1
ENSG0000(	TIGD4	1	protein_coding	-378
ENSG0000(	CNTROB	1	protein_coding	668
ENSG0000(	TRAPPC1	1	protein_coding	-645
ENSG0000(	HNRNPA3	1	protein_coding	-93
ENSG0000(	SLC16A5	1	protein_coding	-964
ENSG0000(	NANP	1	protein_coding	-562
ENSG0000(	GLB1	1	protein_coding	-208
ENSG0000(	CRTAP	1	protein_coding	314
ENSG0000(	HSPB2	1	protein_coding	-263
ENSG0000(	HSPB2	1	protein_coding	-662
ENSG0000(	UBB	1	protein_coding	217
ENSG0000(	SETMAR	1	protein_coding	-25
ENSG0000(	SETMAR	1	protein_coding	503
ENSG0000(	KRT8	1	protein_coding	179
ENSG0000(	HARS	1	protein_coding	541
ENSG0000(	SLC23A1	1	protein_coding	-965
ENSG0000(	NUDT9	1	protein_coding	-17
ENSG0000(	PA2G4	1	protein_coding	182
ENSG0000(	NUDCD2	1	protein_coding	-21
ENSG0000(	NUDCD2	1	protein_coding	-786
ENSG0000(	IRF2BP1	1	protein_coding	-873
ENSG0000(	HSPA4	1	protein_coding	-336
ENSG0000(	FOXA3	1	protein_coding	-680
ENSG0000(	FOXA3	1	protein_coding	494
ENSG0000(	GTSF1	1	protein_coding	-136
ENSG0000(	ARMC10	1	protein_coding	-95
ENSG0000(	ACYP2	1	protein_coding	-921
ENSG0000(	ZNF296	1	protein_coding	-74

ENSG0000(	POLH	1	protein_coding	-600
ENSG0000(	POLH	1	protein_coding	229
ENSG0000(	TRIAP1	1	protein_coding	64
ENSG0000(	TMEM43	1	protein_coding	-797
ENSG0000(	TMEM43	1	protein_coding	686
ENSG0000(	RPS9	1	protein_coding	255
ENSG0000(	NDUFA3	1	protein_coding	-34
ENSG0000(	NDUFA3	1	protein_coding	819
ENSG0000(	OSCAR	1	protein_coding	-1
ENSG0000(	OSCAR	1	protein_coding	-854
ENSG0000(	PAQR8	1	protein_coding	463
ENSG0000(	DNAJC24	1	protein_coding	-55
ENSG0000(	DCDC1	1	protein_coding	26
ENSG0000(	TSNARE1	1	protein_coding	745
ENSG0000(	C11orf24	1	protein_coding	-531
ENSG0000(	MFN1	1	protein_coding	-68
ENSG0000(	ATP6V0E2	1	protein_coding	-124
ENSG0000(	ATP6V0E2	1	protein_coding	676
ENSG0000(	JAGN1	1	protein_coding	207
ENSG0000(	RLN3	1	protein_coding	-66
ENSG0000(	C1GALT1C1	1	protein_coding	428
ENSG0000(	C9orf16	1	protein_coding	-310
ENSG0000(	C9orf16	1	protein_coding	-748
ENSG0000(	C9orf16	1	protein_coding	152
ENSG0000(	C9orf16	1	protein_coding	577
ENSG0000(	ZNF672	1	protein_coding	657
ENSG0000(	ZNF692	1	protein_coding	-316
ENSG0000(	ZNF692	1	protein_coding	501
ENSG0000(	ZNF692	1	protein_coding	935
ENSG0000(	GRIK1	1	protein_coding	-352
ENSG0000(	JUNB	1	protein_coding	67
ENSG0000(	LRG1	1	protein_coding	454
ENSG0000(	FAM98B	1	protein_coding	9
ENSG0000(	CANT1	1	protein_coding	874
ENSG0000(	ZDHHC16	1	protein_coding	-92
ENSG0000(	EXOSC1	1	protein_coding	-21
ENSG0000(	ESCO2	1	protein_coding	712
ENSG0000(	ASXL1	1	protein_coding	-599
ENSG0000(	LRRC8C	1	protein_coding	-303
ENSG0000(	LRRC8C	1	protein_coding	207
ENSG0000(	LRRC8D	1	protein_coding	-486
ENSG0000(	ETFDH	1	protein_coding	28
ENSG0000(	BCL2L1	1	protein_coding	714
ENSG0000(	RAB4B-EGLN2	1	protein_coding	-439
ENSG0000(	RAB4B-EGLN2	1	protein_coding	737
ENSG0000(	CXXC5	1	protein_coding	-457
ENSG0000(	ZNF274	1	protein_coding	-577
ENSG0000(	PLEKHG5	1	protein_coding	801
ENSG0000(	TCEA2	1	protein_coding	305
ENSG0000(	TCEA2	1	protein_coding	714
ENSG0000(	HDAC3	1	protein_coding	258
ENSG0000(	WDR87	1	protein_coding	-347
ENSG0000(	METTL18	1	protein_coding	294
ENSG0000(	COL8A2	1	protein_coding	-760

ENSG0000(	RRM2	1	protein_coding	-319
ENSG0000(	TRAPPC12	1	protein_coding	-950
ENSG0000(	RPS21	1	protein_coding	-969
ENSG0000(	MRM3	1	protein_coding	-897
ENSG0000(	MRM3	1	protein_coding	103
ENSG0000(	RNASEH1	1	protein_coding	21
ENSG0000(	OR52A5	1	protein_coding	409
ENSG0000(	PPIH	1	protein_coding	-159
ENSG0000(	JMJD1C	1	protein_coding	969
ENSG0000(	MOB3A	1	protein_coding	556
ENSG0000(	CALB2	1	protein_coding	-34
ENSG0000(	TEFM	1	protein_coding	551
ENSG0000(	MBOAT1	1	protein_coding	257
ENSG0000(	ZNF131	1	protein_coding	-277
ENSG0000(	DPAGT1	1	protein_coding	-82
ENSG0000(	HINFP	1	protein_coding	-380
ENSG0000(	BPGM	1	protein_coding	439
ENSG0000(	POP7	1	protein_coding	936
ENSG0000(	STARD5	1	protein_coding	-514
ENSG0000(	MCRIP2	1	protein_coding	-268
ENSG0000(	MCRIP2	1	protein_coding	-583
ENSG0000(	C2CD2L	1	protein_coding	1
ENSG0000(	GTPBP2	1	protein_coding	-729
ENSG0000(	MANEA	1	protein_coding	314
ENSG0000(	HCFC1	1	protein_coding	147
ENSG0000(	CHCHD1	1	protein_coding	489
ENSG0000(	SMPDL3A	1	protein_coding	934
ENSG0000(	RAD9A	1	protein_coding	-117
ENSG0000(	LRRC20	1	protein_coding	443
ENSG0000(	MUS81	1	protein_coding	9
ENSG0000(	TMEM217	1	protein_coding	208
ENSG0000(	FAM192A	1	protein_coding	-783
ENSG0000(	RAB37	1	protein_coding	839
ENSG0000(	DCP2	1	protein_coding	-478
ENSG0000(	RPL38	1	protein_coding	116
ENSG0000(	CES2	1	protein_coding	179
ENSG0000(	DMXL1	1	protein_coding	983
ENSG0000(	METAP1D	1	protein_coding	518
ENSG0000(	ZNF621	1	protein_coding	-327
ENSG0000(	ZNF621	1	protein_coding	525
ENSG0000(	NADSYN1	1	protein_coding	321
ENSG0000(	DHCR7	1	protein_coding	-561
ENSG0000(	RNASEH2C	1	protein_coding	135
ENSG0000(	MYEOV	1	protein_coding	312
ENSG0000(	ANKRD13D	1	protein_coding	-172
ENSG0000(	MYD88	1	protein_coding	-992
ENSG0000(	MYD88	1	protein_coding	642
ENSG0000(	OXSR1	1	protein_coding	-539
ENSG0000(	OXSR1	1	protein_coding	62
ENSG0000(	KAT5	1	protein_coding	-862
ENSG0000(	KAT5	1	protein_coding	103
ENSG0000(	DCAKD	1	protein_coding	145
ENSG0000(	GRK2	1	protein_coding	-560
ENSG0000(	FAM222B	1	protein_coding	61

ENSG0000(	FAM222B	1	protein_coding	456
ENSG0000(	RXFP4	1	protein_coding	-798
ENSG0000(	TRMT112	1	protein_coding	-237
ENSG0000(	TRMT112	1	protein_coding	256
ENSG0000(	ADCK5	1	protein_coding	415
ENSG0000(	ESRRA	1	protein_coding	-660
ENSG0000(	COMMD1	1	protein_coding	-28
ENSG0000(	MTX1	1	protein_coding	-310
ENSG0000(	CKS1B	1	protein_coding	-801
ENSG0000(	CKS1B	1	protein_coding	259
ENSG0000(	AHSA2	1	protein_coding	-672
ENSG0000(	ABLIM3	1	protein_coding	-215
ENSG0000(	MFSD4B	1	protein_coding	-104
ENSG0000(	VANGL1	1	protein_coding	-228
ENSG0000(	GPR137	1	protein_coding	238
ENSG0000(	GPR137	1	protein_coding	759
ENSG0000(	ZNF449	1	protein_coding	364
ENSG0000(	ZBTB21	1	protein_coding	367
ENSG0000(	MAP3K11	1	protein_coding	-24
ENSG0000(	TRIB1	1	protein_coding	-92
ENSG0000(	SFT2D3	1	protein_coding	-455
ENSG0000(	SFT2D3	1	protein_coding	-832
ENSG0000(	SFT2D3	1	protein_coding	425
ENSG0000(	ARV1	1	protein_coding	110
ENSG0000(	MINOS1	1	protein_coding	-246
ENSG0000(	RNF26	1	protein_coding	656
ENSG0000(	PPP1R14B	1	protein_coding	-289
ENSG0000(	PPP1R14B	1	protein_coding	699
ENSG0000(	SSSCA1	1	protein_coding	-73
ENSG0000(	SSSCA1	1	protein_coding	-465
ENSG0000(	SMARCC1	1	protein_coding	7
ENSG0000(	SMARCC1	1	protein_coding	533
ENSG0000(	ZNF622	1	protein_coding	51
ENSG0000(	ADGRF3	1	protein_coding	728
ENSG0000(	CEP83	1	protein_coding	-524
ENSG0000(	CEP83	1	protein_coding	454
ENSG0000(	PC	1	protein_coding	-504
ENSG0000(	PC	1	protein_coding	17
ENSG0000(	NMNAT1	1	protein_coding	248
ENSG0000(	LRFN4	1	protein_coding	-648
ENSG0000(	RCE1	1	protein_coding	749
ENSG0000(	TOMM20	1	protein_coding	-150
ENSG0000(	TOMM20	1	protein_coding	435
ENSG0000(	CNP	1	protein_coding	-521
ENSG0000(	ENDOV	1	protein_coding	-65
ENSG0000(	PLK3	1	protein_coding	-429
ENSG0000(	DPY19L1	1	protein_coding	164
ENSG0000(	RBM4B	1	protein_coding	-225
ENSG0000(	RBM4B	1	protein_coding	-590
ENSG0000(	3-Mar	1	protein_coding	432
ENSG0000(	3-Mar	1	protein_coding	669
ENSG0000(	SWSAP1	1	protein_coding	-160
ENSG0000(	RBM4	1	protein_coding	-213
ENSG0000(	RBM4	1	protein_coding	422

ENSG0000(	NRROS	1	protein_coding	-358
ENSG0000(	NRROS	1	protein_coding	680
ENSG0000(	CEP19	1	protein_coding	347
ENSG0000(	FBXO45	1	protein_coding	-116
ENSG0000(	C16orf91	1	protein_coding	57
ENSG0000(	FAM174A	1	protein_coding	-69
ENSG0000(	CYB561D1	1	protein_coding	-260
ENSG0000(	TRMT10C	1	protein_coding	-46
ENSG0000(	CTU2	1	protein_coding	37
ENSG0000(	C12orf66	1	protein_coding	-174
ENSG0000(	C12orf66	1	protein_coding	374
ENSG0000(	PIGG	1	protein_coding	848
ENSG0000(	PITPNA	1	protein_coding	-235
ENSG0000(	ZBTB4	1	protein_coding	-179
ENSG0000(	ZHX3	1	protein_coding	600
ENSG0000(	SLC16A13	1	protein_coding	-301
ENSG0000(	SLC6A19	1	protein_coding	-830
ENSG0000(	EXO1	1	protein_coding	-481
ENSG0000(	EXO1	1	protein_coding	104
ENSG0000(	BBS1	1	protein_coding	-16
ENSG0000(	DENND4A	1	protein_coding	-363
ENSG0000(	KLK15	1	protein_coding	127
ENSG0000(	NPAS4	1	protein_coding	809
ENSG0000(	MSL2	1	protein_coding	600
ENSG0000(	ZNF497	1	protein_coding	652
ENSG0000(	ANGEL2	1	protein_coding	-4
ENSG0000(	ANGEL2	1	protein_coding	-560
ENSG0000(	ZNF266	1	protein_coding	-77
ENSG0000(	FGFBP3	1	protein_coding	383
ENSG0000(	RPL15	1	protein_coding	602
ENSG0000(	C4orf32	1	protein_coding	621
ENSG0000(	THAP6	1	protein_coding	232
ENSG0000(	THAP6	1	protein_coding	807
ENSG0000(	CEP135	1	protein_coding	-100
ENSG0000(	PDE12	1	protein_coding	-205
ENSG0000(	YIF1A	1	protein_coding	-561
ENSG0000(	NDUFA11	1	protein_coding	62
ENSG0000(	CATSPERD	1	protein_coding	-211
ENSG0000(	C19orf70	1	protein_coding	102
ENSG0000(	KCTD13	1	protein_coding	564
ENSG0000(	ATR	1	protein_coding	158
ENSG0000(	PDIK1L	1	protein_coding	-440
ENSG0000(	PDIK1L	1	protein_coding	719
ENSG0000(	SH3BP5L	1	protein_coding	99
ENSG0000(	ABO	1	protein_coding	984
ENSG0000(	PARL	1	protein_coding	-46
ENSG0000(	HIGD2B	1	protein_coding	-560
ENSG0000(	ZNF408	1	protein_coding	-439
ENSG0000(	ZNF408	1	protein_coding	69
ENSG0000(	ARHGAP1	1	protein_coding	-271
ENSG0000(	ARHGAP1	1	protein_coding	237
ENSG0000(	ATG13	1	protein_coding	347
ENSG0000(	APITD1	1	protein_coding	-194
ENSG0000(	APITD1	1	protein_coding	685

ENSG0000(	LSM1	1	protein_coding	456
ENSG0000(	BANF1	1	protein_coding	29
ENSG0000(	TMEM9B	1	protein_coding	-134
ENSG0000(	EIF1AD	1	protein_coding	-907
ENSG0000(	EIF1AD	1	protein_coding	69
ENSG0000(	PCSK1	1	protein_coding	-151
ENSG0000(	SART1	1	protein_coding	-34
ENSG0000(	SART1	1	protein_coding	-315
ENSG0000(	LRRC25	1	protein_coding	-236
ENSG0000(	LRRC25	1	protein_coding	91
ENSG0000(	CLCF1	1	protein_coding	-325
ENSG0000(	UCP3	1	protein_coding	-737
ENSG0000(	UCP2	1	protein_coding	-473
ENSG0000(	UCP2	1	protein_coding	121
ENSG0000(	MRPL48	1	protein_coding	483
ENSG0000(	TMEM70	1	protein_coding	120
ENSG0000(	ZNF77	1	protein_coding	-58
ENSG0000(	B3GNTL1	1	protein_coding	-499
ENSG0000(	AURKAIP1	1	protein_coding	-988
ENSG0000(	AURKAIP1	1	protein_coding	246
ENSG0000(	ZNF169	1	protein_coding	-85
ENSG0000(	ZNF169	1	protein_coding	155
ENSG0000(	SWI5	1	protein_coding	-24
ENSG0000(	SWI5	1	protein_coding	832
ENSG0000(	PLEKHF2	1	protein_coding	-363
ENSG0000(	UNC119B	1	protein_coding	-402
ENSG0000(	LYSMD3	1	protein_coding	-519
ENSG0000(	B3GALT6	1	protein_coding	-738
ENSG0000(	ZNF613	1	protein_coding	25
ENSG0000(	MBLAC2	1	protein_coding	373
ENSG0000(	SLC35A4	1	protein_coding	81
ENSG0000(	SLC35A4	1	protein_coding	339
ENSG0000(	IP6K1	1	protein_coding	-215
ENSG0000(	IP6K1	1	protein_coding	-635
ENSG0000(	IP6K1	1	protein_coding	664
ENSG0000(	CSTF3	1	protein_coding	398
ENSG0000(	YES1	1	protein_coding	-620
ENSG0000(	CHMP6	1	protein_coding	-116
ENSG0000(	UFSP1	1	protein_coding	-717
ENSG0000(	TCP11L1	1	protein_coding	-225
ENSG0000(	CCDC57	1	protein_coding	-666
ENSG0000(	MYPOP	1	protein_coding	-510
ENSG0000(	RTTN	1	protein_coding	-101
ENSG0000(	ZBTB8OS	1	protein_coding	104
ENSG0000(	COX8A	1	protein_coding	98
ENSG0000(	COX8A	1	protein_coding	359
ENSG0000(	B3GNT4	1	protein_coding	-248
ENSG0000(	CDC26	1	protein_coding	101
ENSG0000(	CRLF3	1	protein_coding	-173
ENSG0000(	SPRYD4	1	protein_coding	325
ENSG0000(	SGF29	1	protein_coding	-15
ENSG0000(	LMNB2	1	protein_coding	-616
ENSG0000(	TYMS	1	protein_coding	442
ENSG0000(	TYMS	1	protein_coding	858

ENSG0000(	TCEANC	1	protein_coding	323
ENSG0000(	C8G	1	protein_coding	-238
ENSG0000(	C8G	1	protein_coding	-486
ENSG0000(	SEC24C	1	protein_coding	-373
ENSG0000(	WDR73	1	protein_coding	305
ENSG0000(	RHOG	1	protein_coding	-791
ENSG0000(	CETN1	1	protein_coding	527
ENSG0000(	FAM210A	1	protein_coding	-4
ENSG0000(	CHD9	1	protein_coding	-240
ENSG0000(	PDDC1	1	protein_coding	707
ENSG0000(	FZD8	1	protein_coding	-172
ENSG0000(	FZD8	1	protein_coding	694
ENSG0000(	CASKIN2	1	protein_coding	-367
ENSG0000(	CASKIN2	1	protein_coding	-696
ENSG0000(	CCDC71	1	protein_coding	-441
ENSG0000(	C10orf71	1	protein_coding	-918
ENSG0000(	C10orf71	1	protein_coding	133
ENSG0000(	PPFIA3	1	protein_coding	-870
ENSG0000(	MIEF2	1	protein_coding	58
ENSG0000(	RABEP2	1	protein_coding	290
ENSG0000(	PIDD1	1	protein_coding	140
ENSG0000(	PIDD1	1	protein_coding	840
ENSG0000(	RPLP2	1	protein_coding	-33
ENSG0000(	RPLP2	1	protein_coding	-733
ENSG0000(	CSTF2T	1	protein_coding	-31
ENSG0000(	PGBD5	1	protein_coding	-326
ENSG0000(	ACAD9	1	protein_coding	4
ENSG0000(	ACAD9	1	protein_coding	596
ENSG0000(	THAP5	1	protein_coding	-88
ENSG0000(	DNAJC28	1	protein_coding	-86
ENSG0000(	SOX12	1	protein_coding	-727
ENSG0000(	HNRNPA0	1	protein_coding	-234
ENSG0000(	YIPF7	1	protein_coding	159
ENSG0000(	ZNF518A	1	protein_coding	317
ENSG0000(	TMEM187	1	protein_coding	-666
ENSG0000(	ZNF619	1	protein_coding	-430
ENSG0000(	AP3S1	1	protein_coding	199
ENSG0000(	CENPBD1	1	protein_coding	213
ENSG0000(	BET1L	1	protein_coding	-392
ENSG0000(	RPS27	1	protein_coding	17
ENSG0000(	RIC8A	1	protein_coding	310
ENSG0000(	DMAP1	1	protein_coding	-5
ENSG0000(	IMPDH2	1	protein_coding	46
ENSG0000(	IMPDH2	1	protein_coding	655
ENSG0000(	STAP2	1	protein_coding	-657
ENSG0000(	DALRD3	1	protein_coding	-923
ENSG0000(	LCORL	1	protein_coding	-617
ENSG0000(	SH2B1	1	protein_coding	-249
ENSG0000(	KDELC2	1	protein_coding	504
ENSG0000(	KDELC2	1	protein_coding	907
ENSG0000(	ZNF543	1	protein_coding	-184
ENSG0000(	CTXN1	1	protein_coding	752
ENSG0000(	EPM2AIP1	1	protein_coding	-407
ENSG0000(	EPM2AIP1	1	protein_coding	561

ENSG0000(	CTNNBIP1	1	protein_coding	-459
ENSG0000(	NSUN3	1	protein_coding	425
ENSG0000(	DHFRL1	1	protein_coding	49
ENSG0000(	COX5A	1	protein_coding	-160
ENSG0000(	COX5A	1	protein_coding	761
ENSG0000(	MPI	1	protein_coding	-231
ENSG0000(	TMEM52	1	protein_coding	-553
ENSG0000(	TMEM52	1	protein_coding	914
ENSG0000(	EFCAB13	1	protein_coding	930
ENSG0000(	EXOSC4	1	protein_coding	410
ENSG0000(	PFAS	1	protein_coding	30
ENSG0000(	GAK	1	protein_coding	-476
ENSG0000(	TUFM	1	protein_coding	58
ENSG0000(	CTC1	1	protein_coding	397
ENSG0000(	SEPW1	1	protein_coding	839
ENSG0000(	EIF3K	1	protein_coding	196
ENSG0000(	MRFAP1L1	1	protein_coding	90
ENSG0000(	SNX18	1	protein_coding	127
ENSG0000(	EXD1	1	protein_coding	-134
ENSG0000(	MRFAP1	1	protein_coding	486
ENSG0000(	CYC1	1	protein_coding	743
ENSG0000(	FARSA	1	protein_coding	-91
ENSG0000(	SPTY2D1	1	protein_coding	-172
ENSG0000(	EDC3	1	protein_coding	-336
ENSG0000(	TCAIM	1	protein_coding	-43
ENSG0000(	TCAIM	1	protein_coding	695
ENSG0000(	CALR	1	protein_coding	-502
ENSG0000(	CALR	1	protein_coding	778
ENSG0000(	DAND5	1	protein_coding	139
ENSG0000(	CLK3	1	protein_coding	-142
ENSG0000(	CLK3	1	protein_coding	346
ENSG0000(	ARID3B	1	protein_coding	-607
ENSG0000(	ELMOD2	1	protein_coding	435
ENSG0000(	MKRN3	1	protein_coding	-42
ENSG0000(	LBX2	1	protein_coding	-823
ENSG0000(	ZFPM1	1	protein_coding	-794
ENSG0000(	PLD6	1	protein_coding	-354
ENSG0000(	PLD6	1	protein_coding	285
ENSG0000(	CDC42EP4	1	protein_coding	-452
ENSG0000(	MAF1	1	protein_coding	416
ENSG0000(	SERTAD2	1	protein_coding	-857
ENSG0000(	AC025335.1	1	protein_coding	871
ENSG0000(	SEPHS2	1	protein_coding	142
ENSG0000(	ZNF784	1	protein_coding	-349
ENSG0000(	C14orf119	1	protein_coding	-227
ENSG0000(	C14orf119	1	protein_coding	627
ENSG0000(	BBS10	1	protein_coding	-101
ENSG0000(	BBS10	1	protein_coding	-424
ENSG0000(	PUF60	1	protein_coding	38
ENSG0000(	SSC5D	1	protein_coding	-512
ENSG0000(	DCTPP1	1	protein_coding	-590
ENSG0000(	ZNF771	1	protein_coding	-194
ENSG0000(	ZNF48	1	protein_coding	396
ENSG0000(	TRNAU1AP	1	protein_coding	-224

ENSG0000(	TRNAU1AP	1	protein_coding	577
ENSG0000(	FAHD1	1	protein_coding	245
ENSG0000(	MYLPF	1	protein_coding	29
ENSG0000(	MYLPF	1	protein_coding	772
ENSG0000(	ZNRF2	1	protein_coding	-56
ENSG0000(	OAZ2	1	protein_coding	-269
ENSG0000(	CCDC43	1	protein_coding	-38
ENSG0000(	CCDC43	1	protein_coding	-605
ENSG0000(	ZNF609	1	protein_coding	-491
ENSG0000(	ZNF609	1	protein_coding	856
ENSG0000(	HARBI1	1	protein_coding	36
ENSG0000(	C11orf71	1	protein_coding	144
ENSG0000(	RNF182	1	protein_coding	-91
ENSG0000(	YOD1	1	protein_coding	-569
ENSG0000(	PPA1	1	protein_coding	51
ENSG0000(	SSR4	1	protein_coding	665
ENSG0000(	ZNF792	1	protein_coding	426
ENSG0000(	SCRIB	1	protein_coding	-454
ENSG0000(	CMTR2	1	protein_coding	324
ENSG0000(	FAM83H	1	protein_coding	825
ENSG0000(	LRRC57	1	protein_coding	-74
ENSG0000(	BBS12	1	protein_coding	919
ENSG0000(	DHTKD1	1	protein_coding	-268
ENSG0000(	HIST3H2A	1	protein_coding	-50
ENSG0000(	ZNF746	1	protein_coding	825
ENSG0000(	POLR2A	1	protein_coding	77
ENSG0000(	ZNF678	1	protein_coding	-104
ENSG0000(	SETD2	1	protein_coding	-974
ENSG0000(	MRPS23	1	protein_coding	-15
ENSG0000(	PHLDA2	1	protein_coding	105
ENSG0000(	YIPF6	1	protein_coding	750
ENSG0000(	GPR3	1	protein_coding	-410
ENSG0000(	LSM10	1	protein_coding	-289
ENSG0000(	LSM10	1	protein_coding	-859
ENSG0000(	LSM10	1	protein_coding	131
ENSG0000(	LSM10	1	protein_coding	502
ENSG0000(	RNF41	1	protein_coding	-470
ENSG0000(	IBA57	1	protein_coding	-503
ENSG0000(	IBA57	1	protein_coding	67
ENSG0000(	IBA57	1	protein_coding	955
ENSG0000(	C5orf24	1	protein_coding	-189
ENSG0000(	PNMAL1	1	protein_coding	496
ENSG0000(	USH1G	1	protein_coding	-997
ENSG0000(	NOP10	1	protein_coding	1
ENSG0000(	ERCC6L2	1	protein_coding	431
ENSG0000(	CREB3L2	1	protein_coding	135
ENSG0000(	TSEN54	1	protein_coding	-109
ENSG0000(	TSEN54	1	protein_coding	220
ENSG0000(	MRPS16	1	protein_coding	611
ENSG0000(	ARL6IP4	1	protein_coding	-73
ENSG0000(	ARL6IP4	1	protein_coding	166
ENSG0000(	ZSCAN22	1	protein_coding	135
ENSG0000(	KBTBD3	1	protein_coding	0
ENSG0000(	KBTBD3	1	protein_coding	991

ENSG0000(	YBEY	1	protein_coding	-57
ENSG0000(	CLN8	1	protein_coding	302
ENSG0000(	IFNL1	1	protein_coding	425
ENSG0000(	PGBD4	1	protein_coding	-78
ENSG0000(	MFSD5	1	protein_coding	185
ENSG0000(	SKA2	1	protein_coding	309
ENSG0000(	TTC3	1	protein_coding	-798
ENSG0000(	TTC3	1	protein_coding	538
ENSG0000(	PAQR7	1	protein_coding	-242
ENSG0000(	C1orf116	1	protein_coding	-43
ENSG0000(	DDX28	1	protein_coding	-7
ENSG0000(	ADGRG3	1	protein_coding	-18
ENSG0000(	ADGRG3	1	protein_coding	790
ENSG0000(	RPL35A	1	protein_coding	297
ENSG0000(	ZNF721	1	protein_coding	-901
ENSG0000(	C11orf54	1	protein_coding	-595
ENSG0000(	OTOP2	1	protein_coding	-21
ENSG0000(	SLC25A10	1	protein_coding	-328
ENSG0000(	SLC25A10	1	protein_coding	631
ENSG0000(	ARHGEF37	1	protein_coding	-245
ENSG0000(	CEP57L1	1	protein_coding	372
ENSG0000(	GPR19	1	protein_coding	89
ENSG0000(	RABIF	1	protein_coding	-106
ENSG0000(	SMDT1	1	protein_coding	100
ENSG0000(	RUVBL2	1	protein_coding	224
ENSG0000(	RUVBL2	1	protein_coding	810
ENSG0000(	GDPGP1	1	protein_coding	-82
ENSG0000(	GDPGP1	1	protein_coding	-478
ENSG0000(	PTTG1IP	1	protein_coding	-439
ENSG0000(	DDX41	1	protein_coding	-6
ENSG0000(	DAZAP2	1	protein_coding	618
ENSG0000(	15-Sep	1	protein_coding	268
ENSG0000(	FHL3	1	protein_coding	818
ENSG0000(	TREX2	1	protein_coding	553
ENSG0000(	EP400	1	protein_coding	-333
ENSG0000(	EP400	1	protein_coding	702
ENSG0000(	FAM46C	1	protein_coding	-695
ENSG0000(	COA5	1	protein_coding	51
ENSG0000(	UTP11	1	protein_coding	-11
ENSG0000(	TANGO2	1	protein_coding	453
ENSG0000(	MRPL54	1	protein_coding	-813
ENSG0000(	MRPL54	1	protein_coding	326
ENSG0000(	ZNF438	1	protein_coding	-317
ENSG0000(	TRMT12	1	protein_coding	-33
ENSG0000(	TRIM52	1	protein_coding	192
ENSG0000(	TBL3	1	protein_coding	-109
ENSG0000(	CHEK2	1	protein_coding	98
ENSG0000(	KCTD16	1	protein_coding	-212
ENSG0000(	RBM12B	1	protein_coding	616
ENSG0000(	NPW	1	protein_coding	223
ENSG0000(	PTP4A2	1	protein_coding	-128
ENSG0000(	PTP4A2	1	protein_coding	504
ENSG0000(	DSCR4	1	protein_coding	-826
ENSG0000(	DSCR4	1	protein_coding	107

ENSG0000(	DIABLO	1	protein_coding	910
ENSG0000(	VPS33B	1	protein_coding	-36
ENSG0000(	TBX1	1	protein_coding	-148
ENSG0000(	ADAP2	1	protein_coding	-74
ENSG0000(	CLDN5	1	protein_coding	-235
ENSG0000(	CLDN5	1	protein_coding	958
ENSG0000(	NR2C2AP	1	protein_coding	369
ENSG0000(	SCFD2	1	protein_coding	403
ENSG0000(	PGP	1	protein_coding	-630
ENSG0000(	C22orf46	1	protein_coding	-722
ENSG0000(	SNRNP35	1	protein_coding	810
ENSG0000(	IRAK1	1	protein_coding	-655
ENSG0000(	HIST2H2AC	1	protein_coding	-193
ENSG0000(	KCNK12	1	protein_coding	-540
ENSG0000(	TSSC4	1	protein_coding	-154
ENSG0000(	TSSC4	1	protein_coding	216
ENSG0000(	KNTC1	1	protein_coding	148
ENSG0000(	WDR27	1	protein_coding	18
ENSG0000(	TXNRD2	1	protein_coding	-14
ENSG0000(	PTP4A3	1	protein_coding	-187
ENSG0000(	PTP4A3	1	protein_coding	-517
ENSG0000(	NUTM1	1	protein_coding	-138
ENSG0000(	HDDC3	1	protein_coding	438
ENSG0000(	ZFP1	1	protein_coding	-28
ENSG0000(	SOCS3	1	protein_coding	-880
ENSG0000(	LPAR5	1	protein_coding	-333
ENSG0000(	LPAR5	1	protein_coding	844
ENSG0000(	XPOT	1	protein_coding	-219
ENSG0000(	XPOT	1	protein_coding	798
ENSG0000(	TMEM173	1	protein_coding	282
ENSG0000(	9-Sep	1	protein_coding	-562
ENSG0000(	CDCA2	1	protein_coding	-1
ENSG0000(	ZBTB40	1	protein_coding	112
ENSG0000(	HIST2H2BE	1	protein_coding	-129
ENSG0000(	CLDN6	1	protein_coding	-37
ENSG0000(	CLDN6	1	protein_coding	-552
ENSG0000(	5-Sep	1	protein_coding	-590
ENSG0000(	5-Sep	1	protein_coding	698
ENSG0000(	EIF4ENIF1	1	protein_coding	-253
ENSG0000(	SERINC4	1	protein_coding	-85
ENSG0000(	SERINC4	1	protein_coding	-769
ENSG0000(	ATL3	1	protein_coding	233
ENSG0000(	TCTE3	1	protein_coding	-84
ENSG0000(	TMEM186	1	protein_coding	-211
ENSG0000(	TMEM186	1	protein_coding	-695
ENSG0000(	RBM33	1	protein_coding	-131
ENSG0000(	RBM43	1	protein_coding	519
ENSG0000(	RBM43	1	protein_coding	937
ENSG0000(	SUMO3	1	protein_coding	-373
ENSG0000(	IMMP2L	1	protein_coding	-139
ENSG0000(	PTRHD1	1	protein_coding	73
ENSG0000(	PTRHD1	1	protein_coding	574
ENSG0000(	NDUFA6	1	protein_coding	463
ENSG0000(	DGAT1	1	protein_coding	124

ENSG0000(	AP3M1	1	protein_coding	-283
ENSG0000(	AP3M1	1	protein_coding	183
ENSG0000(	UBOX5	1	protein_coding	298
ENSG0000(	UBOX5	1	protein_coding	598
ENSG0000(	CIB1	1	protein_coding	322
ENSG0000(	CIB1	1	protein_coding	718
ENSG0000(	C5orf47	1	protein_coding	-326
ENSG0000(	FAF1	1	protein_coding	-271
ENSG0000(	C6orf120	1	protein_coding	-159
ENSG0000(	PURA	1	protein_coding	-179
ENSG0000(	TNFAIP2	1	protein_coding	574
ENSG0000(	PGBD2	1	protein_coding	194
ENSG0000(	ZNF74	1	protein_coding	-147
ENSG0000(	ZBTB37	1	protein_coding	-157
ENSG0000(	CCDC137	1	protein_coding	-535
ENSG0000(	CCDC137	1	protein_coding	369
ENSG0000(	ARL15	1	protein_coding	-248
ENSG0000(	ARL15	1	protein_coding	908
ENSG0000(	TMEM105	1	protein_coding	310
ENSG0000(	PARK2	1	protein_coding	-105
ENSG0000(	MAPK11	1	protein_coding	-172
ENSG0000(	MRPL30	1	protein_coding	111
ENSG0000(	SH3BGR	1	protein_coding	-47
ENSG0000(	FAM174B	1	protein_coding	-378
ENSG0000(	FAM174B	1	protein_coding	146
ENSG0000(	FAM174B	1	protein_coding	581
ENSG0000(	TMEM179B	1	protein_coding	259
ENSG0000(	MUC1	1	protein_coding	-376
ENSG0000(	MUC1	1	protein_coding	-975
ENSG0000(	FAAP100	1	protein_coding	238
ENSG0000(	SP1	1	protein_coding	-699
ENSG0000(	MRPL40	1	protein_coding	403
ENSG0000(	FAM212A	1	protein_coding	-461
ENSG0000(	PCGF3	1	protein_coding	-381
ENSG0000(	LMLN	1	protein_coding	535
ENSG0000(	SNAI3	1	protein_coding	-261
ENSG0000(	SNAI3	1	protein_coding	-540
ENSG0000(	ZBTB3	1	protein_coding	14
ENSG0000(	EP400NL	1	protein_coding	780
ENSG0000(	PRAME	1	protein_coding	-358
ENSG0000(	DRG1	1	protein_coding	632
ENSG0000(	ZNF696	1	protein_coding	-512
ENSG0000(	ZNF696	1	protein_coding	186
ENSG0000(	WDR53	1	protein_coding	180
ENSG0000(	PIGP	1	protein_coding	-593
ENSG0000(	PIGP	1	protein_coding	743
ENSG0000(	GNB1L	1	protein_coding	534
ENSG0000(	THNSL1	1	protein_coding	24
ENSG0000(	IFITM1	1	protein_coding	572
ENSG0000(	KLHDC8B	1	protein_coding	-754
ENSG0000(	SETD4	1	protein_coding	-143
ENSG0000(	NDUFA13	1	protein_coding	976
ENSG0000(	ZNF566	1	protein_coding	10
ENSG0000(	ZNF566	1	protein_coding	581

ENSG0000(	ZNF284	1	protein_coding	-57
ENSG0000(	AIDA	1	protein_coding	198
ENSG0000(	C15orf41	1	protein_coding	390
ENSG0000(	POLR3C	1	protein_coding	354
ENSG0000(	WWOX	1	protein_coding	-175
ENSG0000(	CCDC84	1	protein_coding	-413
ENSG0000(	BCL9L	1	protein_coding	906
ENSG0000(	KIF18B	1	protein_coding	-362
ENSG0000(	ZNRF1	1	protein_coding	-408
ENSG0000(	BLOC1S4	1	protein_coding	-68
ENSG0000(	ZNF749	1	protein_coding	-16
ENSG0000(	KLHL32	1	protein_coding	347
ENSG0000(	ZNF17	1	protein_coding	-8
ENSG0000(	KDM4D	1	protein_coding	-166
ENSG0000(	TOR3A	1	protein_coding	443
ENSG0000(	PRELID2	1	protein_coding	-236
ENSG0000(	PRELID2	1	protein_coding	-765
ENSG0000(	NUDT17	1	protein_coding	114
ENSG0000(	NUDT17	1	protein_coding	826
ENSG0000(	TMEM222	1	protein_coding	-59
ENSG0000(	TMEM222	1	protein_coding	468
ENSG0000(	FOXD2	1	protein_coding	691
ENSG0000(	GPATCH8	1	protein_coding	-336
ENSG0000(	NF2	1	protein_coding	-380
ENSG0000(	C6orf1	1	protein_coding	-541
ENSG0000(	UBE2H	1	protein_coding	-394
ENSG0000(	KATNA1	1	protein_coding	243
ENSG0000(	ARAP1	1	protein_coding	-33
ENSG0000(	ZFP91	1	protein_coding	705
ENSG0000(	BCR	1	protein_coding	-263
ENSG0000(	HYAL3	1	protein_coding	-810
ENSG0000(	HYAL3	1	protein_coding	228
ENSG0000(	TPCN1	1	protein_coding	-420
ENSG0000(	HEXIM1	1	protein_coding	207
ENSG0000(	POFUT2	1	protein_coding	-128
ENSG0000(	ERCC6L	1	protein_coding	-224
ENSG0000(	C1QL4	1	protein_coding	187
ENSG0000(	RTN4RL2	1	protein_coding	-820
ENSG0000(	ZACN	1	protein_coding	755
ENSG0000(	KANK3	1	protein_coding	-375
ENSG0000(	ESPN	1	protein_coding	477
ENSG0000(	CMC1	1	protein_coding	2
ENSG0000(	CHP1	1	protein_coding	39
ENSG0000(	IFT140	1	protein_coding	149
ENSG0000(	TLR5	1	protein_coding	414
ENSG0000(	NANOS3	1	protein_coding	-321
ENSG0000(	NANOS3	1	protein_coding	384
ENSG0000(	ISG15	1	protein_coding	-175
ENSG0000(	VMAC	1	protein_coding	-913
ENSG0000(	VMAC	1	protein_coding	271
ENSG0000(	TCEA1	1	protein_coding	-960
ENSG0000(	NHEJ1	1	protein_coding	651
ENSG0000(	MCRS1	1	protein_coding	-77
ENSG0000(	CARD9	1	protein_coding	552

ENSG0000(	ZFP69B	1	protein_coding	-108
ENSG0000(	ZFP69B	1	protein_coding	557
ENSG0000(	PALM3	1	protein_coding	353
ENSG0000(	LRRC74B	1	protein_coding	-269
ENSG0000(	C17orf99	1	protein_coding	874
ENSG0000(	PRSS45	1	protein_coding	575
ENSG0000(	MESP2	1	protein_coding	354
ENSG0000(	TMPPE	1	protein_coding	-637
ENSG0000(	ZC3H6	1	protein_coding	-280
ENSG0000(	LAMTOR4	1	protein_coding	43
ENSG0000(	HES4	1	protein_coding	-790
ENSG0000(	C19orf35	1	protein_coding	-69
ENSG0000(	PRR19	1	protein_coding	-253
ENSG0000(	ZP3	1	protein_coding	223
ENSG0000(	BLOC1S5	1	protein_coding	-204
ENSG0000(	H2AFX	1	protein_coding	17
ENSG0000(	SRSF10	1	protein_coding	243
ENSG0000(	DUSP28	1	protein_coding	257
ENSG0000(	SUMO2	1	protein_coding	-346
ENSG0000(	RHCE	1	protein_coding	315
ENSG0000(	VWC2	1	protein_coding	-504
ENSG0000(	MTF1	1	protein_coding	-475
ENSG0000(	MTF1	1	protein_coding	77
ENSG0000(	ZNF563	1	protein_coding	-138
ENSG0000(	KLRG2	1	protein_coding	-425
ENSG0000(	TRMT2B	1	protein_coding	424
ENSG0000(	UTS2B	1	protein_coding	280
ENSG0000(	NELFB	1	protein_coding	401
ENSG0000(	KCTD21	1	protein_coding	464
ENSG0000(	ADAT2	1	protein_coding	-81
ENSG0000(	NDUFA4	1	protein_coding	33
ENSG0000(	ALKBH2	1	protein_coding	20
ENSG0000(	SF3B3	1	protein_coding	426
ENSG0000(	CLDN4	1	protein_coding	172
ENSG0000(	ZAR1L	1	protein_coding	-175
ENSG0000(	TSPYL1	1	protein_coding	136
ENSG0000(	BLOC1S2	1	protein_coding	-82
ENSG0000(	KIAA0895L	1	protein_coding	-21
ENSG0000(	KIAA0895L	1	protein_coding	473
ENSG0000(	ZNF250	1	protein_coding	131
ENSG0000(	WDSUB1	1	protein_coding	-217
ENSG0000(	WDSUB1	1	protein_coding	-652
ENSG0000(	ZNF79	1	protein_coding	-206
ENSG0000(	TMEM63A	1	protein_coding	849
ENSG0000(	HRCT1	1	protein_coding	-250
ENSG0000(	RYR1	1	protein_coding	486
ENSG0000(	SRGAP3	1	protein_coding	-341
ENSG0000(	SRGAP3	1	protein_coding	200
ENSG0000(	TUBB	1	protein_coding	-369
ENSG0000(	XPNPEP3	1	protein_coding	-127
ENSG0000(	SFTA2	1	protein_coding	-202
ENSG0000(	NIF3L1	1	protein_coding	316
ENSG0000(	IARS	1	protein_coding	-171
ENSG0000(	CD55	1	protein_coding	-316

ENSG0000(	LONP1	1	protein_coding	107
ENSG0000(	ASB13	1	protein_coding	-328
ENSG0000(	ZNF34	1	protein_coding	202
ENSG0000(	ZNF140	1	protein_coding	741
ENSG0000(	ZNF774	1	protein_coding	-71
ENSG0000(	XRCC6	1	protein_coding	-466
ENSG0000(	ZNF775	1	protein_coding	-173
ENSG0000(	ZNF775	1	protein_coding	-696
ENSG0000(	ZNF775	1	protein_coding	753
ENSG0000(	C20orf96	1	protein_coding	9
ENSG0000(	ANAPC7	1	protein_coding	-481
ENSG0000(	TPK1	1	protein_coding	179
ENSG0000(	SLC6A9	1	protein_coding	-659
ENSG0000(	MYO18A	1	protein_coding	-21
ENSG0000(	MAN2A2	1	protein_coding	132
ENSG0000(	XRCC2	1	protein_coding	67
ENSG0000(	XRCC2	1	protein_coding	623
ENSG0000(	SDHAF3	1	protein_coding	958
ENSG0000(	ZKSCAN5	1	protein_coding	71
ENSG0000(	TRAPPC4	1	protein_coding	22
ENSG0000(	TRAPPC4	1	protein_coding	814
ENSG0000(	TTC30B	1	protein_coding	62
ENSG0000(	ZNF33B	1	protein_coding	-331
ENSG0000(	ZNF33B	1	protein_coding	481
ENSG0000(	S100A2	1	protein_coding	837
ENSG0000(	HIST1H2AG	1	protein_coding	-110
ENSG0000(	C6orf106	1	protein_coding	-441
ENSG0000(	PPTC7	1	protein_coding	666
ENSG0000(	HIST3H2BB	1	protein_coding	-183
ENSG0000(	PDLIM7	1	protein_coding	-877
ENSG0000(	AP2A1	1	protein_coding	-785
ENSG0000(	FUT11	1	protein_coding	-45
ENSG0000(	LAGE3	1	protein_coding	-26
ENSG0000(	WDR45	1	protein_coding	-371
ENSG0000(	SERTAD1	1	protein_coding	-237
ENSG0000(	SERTAD1	1	protein_coding	937
ENSG0000(	ZNF398	1	protein_coding	-44
ENSG0000(	ZNF441	1	protein_coding	-193
ENSG0000(	HIST1H4C	1	protein_coding	325
ENSG0000(	MAFG	1	protein_coding	-790
ENSG0000(	GAL3ST4	1	protein_coding	335
ENSG0000(	DYNC1H1	1	protein_coding	-682
ENSG0000(	ZNF772	1	protein_coding	-104
ENSG0000(	PCNX3	1	protein_coding	-366
ENSG0000(	LRRC8B	1	protein_coding	-195
ENSG0000(	LRRC8B	1	protein_coding	-601
ENSG0000(	ABCB8	1	protein_coding	124
ENSG0000(	HIST1H3J	1	protein_coding	-200
ENSG0000(	SND1	1	protein_coding	-609
ENSG0000(	SND1	1	protein_coding	605
ENSG0000(	ZNF785	1	protein_coding	-103
ENSG0000(	PSMD12	1	protein_coding	501
ENSG0000(	ENTPD4	1	protein_coding	-139
ENSG0000(	KANK2	1	protein_coding	-317

ENSG0000(	RAD54B	1	protein_coding	-217
ENSG0000(	ZNF165	1	protein_coding	89
ENSG0000(	SYNGAP1	1	protein_coding	-883
ENSG0000(	FITM2	1	protein_coding	-148
ENSG0000(	BLM	1	protein_coding	-237
ENSG0000(	DDI2	1	protein_coding	-398
ENSG0000(	DDI2	1	protein_coding	119
ENSG0000(	DDI2	1	protein_coding	594
ENSG0000(	MRPL21	1	protein_coding	17
ENSG0000(	ZNF786	1	protein_coding	23
ENSG0000(	ZNF786	1	protein_coding	482
ENSG0000(	ZNF517	1	protein_coding	409
ENSG0000(	IPP	1	protein_coding	-298
ENSG0000(	IPP	1	protein_coding	296
ENSG0000(	MAP3K5	1	protein_coding	-263
ENSG0000(	MAP3K5	1	protein_coding	773
ENSG0000(	STMN3	1	protein_coding	-598
ENSG0000(	STMN3	1	protein_coding	-935
ENSG0000(	STMN3	1	protein_coding	68
ENSG0000(	SPN	1	protein_coding	619
ENSG0000(	ZNF628	1	protein_coding	-73
ENSG0000(	ZNF628	1	protein_coding	677
ENSG0000(	RPF2	1	protein_coding	-1
ENSG0000(	C5orf56	1	protein_coding	-344
ENSG0000(	C5orf56	1	protein_coding	424
ENSG0000(	C5orf56	1	protein_coding	788
ENSG0000(	ATG7	1	protein_coding	-188
ENSG0000(	TTC30A	1	protein_coding	28
ENSG0000(	RAB40C	1	protein_coding	-620
ENSG0000(	HHLA3	1	protein_coding	141
ENSG0000(	CCDC154	1	protein_coding	-180
ENSG0000(	CDC42SE1	1	protein_coding	-120
ENSG0000(	ZNF460	1	protein_coding	427
ENSG0000(	RPS26	1	protein_coding	164
ENSG0000(	PSAP	1	protein_coding	116
ENSG0000(	LHFPL5	1	protein_coding	222
ENSG0000(	MCMBP	1	protein_coding	-37
ENSG0000(	MCMBP	1	protein_coding	-213
ENSG0000(	EME2	1	protein_coding	-901
ENSG0000(	EME2	1	protein_coding	306
ENSG0000(	TAF13	1	protein_coding	-100
ENSG0000(	ATAD3A	1	protein_coding	-194
ENSG0000(	ZNF461	1	protein_coding	-82
ENSG0000(	C4orf22	1	protein_coding	346
ENSG0000(	ZNF181	1	protein_coding	-246
ENSG0000(	FAM212B	1	protein_coding	6
ENSG0000(	NKIRAS1	1	protein_coding	304
ENSG0000(	MEIG1	1	protein_coding	-275
ENSG0000(	ADH5	1	protein_coding	-350
ENSG0000(	HIST1H2BK	1	protein_coding	-253
ENSG0000(	HIST1H2BK	1	protein_coding	-854
ENSG0000(	SPG7	1	protein_coding	-96
ENSG0000(	ZNF311	1	protein_coding	-255
ENSG0000(	PLCG2	1	protein_coding	-868

ENSG0000(	RPL12	1	protein_coding	322
ENSG0000(	LEKR1	1	protein_coding	684
ENSG0000(	KEL	1	protein_coding	783
ENSG0000(	IRAK4	1	protein_coding	-306
ENSG0000(	CCDC151	1	protein_coding	603
ENSG0000(	MRPL42	1	protein_coding	-87
ENSG0000(	MRPL42	1	protein_coding	259
ENSG0000(	ENTPD7	1	protein_coding	-613
ENSG0000(	ZNF335	1	protein_coding	-82
ENSG0000(	MAK16	1	protein_coding	400
ENSG0000(	DSCR8	1	protein_coding	-197
ENSG0000(	DSCR8	1	protein_coding	736
ENSG0000(	5-Mar	1	protein_coding	-759
ENSG0000(	ZBTB14	1	protein_coding	232
ENSG0000(	TMPRSS11F	1	protein_coding	-201
ENSG0000(	TMPRSS11F	1	protein_coding	97
ENSG0000(	ZNF248	1	protein_coding	340
ENSG0000(	TOR4A	1	protein_coding	-322
ENSG0000(	ZNF770	1	protein_coding	-93
ENSG0000(	MIER1	1	protein_coding	-204
ENSG0000(	MIER1	1	protein_coding	698
ENSG0000(	DDRGK1	1	protein_coding	281
ENSG0000(	TFDP1	1	protein_coding	-378
ENSG0000(	SZT2	1	protein_coding	290
ENSG0000(	RP11-566K11.2	1	protein_coding	-332
ENSG0000(	RP11-566K11.2	1	protein_coding	-861
ENSG0000(	RP11-566K11.2	1	protein_coding	46
ENSG0000(	DDX42	1	protein_coding	-243
ENSG0000(	RPL23A	1	protein_coding	673
ENSG0000(	SLC29A3	1	protein_coding	429
ENSG0000(	UBL5	1	protein_coding	-61
ENSG0000(	HELZ	1	protein_coding	-922
ENSG0000(	TMEM116	1	protein_coding	263
ENSG0000(	UCKL1	1	protein_coding	-244
ENSG0000(	ZNF485	1	protein_coding	-28
ENSG0000(	ZKSCAN8	1	protein_coding	-99
ENSG0000(	UVRAG	1	protein_coding	-356
ENSG0000(	TCAF1	1	protein_coding	629
ENSG0000(	TPM2	1	protein_coding	-406
ENSG0000(	CNGA1	1	protein_coding	-344
ENSG0000(	ZNF789	1	protein_coding	36
ENSG0000(	DDX39B	1	protein_coding	504
ENSG0000(	LRBA	1	protein_coding	-871
ENSG0000(	LRBA	1	protein_coding	137
ENSG0000(	TTC37	1	protein_coding	-348
ENSG0000(	ANKRD13B	1	protein_coding	307
ENSG0000(	ECI2	1	protein_coding	-951
ENSG0000(	CTR9	1	protein_coding	94
ENSG0000(	CTR9	1	protein_coding	518
ENSG0000(	SLC5A3	1	protein_coding	-453
ENSG0000(	GPATCH3	1	protein_coding	-129
ENSG0000(	PLXNB3	1	protein_coding	244
ENSG0000(	RPL10A	1	protein_coding	-519
ENSG0000(	RPL10A	1	protein_coding	92

ENSG0000(	COLGALT2	1	protein_coding	-144
ENSG0000(	EPS8L3	1	protein_coding	72
ENSG0000(	EPS8L3	1	protein_coding	943
ENSG0000(	RCSD1	1	protein_coding	-449
ENSG0000(	ZNF830	1	protein_coding	-26
ENSG0000(	FOXJ3	1	protein_coding	579
ENSG0000(	HMGN2	1	protein_coding	-597
ENSG0000(	OPA1	1	protein_coding	-165
ENSG0000(	OPA1	1	protein_coding	657
ENSG0000(	DENND4B	1	protein_coding	-139
ENSG0000(	DENND4B	1	protein_coding	970
ENSG0000(	ZNF277	1	protein_coding	-15
ENSG0000(	KTI12	1	protein_coding	84
ENSG0000(	SELT	1	protein_coding	281
ENSG0000(	TSEN15	1	protein_coding	390
ENSG0000(	LTN1	1	protein_coding	18
ENSG0000(	RUNDC1	1	protein_coding	-66
ENSG0000(	RUNDC1	1	protein_coding	444
ENSG0000(	PRC1	1	protein_coding	432
ENSG0000(	L1CAM	1	protein_coding	47
ENSG0000(	C1orf174	1	protein_coding	-418
ENSG0000(	C1orf174	1	protein_coding	426
ENSG0000(	C9orf114	1	protein_coding	-82
ENSG0000(	CCDC167	1	protein_coding	-261
ENSG0000(	NAGA	1	protein_coding	-416
ENSG0000(	SMG5	1	protein_coding	-222
ENSG0000(	SMG5	1	protein_coding	981
ENSG0000(	KIF1BP	1	protein_coding	53
ENSG0000(	KIF1BP	1	protein_coding	400
ENSG0000(	KIF1BP	1	protein_coding	719
ENSG0000(	TGM2	1	protein_coding	843
ENSG0000(	PJA2	1	protein_coding	-503
ENSG0000(	SGMS1	1	protein_coding	-358
ENSG0000(	COX20	1	protein_coding	-495
ENSG0000(	COX20	1	protein_coding	704
ENSG0000(	CHML	1	protein_coding	-451
ENSG0000(	TATDN3	1	protein_coding	37
ENSG0000(	CENPW	1	protein_coding	147
ENSG0000(	SOX18	1	protein_coding	-499
ENSG0000(	SOX18	1	protein_coding	-908
ENSG0000(	EFCAB7	1	protein_coding	223
ENSG0000(	ZYG11A	1	protein_coding	704
ENSG0000(	SYS1	1	protein_coding	928
ENSG0000(	INPP5B	1	protein_coding	107
ENSG0000(	PHACTR4	1	protein_coding	-93
ENSG0000(	TMEM57	1	protein_coding	-980
ENSG0000(	PFDN6	1	protein_coding	146
ENSG0000(	RXRB	1	protein_coding	-126
ENSG0000(	RXRB	1	protein_coding	-878
ENSG0000(	RXRB	1	protein_coding	768
ENSG0000(	OXLD1	1	protein_coding	-97
ENSG0000(	OXLD1	1	protein_coding	807
ENSG0000(	BRD2	1	protein_coding	-353
ENSG0000(	BRD2	1	protein_coding	870

ENSG0000(	HLA-DMA	1	protein_coding	-435
ENSG0000(	HLA-DMA	1	protein_coding	-884
ENSG0000(	HLA-DMA	1	protein_coding	788
ENSG0000(	RNF5	1	protein_coding	-402
ENSG0000(	AGPAT1	1	protein_coding	145
ENSG0000(	PRRT1	1	protein_coding	-185
ENSG0000(	PRRT1	1	protein_coding	-799
ENSG0000(	SMIM5	1	protein_coding	627
ENSG0000(	STK19	1	protein_coding	904
ENSG0000(	DXO	1	protein_coding	-413
ENSG0000(	DXO	1	protein_coding	-819
ENSG0000(	DXO	1	protein_coding	298
ENSG0000(	SKIV2L	1	protein_coding	25
ENSG0000(	NELFE	1	protein_coding	6
ENSG0000(	ZBTB12	1	protein_coding	-616
ENSG0000(	SDHD	1	protein_coding	-286
ENSG0000(	SDHD	1	protein_coding	19
ENSG0000(	EHMT2	1	protein_coding	-123
ENSG0000(	NEU1	1	protein_coding	-863
ENSG0000(	NEU1	1	protein_coding	461
ENSG0000(	C6orf48	1	protein_coding	297
ENSG0000(	HSPA1B	1	protein_coding	-720
ENSG0000(	LSM2	1	protein_coding	75
ENSG0000(	LSM2	1	protein_coding	742
ENSG0000(	VARS	1	protein_coding	1
ENSG0000(	VWA7	1	protein_coding	-61
ENSG0000(	MBD5	1	protein_coding	-533
ENSG0000(	MSH5	1	protein_coding	-437
ENSG0000(	MSH5	1	protein_coding	247
ENSG0000(	C6orf25	1	protein_coding	-846
ENSG0000(	LY6G6F	1	protein_coding	10
ENSG0000(	ABHD16A	1	protein_coding	934
ENSG0000(	LY6G5C	1	protein_coding	224
ENSG0000(	CSNK2B	1	protein_coding	-192
ENSG0000(	C6orf47	1	protein_coding	679
ENSG0000(	APOM	1	protein_coding	618
ENSG0000(	BAG6	1	protein_coding	-328
ENSG0000(	NCR3	1	protein_coding	438
ENSG0000(	LST1	1	protein_coding	-364
ENSG0000(	LST1	1	protein_coding	-611
ENSG0000(	NFKBIL1	1	protein_coding	-131
ENSG0000(	MICA	1	protein_coding	-8
ENSG0000(	POU5F1	1	protein_coding	-47
ENSG0000(	CCHCR1	1	protein_coding	-360
ENSG0000(	CCHCR1	1	protein_coding	-966
ENSG0000(	C6orf136	1	protein_coding	-199
ENSG0000(	MRPS18B	1	protein_coding	-329
ENSG0000(	TRIM39	1	protein_coding	-183
ENSG0000(	AKT1S1	1	protein_coding	986
ENSG0000(	SPDYC	1	protein_coding	-788
ENSG0000(	SPDYC	1	protein_coding	46
ENSG0000(	MRPL53	1	protein_coding	593
ENSG0000(	MROH6	1	protein_coding	-324
ENSG0000(	FAM216A	1	protein_coding	142

ENSG0000(	ZBTB48	1	protein_coding	837
ENSG0000(	UQCC3	1	protein_coding	-130
ENSG0000(	UQCC3	1	protein_coding	-639
ENSG0000(	ZNF425	1	protein_coding	-25
ENSG0000(	C12orf73	1	protein_coding	147
ENSG0000(	SPIRE2	1	protein_coding	-480
ENSG0000(	SDHAF1	1	protein_coding	1
ENSG0000(	C4orf46	1	protein_coding	103
ENSG0000(	PSMB10	1	protein_coding	-47
ENSG0000(	E2F4	1	protein_coding	572
ENSG0000(	PDE7A	1	protein_coding	29
ENSG0000(	TMSB4X	1	protein_coding	-212
ENSG0000(	HMGN1	1	protein_coding	6
ENSG0000(	CDPF1	1	protein_coding	-531
ENSG0000(	SMIM11A	1	protein_coding	-335
ENSG0000(	SMIM11A	1	protein_coding	330
ENSG0000(	ITSN1	1	protein_coding	-329
ENSG0000(	ITSN1	1	protein_coding	-695
ENSG0000(	PLPP6	1	protein_coding	660
ENSG0000(	RNPS1	1	protein_coding	52
ENSG0000(	HN1L	1	protein_coding	935
ENSG0000(	TSSK2	1	protein_coding	479
ENSG0000(	HACD2	1	protein_coding	-165
ENSG0000(	PRSS50	1	protein_coding	591
ENSG0000(	DIO1	1	protein_coding	-857
ENSG0000(	ZNF611	1	protein_coding	271
ENSG0000(	NUP62	1	protein_coding	-985
ENSG0000(	NUP62	1	protein_coding	93
ENSG0000(	NUP62	1	protein_coding	684
ENSG0000(	DENND1B	1	protein_coding	-117
ENSG0000(	SFT2D2	1	protein_coding	-183
ENSG0000(	SFT2D2	1	protein_coding	206
ENSG0000(	SCAF8	1	protein_coding	-422
ENSG0000(	KLHL23	1	protein_coding	-103
ENSG0000(	KLHL23	1	protein_coding	636
ENSG0000(	MLLT11	1	protein_coding	215
ENSG0000(	DNLZ	1	protein_coding	2
ENSG0000(	SUPT4H1	1	protein_coding	182
ENSG0000(	ANKRD39	1	protein_coding	-75
ENSG0000(	QTRT1	1	protein_coding	717
ENSG0000(	MXD3	1	protein_coding	-113
ENSG0000(	COG8	1	protein_coding	-8
ENSG0000(	COG8	1	protein_coding	-431
ENSG0000(	SIPA1	1	protein_coding	465
ENSG0000(	SIPA1	1	protein_coding	827
ENSG0000(	ARL2	1	protein_coding	-133
ENSG0000(	ARL2	1	protein_coding	-742
ENSG0000(	ARL2	1	protein_coding	249
ENSG0000(	SRA1	1	protein_coding	583
ENSG0000(	ZBTB9	1	protein_coding	798
ENSG0000(	HEXA	1	protein_coding	405
ENSG0000(	LEPROT	1	protein_coding	810
ENSG0000(	ADAT3	1	protein_coding	-67
ENSG0000(	GPSM3	1	protein_coding	-628

ENSG0000(	ATF6B	1	protein_coding	-159
ENSG0000(	ATF6B	1	protein_coding	166
ENSG0000(	ATF6B	1	protein_coding	830
ENSG0000(	TREX1	1	protein_coding	-96
ENSG0000(	CLIC1	1	protein_coding	-431
ENSG0000(	CLIC1	1	protein_coding	253
ENSG0000(	DDAH2	1	protein_coding	837
ENSG0000(	GTF2H4	1	protein_coding	-77
ENSG0000(	DDX47	1	protein_coding	9
ENSG0000(	CCL27	1	protein_coding	-712
ENSG0000(	NUDT19	1	protein_coding	483
ENSG0000(	NUDT19	1	protein_coding	951
ENSG0000(	NAXD	1	protein_coding	-113
ENSG0000(	MEF2B	1	protein_coding	-569
ENSG0000(	REPIN1	1	protein_coding	-95
ENSG0000(	REPIN1	1	protein_coding	428
ENSG0000(	ZNF891	1	protein_coding	-247
ENSG0000(	SMIM7	1	protein_coding	-232
ENSG0000(	SMIM7	1	protein_coding	219
ENSG0000(	CPNE1	1	protein_coding	-94
ENSG0000(	LYRM4	1	protein_coding	151
ENSG0000(	ALG3	1	protein_coding	-823
ENSG0000(	SH3D21	1	protein_coding	806
ENSG0000(	C19orf38	1	protein_coding	98
ENSG0000(	FAM187A	1	protein_coding	-171
ENSG0000(	PPME1	1	protein_coding	214
ENSG0000(	STARD10	1	protein_coding	536
ENSG0000(	CPEB1	1	protein_coding	466
ENSG0000(	TOMM6	1	protein_coding	-522
ENSG0000(	HNRNPUL2	1	protein_coding	33
ENSG0000(	METTL12	1	protein_coding	-530
ENSG0000(	METTL12	1	protein_coding	335
ENSG0000(	ISPD	1	protein_coding	-301
ENSG0000(	ISPD	1	protein_coding	739
ENSG0000(	C22orf29	1	protein_coding	491
ENSG0000(	NEURL4	1	protein_coding	-476
ENSG0000(	PEX26	1	protein_coding	-199
ENSG0000(	FASTKD5	1	protein_coding	-1
ENSG0000(	FASTKD5	1	protein_coding	299
ENSG0000(	FASTKD5	1	protein_coding	771
ENSG0000(	DDX3X	1	protein_coding	561
ENSG0000(	DDX3X	1	protein_coding	846
ENSG0000(	VPS16	1	protein_coding	-264
ENSG0000(	MYL5	1	protein_coding	852
ENSG0000(	RPL17-C18orf32	1	protein_coding	21
ENSG0000(	TMEM242	1	protein_coding	-24
ENSG0000(	TMEM167B	1	protein_coding	-215
ENSG0000(	TMEM167B	1	protein_coding	832
ENSG0000(	C1orf167	1	protein_coding	275
ENSG0000(	TTC34	1	protein_coding	-27
ENSG0000(	FNIP1	1	protein_coding	-13
ENSG0000(	SP9	1	protein_coding	306
ENSG0000(	CKLF	1	protein_coding	-767
ENSG0000(	CEBPZOS	1	protein_coding	-93

ENSG0000(	CEBPZOS	1	protein_coding	471
ENSG0000(	NBPF1	1	protein_coding	-77
ENSG0000(	UMAD1	1	protein_coding	-639
ENSG0000(	UMAD1	1	protein_coding	147
ENSG0000(	FAM228B	1	protein_coding	-308
ENSG0000(	C6orf226	1	protein_coding	406
ENSG0000(	C6orf226	1	protein_coding	749
ENSG0000(	C19orf73	1	protein_coding	622
ENSG0000(	FADS3	1	protein_coding	885
ENSG0000(	CCNL2	1	protein_coding	-228
ENSG0000(	CCNL2	1	protein_coding	821
ENSG0000(	MYBPHL	1	protein_coding	-544
ENSG0000(	BTBD19	1	protein_coding	-147
ENSG0000(	VPS52	1	protein_coding	478
ENSG0000(	CERS1	1	protein_coding	-839
ENSG0000(	SMIM13	1	protein_coding	-310
ENSG0000(	MCRIP1	1	protein_coding	-524
ENSG0000(	MCRIP1	1	protein_coding	467
ENSG0000(	SLC26A6	1	protein_coding	873
ENSG0000(	ERCC6	1	protein_coding	74
ENSG0000(	LINC00694	1	protein_coding	216
ENSG0000(	TMEM185B	1	protein_coding	461
ENSG0000(	TMEM185B	1	protein_coding	860
ENSG0000(	SRRM5	1	protein_coding	-100
ENSG0000(	WDR46	1	protein_coding	80
ENSG0000(	SCAMP4	1	protein_coding	97
ENSG0000(	C19orf24	1	protein_coding	-371
ENSG0000(	C19orf24	1	protein_coding	225
ENSG0000(	TSTD3	1	protein_coding	762
ENSG0000(	DHFR	1	protein_coding	-452
ENSG0000(	PATL2	1	protein_coding	-287
ENSG0000(	ACBD6	1	protein_coding	-395
ENSG0000(	ACBD6	1	protein_coding	533
ENSG0000(	RPS18	1	protein_coding	-440
ENSG0000(	TAPBP	1	protein_coding	-304
ENSG0000(	TAPBP	1	protein_coding	344
ENSG0000(	ZBED9	1	protein_coding	113
ENSG0000(	ZBED9	1	protein_coding	465
ENSG0000(	AC109829.1	1	protein_coding	-213
ENSG0000(	TMEM238	1	protein_coding	-141
ENSG0000(	HIST1H2BN	1	protein_coding	-662
ENSG0000(	RPS28	1	protein_coding	656
ENSG0000(	FAM133B	1	protein_coding	222
ENSG0000(	RP11-166B2.1	1	protein_coding	57
ENSG0000(	HNRNPUL2-BSCL2	1	protein_coding	68
ENSG0000(	C19orf81	1	protein_coding	112
ENSG0000(	ZSCAN31	1	protein_coding	63
ENSG0000(	ZSCAN31	1	protein_coding	866
ENSG0000(	SMIM1	1	protein_coding	334
ENSG0000(	B3GALT4	1	protein_coding	222
ENSG0000(	B3GALT4	1	protein_coding	555
ENSG0000(	ZBTB22	1	protein_coding	-34
ENSG0000(	ZBED5	1	protein_coding	-367
ENSG0000(	RGL2	1	protein_coding	-258

ENSG0000(	LINC00959	1	protein_coding	389
ENSG0000(	KIFC1	1	protein_coding	-380
ENSG0000(	C9orf69	1	protein_coding	37
ENSG0000(	C9orf69	1	protein_coding	959
ENSG0000(	RBM14	1	protein_coding	144
ENSG0000(	ALKBH6	1	protein_coding	-376
ENSG0000(	ALKBH6	1	protein_coding	24
ENSG0000(	NME1	1	protein_coding	-119
ENSG0000(	GET4	1	protein_coding	-313
ENSG0000(	COX19	1	protein_coding	212
ENSG0000(	PPIL3	1	protein_coding	-339
ENSG0000(	TMEM189	1	protein_coding	-473
ENSG0000(	TMEM189	1	protein_coding	229
ENSG0000(	CRCP	1	protein_coding	260
ENSG0000(	CRCP	1	protein_coding	829
ENSG0000(	RPL36A	1	protein_coding	142
ENSG0000(	RPP21	1	protein_coding	-197
ENSG0000(	RPP21	1	protein_coding	-690
ENSG0000(	ARPC1A	1	protein_coding	-105
ENSG0000(	ARPC1A	1	protein_coding	-356
ENSG0000(	ATP5O	1	protein_coding	998
ENSG0000(	PWP2	1	protein_coding	-224
ENSG0000(	MTFP1	1	protein_coding	880
ENSG0000(	C22orf39	1	protein_coding	779
ENSG0000(	EIF6	1	protein_coding	-132
ENSG0000(	MRPL20	1	protein_coding	33
ENSG0000(	GNG10	1	protein_coding	-11
ENSG0000(	AP5Z1	1	protein_coding	-571
ENSG0000(	EIF4EBP3	1	protein_coding	-158
ENSG0000(	MICAL3	1	protein_coding	-168
ENSG0000(	MICAL3	1	protein_coding	444
ENSG0000(	PPAN-P2RY11	1	protein_coding	-176
ENSG0000(	C7orf73	1	protein_coding	-947
ENSG0000(	C7orf73	1	protein_coding	10
ENSG0000(	KCTD7	1	protein_coding	-296
ENSG0000(	NAT6	1	protein_coding	-857
ENSG0000(	NAT6	1	protein_coding	181
ENSG0000(	ZNF487	1	protein_coding	-323
ENSG0000(	TTC4	1	protein_coding	-154
ENSG0000(	MRPS6	1	protein_coding	-107
ENSG0000(	ACY1	1	protein_coding	-276
ENSG0000(	ACY1	1	protein_coding	-658
ENSG0000(	ACY1	1	protein_coding	836
ENSG0000(	DDOST	1	protein_coding	-28
ENSG0000(	TMEM199	1	protein_coding	61
ENSG0000(	TMEM199	1	protein_coding	362
ENSG0000(	DNAJC25-GNG10	1	protein_coding	-233
ENSG0000(	TMEM141	1	protein_coding	111
ENSG0000(	ETV5	1	protein_coding	911
ENSG0000(	RBM12	1	protein_coding	-94
ENSG0000(	SCARF2	1	protein_coding	-906
ENSG0000(	SCARF2	1	protein_coding	518
ENSG0000(	UBE2V1	1	protein_coding	-1
ENSG0000(	AC037459.4	1	protein_coding	-68

ENSG0000(	RBM14-RBM4	1	protein_coding	100
ENSG0000(	RP11-432B6.3	1	protein_coding	-280
ENSG0000(	RP11-432B6.3	1	protein_coding	-808
ENSG0000(	RP11-432B6.3	1	protein_coding	374
ENSG0000(	RP11-514O12.4	1	protein_coding	657
ENSG0000(	AP000304.12	1	protein_coding	-56
ENSG0000(	ZNF324B	1	protein_coding	-73
ENSG0000(	PDCD6	1	protein_coding	-114
ENSG0000(	TMEM158	1	protein_coding	-493
ENSG0000(	ATP6V1E2	1	protein_coding	597
ENSG0000(	XXbac-BPG32J3.19	1	protein_coding	-9
ENSG0000(	ZNF674	1	protein_coding	-61
ENSG0000(	APITD1-CORT	1	protein_coding	147
ENSG0000(	RP11-724O16.1	1	protein_coding	216
ENSG0000(	C8orf88	1	protein_coding	-521
ENSG0000(	C8orf88	1	protein_coding	-771
ENSG0000(	C8orf88	1	protein_coding	545
ENSG0000(	CCDC71L	1	protein_coding	967
ENSG0000(	ZNF260	1	protein_coding	-418
ENSG0000(	PINX1	1	protein_coding	-303
ENSG0000(	LRRC24	1	protein_coding	374
ENSG0000(	HSPB2-C11orf52	1	protein_coding	-757
ENSG0000(	RP11-849H4.2	1	protein_coding	532
ENSG0000(	RP11-691N7.6	1	protein_coding	410
ENSG0000(	EEF1G	1	protein_coding	-41
ENSG0000(	EEF1G	1	protein_coding	-519
ENSG0000(	CKLF-CMTM1	1	protein_coding	-791
ENSG0000(	SYS1-DBNDD2	1	protein_coding	-335
ENSG0000(	MPV17L2	1	protein_coding	876
ENSG0000(	ATP6V1G2-DDX39B	1	protein_coding	-130
ENSG0000(	BORCS8	1	protein_coding	207
ENSG0000(	RP11-872D17.8	1	protein_coding	-672
ENSG0000(	ANKHD1-EIF4EBP3	1	protein_coding	-525
ENSG0000(	ANKHD1-EIF4EBP3	1	protein_coding	300
ENSG0000(	ANKHD1-EIF4EBP3	1	protein_coding	554
ENSG0000(	BRK1	1	protein_coding	-137
ENSG0000(	ZFP91-CNTF	1	protein_coding	644
ENSG0000(	MSH5-SAPCD1	1	protein_coding	-509
ENSG0000(	MSH5-SAPCD1	1	protein_coding	175
ENSG0000(	RP11-80H18.3	1	protein_coding	3
ENSG0000(	AP002884.2	1	protein_coding	-111
ENSG0000(	AP002884.2	1	protein_coding	-416
ENSG0000(	EID1	1	protein_coding	221
ENSG0000(	RP11-831H9.11	1	protein_coding	-465
ENSG0000(	RP11-831H9.11	1	protein_coding	400
ENSG0000(	POLR2M	1	protein_coding	-129
ENSG0000(	RP11-212D19.4	1	protein_coding	-275
ENSG0000(	CTC-435M10.3	1	protein_coding	-936
ENSG0000(	RP4-559A3.7	1	protein_coding	743
ENSG0000(	AP000721.4	1	protein_coding	85
ENSG0000(	AP000721.4	1	protein_coding	346
ENSG0000(	ZNF10	1	protein_coding	146
ENSG0000(	HMBS	1	protein_coding	916
ENSG0000(	AP003419.11	1	protein_coding	-918

ENSG0000(	RP4-734P14.4	1	protein_coding	-205
ENSG0000(	RP11-111K18.1	1	protein_coding	162
ENSG0000(	STH	1	protein_coding	-488
ENSG0000(	CTD-2140B24.4	1	protein_coding	-263
ENSG0000(	RP11-512M8.5	1	protein_coding	-23
ENSG0000(	RP11-87C12.2	1	protein_coding	-211
ENSG0000(	HP	1	protein_coding	-642
ENSG0000(	NHLRC4	1	protein_coding	-12
ENSG0000(	GATC	1	protein_coding	-89
ENSG0000(	RPL36A-HNRNPH2	1	protein_coding	-45
ENSG0000(	INAFM1	1	protein_coding	-699
ENSG0000(	CNPY2	1	protein_coding	-69
ENSG0000(	RTEL1	1	protein_coding	-195
ENSG0000(	RTEL1	1	protein_coding	471
ENSG0000(	RTEL1	1	protein_coding	808
ENSG0000(	PPT2-EGFL8	1	protein_coding	714
ENSG0000(	RP11-108O10.8	1	protein_coding	-236
ENSG0000(	RP11-108O10.8	1	protein_coding	326
ENSG0000(	CTC-260F20.3	1	protein_coding	485
ENSG0000(	CTD-2135J3.4	1	protein_coding	-340
ENSG0000(	RP11-561B11.2	1	protein_coding	-338
ENSG0000(	CEP95	1	protein_coding	252
ENSG0000(	TUBB3	1	protein_coding	644
ENSG0000(	BLOC1S5-TXNDC5	1	protein_coding	-254
ENSG0000(	NDUFC2-KCTD14	1	protein_coding	-959
ENSG0000(	NDUFC2-KCTD14	1	protein_coding	107
ENSG0000(	NDUFC2-KCTD14	1	protein_coding	466
ENSG0000(	ITGB3	1	protein_coding	416
ENSG0000(	RP11-290H9.2	1	protein_coding	365
ENSG0000(	RP11-96O20.4	1	protein_coding	-467
ENSG0000(	C16orf95	1	protein_coding	338
ENSG0000(	RP11-106M3.2	1	protein_coding	-90
ENSG0000(	RP11-403P17.5	1	protein_coding	85
ENSG0000(	AC002310.13	1	protein_coding	-999
ENSG0000(	AC002310.13	1	protein_coding	48
ENSG0000(	XKR7	1	protein_coding	547
ENSG0000(	CCPG1	1	protein_coding	925
ENSG0000(	AC002310.11	1	protein_coding	99
ENSG0000(	TPBGL	1	protein_coding	499
ENSG0000(	TPBGL	1	protein_coding	846
ENSG0000(	C15orf65	1	protein_coding	-962
ENSG0000(	CTC-479C5.12	1	protein_coding	601
ENSG0000(	GFY	1	protein_coding	-919
ENSG0000(	CORO7	1	protein_coding	-986
ENSG0000(	CORO7	1	protein_coding	92
ENSG0000(	RP11-156P1.2	1	protein_coding	-160
ENSG0000(	IKBKE	1	protein_coding	28
ENSG0000(	RNF115	1	protein_coding	-364
ENSG0000(	AP000275.65	1	protein_coding	-274
ENSG0000(	AP000275.65	1	protein_coding	-775
ENSG0000(	RPL17	1	protein_coding	65
ENSG0000(	SEC22B	1	protein_coding	27
ENSG0000(	MRPS21	1	protein_coding	-38
ENSG0000(	MRPS21	1	protein_coding	393

ENSG0000(	AARSD1	1	protein_coding	24
ENSG0000(	AC006538.4	1	protein_coding	624
ENSG0000(	PTGES3L	1	protein_coding	-65
ENSG0000(	PTGES3L	1	protein_coding	-575
ENSG0000(	RP11-322E11.6	1	protein_coding	25
ENSG0000(	RP11-322E11.6	1	protein_coding	592
ENSG0000(	RP11-318A15.7	1	protein_coding	257
ENSG0000(	AC104532.2	1	protein_coding	-896
ENSG0000(	AC104532.2	1	protein_coding	288
ENSG0000(	CTB-50L17.14	1	protein_coding	47
ENSG0000(	S1PR2	1	protein_coding	-869
ENSG0000(	AC024592.12	1	protein_coding	-157
ENSG0000(	SMIM22	1	protein_coding	440
ENSG0000(	LIN37	1	protein_coding	143
ENSG0000(	NDUFA7	1	protein_coding	-417
ENSG0000(	AC004076.9	1	protein_coding	-138
ENSG0000(	CTD-2583A14.10	1	protein_coding	-33
ENSG0000(	CTC-429P9.4	1	protein_coding	-108
ENSG0000(	CTC-429P9.4	1	protein_coding	-559
ENSG0000(	CTD-2278I10.6	1	protein_coding	-338
ENSG0000(	IKBKG	1	protein_coding	-181
ENSG0000(	IKBKG	1	protein_coding	349
ENSG0000(	IKBKG	1	protein_coding	698
ENSG0000(	ZNF587B	1	protein_coding	-55
ENSG0000(	CTD-2192J16.24	1	protein_coding	-797
ENSG0000(	CTD-3105H18.18	1	protein_coding	-263
ENSG0000(	EGLN2	1	protein_coding	-460
ENSG0000(	EGLN2	1	protein_coding	-790
ENSG0000(	C7orf55-LUC7L2	1	protein_coding	-12
ENSG0000(	ZNF559-ZNF177	1	protein_coding	646
ENSG0000(	TSNAX-DISC1	1	protein_coding	-60
ENSG0000(	MINOS1-NBL1	1	protein_coding	-248
ENSG0000(	NCBP2-AS2	1	protein_coding	-99
ENSG0000(	NCBP2-AS2	1	protein_coding	675
ENSG0000(	BORCS7-ASMT	1	protein_coding	-205
ENSG0000(	RPS10-NUDT3	1	protein_coding	-179
ENSG0000(	RPS10-NUDT3	1	protein_coding	306
ENSG0000(	RPS10-NUDT3	1	protein_coding	665
ENSG0000(	SRXN1	1	protein_coding	-600
ENSG0000(	RP11-244H3.4	1	protein_coding	33
ENSG0000(	RP11-302M6.4	1	protein_coding	-371
ENSG0000(	RP11-302M6.4	1	protein_coding	139
ENSG0000(	GTF2H5	1	protein_coding	215
ENSG0000(	NUDT3	1	protein_coding	-263
ENSG0000(	NUDT3	1	protein_coding	892
ENSG0000(	RP11-438J1.1	1	protein_coding	-362
ENSG0000(	ZNF595	1	protein_coding	-163
ENSG0000(	COG8	1	protein_coding	-347
ENSG0000(	COG8	1	protein_coding	76
ENSG0000(	CTD-2410N18.5	1	protein_coding	-361
ENSG0000(	CTC-487M23.8	1	protein_coding	-268
ENSG0000(	CTC-487M23.8	1	protein_coding	47
ENSG0000(	DCP1A	1	protein_coding	-74
ENSG0000(	RP11-216L13.17	1	protein_coding	87

ENSG0000(	RP11-500M8.7	1	protein_coding	-156
ENSG0000(	ARL2-SNX15	1	protein_coding	-202
ENSG0000(	ARL2-SNX15	1	protein_coding	-811
ENSG0000(	ARL2-SNX15	1	protein_coding	180
ENSG0000(	RP11-38C17.1	1	protein_coding	-10
ENSG0000(	RP11-38C17.1	1	protein_coding	340
ENSG0000(	CTC-432M15.3	1	protein_coding	-93
ENSG0000(	RP11-761B3.1	1	protein_coding	-515
ENSG0000(	RP11-761B3.1	1	protein_coding	90
ENSG0000(	TM4SF19-TCTEX1D2	1	protein_coding	59
ENSG0000(	TM4SF19-TCTEX1D2	1	protein_coding	770
ENSG0000(	CWC25	1	protein_coding	-195
ENSG0000(	CWC25	1	protein_coding	49
ENSG0000(	TAF9	1	protein_coding	236
ENSG0000(	RP11-475E11.9	1	protein_coding	361
ENSG0000(	RCC1L	1	protein_coding	180
ENSG0000(	HIST1H2BO	1	protein_coding	-156
ENSG0000(	HIST1H2AH	1	protein_coding	-77
ENSG0000(	HIST1H2AH	1	protein_coding	524
ENSG0000(	PPP4R3B	1	protein_coding	-41
ENSG0000(	PPP4R3B	1	protein_coding	486
ENSG0000(	SYNRG	1	protein_coding	-300
ENSG0000(	NUDT18	1	protein_coding	-389
ENSG0000(	ZNF2	1	protein_coding	139
ENSG0000(	HIST1H4I	1	protein_coding	656
ENSG0000(	TADA2A	1	protein_coding	-646
ENSG0000(	HIST1H4D	1	protein_coding	89
ENSG0000(	PIGW	1	protein_coding	64
ENSG0000(	PIGW	1	protein_coding	500
ENSG0000(	ZNF670	1	protein_coding	-48
ENSG0000(	RP11-467J12.4	1	protein_coding	65
ENSG0000(	AC009133.22	1	protein_coding	319
ENSG0000(	ACACA	1	protein_coding	591
ENSG0000(	C11orf98	1	protein_coding	-426
ENSG0000(	C11orf98	1	protein_coding	439
ENSG0000(	HIST1H2AM	1	protein_coding	-157
ENSG0000(	HIST1H4B	1	protein_coding	-17
ENSG0000(	AL358852.1	1	protein_coding	822
ENSG0000(	AC006449.2	1	protein_coding	-423
ENSG0000(	AC019206.1	1	protein_coding	161
ENSG0000(	GVQW2	1	protein_coding	13
ENSG0000(	AF131216.1	1	protein_coding	773
ENSG0000(	AC138028.1	1	protein_coding	20
ENSG0000(	AC138028.1	1	protein_coding	299
ENSG0000(	CCDC163P	1	protein_coding	99
ENSG0000(	PAGR1	1	protein_coding	-547
ENSG0000(	PAGR1	1	protein_coding	174
ENSG0000(	MATR3	1	protein_coding	-186
ENSG0000(	MATR3	1	protein_coding	116
ENSG0000(	RP11-717K11.2	1	protein_coding	-30
ENSG0000(	CTD-2574D22.6	1	protein_coding	19
ENSG0000(	AC126407.1	1	protein_coding	731
ENSG0000(	CTB-127M13.1	1	protein_coding	-376
ENSG0000(	RP11-231C18.3	1	protein_coding	366

ENSG0000(	CYP3A7-CYP3A51P	1	protein_coding	716
ENSG0000(	C11orf71	1	protein_coding	-75
ENSG0000(	RP11-96L14.8	1	protein_coding	-965
ENSG0000(	AL513122.1	1	protein_coding	-446
ENSG0000(	RP5-994D16.12	1	protein_coding	79

: v2.0: decoding transcriptional regulatory networks of non-coding RNAs and protein-c
lecoding the transcriptional regulation of long non-coding RNA and microRNA genes frc

:04533, regulatory domain is [-1kb, 1kb]

Binding Site Start	Binding Site End	Motif(start:end:strand:sequence)
169894001	169894377	-
143511256	143511877	-
143510864	143511246	-
41072106	41072749	-
24416901	24417211	-
24415355	24415971	-
92246495	92246783	-
92246495	92246783	-
201116287	201116420	-
50088604	50088828	-
50089131	50089300	-
28357577	28357705	-
28357844	28358041	-
23019054	23019449	-
93232100	93232473	-
35757806	35758123	-
47188852	47189290	-
18831603	18831770	-
20806770	20807123	-
2327756	2327880	-
3881067	3881196	-
105013122	105014147	-
82248197	82248772	-
120468513	120468688	-
44389575	44390408	-
18588385	18588629	-
18587874	18588027	-
47821839	47822107	-
32927465	32927652	-
32928616	32928793	-
24255902	24256089	-
140177229	140177505	-
140177513	140177962	-
13990606	13991051	-
141550768	141551555	-
54801480	54801705	-
77798225	77798499	-
30505009	30505218	-
30503985	30504188	-
39390855	39391491	-
7927702	7927888	-
134370623	134371371	-
2817071	2817174	2817199:2817210:+:CCCCCAGGGGGC
45079036	45079402	-
17932548	17933094	-
112619740	112620161	-

788620	788877	-
229286	229587	-
566848	567119	-
1611823	1612100	-
16129873	16130230	-
170553632	170554192	-
47620057	47620216	-
209582725	209582900	-
1780255	1780712	-
1781137	1781415	-
1779883	1780081	-
1779230	1779694	-
4343391	4343495	-
107093622	107094242	-
12994718	12995233	-
37084545	37085043	-
37085060	37085278	-
56074599	56075129	-
114757544	114758594	-
111483901	111484235	-
27725358	27725899	27725756:27725767:+:CCTCCAGGTGGC
142146940	142147385	-
73623591	73623765	-
73522385	73522959	-
13712118	13712596	-
231336005	231336620	-
231337647	231338314	-
171780860	171781391	-
171781951	171782286	-
32350072	32350281	-
45667716	45667988	-
45668383	45668855	-
52455190	52455337	-
49817966	49818289	-
6873860	6874096	-
101390754	101391177	-
26087549	26087917	-
41242642	41242935	-
23790181	23790947	-
51153159	51153673	-
93332121	93332288	-
3761605	3762096	3761750:3761761:+:CCACGCGGGGGC
58219320	58219643	-
43690371	43690876	-
796742	797169	-
45388221	45388663	-
45388691	45388936	-
17208256	17208523	-
17208804	17208950	-
45692475	45692738	-
45692761	45693275	-
46346370	46347155	-
53348847	53349350	-
39970461	39971129	-

39970212	39970446	-
83192735	83192975	-
200864630	200864855	-
106357523	106357851	106357657:106357668:+:ACACCAGAGGGC
65261859	65262111	-
63588437	63589195	-
41990497	41990673	-
48676562	48676701	-
139294223	139294488	-
50345665	50345801	-
52706141	52706280	-
52705953	52706064	-
9475003	9475431	-
35557196	35557797	-
52197870	52197993	-
193105506	193105930	-
35297392	35298117	-
35298644	35298986	-
42563615	42564578	-
138773362	138773917	-
122399772	122400042	-
35771519	35771916	-
47248724	47249414	-
32107872	32108357	-
63658447	63658787	-
166957539	166957701	-
892197	892522	-
64093530	64094066	-
64093085	64093517	-
136288396	136290818	-
69398359	69398776	-
47848712	47849037	-
47848025	47848305	-
49463020	49463768	-
109213958	109214241	-
5978236	5978586	-
3377735	3378195	-
3378605	3378843	-
151231756	151232260	-
151233136	151233362	-
143450588	143450920	-
75565692	75565879	-
73994733	73995030	-
60699658	60699900	-
58660060	58660394	-
39366280	39366652	-
119381780	119382190	-
16352460	16353215	-
16353294	16353438	-
154038382	154038904	-
67871997	67872134	-
55307795	55308469	-
49877280	49877667	-
19312548	19312867	-

19312919	19313242	-
20282936	20283419	-
78540005	78540242	-
6624588	6625024	-
50690621	50690919	50690763:50690774:+:CCTGCAGGGGGC
89954428	89954766	-
170297155	170297379	-
43053454	43053804	-
43053028	43053434	-
125241615	125242125	-
125241107	125241444	-
1049409	1049674	-
1049919	1050094	-
186375216	186375939	186375615:186375626:+:CCACTAGAGGGC
117602548	117602934	-
176646888	176647090	-
176388429	176388895	-
102238420	102238658	-
11976490	11976683	-
42217445	42218043	-
12229625	12229925	-
65481834	65482112	-
74254519	74254740	-
74254003	74254148	-
26151807	26151966	-
13814040	13814359	-
51048048	51048389	-
131008487	131009057	-
6235196	6235484	-
40694664	40694859	-
40693849	40694155	-
138835199	138835467	-
31135723	31136114	-
19312548	19312867	-
19312919	19313242	-
19251308	19252031	-
36223355	36224695	-
36224932	36225214	-
136848087	136848244	-
243255128	243255589	-
243255710	243256648	-
10210219	10210612	10210347:10210358:+:CCACCAGGGGGC
73598547	73598911	-
16352460	16353215	-
16353294	16353438	-
150977299	150977493	-
148697024	148698199	-
154038382	154038904	-
78840929	78841157	-
20351331	20351583	-
106325637	106326124	-
23777731	23778337	-
182792872	182793197	-
53890027	53890736	-

53838260	53838832	-
140062634	140063451	-
111631032	111631273	-
102422299	102422449	-
102420784	102421187	-
68720890	68721153	-
26785800	26788506	-
29236800	29237161	-
166382743	166382936	-
166382120	166382286	-
38137374	38137596	-
24948948	24949584	-
183038831	183039172	-
56300728	56301070	-
56301127	56301486	-
56299839	56300029	-
41514457	41514623	-
41513770	41513971	-
59707276	59707523	-
47608161	47608270	-
39390855	39391491	-
1827018	1827353	-
27014400	27015120	-
2301722	2301901	-
2302535	2302780	-
129054372	129055307	-
19192055	19192713	-
19192055	19192713	-
19033979	19034253	-
1040154	1040503	-
111755606	111756030	111755622:111755633:+:CCAGAAGAGGGC
83067301	83067460	-
83067480	83067788	-
1174347	1174547	-
34887897	34888478	-
34791797	34792051	-
34791237	34791785	-
109547532	109548789	-
984125	984299	-
37257738	37258157	-
15847102	15847511	-
186485726	186486439	-
212414929	212415152	-
212415183	212415735	-
83859313	83859946	-
6580787	6581041	-
238426258	238426402	-
42681223	42681844	-
10416301	10417194	10416660:10416671:-:GCCCCCCAGTGG
212285662	212285857	-
212286308	212286720	-
8006073	8006206	-
43650513	43650888	-
40691112	40691914	-

40691971	40692665	-
197146789	197147049	-
197146141	197146357	-
43368367	43368740	-
43367143	43367493	-
47378408	47378819	-
125986087	125986916	-
100426531	100426763	-
134713477	134713895	-
65644232	65644711	-
65644717	65644937	-
57587039	57587251	-
1049409	1049674	-
1049919	1050094	-
230415920	230416032	-
70608395	70608783	-
72713706	72713823	-
6341857	6342047	-
73995390	73995540	-
55307795	55308469	-
8621828	8622155	-
43510999	43511576	-
218285128	218285434	-
153794053	153794307	-
4801947	4802171	-
67028539	67028947	-
42676351	42676474	-
132537057	132537186	-
48475791	48476273	-
59154787	59155133	-
86106427	86106657	-
20051560	20051960	-
46916340	46916652	-
62305607	62306060	-
53969824	53970009	-
160399506	160399784	-
38899453	38899991	-
38899131	38899397	-
135034944	135035060	-
135035314	135035490	-
229508265	229508862	-
229508869	229509528	-
44746847	44747007	-
6425165	6425363	-
108934514	108934878	-
17208256	17208523	-
17208804	17208950	-
19291874	19293064	-
19122130	19123648	-
2401474	2401720	-
2401768	2401985	-
76643541	76644373	-
76642939	76643343	-
35749916	35750085	-

100723098	100723288	-
44985668	44986126	-
22052664	22052846	-
101001567	101001727	-
101001903	101002415	-
10117034	10117602	-
17939821	17940158	-
120950967	120951702	-
892197	892522	-
891716	891837	-
149086429	149086850	-
171522012	171522449	-
170583989	170584214	170583925:170583936:-:GCCTCCTAGAGG
67695105	67695376	-
14118458	14118809	-
196082356	196082606	-
196081956	196082138	-
17836590	17836744	-
17836006	17836429	-
132963974	132964168	-
53422951	53423140	-
163459914	163460408	-
163460722	163461130	-
132295837	132296146	-
68084483	68084777	68084709:68084720:-:GCCCGCTAGTGG
172006776	172007116	-
16660521	16660828	-
16660135	16660311	-
84980202	84980461	-
84981289	84981513	-
35142305	35142490	-
185283285	185283470	-
1773420	1773604	-
1772190	1772420	-
77637718	77638102	-
77638357	77638448	-
176541686	176541912	-
176542254	176542779	-
18526681	18527097	-
3965165	3965367	-
59359733	59359909	-
65530413	65530614	-
8878507	8878765	-
4670215	4670363	-
17334699	17335015	-
17333920	17334595	-
21177051	21177431	-
97996268	97996480	-
5563713	5563867	5563746:5563757:+:CCGCCAGGTGGC
36188040	36188223	-
171809998	171810212	-
36054375	36054842	36054615:36054626:+:CCTCTAGGTGGC
36053940	36054275	-
107580480	107580924	-

108561171	108561555	-
44975414	44975734	-
12556780	12557253	-
135875389	135875616	-
114439719	114440306	-
114400253	114400560	-
56636455	56636743	-
47380547	47381163	-
36991983	36993232	-
36993351	36993800	-
173714431	173715173	-
173715314	173715745	-
28598895	28599460	-
28743109	28743450	-
119321545	119321769	-
21669479	21669779	-
21669818	21670666	-
7903314	7903896	-
202341605	202341971	-
202348263	202348963	-
202348998	202349603	-
182716476	182716838	-
40750064	40750653	-
171687093	171687822	-
95789664	95789877	-
76860155	76860301	-
76861518	76861754	-
157981322	157981722	-
157981051	157981313	-
100429771	100429978	-
100427967	100428615	100428287:100428298:+:CCACCCGAGGGC
51878934	51879786	-
51878389	51878784	-
51877987	51878291	-
42391505	42391627	-
17922565	17922924	-
1231241	1231812	-
43926523	43926769	-
267905	269017	-
267236	267746	-
178478350	178479485	-
178193930	178194189	-
46616898	46617123	-
70169832	70170014	-
75108310	75109252	-
42268069	42268459	-
29790434	29790660	-
29791012	29791364	-
15591325	15591904	-
10502772	10502982	-
46364258	46364451	-
151409524	151409962	-
151409989	151410256	151410096:151410107:-:GCCCCCTCGCGG
109095164	109095799	-

135510175	135511284	-
73137075	73137366	-
102305099	102305375	-
37095904	37096131	-
2570674	2572465	-
50908497	50908685	50908601:50908612:-:GCCCTCTGGTGG
113812167	113812481	-
69191220	69191594	-
141923359	141923553	-
62714873	62715176	-
151277150	151277569	-
50878542	50878681	-
134917851	134918010	-
154937314	154937557	-
48047488	48048058	-
61538982	61539481	-
42052326	42052500	-
11933986	11934427	-
44989483	44989606	-
41870468	41870740	-
58385776	58386120	-
58386636	58386987	-
58387077	58387401	-
68350179	68350380	-
40257615	40258091	-
96208334	96208549	-
108856321	108856876	-
32221347	32221633	-
32222204	32222668	-
32179296	32179978	-
32178638	32179291	-
32180434	32180595	-
26245215	26245659	-
26244082	26244376	-
33736566	33736748	-
44564300	44564491	-
69369582	69369973	-
109041970	109042172	-
160992246	160993030	-
15094312	15094453	-
70398071	70398223	-
70397688	70397941	-
51346006	51346202	-
52403613	52404368	-
139665499	139665767	-
139665908	139666260	-
46246910	46247377	-
33290291	33290652	-
49967122	49967323	49967145:49967156:+:CCAGCAGAGGGC
181051	181181	-
40716299	40716651	-
16786569	16786692	-
95924190	95924618	-
48872359	48872588	-

48835715	48836064	48835898:48835909:-:GCCCTCTTGTGG
100866890	100867217	-
100896059	100896228	-
48965913	48966315	-
63827004	63827533	-
177023320	177023501	-
55508796	55508919	-
2962919	2963240	-
2962415	2962577	-
2844531	2844701	-
69829307	69829587	-
27780622	27780760	-
56392335	56392848	-
63367018	63367189	-
63368075	63368216	-
54136606	54137206	-
55038126	55038590	-
119759865	119760151	-
6425301	6426020	-
32763010	32763185	-
47282345	47282814	-
2524460	2524693	2524554:2524565:+:CCAACAGGGGGC
2800143	2800307	-
3847551	3847782	-
120470011	120470412	-
17968825	17968960	17968712:17968723:+:CCACGAGGAGGC
17968632	17968818	17968713:17968724:+:CCACGAGGAGGC
112419364	112419668	-
111841620	111842075	-
13784737	13785072	-
121581438	121581841	-
20052342	20052556	-
120194956	120195404	-
112012616	112013229	-
35153807	35153917	-
19644028	19644221	19644018:19644029:-:GCCCCCTGGTGG
78214114	78214364	-
8081983	8082325	-
40466067	40466600	-
40464996	40465336	-
96502229	96502387	-
51973911	51974266	51974097:51974108:-:GCCCTCTTGTGG
141013864	141015337	-
26900118	26901072	-
10270350	10270588	-
10270911	10271057	10271072:10271083:-:GCCCCCAGGTGG
20507995	20508308	-
20507268	20507473	-
65133859	65134184	-
49473868	49474205	49474150:49474161:+:CCACCAGGGGGC
49474709	49474839	-
21783133	21784109	-
21782570	21783031	-
76560056	76560252	-

107890790	107891366	-
107891494	107891895	-
56651043	56651185	-
56651043	56651185	-
173075005	173075548	-
133572853	133573263	-
18183264	18183423	-
35122051	35122369	-
22957190	22957447	-
30621821	30622103	-
31208299	31208471	-
41558515	41558758	-
42491079	42491346	-
158819664	158820170	-
35769789	35770140	-
19940624	19943389	-
48800630	48801366	-
53324237	53324743	-
54280324	54280705	-
54280797	54281421	-
12681829	12682031	-
120842172	120843297	-
127779048	127779226	-
98255944	98256189	-
114504346	114504717	-
93073229	93073379	-
28532828	28533011	-
35126984	35127125	35126881:35126892:+:CCGGCAGGGGGC
1782688	1782869	-
12008281	12008958	-
35728124	35728891	-
43687374	43688444	-
52670844	52671157	-
52361713	52362054	-
44245669	44245948	-
27472251	27473318	-
67883794	67884171	-
68331319	68332203	-
26947303	26947430	-
6393932	6394170	-
91500271	91500656	-
10836363	10836982	-
27503783	27504223	-
27504234	27504406	-
24951670	24951997	-
58572563	58572932	-
17074717	17075181	-
38335556	38335826	-
38374123	38374319	-
30922201	30923488	-
30957084	30957463	-
30957576	30958005	-
30895011	30895670	-
30895703	30896118	-

30894392	30894854	-
30894219	30894378	-
1259321	1259507	-
1241509	1241825	-
1238649	1238784	-
14571839	14572271	-
2427005	2427201	-
633391	633695	-
2096030	2096205	-
2051375	2051839	-
2051167	2051291	2051351:2051362:+:CCAGAAGAGGGC
20016114	20018819	-
20116488	20117551	-
20113496	20116484	-
20128612	20129988	-
20495869	20497028	-
20494587	20495620	-
20494587	20495620	-
20495869	20497028	-
20856713	20860100	-
20916672	20918618	-
20979497	20980973	-
21009635	21010585	-
21665035	21665496	-
21665711	21666568	-
21734005	21735905	-
21867629	21868390	-
21866357	21867435	-
21952552	21953647	21952951:21952962:-:GCCCCCTGGCGG
21951623	21952474	-
21981767	21983147	-
19143846	19145085	-
19446268	19448639	-
26484113	26484431	-
26484113	26484431	-
26618131	26618355	-
37905539	37905677	-
37953245	37953370	-
31753160	31753482	-
38506712	38506982	-
38570732	38570858	-
28742217	28742431	-
42648589	42648710	-
43090155	43090286	-
39320028	39320180	-
36027886	36028041	-
36252814	36252946	-
36507275	36507571	-
36529091	36529469	-
40044106	40044481	-
40818788	40819008	40818989:40819000:+:CCAGGAGGTGGC
40856857	40857042	40856895:40856906:+:CCTCCAGGGGGC
41205044	41205249	-
41205263	41205456	-

41286190	41286644	-
41620566	41620738	-
46737342	46737474	-
22918408	22919479	-
49688741	49688917	-
50831743	50831878	-
69398359	69398776	-
1493131	1493302	-
23034811	23035363	-
23095276	23095506	-
23320543	23321065	-
35122051	35122369	-
35825982	35826129	-
45934537	45935012	-
34955267	34955767	-
35092758	35093634	-
35411631	35412345	-
47357206	47357975	-
47356584	47356979	-
44910825	44911026	-
44966218	44966442	-
56392335	56392848	-
63864298	63864502	-
63865143	63865364	-
59007610	59007765	-
63980817	63981236	63980970:63980981:+:CCACGAGGAGGC
63980292	63980744	-
62937763	62937987	62937994:62938005:-:GCCCCCTCGCGG
62937439	62937712	-
62937763	62937987	62937994:62938005:-:GCCCCCTCGCGG
62937439	62937712	-
63627322	63627553	-
63625959	63626299	-
3768100	3768286	-
13784737	13785072	-
363285	363481	-
543823	544678	-
5126210	5126649	-
1265263	1266052	-
32109319	32109848	-
32277154	32277483	-
37289422	37289789	-
33401285	33401755	-
33400934	33401267	-
38033404	38033624	-
38033745	38034142	38033754:38033765:-:GCCATCTTGTGG
38032851	38033369	-
33685469	33685694	-
38033404	38033624	-
38032851	38033369	-
38033745	38034142	38033754:38033765:-:GCCATCTTGTGG
33720149	33720567	-
38748805	38749018	-
38961882	38962094	-

2570674	2572465	-
3013533	3013734	-
2655223	2655522	-
12991518	12992121	-
13726587	13726748	-
32019089	32019217	-
9135703	9135931	-
9136337	9136681	-
9463034	9463446	-
154485838	154485990	-
47581738	47581884	-
72181889	72182044	-
101408055	101408257	-
49443881	49444226	-
11578221	11578581	-
11578090	11578217	-
4615564	4615910	-
67160063	67160300	-
67164914	67165047	-
67807069	67807376	-
69564157	69564506	-
49857749	49857877	49857828:49857839:+:CCGCCAGGGGGC
67660645	67660815	-
67660082	67660575	-
67660645	67660815	-
67660082	67660575	-
1772190	1772420	-
68843311	68843471	-
70524009	70524417	-
68245052	68245206	-
68244797	68245000	-
68245706	68245871	-
75000125	75000493	-
77191076	77191336	-
84820305	84820619	-
4768418	4768707	-
15949141	15949358	-
740913	741141	-
86554630	86554780	-
720545	720760	720773:720784:-:GCCCCCGGCTGG
675251	675381	-
674948	675100	674993:675004:-:GCCCCCTGGGGG
3400827	3401236	-
23557287	23557577	23557505:23557516:+:CCACCAGGGGGC
23557287	23557577	23557505:23557516:+:CCACCAGGGGGC
2776453	2776725	-
12803481	12803662	-
23149446	23150002	-
4473960	4474098	-
4425320	4425906	-
4426583	4426799	-
14632341	14632550	-
29679711	29679936	-
31032768	31033328	-

31105780	31106068	-
63833999	63834535	-
63834539	63834939	-
64387549	64387949	-
82985710	82986152	-
41972850	41973058	41972831:41972842:-:GCCCCCTTCTGG
42491079	42491346	-
48811002	48811406	-
48810302	48810632	-
28098942	28099115	28098962:28098973:+:CCACCAGGGGGC
50355133	50355519	-
44537483	44538124	-
44663149	44664365	-
40894979	40895194	-
41331961	41332326	41332166:41332177:-:GCCACCCGGTGG
45523193	45523406	-
45586781	45586986	-
28890144	28890406	-
28889206	28889970	-
89757268	89757462	-
96644411	96645184	-
98117072	98117421	-
143553816	143554005	-
143617616	143617757	-
22367847	22368115	-
11284040	11284550	-
30094937	30095064	-
30728076	30728306	-
25458908	25459082	-
26382908	26383153	-
48933884	48933987	-
48993539	48993803	48993477:48993488:+:CCTCCAGGGGGC
48994166	48994348	-
38619051	38619503	-
38831425	38831724	-
38899131	38899397	-
38899453	38899991	-
49113940	49114160	-
49453223	49453941	-
49452983	49453194	-
12806289	12806603	-
2236331	2236964	2236789:2236800:+:ACACCAGAGGGC
13103049	13103742	-
49876572	49877218	-
49877280	49877667	-
49929280	49930061	-
49929012	49929146	-
2977656	2977899	-
2783384	2784149	-
49817966	49818289	-
7919906	7920153	-
7921186	7921438	-
13774230	13774596	-
14031986	14032283	-

14137475	14137609	14137487:14137498:-:GCCCCCTTGAGG
14136711	14136973	-
50025802	50026281	-
55259105	55259729	-
15125499	15125729	-
39846758	39846986	-
39834202	39834587	-
34358928	34359425	-
40285773	40286065	-
4246687	4247305	-
46788287	46788649	-
3626800	3627139	-
47256138	47256398	-
3505798	3506055	-
10252022	10252216	-
41877795	41877964	-
41877998	41878196	-
10289244	10289669	-
51365902	51366147	-
17266916	17267243	-
10380746	10381382	-
10380143	10380399	-
10419646	10420085	-
41996461	41996594	-
42325042	42325286	42325306:42325317:-:GCCCTCCAGTGG
48445329	48445815	-
48363618	48364179	-
50723691	50723837	-
48256604	48256695	-
48255555	48256040	-
48614227	48614465	-
48614005	48614219	-
48740882	48741187	-
48740085	48740325	-
12723221	12723625	-
12887117	12887289	-
12887785	12888150	-
12886978	12887102	-
12881115	12881435	-
12880684	12880842	-
54160348	54160598	-
54159285	54160015	-
54115206	54115915	-
54115206	54115915	-
17859608	17859892	-
17860123	17860409	-
18001995	18002133	-
18438397	18438600	-
18438611	18438795	-
18918731	18919146	-
18918731	18919146	-
19033979	19034253	-
35545435	35545606	-
18557564	18557830	-

19321199	19321378	-
35040915	35041064	-
42242604	42243026	-
37906921	37907128	-
43755085	43755455	-
94004247	94004608	-
106285790	106286293	-
19708659	19708895	-
107168363	107169015	-
107564255	107564626	-
135211162	135211358	-
107743750	107744290	-
135977159	135977726	-
139109606	139110342	-
139108975	139109540	-
44606981	44607589	-
44848344	44848571	-
156639829	156640199	-
141737981	141738715	-
73739437	73739568	-
73738506	73738639	-
30026381	30026838	-
30026381	30026838	-
30478415	30478653	-
30594651	30594983	-
142855235	142855688	142855484:142855495:+:CCCCTAGGTGGC
143287672	143288198	-
143288759	143289306	-
76302169	76302463	-
99409202	99409640	-
99409202	99409640	-
100611837	100612399	-
6104401	6104628	-
128409499	128409693	-
100539225	100539377	-
129610940	129611488	-
12210702	12211293	-
149837934	149838606	-
149838624	149839211	-
16644644	16645017	-
150322788	150324948	-
42931878	42932147	-
42931878	42932147	-
43926523	43926769	-
66920766	66921192	-
73557857	73558085	-
74288897	74289142	-
98119351	98119557	-
98119612	98119815	-
98193072	98193365	98193205:98193216:+:CCAGCCGGGGGC
98192201	98192375	-
99104528	99104871	-
128190561	128191975	-
127877976	127878154	127877870:127877881:+:CCCCCAGGTGGC

127878299	127878618	-
127877065	127877472	-
470354	470528	-
35749916	35750085	-
137028792	137029026	-
38069776	38070091	-
13302438	13302609	-
119081088	119081269	-
17201639	17201860	-
17200921	17201274	-
68900582	68901054	-
48305804	48306368	-
119892508	119892679	-
119892701	119892838	-
72626270	72626648	-
72625791	72626257	-
84328101	84328369	-
91798079	91798391	-
91798810	91798989	-
102242197	102242366	-
92290327	92290480	-
27241370	27241595	-
103918558	103918816	103918664:103918675:-:GCCTCCTAGAGG
5684096	5684735	-
58385381	58385687	-
59906906	59907234	-
55408242	55408589	-
55384628	55384851	-
68332361	68333059	-
94402299	94402442	-
29294171	29294490	29294478:29294489:-:GCCATCTGCTGG
39980489	39980850	-
40139783	40139995	-
65100385	65100562	-
58518233	58518725	-
59565085	59565701	-
59893527	59893854	-
46922788	46923157	-
47940947	47941739	-
59893527	59893854	-
48101558	48101697	-
16217029	16217357	-
4902905	4903032	-
5420075	5420272	-
63843159	63843367	-
19377988	19378167	-
31901641	31901801	-
32350072	32350281	-
78781250	78781404	-
42535532	42535810	-
42535289	42535421	-
42536675	42536810	-
42744264	42744486	-
49969477	49969620	-

42980957	42981060	-
42980418	42980579	-
50372688	50373368	-
44123897	44124268	-
44899028	44900053	-
68512268	68512493	-
2030506	2030624	-
28335381	28335754	-
28335381	28335754	-
28576816	28577590	28577311:28577322:+:CCCCGAGGGGGC
28952151	28952585	-
55636817	55637150	-
141220759	141221057	-
143335882	143336027	-
108620382	108620709	-
7068006	7068238	-
109815116	109815508	-
24584595	24584747	-
25160426	25160839	-
152536350	152536748	-
13545350	13545497	-
2934974	2935108	-
2935281	2935563	-
39528288	39528461	-
39528103	39528268	-
39527780	39528051	-
20363508	20363698	-
20364084	20364466	-
20364488	20364803	-
116788061	116788297	-
47642745	47643071	-
47643436	47643739	-
47767583	47767793	-
47767301	47767440	-
64888819	64889430	-
64887889	64888227	64888059:64888070:-:GCCCCCTAGTGG
59616406	59616746	-
68004213	68004384	68004270:68004281:-:GCCACCTCCTGG
64723547	64723689	64723636:64723647:-:GCCCTCTGCTGG
60841643	60841991	-
60841181	60841615	-
60913971	60914448	-
90222613	90222842	-
72112155	72112893	-
94128551	94128811	-
116822679	116822838	-
10541237	10541786	-
117986442	117986583	-
102346681	102346922	-
118924555	118924850	-
63939569	63939787	-
3057778	3058078	-
2302535	2302780	-
67505759	67505964	-

17077341	17077939	-
67483086	67483508	-
3797959	3798310	3798255:3798266:+:CCACCAAAGGGC
68030076	68030409	-
68030896	68031193	-
68121684	68121908	-
121888178	121889016	-
6827378	6827524	-
49568035	49568425	-
107761325	107761478	-
107760410	107760525	-
108633672	108634020	-
30755228	30755493	-
30754052	30754220	-
51083563	51083720	-
56333648	56334013	-
56333064	56333319	-
56646599	56646776	-
56687518	56687744	-
122527032	122527755	-
52948220	52948375	-
52949669	52949882	-
57458988	57459468	-
10613515	10613678	-
2812378	2812536	-
2812869	2813086	-
110449739	110449993	-
110469212	110469568	110469302:110469313:+:CCCCGAGGGGGC
110468260	110468751	-
110502084	110502633	-
110501272	110501534	-
112108730	112108925	-
122974611	122974901	-
122975060	122975369	-
123633157	123633467	-
123633157	123633467	-
123601603	123601992	-
54301130	54301449	-
67268630	67269157	-
68686417	68687089	-
70243051	70243513	-
69239172	69239362	-
75511350	75511777	-
6724213	6724422	-
6851554	6851944	-
101696826	101697208	-
6851554	6851944	-
118375966	118376517	-
22544582	22544884	-
120437606	120437747	-
120437606	120437747	-
106774162	106774533	-
120470011	120470412	-
24666405	24666851	-

116570886	116571562	-
10722642	10722867	-
10694520	10695304	-
87322871	87323173	-
125956581	125957117	125956768:125956779:-:GCCCTCTAGTGG
149963751	149964121	-
152983733	152984038	-
153003178	153003493	-
153002000	153002423	-
35342107	35342513	35342387:35342398:+:GCACCAGAGGGC
36027345	36027647	-
49636363	49637134	-
36593581	36594579	-
159762091	159762426	-
159789703	159790816	-
53061758	53062090	-
89819952	89820130	-
89819624	89819772	-
39115039	39115687	-
20400824	20401580	-
24666405	24666851	-
132814086	132814487	-
24721338	24722121	-
24774709	24775083	-
108261288	108261703	-
108260181	108260484	-
145735604	145735969	-
33199704	33200136	-
33200474	33201155	-
33418521	33419034	-
33417943	33418376	-
162727747	162728006	-
41735522	41736059	-
42050765	42051097	-
170553632	170554192	-
42747603	42747798	-
42983808	42984199	-
43075894	43076157	-
43181669	43181875	-
43182486	43182701	-
43182704	43183189	-
43229389	43230012	-
43228936	43229121	-
693059	693226	-
693292	693564	-
75284840	75285102	-
2245765	2246224	-
43769455	43770087	43769631:43769642:+:TCACTAGGGGGC
4020821	4021295	-
44219046	44219302	-
132488826	132488950	-
140691255	140691711	-
6712470	6712855	-
43313489	43314127	-

138178192	138179072	-
138178192	138179072	-
44808629	44809206	-
138575536	138576204	-
138575033	138575489	-
140564651	140564937	-
140564430	140564643	-
140639016	140639287	-
140647707	140647991	-
75511778	75512309	-
176447727	176448045	-
176448062	176448291	176448239:176448250:-:GCCCTCTGGCGG
141245772	141245903	-
157142684	157142939	-
180072044	180072308	-
180495071	180495332	-
80655341	80655530	-
163437977	163438136	-
33441215	33441507	-
34915484	34915812	-
132556620	132557101	-
132556620	132557101	-
37371341	37372053	-
134226263	134226601	-
134648359	134648722	-
134648979	134649184	-
40755882	40756194	-
40756207	40756376	-
168486267	168486589	-
135399387	135399608	-
146446707	146447465	-
149551466	149551937	-
150000263	150000889	-
173887586	173888281	-
177474847	177475036	-
177023320	177023501	-
160399506	160399784	-
53891975	53892214	-
138187258	138187425	-
167734641	167735042	-
48847810	48847932	-
48846989	48847114	-
100709449	100709567	-
101685934	101686295	-
197960431	197961037	-
196943192	196943403	-
196942366	196942680	-
47282345	47282814	-
47281928	47282281	-
47476381	47477028	-
47475944	47476322	47476052:47476063:-:GCCCTCTGGTGG
131502338	131502585	-
50568045	50568280	-
50610819	50610995	-

50610819	50610995	-
39051812	39052154	-
39107530	39107885	-
51984045	51984182	-
51982811	51983192	-
51982483	51982756	51982628:51982639:+:CCTGCAGGGGGC
40308939	40309790	-
40308648	40308935	-
51973911	51974266	51974097:51974108:-:GCCCTCTTGTGG
51975290	51975450	-
183635261	183635720	-
184361100	184361296	-
96740390	96740691	-
231482731	231482853	231482906:231482917:+:CCAGCAGGTGGC
97662815	97663524	-
24075963	24076472	-
24972141	24972412	-
24971061	24971397	-
152175662	152176214	-
157875246	157875537	-
27356112	27356961	-
27370310	27370664	-
161308459	161308882	-
27370310	27370664	-
27409594	27409860	-
27409948	27410248	-
1438166	1438500	-
74482644	74482797	-
74483280	74483468	-
74529212	74529380	-
55112483	55112896	-
55111639	55112478	-
74529212	74529380	-
98608398	98608531	-
216696273	216696403	-
61888564	61888827	-
63050816	63051028	-
86563403	86563902	-
102064190	102064624	-
102065224	102065487	-
105438857	105439054	-
219218773	219218971	-
219245532	219245661	-
8679495	8679680	-
9842941	9843272	-
10689849	10690055	-
170928286	170928700	-
171433315	171433997	-
171434226	171434699	-
171999740	172000460	-
135985742	135986240	-
135985475	135985684	-
135984973	135985328	-
38751194	38751962	-

64989291	64989518	-
175167601	175168359	-
43595892	43596360	-
15526294	15527190	-
174998557	174999807	-
52927562	52927749	-
52927854	52928001	-
3857250	3857494	-
54052332	54053031	-
54052332	54053031	-
6209350	6209537	-
180153690	180154547	-
108746212	108746966	108746505:108746516:+:CCGCTAGGGGGC
108747175	108747723	-
29181986	29182560	-
29180865	29181546	-
29230998	29231496	-
32292751	32293051	-
112619476	112619705	-
112619740	112620161	-
32816736	32817274	-
155261797	155262397	-
34928824	34929068	-
156054251	156054625	-
156055064	156055329	-
156501337	156501609	-
156501632	156501881	-
11060911	11061113	-
185156797	185157198	-
185157373	185157657	-
65421277	65421515	-
185317922	185318175	-
11926660	11926806	-
11979552	11980085	-
11980689	11981022	-
183471683	183472376	-
193058694	193059209	-
193059816	193060030	-
193060041	193060319	-
193060041	193060319	-
193059816	193060030	-
114581763	114582067	-
70410721	70411134	-
113759435	113760142	-
15975981	15976612	-
116570228	116570503	-
201022515	201023086	-
201171387	201171610	-
36089205	36089750	-
36464648	36465170	-
36463235	36463883	-
231337647	231338314	-
231240652	231241201	-
231528276	231528909	-

37691846	37692688	-
38860094	38860551	-
38858811	38859220	-
236794710	236795128	-
236795621	236795939	-
39789374	39789550	-
39902373	39902589	39902410:39902421:-:GCCACCTGGCGG
40160912	40161619	-
40783188	40783525	-
75723962	75724206	-
16981621	16981752	-
84479138	84479534	-
84573883	84574138	-
205121935	205122681	-
205775173	205775691	-
21346210	21346497	-
23559135	23559656	-
207752314	207752697	-
150321580	150321870	-
42767254	42767486	-
42765988	42766274	-
42958709	42959606	-
43358056	43358555	-
43946443	43947264	-
43974295	43974927	-
45550333	45551216	-
45551240	45551634	-
45521706	45522679	-
46133310	46133742	-
46174929	46175466	-
169367566	169368129	-
169485934	169486667	-
46339511	46339889	-
46340328	46341201	-
93179951	93180369	-
93345600	93345995	-
171485076	171485798	-
101025963	101026551	-
173477962	173478472	-
173824348	173825415	-
209827600	209828065	-
25232398	25233516	-
99969849	99970070	-
99970201	99970541	-
25818147	25818503	-
211675797	211676058	-
26530256	26530460	-
26431803	26432860	-
212791451	212792277	-
26692056	26693997	-
214602747	214603230	214603063:214603074:+:CCAGTAGAGGGC
27914436	27915417	-
27773666	27774007	-
52056611	52057236	-

52055222	52055777	-
158830096	158830911	-
158830915	158831185	-
136753042	136753292	-
47472151	47472283	-
47483891	47484462	-
47483543	47483703	-
119019147	119019343	-
119018230	119018676	-
200620462	200620814	-
201376984	201377175	-
200670211	200670723	-
78188246	78188471	-
70354766	70355124	-
145813864	145814496	-
137866108	137866624	-
137867539	137867866	-
21021125	21021249	-
113904529	113904911	-
113905159	113905532	113905256:113905267:-:TCCCTCTGGTGG
77076277	77076597	-
20650998	20651130	20651160:20651171:-:GCCCCCTGGTGG
196068465	196068703	-
134370623	134371371	-
9424212	9424394	-
9424212	9424394	-
196969231	196969345	-
236604275	236605066	-
108934514	108934878	-
128656596	128657533	-
121074386	121074822	-
120793343	120794392	-
120893838	120894452	-
125189873	125190135	-
125190376	125190616	-
122264962	122265450	122265374:122265385:-:GCCACCCAGTGG
99821704	99821929	-
99822318	99822464	-
123930597	123930968	123930661:123930672:-:GCCACCTGGGGG
63422567	63422719	-
72927015	72927244	-
75126931	75127185	-
77029361	77029497	-
90331789	90332005	-
73949888	73950044	-
46541820	46542100	-
27663442	27664019	-
27032773	27033101	-
27033392	27033621	-
33598421	33598633	-
95693648	95693790	-
168579877	168580320	-
4792283	4792490	-
149749756	149750275	-

149749756	149750275	-
140174718	140174965	-
140664543	140664918	-
175022992	175024000	-
173824348	173825415	-
170531932	170532247	-
170532309	170532675	-
159789703	159790816	-
146521815	146522214	-
146522226	146522397	-
146522552	146522835	-
109334031	109334367	-
109334031	109334367	-
27154106	27154233	-
27155489	27155680	-
28642533	28643803	-
41061156	41061379	-
41061156	41061379	-
31162313	31162667	-
138543582	138543783	-
138331476	138331803	-
139294223	139294488	-
139341667	139341986	139341766:139341777:-:GCTCTCTAGTGG
138464445	138464709	-
150602903	150603408	-
142595898	142596318	-
142596356	142596560	-
98515579	98515855	-
93569373	93569561	-
98644608	98644977	-
98644177	98644364	-
23069310	23069501	-
27491375	27491511	-
11859398	11859581	-
11273340	11273828	-
11273856	11274104	-
12064150	12064321	-
54101138	54101348	-
49707787	49708852	-
96336177	96336468	-
154627731	154627857	-
153344538	153344782	-
32971834	32972154	-
52927562	52927749	-
52927854	52928001	-
185044613	185044947	-
185157373	185157657	-
185156797	185157198	-
119665074	119665184	-
34439266	34439415	-
71839456	71840017	-
71840022	71840285	-
32013678	32014096	-
32072180	32072725	-

46406585	46407264	-
39978058	39978348	-
39458546	39459228	-
110963214	110964228	-
108963000	108963247	-
144332727	144332872	-
75511778	75512309	-
176361679	176362093	176361748:176361759:+:CCACTAGGTAGC
11797523	11797852	-
158168330	158168802	-
80205869	80206129	-
92831804	92832602	-
100132820	100133179	-
91022362	91022724	-
91021893	91022334	-
93179951	93180369	-
35800129	35801022	-
35801026	35801215	-
12686661	12686891	12686819:12686830:-:GCCCCCTACAGG
33076154	33076355	-
37904208	37904902	37904410:37904421:+:CCTGCAGGGGGC
135170460	135171138	-
135169697	135170243	-
138001357	138002508	-
73168554	73168915	-
73096603	73097037	-
68526947	68527178	-
69123312	69123951	-
50960320	50960749	-
51236036	51236226	-
51236814	51237300	-
26832095	26832395	-
14419529	14419798	-
14419237	14419399	-
52023747	52023883	-
54301130	54301449	-
49130059	49130866	-
47578784	47579134	-
46672796	46673155	-
47313581	47314146	-
110874184	110874476	-
99521143	99521766	-
171434226	171434699	-
165953951	165954365	165954241:165954252:+:CCACAAGGTGGC
151289826	151290094	-
159616188	159616638	-
121801047	121801439	-
109730275	109730611	-
41364060	41364854	-
57524949	57525147	-
47501258	47501779	-
58389684	58389989	-
59031613	59032249	-
43457356	43457817	-

49278562	49278993	-
49279006	49279313	-
49046804	49047092	-
50153922	50154493	-
50153309	50153701	-
49982008	49982582	-
45891258	45891436	-
45891050	45891255	-
7869697	7869970	-
73828778	73829087	-
43596199	43596834	-
13573531	13574079	-
2765212	2765519	-
34757383	34757933	-
43576153	43576604	-
43575409	43575689	-
43427658	43428349	-
42979087	42979874	-
2987388	2987634	-
2988301	2988925	-
27375015	27375413	-
34426005	34426449	-
34425651	34425834	-
34425279	34425487	-
27132715	27133148	-
42745842	42745999	-
43629304	43630479	-
43013204	43013413	-
43013204	43013413	-
7389587	7389809	-
5003896	5004324	-
5004395	5004681	-
5003387	5003730	-
17706843	17707352	-
17706206	17706399	17706314:17706325:-:GGCCCCTGGTGG
17705977	17706159	-
74364785	74365224	-
62556320	62556545	-
10010920	10011048	-
58630032	58630693	-
67226960	67227320	-
58735164	58735338	-
58733718	58733931	-
95300798	95300945	-
44141687	44141989	-
44142538	44142705	-
132491398	132491543	-
132490664	132490850	-
119871845	119872065	-
119871845	119872065	-
50311341	50311532	-
2962919	2963240	-
75260844	75261105	-
75205454	75205795	-

75289395	75289871	-
75270726	75270997	-
75270423	75270668	75270515:75270526:+:CCAGCAGGAGGC
132406734	132407171	-
132669428	132669999	-
132670050	132670414	-
132670050	132670414	-
132669428	132669999	-
132582118	132582289	-
112542288	112542666	-
6394101	6394343	-
6361328	6361490	-
123733054	123733233	-
123732169	123732318	-
38853790	38854022	-
62783530	62783914	-
6736578	6736826	-
45467451	45467654	-
45692475	45692738	-
45692761	45693275	-
45496072	45496356	-
45507103	45507633	-
45863068	45863549	-
45862318	45863027	-
2470378	2470971	-
3819782	3820098	-
3820226	3820983	-
18288226	18288477	-
16729791	16730172	-
17968825	17968960	17968712:17968723:+:CCACGAGGAGGC
17968632	17968818	17968713:17968724:+:CCACGAGGAGGC
3209101	3209325	-
3185623	3185925	-
23344657	23345437	-
31604147	31604433	-
34516266	34516494	-
35277236	35277340	-
50359497	50359747	-
35641299	35641875	-
45011216	45011911	-
45011955	45013297	-
45011216	45011911	-
113208654	113208778	-
113208036	113208223	-
34404442	34404586	-
35628775	35629146	-
35648167	35648440	-
65386065	65386621	-
64317549	64318108	-
64318164	64318478	-
49665013	49665171	-
49665013	49665171	-
42779757	42779911	-
42833310	42833475	-

4314215	4314440	-
4314030	4314161	4314077:4314088:-:GCCCCCTCCTGG
27671760	27672326	-
55412001	55412144	-
32142015	32142208	-
158856286	158856719	-
131095665	131096403	-
131124888	131126574	-
153901530	153901661	-
4124233	4124369	-
101408055	101408257	-
179678487	179678639	-
179678644	179678809	-
17309910	17310059	-
17309201	17309364	-
193593693	193593908	-
193592601	193593355	-
66302521	66302882	-
68609969	68610466	-
150322374	150322637	150322418:150322429:-:GACCTCTGGTGG
150321733	150322312	-
932722	932977	-
25858880	25859375	-
9834637	9835026	-
9835792	9835923	-
9827718	9827942	-
19251308	19252031	-
19210419	19210631	-
20787548	20788138	-
16572757	16573083	-
16888699	16888915	-
2980584	2980774	-
690341	690625	-
788620	788877	-
4968389	4968591	-
4830781	4831065	-
82358312	82358765	-
219277724	219277973	-
77536539	77536789	-
92528603	92529221	-
91880486	91880907	91880791:91880802:-:GCCCCCTCGTGG
92528603	92529221	-
55346041	55346267	-
20319999	20320323	-
20320428	20320714	-
20079554	20080399	-
21640775	21644465	-
39520139	39520438	-
37020092	37020376	-
37020092	37020376	-
37244155	37244287	37244076:37244087:+:CCAGTTGGGGGC
37953245	37953370	-
112206464	112206680	-
128862273	128862922	-

108569640	108570036	-
128738392	128739613	-
130069871	130070621	-
123557868	123558362	-
123557096	123557861	-
176269762	176270216	-
189763054	189763271	-
171922516	171923189	-
9334044	9334266	-
41116435	41117038	-
60477996	60478862	-
58770633	58771060	-
52569184	52569432	-
129440215	129440565	-
56063837	56064560	-
5420075	5420272	-
7583521	7583895	-
132775031	132775253	-
48716273	48716775	-
43758387	43758887	-
10653737	10654253	-
10569371	10569682	-
112922086	112922212	-
76141275	76141408	-
76501716	76501972	-
38105510	38105763	-
20185603	20186263	-
19321199	19321378	-
88977236	88977441	-
88977999	88978401	-
88976686	88976872	-
169751506	169751836	-
169724853	169725593	-
11529148	11529420	-
11434995	11435364	-
3099799	3100203	-
44845499	44845982	-
44915040	44915240	-
38403095	38403336	38403123:38403134:+:CCGCCAGGGGGC
17334699	17335015	-
17333920	17334595	-
17405192	17405434	-
17468763	17468887	-
17556115	17556279	-
17309201	17309364	-
17309910	17310059	-
17292723	17293022	-
17511974	17512195	-
2327756	2327880	-
158232877	158233140	-
107028210	107028424	-
107028595	107028822	-
158999633	159000294	-
158999064	158999318	-

6278880	6279057	-
240025385	240025512	-
17688287	17688402	-
18418508	18419147	-
18373698	18374648	-
18375372	18375657	-
18322381	18322762	-
18282283	18282597	-
49155805	49156400	-
49154667	49155034	-
63980292	63980744	-
152259	152393	-
157011276	157011577	-
26233627	26234152	-
26234232	26235391	-
62426720	62426843	-
130693833	130694231	-
58555139	58555380	-
58543830	58543989	-
636332	636764	-
636144	636323	-
635573	635717	-
47113364	47114100	-
47019356	47019635	-
40191025	40191239	-
3796445	3796620	3796505:3796516:+:CCACCAGGGTGC
3796855	3797111	3796910:3796921:+:CCAGGAGAGGGC
28258696	28259293	-
27934930	27935148	-
28235828	28236602	-
28236608	28237272	-
28328686	28329286	-
9140133	9140501	-
9140525	9140754	-
10105782	10106310	-
10232074	10232208	-
154763398	154763616	-
67718407	67718564	-
149546156	149546523	149546356:149546367:+:CCACCAGGGGGC
149545266	149545588	149545300:149545311:-:GCCCTCTTCTGG
149718129	149718755	-
43619934	43620159	-
43618941	43619625	-
89658337	89658577	89658370:89658381:+:CCACCCGGGGGC
89657772	89657882	-
177378832	177379741	-
177446027	177446261	-
177446378	177446630	-
40039328	40040484	-
17074717	17075181	-
15601397	15601926	-
15205662	15206112	-
15206244	15206576	-
15098870	15099418	-

14402023	14402289	-
14403097	14403512	-
50333526	50333919	-
50334081	50334369	-
50332556	50332810	-
50329649	50329837	-
132258432	132258728	-
42608986	42609199	-
42998466	42998735	-
140647707	140647991	-
140400991	140401774	-
140402069	140402345	-
140402353	140402570	-
133253467	133253826	-
1309793	1309909	-
1310227	1310354	1310190:1310201:+:CCACCAGGTGGC
70270276	70270506	-
1528385	1528760	-
1528028	1528349	1528058:1528069:-:GCCTCCTGGTGG
2963833	2963999	-
2154959	2155397	-
145919038	145919682	-
145859670	145859987	-
26913881	26914666	-
42843081	42843559	-
32971834	32972154	-
58199826	58199946	-
14007244	14007427	-
14006467	14006951	-
13905792	13906139	-
13905792	13906139	-
12664119	12664737	-
13420768	13421582	-
13419751	13420049	-
711328	712138	-
6235196	6235484	-
6604055	6604234	-
86106427	86106657	-
130870816	130871016	130871028:130871039:+:CCAACAGGGGGC
6908566	6908872	-
99394042	99394417	-
99425051	99425829	-
54759208	54759478	-
70688060	70688626	70688235:70688246:-:GCCCTCTATTGG
75786160	75786526	-
75785114	75786046	-
75783903	75784984	-
75785114	75786046	-
7307087	7307229	-
7314708	7314880	-
7219610	7219831	-
98117072	98117421	-
134904403	134904762	-
28854582	28854970	-

28855041	28855302	-
69339521	69339661	-
69339930	69340098	-
70686730	70686913	-
2840370	2840507	-
5126210	5126649	-
23350347	23350555	-
155934163	155934824	-
156678019	156678484	156678213:156678224:-:GCCCTCTGGCGG
68903658	68903978	-
45499372	45500589	-
43969958	43970337	-
45583398	45584165	-
45340276	45341088	-
44522192	44522428	-
59940420	59940819	-
16237014	16237560	-
15526294	15527190	-
150758722	150759233	-
20902846	20902991	-
204494173	204494368	-
205211481	205212308	-
203273097	203273489	-
44989483	44989606	-
55339395	55339688	-
55280858	55281147	-
64165034	64165331	-
63505925	63506169	-
15889051	15889396	-
30506559	30506742	-
140672692	140672865	-
140673195	140673747	-
151085288	151085881	-
151085071	151085260	-
92145693	92145917	-
146182406	146183101	-
81685956	81686087	-
82358312	82358765	-
30082842	30083127	-
30082516	30082792	-
69166646	69166953	-
69167069	69167261	-
10164790	10165029	-
9362662	9363044	-
9363234	9363554	-
37101214	37101323	-
35546032	35546540	-
34101774	34102215	-
109620232	109620420	-
109619083	109619819	-
108691704	108692475	-
109283435	109284101	-
109282494	109283106	-
112466024	112466274	112466306:112466317:-:GCCCCCTGGTGT

110407463	110408048	-
113905159	113905532	113905256:113905267:-:TCCCTCTGGTGG
113904529	113904911	-
6866253	6866519	-
6866253	6866519	-
18394495	18394702	-
193121746	193122429	-
201955146	201956024	-
47215083	47215607	-
47248724	47249414	-
132023867	132023984	-
31065926	31066260	-
31065411	31065906	-
31815808	31816350	-
33430195	33430360	-
37691846	37692688	-
35808914	35809259	-
52055222	52055777	-
52056611	52057236	-
52403613	52404368	-
31102151	31102424	-
36129660	36129999	-
36829166	36829763	-
57530267	57530744	-
47349018	47349360	47349145:47349156:+:CCTCTAGAGGGC
61792437	61792989	-
61792437	61792989	-
55396697	55397005	-
100588792	100588962	100588834:100588845:-:GCCCCCTGCAGG
99200520	99200768	-
110713169	110713322	-
112707208	112707937	112707519:112707530:+:CCACAAGATGGC
111092030	111092543	-
83708137	83708515	-
74952546	74952684	-
87726395	87726600	-
85741198	85741357	-
78297275	78297484	-
157142684	157142939	-
77798225	77798499	-
108569640	108570036	-
128455228	128455821	-
105532032	105532364	-
105532375	105532752	-
116210918	116211418	-
73461766	73461915	-
87589889	87590344	-
33891715	33892400	-
55829707	55829948	-
56266815	56266931	-
56333648	56334013	-
56333064	56333319	-
54280324	54280705	-
54280797	54281421	-

56468735	56469048	-
143843279	143843506	-
109382821	109383474	-
108294811	108295410	-
135498280	135499127	-
135497295	135497896	135497511:135497522:+:CCAGGAGAGGGC
48270233	48270804	-
74483280	74483468	-
74482644	74482797	-
68808475	68808626	-
66751699	66751962	-
67160063	67160300	-
67247834	67248183	-
247078721	247078886	-
230641879	230642203	-
230642503	230642780	-
229559144	229559453	-
182392488	182392762	-
182838802	182839241	-
179954249	179954750	-
182789884	182790282	-
172443915	172444298	172444150:172444161:-:GCCCCCTCCTGG
230712401	230712637	-
99490475	99490665	-
99154828	99155147	-
99155152	99155522	-
96266211	96266540	-
96266575	96266798	-
108448641	108448810	-
105337004	105337482	-
107685627	107686072	-
105107735	105107956	-
37986564	37988154	-
48094244	48094354	-
49443881	49444226	-
2403129	2403281	-
22726470	22726756	-
45111524	45112128	-
45111269	45111495	-
45089592	45089708	-
50479091	50479428	-
45051024	45051472	-
58006720	58008003	-
63600575	63601018	-
63549752	63550252	-
179604468	179604709	-
179604468	179604709	-
185938180	185938443	-
185937780	185938177	-
185936985	185937445	-
159712594	159712780	-
157257344	157258049	-
220046474	220047026	-
220045837	220046204	-

150067197	150067743	-
215568149	215568413	-
213050433	213051326	-
127811124	127811467	-
130342365	130342985	-
128028353	128028496	-
130342365	130342985	-
27155489	27155680	-
127784928	127785060	127785132:127785143:-:GCCCCCTGGCGG
127786411	127786660	-
110257074	110257497	-
128455595	128455772	-
128456113	128456246	-
104094073	104094256	-
127396500	127396817	-
127396218	127396375	-
120580171	120580890	-
113275313	113275663	-
114587480	114588055	-
124852836	124853134	-
97632831	97633060	-
124415239	124415754	-
97922000	97922550	-
125199479	125199715	-
125200843	125201207	-
97696628	97696884	-
97632831	97633060	-
119855641	119855791	-
123041833	123042147	-
127736134	127736544	-
37485936	37486574	-
26947303	26947430	-
36486650	36486754	-
34989260	34989422	-
37422457	37422722	-
37421835	37422150	-
42928824	42929502	-
41546044	41546301	-
36875566	36876114	-
17988530	17988676	-
16237512	16237691	-
16238944	16239379	-
16239419	16239725	-
43516832	43517355	-
43516409	43516740	-
41786827	41787452	-
3117824	3118360	-
3118615	3118812	-
33712010	33712304	-
31158471	31158726	-
31159046	31159362	-
30743093	30743528	-
30741881	30742378	-
30716484	30718022	-

30690267	30690799	-
30907807	30908406	-
42050765	42051097	-
17601081	17601626	-
10694520	10695304	-
77127755	77127931	-
75352057	75352318	-
75350843	75351365	-
75350637	75350775	-
83071876	83072137	-
83072217	83072608	-
83071647	83071871	-
76671300	76671444	-
78575040	78575451	-
71927903	71928342	-
54134465	54134847	-
63039345	63039588	-
55772456	55772706	-
103092237	103092528	-
119030807	119031006	-
119031016	119031191	-
111878954	111879245	-
111879265	111880057	-
44427104	44427546	-
44427559	44428118	-
58933792	58934145	-
41774468	41774684	-
41331961	41332326	41332166:41332177:-:GCCACCCGGTGG
41402346	41402743	-
42548661	42549092	-
43369980	43370362	-
41493424	41493601	-
56918203	56918358	-
56917852	56918135	-
55196736	55197119	-
40764042	40764230	-
88891310	88891718	-
77979292	77979694	-
100249337	100249856	-
27489359	27490217	-
27085590	27085970	-
55693557	55693862	-
37196861	37197056	-
37196118	37196671	-
65130693	65130977	-
27133888	27134146	-
24792577	24793039	24792917:24792928:+:CCAGCAGGGGGC
24793047	24793571	-
73168554	73168915	-
72216637	72217172	-
70671486	70672322	-
70673523	70673932	-
68560057	68560404	-
68471695	68472468	-

216412318	216412564	-
169824761	169825336	169824989:169825000:-:GCCACCTGGGGG
169584077	169584623	-
169573009	169574029	-
174249072	174249317	-
174247504	174248049	-
174396084	174396291	-
174395282	174395849	-
186590828	186591170	-
122793844	122794014	-
50424650	50424985	-
51829228	51829470	-
65212175	65212357	-
74434951	74435173	-
74434028	74434482	-
74460777	74462207	-
88379219	88379348	-
83034648	83034976	-
82900648	82900816	-
80266737	80267016	-
76148293	76148677	-
78775487	78775845	-
75724810	75725065	-
75724810	75725065	-
109703049	109703229	-
109433513	109433693	109433563:109433574:+:CCTGCAGGGGGC
32755024	32755386	-
22624613	22625081	-
22625547	22625953	-
71685978	71686413	-
95858770	95859032	-
103965199	103965924	-
113421984	113422542	-
56222297	56222510	-
32315400	32315639	-
49111433	49111630	-
123365154	123365763	-
122266166	122266727	-
121802717	121803166	-
113208036	113208223	-
113208654	113208778	-
23058026	23058191	23058211:23058222:-:GCCCCCTGGTGG
63641380	63641734	-
89954428	89954766	-
43824610	43825152	-
56918203	56918358	-
56917852	56918135	-
45023465	45023579	-
43369980	43370362	-
59689506	59689748	-
40439630	40439892	-
40440390	40440486	-
75368268	75368718	-
75368029	75368265	-

72686521	72686897	-
73995390	73995540	-
74201943	74202082	-
74202829	74202969	-
89243550	89243782	-
89087497	89088055	-
90868206	90868967	-
90869043	90869549	-
4788771	4788901	-
31508600	31508952	-
14632341	14632550	-
81096643	81096870	-
81095843	81095994	-
84116754	84117144	-
84117415	84117917	-
667891	668113	-
3400827	3401236	-
1965015	1965199	-
1963914	1964254	-
89873043	89873328	-
89872532	89872681	-
16217029	16217357	-
67807069	67807376	-
55751156	55751319	-
44071032	44071237	-
59618998	59619274	-
60393145	60393429	-
60677317	60677904	-
59707276	59707523	-
12377770	12378105	-
11980236	11980415	-
36129660	36129999	-
23502998	23503465	-
23504267	23504621	-
4834298	4834470	-
80036481	80036613	-
81871369	81871827	-
81871907	81872374	-
81890437	81890641	-
82752060	82752187	-
82717166	82717300	-
82457967	82458482	-
82458681	82458872	-
80544697	80545177	-
82519066	82519319	-
75896494	75896627	-
79839880	79840053	79839929:79839940:+:CCGGCAGGGGGC
39668575	39668719	39668599:39668610:-:GCCCCCTCGCGG
2739308	2739540	-
507063	507486	-
41878374	41878626	-
4791267	4791818	-
6772786	6772935	-
17405192	17405434	-

5790501	5791446	-
41310292	41310687	-
41349850	41349982	-
327307	327605	-
326983	327231	-
33324204	33325269	-
31659398	31659910	-
44349448	44349567	44349459:44349470:-:GCCCGCTGGTGG
65524550	65524685	-
65525735	65525989	-
36156797	36157042	-
32393225	32393808	-
104796157	104796287	-
104796368	104796487	-
47113364	47114100	-
48513585	48513727	-
45079036	45079402	-
240565599	240565839	-
240586390	240586519	-
50025802	50026281	-
50476517	50476629	-
49422543	49423116	-
50414632	50415028	50414937:50414948:-:GCCCCCTCGTGG
49486536	49486764	-
49487422	49487808	-
51108181	51108720	-
51107623	51107896	-
49590301	49590503	-
10472011	10472912	-
10398217	10399008	-
26169392	26169701	-
35718915	35719496	-
35557196	35557797	-
127880800	127881143	-
27366614	27367429	-
26889455	26889997	-
27341888	27342142	-
27341512	27341723	-
27342560	27343369	-
27233611	27234092	-
21937636	21938079	-
21936164	21936651	-
67430386	67431391	-
31576298	31576437	-
31576700	31576831	-
44774950	44775948	-
44776134	44776296	-
44739322	44740210	-
46616898	46617123	-
93078894	93079300	-
116667995	116668293	-
110031602	110031883	-
109426227	109426935	-
109248954	109249364	-

145910143	145910348	145910253:145910264:+:CCTCCAGGGGGC
165698191	165698442	-
165697929	165698001	-
169367566	169368129	-
166838760	166839312	-
167937331	167937810	-
167935978	167936773	-
167935379	167935840	-
167935978	167936773	-
165630868	165631424	-
167914076	167914300	-
176207282	176207948	-
161165756	161166462	-
161167121	161167347	-
161505244	161505753	-
161117477	161117884	-
161159037	161159594	-
156749522	156749870	-
156728114	156728484	-
156740974	156741572	-
156728114	156728484	-
156705913	156706217	156706127:156706138:-:GCCCCCTGCTGG
156767064	156767661	-
179881582	179882965	-
219173780	219174128	-
150507775	150507985	-
151281360	151281879	-
150487080	150487240	-
150487335	150487670	-
150926088	150926281	-
150235675	150236112	-
150982072	150982392	-
150629127	150629385	-
150877307	150877458	-
151070194	151070697	-
212034663	212035665	212035282:212035293:+:CCACCTGGTGGC
206634393	206635747	-
206611961	206612696	-
212034663	212035665	212035282:212035293:+:CCACCTGGTGGC
223846300	223846680	-
223844549	223845437	-
154325184	154325326	-
154326236	154326574	-
155050066	155050430	-
155050440	155051008	-
154220045	154220702	-
154272261	154272565	154272340:154272351:-:GCCCTCTGGCGG
155077804	155078385	-
154220045	154220702	-
153727540	153727908	-
155301144	155301503	-
155300764	155300957	-
231240652	231241201	-
230978802	230979378	-

243255128	243255589	-
243255710	243256648	-
224330095	224330654	-
225999411	226000513	-
225998509	225999385	-
228140365	228140643	-
227318982	227319482	-
224435102	224435285	-
9004509	9004880	-
225923404	225923666	-
203025636	203026464	-
202161525	202161807	-
202161214	202161515	202161336:202161347:-:GCCCCCTGCAGG
202160777	202161081	-
202145044	202145619	-
42168680	42169151	-
47176662	47177390	-
47176197	47176645	-
55231941	55232810	-
96266211	96266540	-
96266575	96266798	-
95165335	95165805	-
96305918	96306389	-
112721292	112721449	-
112255004	112255179	-
99154828	99155147	-
99155152	99155522	-
135741225	135741644	-
127858475	127858605	-
174395282	174395849	-
174396084	174396291	-
176002366	176002629	-
169694187	169694581	-
169694932	169695314	-
4467095	4467595	-
238240237	238240368	-
231781452	231781786	-
9704398	9704567	9704625:9704636:+:CCAGTAGGCGGC
11846753	11847108	-
218398169	218398329	-
219498309	219498495	-
219497963	219498180	-
15426823	15427242	-
39154236	39154463	-
37243087	37243438	-
37243480	37244027	-
37862637	37862864	-
56316247	56316563	-
150546433	150546963	-
194633693	194633943	-
197749627	197749823	-
197749268	197749590	-
196337836	196338792	-
196337461	196337745	-

184134044	184134426	-
184135023	184135526	-
184250224	184250518	-
53378078	53378331	-
993913	994098	-
992618	993275	-
121823066	121823397	-
118685487	118685643	-
119212834	119213024	-
163166884	163167024	-
169010113	169010397	-
10440975	10441592	-
10352845	10353398	-
40835233	40835683	-
40834873	40835224	-
36150838	36151188	-
81750968	81751234	-
76402887	76403061	-
69093680	69093893	-
115841481	115841878	-
144170481	144170758	-
134758334	134758529	-
134759035	134759440	-
148383514	148384075	-
173615627	173616172	-
175796938	175797122	-
13328591	13329017	-
13327991	13328315	-
5260658	5260919	-
181222025	181222664	-
181221732	181222011	-
176388429	176388895	-
47309761	47309995	-
49463020	49463768	-
43307946	43308077	-
42879194	42879690	-
42879926	42880462	-
44265159	44265330	-
87589889	87590344	-
132798973	132799257	-
136249785	136250712	-
158644502	158644784	-
158536124	158536298	-
35268290	35268737	-
12403785	12404276	-
1137292	1137463	-
65084525	65084827	-
44885617	44886167	-
112790477	112790789	-
100427967	100428615	100428287:100428298:+:CCACCCGAGGGC
134986389	134987293	134986725:134986736:-:GCCCCCTGCGGG
139340154	139342541	-
140673195	140673747	-
72571825	72574490	-

48521421	48521652	-
71532639	71533028	-
27772202	27773119	-
28890144	28890406	-
28889206	28889970	-
37762417	37762751	-
41529304	41529560	-
80029619	80029932	-
97644445	97644742	-
109539979	109540432	-
100150119	100150742	-
116765522	116766170	-
37119582	37120397	-
112717606	112717967	-
121370577	121370757	-
121369714	121369973	-
122264962	122265450	122265374:122265385:-:GCCACCCAGTGG
113401425	113401741	113401509:113401520:+:CCACGAGGGGGC
113340242	113340385	-
133336471	133336660	-
133336077	133336431	-
133348203	133348486	-
133417946	133418407	-
133348203	133348486	-
128149212	128149425	-
127450887	127451280	-
137578856	137579191	-
15860410	15860642	-
16817018	16817238	-
17454618	17455219	-
63132710	63133245	-
78029083	78030097	-
72354486	72355162	-
102132451	102132918	102132628:102132639:+:CCACTAGGGGGT
3797959	3798310	3798255:3798266:+:CCACCAAAGGGC
6926392	6926627	-
32583596	32583810	-
57335828	57336365	-
46936552	46936798	-
47176117	47176411	-
77994991	77995410	-
108223018	108223405	-
108222317	108222826	-
108222317	108222826	-
108223018	108223405	-
72103333	72103766	-
61333469	61333985	-
61362586	61362940	-
62611770	62611982	-
62611770	62611982	-
62622163	62622504	-
125625255	125625495	-
125625661	125625908	-
117178410	117178843	-

33662308	33662437	-
36773610	36774001	-
36773190	36773442	-
35954307	35954669	-
35953480	35953778	-
64206189	64206875	-
64206886	64207644	-
65122265	65122540	-
65122265	65122540	-
30091821	30092234	30091993:30092004:+:CCACGCGGGGGC
30092663	30093090	-
30053022	30053204	-
30053022	30053204	-
29995894	29996234	-
29995655	29995867	-
30895703	30896118	-
94973411	94973617	-
36829166	36829763	-
39265966	39266183	-
110871442	110871699	-
176195202	176195317	-
69961974	69962093	-
111926784	111926933	-
111926355	111926745	-
112024575	112024969	-
112025075	112025393	-
112073942	112074338	-
112073942	112074338	-
112086676	112086907	-
112086325	112086647	-
112226046	112226284	-
124916940	124917879	-
138241596	138242079	-
25062079	25062818	-
25061696	25062063	-
2004419	2004569	-
24494202	24494483	-
110501272	110501534	-
110502084	110502633	-
77925809	77926067	-
98930631	98930884	-
677264	677629	-
78046201	78046480	78046368:78046379:-:GCCCTCTGCTGG
78080753	78080895	-
78079560	78079956	-
78079246	78079552	-
108748392	108748740	-
12195796	12196206	-
129093170	129093801	-
129093170	129093801	-
134253466	134253601	-
134253466	134253601	-
7788049	7788187	-
184734070	184734563	184734361:184734372:-:GGCCCCTTGTGG

185143097	185143364	-
31729349	31729886	-
31728233	31728483	-
44678251	44678543	-
41924652	41925403	-
43483850	43484148	-
43484158	43484530	43484410:43484421:-:GCCCCCTTGGGG
118755251	118755370	-
119596990	119597220	-
174159920	174160385	-
37084545	37085043	-
37085060	37085278	-
38751194	38751962	-
172555403	172555829	-
162497318	162497664	-
75717191	75717475	-
14954101	14954470	-
43226809	43227108	-
43227352	43227523	-
36242332	36242592	-
32106291	32106472	-
32165498	32165854	-
134648979	134649184	-
134648359	134648722	-
66925436	66925748	-
66924602	66924778	-
146028311	146028523	-
69369582	69369973	-
27022434	27022799	-
181033041	181034209	-
112860767	112861072	-
112861076	112861392	-
11743057	11743228	-
111884071	111884464	-
90410104	90410634	-
90409218	90409740	-
10886719	10887050	-
244864688	244865630	-
108719118	108719549	-
156341148	156341643	-
35497705	35498125	-
35497026	35497671	-
1524120	1524382	-
4615176	4615413	-
4615564	4615910	-
110713169	110713322	-
33440604	33441401	-
88691181	88691409	-
85010683	85011041	-
35141439	35141653	-
98929127	98929377	-
56834874	56835109	-
86913893	86914419	-
59220750	59221110	-

24426505	24426823	-
14664107	14664306	-
52365391	52365795	-
171460763	171461102	-
123154060	123154201	-
169694932	169695314	-
169694187	169694581	-
73972336	73972776	-
25608325	25608668	25608447:25608458:-:GCCACCAGGTGG
25734870	25735417	-
25734870	25735417	-
12483983	12484416	-
14651247	14652364	-
16264946	16265341	-
16264946	16265341	-
50287689	50288074	50287768:50287779:+:CCAGTAGGTGGC
9615411	9615853	-
50372688	50373368	-
11997607	11997795	-
102655402	102655595	-
103020127	103020358	-
103020992	103021355	-
91070287	91070492	-
110958876	110959181	-
6708094	6708429	-
97736935	97737058	-
15729536	15729767	-
133026124	133026339	-
112707455	112707932	-
112715961	112716762	-
112955998	112956303	-
22766292	22767112	-
135557470	135558899	-
178112243	178112777	-
178113077	178113285	-
149028299	149029495	-
149027485	149028024	-
22001049	22001287	22001157:22001168:+:CCGCCAGGGGGC
149729885	149730004	-
149960507	149960881	-
75027571	75027707	-
73949888	73950044	-
74150645	74151115	-
74151138	74151554	-
94895064	94895333	-
95268925	95270073	-
28885259	28885727	-
29019098	29019542	-
29024390	29025597	-
29073568	29073891	-
29072739	29073557	-
31732435	31733109	-
141879081	141879472	141879350:141879361:-:GCCATCTAGAGG
96260788	96261069	-

96260788	96261069	-
69179509	69180738	-
73523793	73524127	-
75099975	75100386	75100116:75100127:-:GCCCCCTGGTGG
37900284	37900431	-
37899312	37899524	-
38176072	38176476	-
123041833	123042147	-
123417332	123417689	-
125091728	125092021	-
30650353	30650753	30650630:30650641:+:CCGCCAGGTGGC
30657594	30657948	-
30747933	30748299	-
100132820	100133179	-
49155444	49155893	-
40160541	40161024	-
40161065	40161230	-
186783443	186784395	-
9843626	9843894	-
10248052	10248424	-
10248817	10249202	-
10292864	10293388	-
10320953	10321357	-
72955395	72955818	-
186375216	186375939	186375615:186375626:+:CCACTAGAGGGC
53196316	53196656	-
16440142	16440472	-
16440505	16440851	16440616:16440627:+:CCAGCAGAGGGC
155003785	155003968	-
155069947	155070306	-
70439262	70439611	-
37267538	37267901	-
37365633	37365987	-
38804884	38805466	-
39445499	39446115	-
110001882	110002284	-
41954160	41954977	-
81294469	81294696	-
139230928	139231596	-
140923928	140924497	-
120641480	120641596	-
89893722	89894449	-
120904558	120904856	-
2585743	2585992	-
2585506	2585719	-
2556566	2556707	-
2229033	2229261	-
235493510	235493652	-
25997987	25998542	25998307:25998318:-:TCCCCCTGGTGG
25996999	25997558	-
6683092	6683659	6683546:6683557:-:GCCCCCTAGTGT
136862429	136862643	-
143367640	143368275	-
28087944	28088765	-

28087549	28087916	-
27012091	27012338	-
6204866	6205382	-
26157658	26158245	-
26157224	26157646	-
26284532	26285699	-
99181047	99181332	-
99336448	99336678	-
53422951	53423140	-
129224252	129224899	-
129225372	129226575	-
49915646	49916036	-
88569682	88570043	-
75525485	75525618	-
204411512	204411834	-
130667679	130668189	-
76444238	76445124	-
28399394	28399741	-
159925597	159925801	-
205631984	205632350	-
88706372	88706626	-
161020759	161021140	-
161045762	161046635	-
161117477	161117884	-
89719384	89719745	-
20633278	20633538	-
24844798	24844916	-
22604399	22604778	-
28885615	28887248	-
28887788	28888206	-
32278057	32278521	-
78139531	78140103	-
136944650	136944869	-
136944887	136945128	-
136943873	136944060	-
32612967	32613146	-
32612281	32612829	-
32728095	32728312	-
32728331	32728805	-
32771955	32772337	-
33230363	33230673	33230659:33230670:-:GCCCCCTTGAGG
33541496	33543437	-
33588728	33589454	-
48908021	48908294	-
48945034	48945257	-
48996355	48996615	-
36320719	36321241	-
36990157	36990785	-
36989594	36989852	-
202958522	202959005	-
203304689	203305284	-
203305973	203306685	-
43106064	43106338	-
43390104	43390239	-

57186840	57186958	-
45686182	45688605	-
45686182	45688605	-
63476198	63476335	-
57976110	57976785	-
143379847	143380356	-
28772594	28772792	-
44002740	44003102	-
44304986	44305207	-
10472011	10472912	-
32205455	32205715	-
32222204	32222668	-
32221347	32221633	-
32394128	32394479	-
1574847	1575206	-
1273105	1273275	-
42403635	42404034	-
42496308	42496480	-
42496308	42496480	-
42879223	42879947	-
42878727	42879099	-
42879223	42879947	-
42878727	42879099	-
42973053	42974229	-
43659700	43659871	-
43659298	43659678	-
43659298	43659678	-
43659700	43659871	-
43718018	43718178	-
43719265	43719455	-
43719871	43720073	43719938:43719949:+:CCAGGAGGTGGC
154546507	154546655	-
43776056	43776313	-
44133332	44133525	-
44133543	44133948	-
44339276	44339727	-
44938974	44939368	-
133149457	133149618	133149666:133149677:-:GCCACCTGCTGG
46156225	46156417	-
46184684	46185172	-
46184203	46184500	-
46227899	46228419	-
133993140	133993381	-
46286133	46286426	-
46635369	46636036	-
136326637	136326776	-
127735316	127735553	127735428:127735439:+:CCAGAAGGTGGC
127734584	127734878	-
127904929	127905086	-
40577520	40577715	-
128772148	128772405	-
40466067	40466600	-
132079831	132080016	-
42219594	42220129	42219718:42219729:+:TCACAAGGGGGC

118251889	118252500	-
28725219	28725716	-
28727983	28728454	-
153627603	153627986	-
154974771	154975052	-
154973667	154974035	-
119067723	119068043	-
154559212	154559524	-
43690371	43690876	-
155308346	155309145	-
156193070	156193189	-
156193823	156194062	156194054:156194065:-:GCCCCCTGCAGG
156054251	156054625	-
99734331	99734441	-
144445049	144445287	-
144478764	144479131	-
102464812	102464959	-
144792369	144792592	-
102464812	102464959	-
103074704	103074866	103074855:103074866:+:CAACCAGGGGGC
3573925	3574073	-
21627828	21630314	-
21632088	21634212	-
196287300	196287540	-
196286972	196287189	-
36054375	36054842	36054615:36054626:+:CCTCTAGGTGGC
36053940	36054275	-
76385060	76385160	-
76737127	76737441	-
48363618	48364179	-
49452983	49453194	-
49453223	49453941	-
50476517	50476629	-
12919126	12919410	-
11155772	11156117	-
11155109	11155377	-
33789094	33789343	-
4731220	4731421	-
4731432	4731623	-
2459717	2459870	-
2428923	2429161	-
2428501	2428679	-
2472044	2472173	-
2968833	2969038	-
72434196	72434770	72434313:72434324:-:GCCCCCCGGTGG
72433769	72434063	-
61362586	61362940	-
62671872	62672098	-
62727209	62727424	-
62728845	62729028	-
65382891	65383120	-
65084525	65084827	-
64358058	64358259	-
64359164	64359304	-

64359317	64359531	-
47232451	47232873	-
47333382	47333764	-
53220963	53221218	-
53220189	53220845	-
53238425	53238789	-
54624427	54624550	-
6553911	6554081	-
28668177	28668686	-
9943356	9943995	-
15741755	15741923	-
30756529	30758279	-
30909479	30909756	-
31644212	31645820	-
32703482	32703733	-
32650493	32651106	-
32740959	32741195	-
32742434	32742889	-
32350671	32351212	-
20185154	20185634	-
20186375	20186722	-
2212526	2212752	-
61724996	61725549	-
62435536	62436083	-
77979292	77979694	-
77979292	77979694	-
109484134	109484462	-
99849320	99849665	-
99850234	99850478	-
100896572	100896934	-
100896572	100896934	-
247332354	247332658	-
247856921	247857121	-
161722034	161722255	-
209784540	209785238	-
212857782	212858052	-
110337782	110338595	-
110338671	110339455	-
179365954	179366128	-
181088255	181088976	-
220089671	220090202	-
222711622	222712159	-
222712659	222713365	-
246566155	246566628	-
246566155	246566628	-
235504669	235505165	-
223700722	223701263	-
228109110	228109373	-
228108673	228109014	-
61017155	61017770	-
61017155	61017770	-
199955756	199955915	-
199955756	199955915	-
151828547	151829072	-

55231941	55232810	-
183078213	183078644	-
183078650	183079222	-
17538383	17538546	-
226061207	226061716	-
169479254	169480137	169479646:169479657:+:CCTGTAGAGGGC
94452518	94452710	-
150362794	150363534	-
96858064	96858277	-
151165520	151166390	-
151175572	151175769	-
127293449	127293704	-
127527014	127527480	-
152047573	152048090	-
207710959	207711139	-
174486139	174486449	-
154963945	154964210	-
154993060	154993332	154993283:154993294:+:CCACCAGAGGGC
154993810	154994515	154994097:154994108:-:GACCTCTGGTGG
69013651	69013785	-
156591200	156591702	-
156282814	156283280	-
219245532	219245661	-
31531658	31532140	-
14125278	14125973	-
14123735	14124549	-
167734641	167735042	-
170870268	170870439	-
170869768	170870241	-
73069774	73070105	-
73069058	73069695	-
156673771	156674412	-
157160075	157160823	-
39458546	39459228	-
39639034	39639349	-
58306153	58306375	58306159:58306170:+:ACACCAGAGGGC
9916579	9916874	-
9933159	9933484	-
9932804	9933125	-
9934039	9934421	-
180601764	180602436	-
74039377	74039697	-
75514610	75514895	-
75514053	75514302	-
27381038	27381236	-
27663442	27664019	-
44976264	44976709	-
44975414	44975734	-
47513294	47514033	-
34859528	34860314	34859899:34859910:-:GCCCTCTGTTGG
35031579	35031837	-
37514759	37515339	-
184361100	184361296	-
185499240	185499521	-

185498559	185498937	-
128680642	128680816	128680870:128680881:+:CCGCCAGAGGGC
129440215	129440565	-
186806472	186806702	-
186806180	186806465	-
196432723	196432961	-
196639233	196639422	-
196639975	196640756	-
6692494	6692792	-
6693262	6693645	-
8159101	8159253	-
42767254	42767486	-
42846058	42846634	-
42846857	42847525	-
99950153	99950394	-
103019618	103019969	-
49673160	49673388	-
127782414	127782791	-
127965898	127966168	-
127965165	127965373	-
127965165	127965373	-
127965898	127966168	-
120066795	120067061	-
139301048	139301569	-
152779825	152780460	-
145098732	145099054	-
145098402	145098678	-
145098119	145098396	-
145098119	145098396	-
145098402	145098678	-
145098732	145099054	-
36150838	36151188	-
90529855	90530351	-
127517271	127517464	-
95554912	95555798	-
150000263	150000889	-
7389587	7389809	-
184649914	184650195	-
184649914	184650195	-
39073127	39073780	-
73565665	73565977	-
159262776	159263115	-
73565665	73565977	-
132807253	132807390	-
139374543	139374741	-
173055730	173056133	-
154189615	154190042	-
154445103	154445279	-
150448667	150449555	-
107564255	107564626	-
141636488	141636736	-
39228581	39228902	-
6707071	6707580	-
41895560	41895924	-

47260285	47260519	-
1043648	1043988	-
1082320	1082455	-
151058649	151059346	-
151057492	151058631	-
151056937	151057100	-
151056042	151056594	-
151057492	151058631	-
151081744	151081935	-
151079803	151080476	-
151083743	151083991	-
125091728	125092021	-
34329124	34329264	-
124372845	124373068	-
124373088	124373325	-
38386232	38386430	-
112486153	112486768	-
123269041	123269551	-
125261592	125261702	-
125262456	125262666	-
33448005	33448171	-
35072749	35072981	-
35103133	35103444	-
135520596	135520765	-
72222996	72223204	-
72222533	72222929	-
63520640	63521123	-
12195796	12196206	-
7788049	7788187	-
49114852	49115167	-
75209687	75210480	-
75210591	75210866	-
117375728	117375900	-
84138785	84139356	-
84139758	84140370	-
136713426	136713569	-
137589430	137589675	-
68955605	68956775	-
20461897	20462146	-
21511345	21511564	-
97318682	97318839	-
45921485	45921642	-
47407287	47407788	-
16437014	16437252	-
93662163	93662302	-
98134267	98134533	-
95789664	95789877	-
38251731	38251970	-
98644608	98644977	-
98644177	98644364	-
40569079	40569469	-
50367817	50368093	-
103523843	103523966	-
103529578	103529693	-

101353695	101353971	-
101587211	101587349	-
43312598	43312759	-
102151984	102152470	-
43880610	43880905	-
49155444	49155893	-
70816554	70816829	-
72473982	72474462	-
54969332	54969639	-
54969332	54969639	-
108008671	108009307	108008821:108008832:+:CCAGCAGAGGGC
102853957	102854175	-
31897647	31897974	-
55732024	55732303	-
72215599	72216209	-
72216637	72217172	-
6419383	6419627	-
73110273	73110652	-
35663027	35663276	-
6604055	6604234	-
6612392	6613114	-
73625987	73626768	-
34254049	34254181	-
81006749	81007806	-
81076993	81077457	-
9314800	9315181	-
51971530	51971970	-
9460185	9460799	-
9572976	9573277	-
9572704	9572902	-
19377988	19378167	-
100082185	100082334	-
100081557	100082069	100081939:100081950:-:GCCCTCTGCTGG
66551295	66552295	-
87037321	87037760	-
113220534	113220724	-
66934543	66934701	-
37527915	37528161	-
37526893	37527111	-
73193157	73193351	-
44711425	44711782	-
84747841	84748035	-
71809268	71809560	71809281:71809292:-:GCCACCTGGAGG
71808826	71809150	-
71808499	71808818	-
64163270	64163967	-
64093530	64094066	-
64093085	64093517	-
64387549	64387949	-
89750744	89750860	-
65185444	65186054	-
57941816	57942178	-
59754040	59754269	59754129:59754140:+:CCACCAGGGGGC
59810649	59810956	-

100479027	100479157	-
67064072	67064560	-
43510999	43511576	-
90954830	90955251	-
30667976	30668121	-
127715665	127716029	-
127931037	127931350	-
127931577	127931814	-
128276007	128276413	-
128276506	128277267	-
128275247	128275462	-
128321913	128323756	-
128321913	128323756	-
128341225	128341764	-
129080445	129080604	-
44899028	44900053	-
128818108	128818424	128818379:128818390:+:CCAGCAGGGGGC
47895052	47895499	-
19067276	19067701	-
75903686	75904026	-
75904475	75904680	75904580:75904591:-:GCCACCTGGAGG
80036481	80036613	-
3642599	3642863	-
3854149	3854781	-
4094832	4095076	-
82735925	82736052	-
71462165	71462359	-
43596199	43596834	-
44164711	44165099	-
46633670	46633995	-
58219320	58219643	-
1103627	1103790	-
1248047	1248178	-
1249054	1249192	-
88662857	88663090	-
89490742	89490899	-
89657772	89657882	-
89658337	89658577	89658370:89658381:+:CCACCCGGGGGC
28712394	28712846	-
89560345	89560797	-
48814608	48814860	48814820:48814831:-:GCCACCTGGAGG
28902170	28902379	-
40777539	40778065	-
40778708	40779249	-
35757806	35758123	-
54448909	54449309	-
54449603	54449916	-
42313963	42314112	-
42219594	42220129	42219718:42219729:+:TCACAAGGGGGC
140458423	140458624	-
140457678	140457876	-
36214115	36214542	-
36214613	36215094	-
36215237	36215555	-

38316976	38317557	-
4403205	4403742	-
144508073	144508410	-
1716472	1716614	-
1717043	1717633	-
5680612	5680976	-
50804479	50804955	-
8321681	8321945	-
8308384	8308708	-
8307737	8308121	-
53078570	53078874	-
67606565	67607001	-
67508466	67508861	-
74465938	74466084	-
76072564	76072866	-
76072883	76073117	-
78130272	78130695	-
55772456	55772706	-
235142	235661	-
2009983	2010313	-
2223611	2223740	-
2252040	2252375	-
2340786	2341153	2341005:2341016:+:CCAGCAGGGGGC
2751605	2751916	-
61342082	61342971	-
61919911	61920227	-
61919163	61919437	-
61949635	61950690	-
62855620	62856649	-
72813780	72814003	-
72814480	72814730	-
74171152	74171473	-
39107530	39107885	-
39406432	39407027	-
70203423	70203661	-
70203958	70204182	-
27837815	27838286	-
100088508	100088818	-
117143907	117144204	-
9397902	9398370	-
228424657	228424892	-
82900648	82900816	-
72273516	72273836	-
72272555	72273352	-
72273874	72275794	-
235651302	235651418	235651412:235651423:-:GCCCCCGACTGG
234610340	234610694	-
234373198	234374011	-
67841835	67842294	-
149587619	149587932	-
26155984	26156479	-
51899219	51899530	-
184474977	184475250	-
57597648	57598072	-

74700439	74700551	-
23453211	23453421	-
9885205	9885724	-
9885839	9886528	-
22141600	22141822	-
22141099	22141348	-
28822306	28822631	-
22244414	22245137	-
61792437	61792989	-
191846468	191846667	-
31410132	31410371	-
62791560	62791755	-
44186939	44187200	-
39025684	39025935	-
39026135	39027032	-
67226960	67227320	-
109984306	109984821	109984585:109984596:-:GCCACCTGGTGG
109655579	109656273	-
29567757	29567949	-
37466066	37466393	-
43330480	43330921	-
43330480	43330921	-
69186458	69186610	-
70346633	70347017	-
68277599	68277805	-
674278	674584	-
122793844	122794014	-
64356074	64356331	-
49927775	49927943	-
49929012	49929146	-
49929280	49930061	-
48008475	48008589	-
55038473	55038652	-
28212112	28212517	-
30355427	30355943	-
30370880	30371044	-
155208276	155208500	-
155135525	155135767	-
155126970	155127355	-
176609264	176609509	-
77862606	77863035	-
37826128	37826277	-
155854276	155855034	-
155853977	155854161	-
93676939	93677477	-
75455948	75456171	-
96494115	96494249	-
70088145	70088379	-
118988598	118989018	-
118988074	118988345	118988221:118988232:+:CCGGCAGGGGGC
136996924	136997064	-
29995241	29995481	-
29995655	29995867	-
29995894	29996234	-

3857250	3857494	-
69437691	69437861	-
69437420	69437679	-
83011554	83012363	-
83010838	83011181	83011014:83011025:+:CCAGCAGGAGGC
82985710	82986152	-
82023645	82023780	-
82023645	82023780	-
81976495	81977034	-
129183801	129183940	-
82065737	82065970	-
43649106	43649455	-
108533720	108533947	-
63840419	63840720	-
43409394	43409545	-
43408801	43409177	-
15601397	15601926	-
101084997	101085263	-
19881998	19882359	19882259:19882270:+:CCACTAGAGGGC
65982184	65982422	-
30559115	30559441	-
30558163	30558300	30558318:30558329:+:GCACTAGAGGGC
30535659	30536050	-
30534660	30534846	-
30526890	30527100	-
144095231	144095918	-
152779825	152780460	-
7932538	7932999	-
7932538	7932999	-
177212193	177212745	-
75086551	75086973	-
25624651	25624824	-
33097243	33097633	-
33114141	33114443	-
111911814	111912142	-
111911363	111911795	-
16380815	16381213	-
4303054	4303503	-
4303525	4304088	-
52949669	52949882	-
140691255	140691711	-
139385416	139385620	139385537:139385548:+:CCACAAGGTGGC
87422339	87422789	-
56104391	56104609	-
163459914	163460408	-
163460722	163461130	-
45886694	45887392	-
133051227	133052024	-
45863068	45863549	-
45864377	45864587	-
54473584	54473893	-
103074704	103074866	103074855:103074866:+:CAACCAGGGGGC
53969824	53970009	-
45076558	45076766	-

43575409	43575689	-
43576153	43576604	-
120446211	120446485	-
14123735	14124549	-
14125278	14125973	-
54200807	54201186	-
54102542	54102844	-
54103481	54103612	-
54102542	54102844	-
54103481	54103612	-
52361713	52362054	-
31369622	31369947	-
31369622	31369947	-
142402317	142402674	-
68272437	68272628	-
179347411	179347836	-
149872352	149873335	-
149873351	149873936	-
9890632	9890889	-
14027988	14028175	-
120629617	120629828	-
128159758	128160140	128160045:128160056:-:GCCCCGTGGTGG
128159326	128159697	-
128160287	128160535	-
128160643	128161029	-
248838552	248839180	-
248859182	248859739	-
248858379	248858908	-
248858053	248858366	-
29940259	29940512	-
12791407	12791717	-
4539719	4540322	-
38453953	38454318	-
79008853	79009133	-
97445965	97446112	-
97445965	97446112	-
27772202	27773119	-
32357487	32358001	-
89632659	89632877	-
89633099	89633458	-
89820402	89820653	-
158672034	158672270	-
31723202	31723348	-
40777539	40778065	-
40778708	40779249	-
139646691	139646991	-
58182295	58182607	-
6519157	6519363	-
64050043	64050237	64049973:64049984:-:GCCCCCCGCTGG
64050346	64050753	-
141636488	141636736	-
37906921	37907128	-
169794534	169794811	-
36125920	36126040	-

10121925	10122092	-
3378605	3378843	-
62386050	62386243	-
781318	781432	-
782285	782465	-
3558434	3558756	-
5132071	5132395	-
42657905	42658626	-
63520640	63521123	-
2096030	2096205	-
71358615	71358742	71358652:71358663:-:GCCCCCTTCTGG
30906148	30906391	-
20212022	20212262	-
43064595	43065201	-
119108312	119108515	-
119121088	119121324	-
134646800	134647693	-
100706909	100707068	-
81324581	81324814	-
636332	636764	-
636144	636323	-
119101855	119102542	-
43629304	43630479	-
95577754	95577958	-
153971560	153971761	-
73782400	73782671	-
122789873	122790091	-
67317504	67318003	-
70382069	70382346	-
65856989	65857280	-
37257738	37258157	-
57186840	57186958	-
74671298	74671535	-
112975757	112976689	-
74203442	74203952	74203553:74203564:+:CCACAAGGGGGC
66934543	66934701	-
119038688	119038821	-
171999740	172000460	-
40524254	40524846	-
40525281	40525523	-
71453359	71453499	-
71453359	71453499	-
65720645	65720980	-
69294332	69294567	-
67288203	67288545	-
38137374	38137596	-
38138971	38139268	-
38164422	38164677	-
38164680	38165621	-
65711064	65711202	-
65711900	65712296	-
45060625	45061303	-
67265722	67265976	-
28855041	28855302	-

28854582	28854970	-
155940737	155941085	-
64318164	64318478	-
64317549	64318108	-
144373417	144373614	-
64304820	64305002	-
61888564	61888827	-
155208276	155208500	-
154973667	154974035	-
154974771	154975052	-
61176402	61177088	-
149141151	149141383	-
111259079	111259408	-
115641621	115641828	-
64269901	64270697	-
64270755	64270886	-
135345103	135345215	-
42009843	42010197	-
65614983	65615829	-
125430173	125430283	-
127700974	127701131	-
127700443	127700908	127700635:127700646:+:CCTCTAGGAGGC
127701879	127701986	-
230978802	230979378	-
19596361	19597099	-
119335059	119335305	-
64247027	64247434	-
64246154	64246330	-
65570112	65570600	-
65569849	65570079	-
47781885	47782314	-
47781385	47781761	-
16465447	16466036	-
26345901	26346277	-
94460348	94460677	-
94459403	94459666	-
66958751	66959010	-
66958282	66958436	-
9943356	9943995	-
66855789	66856208	-
66843528	66843639	-
235128770	235129402	-
235128377	235128626	-
41966125	41966313	-
80414957	80415242	-
44799575	44800016	-
35038018	35038197	-
66677974	66678318	-
66678357	66678666	-
127030290	127030462	-
127030020	127030259	-
11374144	11374904	-
66637989	66638818	-
66638859	66639218	-

196639233	196639422	-
196639975	196640756	-
196711870	196712023	-
196568372	196568616	-
1420543	1420944	-
100535110	100535360	-
109493517	109494065	-
101561654	101561977	-
88706372	88706626	-
64222378	64222563	-
64221806	64222039	-
499984	500130	-
1562991	1563111	-
7484326	7484559	-
41316967	41317177	-
7035651	7035896	-
1200606	1200922	-
241847268	241847703	-
241847719	241848422	-
66510412	66510766	-
65792403	65792909	-
50837009	50837163	-
66421572	66422053	-
136196465	136196818	-
58361975	58362417	-
213015655	213016005	-
213016273	213016499	-
9435535	9435775	-
91908970	91909231	-
23916827	23917466	-
112145887	112146147	-
75514053	75514302	-
75514610	75514895	-
55948640	55948901	55948771:55948782:+:CCTCCAGGGGGC
57555808	57556332	-
66289608	66289854	-
5903610	5904279	-
5720357	5720573	-
5680612	5680976	-
29926276	29926667	-
142578505	142578832	-
26110257	26111192	-
26111689	26112078	-
248826143	248826925	-
133274065	133274395	-
183884816	183885143	-
72686521	72686897	-
46700285	46700471	-
46700652	46701121	-
46700652	46701121	-
46700285	46700471	-
46617744	46618003	46617930:46617941:-:GCCCCCGGGTGG
10429860	10430637	10430368:10430379:+:CCGCCAGGGGGC
10430965	10431289	-

38176072	38176476	-
66001730	66002484	-
8965031	8965259	-
66002913	66003254	-
66001730	66002484	-
96434237	96434351	-
65961537	65961772	-
65961212	65961534	-
18397744	18397962	-
18397457	18397596	-
67374273	67374732	67374313:67374324:-:GCCCCCTGCGGG
74010026	74010319	-
73983521	73984040	-
73983057	73983315	-
73787604	73787993	-
73972336	73972776	-
2944873	2945186	-
83052130	83052488	-
1376310	1376656	-
1375051	1375448	-
94259141	94259310	-
94259332	94259598	94259540:94259551:-:GTCCTCTGGTGG
128275247	128275462	-
128276007	128276413	-
95133240	95133640	-
120709955	120710110	-
90529855	90530351	-
1231241	1231812	-
51926850	51927493	-
90474142	90474649	-
140564430	140564643	-
140564651	140564937	-
49786642	49786873	-
49787001	49787354	-
49785789	49785968	-
33161863	33162084	-
812662	813670	-
80991418	80991544	-
100890343	100890527	-
33039029	33039353	-
82213402	82213590	-
45902993	45903236	-
70205919	70206174	-
32650493	32651106	-
63974611	63974797	-
63974886	63975045	-
122203219	122203370	-
113275313	113275663	-
30824857	30825042	-
56468735	56469048	-
28553840	28553958	-
2457545	2457680	-
657903	658188	-
658194	658728	-

13653433	13653590	-
136944887	136945128	-
136944650	136944869	-
73743509	73744512	-
84653853	84654224	-
3841660	3841888	3841733:3841744:-:GCCCTCTGGTGC
580731	581035	-
13726587	13726748	-
53054671	53054914	53054961:53054972:+:CCACTAGGTGGC
776702	776860	776905:776916:+:CCAGGAGGGGGC
35642371	35642530	-
35641517	35641652	-
75515809	75516092	-
75516112	75516446	-
49166634	49166890	-
49297913	49298635	-
49298674	49299976	-
49118419	49118617	-
18260525	18260658	-
28936062	28936411	-
809274	809953	-
808688	809138	-
809274	809953	-
808688	809138	-
51699215	51700029	-
230426616	230426779	-
128879438	128879761	-
128880120	128880263	-
108569640	108570036	-
33491451	33492161	-
324796	324938	-
137754456	137754765	-
44678251	44678543	-
96129867	96130195	-
153971560	153971761	-
40476505	40476859	-
115841481	115841878	-
89972121	89972521	-
207681	207960	-
153990487	153991063	-
207681	207960	-
44213142	44213757	-
49029241	49029483	-
49028604	49028902	-
4343391	4343495	-
49023135	49023298	-
18022240	18022747	-
28846291	28846410	-
108497780	108498077	-
108497280	108497770	-
57320187	57320462	-
7925350	7925479	7925327:7925338:-:GCCTCCTTGTGG
36993351	36993800	-
36991983	36993232	-

9910552	9911038	-
94063120	94063560	-
94063120	94063560	-
74938253	74938404	-
74937303	74937511	-
74889514	74890033	-
1919551	1920101	-
1918233	1918486	-
47324062	47324376	-
144078915	144079156	-
8247577	8247718	-
932722	932977	-
28846291	28846410	-
8247577	8247718	-
47779317	47779504	-
38619051	38619503	-
6709580	6710000	-
54517795	54517976	-
41230491	41231263	-
6640435	6640718	-
144095655	144095884	-
12933736	12934520	-
18634653	18635274	-
74696514	74696742	-
44337903	44338247	-
44338658	44338968	-
12937923	12938227	-
12939161	12939549	-
12965003	12965592	-
74598115	74598599	-
74598616	74599075	-
74540425	74540713	-
140524431	140524754	-
23565434	23565837	-
74504078	74504200	-
88452412	88452633	-
17206588	17206751	-
17205960	17206100	-
73312548	73312707	-
144104809	144105020	-
64751787	64751938	-
7915285	7915525	-
30445752	30446327	-
55624898	55625003	-
23094366	23094709	-
23095276	23095506	-
76348414	76348673	-
76348749	76348984	-
143829622	143830021	-
55487820	55487963	-
30430413	30430918	-
30406811	30407393	-
30378436	30378567	-
28552688	28553032	-

28553415	28553907	-
1827018	1827353	-
30370880	30371044	-
30371643	30371767	-
30284184	30284317	-
64703391	64703710	-
44689674	44689960	-
44690267	44690501	-
64460084	64460417	-
64461477	64461718	-
46617744	46618003	46617930:46617941:-:GCCCCCGGGTGG
114400253	114400560	-
13923871	13924838	-
207053281	207053818	-
70233698	70234022	-
153794053	153794307	-
34963535	34963711	-
143815751	143815916	-
71289257	71289525	-
143732915	143733037	-
42548661	42549092	-
122733436	122733804	-
12068623	12068784	-
228457621	228458226	-
149496850	149497135	-
7484326	7484559	-
227563174	227563702	-
47164709	47165173	-
57849767	57850376	-
2929248	2929453	-
68498931	68499213	-
27392042	27392424	-
36398036	36398326	-
36398595	36398907	-
36397528	36397994	-
36397263	36397518	-
56222297	56222510	-
228165154	228165468	-
228165768	228165995	-
228166511	228167028	-
134845018	134845962	-
46471006	46471129	-
74924190	74924316	-
34342896	34343456	34343237:34343248:+:CCAGAAGGGGGC
95876002	95876260	-
138001357	138002508	-
75515809	75516092	-
75516112	75516446	-
73251831	73252333	-
122979927	122980045	-
122980142	122980309	-
58326795	58327511	-
106077649	106077882	-
106076562	106076986	-

46286133	46286426	-
1755941	1756218	-
39296689	39296810	-
34101774	34102215	-
53251205	53251665	-
59154787	59155133	-
37072264	37072591	-
37073373	37074153	-
25871312	25871678	-
207032625	207032973	-
68023098	68023801	-
57668063	57668273	-
57668915	57669037	-
197949826	197950741	-
499984	500130	-
93740857	93741133	-
74924190	74924316	-
81711841	81711973	-
81712679	81713053	81712684:81712695:-:GCCCTCCAGTGG
149551466	149551937	-
109095164	109095799	-
12695926	12696310	-
202888944	202889539	-
42079672	42079908	-
48993539	48993803	48993477:48993488:+:CCTCCAGGGGGC
48994166	48994348	-
90233505	90233946	-
90233253	90233406	-
44874189	44874496	-
177517356	177517594	-
51238747	51239072	-
86913893	86914419	-
38004526	38005051	38004595:38004606:+:CCCCCAGGGGGC
153469970	153470098	-
131949526	131949647	-
131950551	131950691	-
117605149	117605328	-
98608398	98608531	-
38008877	38009616	-
20016114	20018819	-
3761605	3762096	3761750:3761761:+:CCACGCGGGGGC
3762655	3763323	-
31032139	31032370	-
124450549	124451023	-
181260825	181261070	-
1971696	1972159	-
28742217	28742431	-
144170481	144170758	-
93740067	93740736	-
2009983	2010313	-
31944817	31945151	-
31943918	31944787	-
38121981	38122392	-
38121134	38121373	-

122226492	122226756	-
91022548	91022731	-
19756201	19756907	-
30906148	30906391	-
19527240	19528320	-
19526213	19526962	-
19202935	19203175	-
53365602	53365743	-
2215326	2215549	-
41688147	41688285	-
123458277	123458624	-
154020565	154020723	-
149886572	149886991	-
47571363	47571595	-
2400181	2400485	-
2400577	2400829	-
122527032	122527755	-
169701886	169702175	169702177:169702188:-:GCCCCCTGCGGG
19940624	19943389	-
141391738	141391873	-
141391365	141391585	-
34342896	34343456	34343237:34343248:+:CCAGAAGGGGGC
90931722	90932540	90932193:90932204:-:GGCCCCTTGTGG
75148330	75148597	-
78360884	78361030	78360946:78360957:+:CCGCCAGAGGGC
6636660	6636901	-
6635528	6635679	-
64404072	64404188	-
64405054	64405241	-
139482574	139482733	-
77279920	77280093	-
25458908	25459082	-
22451742	22452182	-
149886572	149886991	-
3020029	3020188	-
3020494	3020752	-
19713646	19714100	-
19714828	19715495	-
31496263	31496460	-
43800007	43800606	-
43800852	43801129	-
63671397	63671979	-
169751506	169751836	-
8797739	8797979	-
8798041	8798646	-
155644063	155644576	-
151261200	151261521	-
151260733	151261152	-
44818974	44819330	-
111562537	111562776	-
24793047	24793571	-
24792577	24793039	24792917:24792928:+:CCAGCAGGGGGC
42090416	42090568	-
144326609	144326963	144326740:144326751:-:GCCCCCTGCCGG

74151138	74151554	-
74150645	74151115	-
3159777	3160020	-
3159443	3159753	-
90233505	90233946	-
90233253	90233406	-
173973285	173973619	173973345:173973356:-:GCCACCTGGTGG
50960320	50960749	-
169701886	169702175	169702177:169702188:-:GCCCCCTGCGGG
140107361	140107833	-
103123958	103124072	-
248906279	248906499	248906223:248906234:-:GCCCTCTGGTGC
20393090	20394844	-
173867715	173868134	-
81665351	81666306	-
81666637	81666828	-
54310692	54310968	-
54309457	54309892	-
81330309	81330419	-
162727747	162728006	-
50270826	50271052	-
99181047	99181332	-
39445499	39446115	-
92810029	92810496	-
92809560	92809917	-
92809113	92809494	92809262:92809273:+:CCGCCAGGGGGC
62787400	62787946	-
155193176	155193408	-
155193752	155194030	-
81553640	81553806	-
53379369	53379583	-
19431645	19432963	-
49802694	49802891	49802732:49802743:+:CCACTAGAGGGC
705309	705423	-
197960431	197961037	-
88686644	88686865	-
88686925	88687142	-
62753969	62754379	-
132084973	132085152	-
22558836	22560602	-
31400093	31400215	-
143289072	143289255	-
143289765	143289957	-
196568372	196568616	-
37073373	37074153	-
37072264	37072591	-
19852274	19856537	-
25016398	25016964	-
313946	314209	-
49170756	49170956	-
36079237	36079828	-
19516618	19516805	-
36489755	36490030	-
36489193	36489449	-

44071910	44072263	-
222712659	222713365	-
36579763	36580238	36579836:36579847:-:GCCACCTCCTGG
145824253	145824630	-
78099142	78099333	-
118997633	118997824	-
118924555	118924850	-
44947842	44948305	-
74998486	74998756	74998632:74998643:+:CCACCAGAGGGT
6715731	6716241	-
57435181	57435444	-
96924851	96925081	-
57411050	57411259	-
94973411	94973617	-
179081570	179082068	-
145835328	145835882	-
145835934	145836334	-
145845373	145846111	145845878:145845889:-:GCCCTCTGGTGG
145846308	145846600	-
27321921	27322249	-
27322336	27322889	-
47436544	47436871	-
44503578	44503955	-
29603098	29603253	-
34249880	34250142	-
129953004	129953683	-
149648280	149649179	-
72793348	72793917	-
58579549	58580081	58579726:58579737:-:GCCCCCTGCCGG
23178407	23180473	-
50300087	50300469	-
50299154	50299326	-
113220534	113220724	-
45148325	45149091	-
45287947	45288105	-
72239199	72239344	-
49336934	49337069	-
57459631	57459825	-
76072564	76072866	-
8343558	8343717	-
6425165	6425363	-
28241387	28241783	-
41230491	41231263	-
1611823	1612100	-
223142667	223143070	-
13861632	13861851	-
13862338	13862555	13862425:13862436:-:GCCCCCTGCTGG
1000669	1001256	-
5903610	5904279	-
5905027	5905229	-
54023323	54023656	-
219160156	219160272	-
49568035	49568425	-
136373049	136373209	-

40449845	40450142	-
40450493	40450824	-
14058707	14058905	-
21045329	21046051	-
78147164	78147289	-
46811920	46812047	-
89760832	89761057	89760923:89760934:+:CCAACAGAGGGC
33097243	33097633	-
112275209	112275417	-
100148788	100149111	100149046:100149057:-:TCCCCCTGGTGG
1000669	1001256	-
2282178	2282312	2282103:2282114:-:GCCCTCTGGGGG
42301711	42301978	-
76397625	76397856	-
8064483	8064753	-
119095298	119095603	-
23980285	23981083	-
240560236	240560385	-
75183181	75183477	-
25429705	25430049	-
49773089	49773224	-
37859848	37860343	-
37859413	37859674	-
12333785	12333932	-
139484043	139484232	-
101051622	101051762	-
191330108	191330404	-
137255470	137255677	-
78188246	78188471	-
143450588	143450920	-
10939946	10940500	-
109093265	109093958	109093555:109093566:-:GCCCCCTGGTGC
70524009	70524417	-
73799626	73799800	-
32315400	32315639	-
116279598	116279937	-
100286734	100286854	-
67184009	67184114	-
67183364	67183771	-
144901959	144902115	-
159286879	159287154	-
159287272	159287630	-
127423879	127424455	-
225881393	225881648	-
35905855	35906028	-
38434117	38434252	-
9363234	9363554	-
9362662	9363044	-
30719589	30720073	-
40856857	40857042	40856895:40856906:+:CCTCCAGGGGGC
30955664	30956013	-
200889464	200889820	-
92293759	92294095	-
207320960	207321422	207321182:207321193:+:CCAGTAGAGGGC

5720357	5720573	-
5666623	5667224	-
144787027	144787259	-
133080343	133080813	-
90351960	90352387	-
41620566	41620738	-
150368400	150368833	-
150367857	150368329	-
150369217	150369868	-
290593	290945	-
110403719	110404703	-
144836062	144836371	-
44031897	44032356	-
29180350	29180517	-
90902152	90902547	-
152675753	152676443	-
152675336	152675748	-
97117396	97117699	-
99504546	99504897	99504631:99504642:-:GCCACCTCCTGG
119018230	119018676	-
119019147	119019343	-
177552753	177553152	-
42638750	42639048	-
42637956	42638218	-
153566958	153567148	-
27132715	27133148	-
34697032	34697568	-
110582587	110582722	-
228457621	228458226	-
177498361	177498606	-
49765917	49766447	-
73772070	73772421	-
154479216	154479351	-
49101468	49101615	-
40426068	40426456	-
40424957	40425220	-
149125982	149126761	149126377:149126388:-:GCCCCCTGCGGG
11766674	11766939	-
26103857	26104543	-
81928439	81928570	-
100168243	100168588	-
101963730	101963960	-
57477565	57477784	-
65614983	65615829	-
89524489	89524791	-
89524050	89524418	-
151028014	151029077	-
27893053	27893559	-
127651298	127651842	-
127652420	127653148	-
30585611	30586138	-
67365915	67366337	-
23457673	23457996	-
11198039	11198177	-

94475187	94475465	-
28080677	28081450	-
33418521	33419034	-
44311041	44311593	-
90716936	90717242	-
15616899	15617304	-
15617403	15617834	-
15617897	15618289	-
68903658	68903978	-
149090563	149090955	-
149090041	149090559	-
144799198	144799370	-
45750646	45751250	-
45750094	45750615	-
136792674	136792888	-
136791594	136791896	-
63658156	63658404	-
63658447	63658787	-
63657520	63657708	-
29663379	29663815	-
55476037	55476479	-
55476905	55477111	-
110981845	110982182	-
132410184	132410399	-
132410947	132411171	-
132411297	132411550	-
11271896	11272344	-
177618707	177619169	-
588643	588829	-
70354766	70355124	-
1444670	1444802	-
151070194	151070697	-
57280403	57280552	-
56041711	56042322	-
71851127	71851392	-
35805395	35805634	-
119892508	119892679	-
119892701	119892838	-
1772190	1772420	-
1773420	1773604	-
109075811	109076394	-
1511838	1512075	-
36666872	36666999	-
80335935	80336196	-
34733745	34734071	-
111755606	111756030	111755622:111755633:+:CCAGAAGAGGGC
23946179	23946396	-
14959095	14959232	-
99089052	99089250	-
27146802	27147301	-
27147470	27147835	-
89490742	89490899	-
29005325	29005818	-
81738054	81738403	81738176:81738187:-:GCCTCCTGGTGG

127450887	127451280	-
156825882	156826447	-
142961639	142962157	-
43758387	43758887	-
11434995	11435364	-
93467274	93467527	-
93467544	93467949	-
99658800	99658984	-
45971972	45972536	-
33485033	33485265	-
38121134	38121373	-
38121981	38122392	-
92290327	92290480	-
5296522	5297120	-
68129919	68130244	-
68129692	68129874	-
37857613	37857920	-
137277282	137277570	-
34988184	34988577	-
66924602	66924778	-
66925436	66925748	-
3204263	3204546	-
113584277	113584408	-
43390104	43390239	-
89918746	89918918	89918753:89918764:-:GCCGCCTGGTGG
89918236	89918370	-
89919103	89919317	-
63773113	63773606	-
28719805	28720325	-
71319584	71319789	-
9827718	9827942	-
67246782	67247041	-
112012616	112013229	-
63956581	63956737	-
43606196	43606561	-
28141377	28142243	-
75814651	75814970	-
143901262	143901877	143901754:143901765:+:CCAGCAGAGGGC
35691375	35691478	-
48016937	48017096	-
99472734	99473018	-
31541180	31542709	-
151016433	151016764	-
151015456	151015725	-
95554912	95555798	-
29590012	29590138	-
4136408	4136689	-
10750980	10751180	-
10751339	10751669	-
34072941	34073292	-
26900118	26901072	-
153764377	153764502	-
35467642	35468135	-
35468176	35468823	-

184037713	184038034	-
109763845	109764066	-
109762902	109763267	-
167629306	167629981	-
34961410	34961597	-
42335115	42335482	-
26471601	26472104	-
193592601	193593355	-
193593693	193593908	-
153946772	153946899	-
153945570	153945882	-
112206464	112206680	-
52033383	52034082	-
150602903	150603408	-
184051935	184052198	-
28992462	28993415	-
42980418	42980579	-
42980957	42981060	-
90995039	90995356	-
153909109	153909243	-
3900373	3901049	-
3899763	3899972	-
128829780	128830026	-
37500001	37500365	-
42071168	42071348	-
156282814	156283280	-
156281611	156282077	156281842:156281853:+:CCAACAGAGGGC
68988601	68988945	-
68988980	68989260	-
68989296	68989582	-
38165612	38165858	-
109410240	109410755	-
50625443	50625600	-
244834522	244835131	-
244835844	244836206	-
241640539	241640872	-
212791451	212792277	-
126340088	126340553	-
64050043	64050237	64049973:64049984:-:GCCCCCCGCTGG
64050346	64050753	-
63523437	63523751	-
52843097	52843332	-
45362662	45363067	45362965:45362976:+:CCTCTAGAGGGC
37946509	37947391	-
28369000	28369977	-
25429705	25430049	-
33288964	33289930	-
33200474	33201155	-
33201225	33201907	-
33199704	33200136	-
81666637	81666828	-
81665351	81666306	-
32967904	32968709	-
32969396	32969663	-

32969396	32969663	-
32969666	32970290	32970136:32970147:-:GCCCCCTGATGG
32967904	32968709	-
32177512	32178391	-
32177512	32178391	-
32154209	32154908	-
32154919	32155426	-
75633904	75634217	-
31971444	31972545	-
31972570	31972841	-
31972870	31973353	-
31971444	31972545	-
31958475	31959733	-
31958475	31959733	-
31902491	31902726	-
112086325	112086647	-
112086676	112086907	-
31897647	31897974	-
31863510	31864028	-
31862116	31862774	-
31834248	31835560	-
31826502	31827527	-
31806607	31807211	-
31805914	31806571	-
31795698	31796207	-
31776535	31778175	-
148020282	148020672	-
31739188	31739832	-
31739839	31740550	-
31717267	31718228	-
31706528	31707261	-
31701710	31703311	-
31683725	31683907	-
31663950	31666137	-
31659459	31660727	-
31652366	31653701	-
31652366	31653701	-
31592481	31592613	-
31585645	31585873	-
31585437	31585587	-
31546521	31546955	-
31399667	31399884	-
31180719	31180837	-
31158471	31158726	-
31159046	31159362	-
30646630	30647049	-
30616550	30618209	-
30326064	30326526	-
49877280	49877667	-
65169177	65169553	-
65169993	65170405	-
74472574	74472885	-
143573217	143573373	-
110468260	110468751	-

6580725	6580950	-
62669947	62670338	-
62669404	62669862	-
149125982	149126761	149126377:149126388:-:GCCCCCTGCGGG
103965199	103965924	-
89817569	89817828	-
35995130	35995269	-
158672034	158672270	-
67936941	67937328	-
67192563	67192917	-
65842079	65842508	-
12974812	12974979	-
39349467	39349815	-
46251143	46251278	-
34374626	34375662	-
34375670	34375948	-
33641915	33642226	-
33641563	33641845	-
4662900	4663048	-
2268253	2268467	-
1679060	1679320	-
19130896	19131677	-
123585172	123585529	-
46811920	46812047	-
53890027	53890736	-
52734738	52734867	-
49930544	49930952	-
49929280	49930061	-
49929012	49929146	-
197775563	197776063	-
168225582	168225926	168225933:168225944:-:GCCCCCCGGTGG
168225974	168226313	-
154732689	154733115	-
169694187	169694581	-
169694932	169695314	-
151057720	151058224	-
136363653	136363921	136363841:136363852:-:GCCCCCTGGCGG
58352745	58353078	-
96858064	96858277	-
10702055	10702238	-
177312719	177313021	-
69339521	69339661	-
69339930	69340098	-
65638477	65638645	-
65638808	65639039	-
65013902	65014056	-
65013279	65013461	-
65014246	65014476	-
140557455	140557883	-
33454646	33454888	-
72375894	72376249	-
65421277	65421515	-
1905231	1905389	-
32195986	32196316	32195969:32195980:-:GGCCTCTGGTGG

32128295	32128530	-
32127929	32128246	-
32127326	32127521	-
48465633	48465795	-
31739839	31740550	-
31739188	31739832	-
31729263	31730298	-
30907807	30908406	-
12813209	12813440	12813268:12813279:+:CCCGCAGGGGGC
34664698	34664822	-
32692362	32692524	-
32692833	32692990	-
110615209	110615483	-
19170770	19170946	-
150367857	150368329	-
150368400	150368833	-
133130542	133130898	-
16660521	16660828	-
16660135	16660311	-
35664830	35665271	-
5260658	5260919	-
184250224	184250518	-
36307065	36307319	-
10836363	10836982	-
44899028	44900053	-
74171152	74171473	-
72793348	72793917	-
82648335	82648455	-
41786827	41787452	-
62727209	62727424	-
62664678	62664878	-
62665357	62665929	-
16421520	16421727	-
16420471	16420695	-
19852274	19856537	-
7329796	7329943	-
18077498	18077943	-
3159777	3160020	-
3159443	3159753	-
3158975	3159277	-
41333801	41334015	-
41334061	41334325	-
2840370	2840507	-
674278	674584	-
49492382	49492534	-
157323224	157324027	-
109089304	109089870	-
109090303	109090966	-
11761965	11762158	-
2801634	2801855	-
131796855	131797298	-
174335173	174335330	-
66551295	66552295	-
37196118	37196671	-

37196861	37197056	-
16613259	16614020	-
7639940	7640203	-
7640563	7641151	-
24075963	24076472	-
42890338	42890483	-
42889959	42890175	-
49118419	49118617	-
61891082	61891250	-
1399432	1399681	-
1398363	1398652	-
109307071	109308100	-
44807025	44809643	-
33271002	33272137	-
18897498	18897635	-
11093630	11093814	-
81833702	81833950	-
81832759	81832911	-
48634518	48634722	-
49539068	49539860	-
44439311	44440149	-
120222783	120223112	-
120222477	120222619	-
43596199	43596834	-
33288964	33289930	-
1905231	1905389	-
1274933	1275199	-
1275574	1275750	-
99521143	99521766	-
80655341	80655530	-
44711425	44711782	-
180503084	180503615	-
180502031	180502811	-
33271002	33272137	-
33314543	33314840	-
33313938	33314148	-
28615914	28616284	-
28615589	28615906	-
27537072	27537273	-
55384628	55384851	-
27836892	27837303	-
8321681	8321945	-
92590053	92590292	-
11976490	11976683	-
62727209	62727424	-
50649480	50649633	-
28355872	28356544	-
28355233	28355578	-
3773026	3773217	-
33277122	33277585	-
33277598	33277775	-
33317740	33318212	-
10859003	10859323	-
33299177	33299987	-

130110371	130110485	-
33390871	33391440	-
136118759	136118894	-
136117836	136117973	-
66616519	66616932	-
36014504	36014727	-
36014114	36014317	-
51153159	51153673	-
876167	876309	-
975230	975544	-
200889464	200889820	-
50153922	50154493	-
50153309	50153701	-
66114726	66115000	-
66115341	66115524	-
101390754	101391177	-
30344743	30345124	-
30344181	30344700	-
99325690	99325894	-
99325459	99325623	-
33914656	33915309	-
44106971	44107159	-
30426301	30426518	-
19446268	19448639	-
35285014	35285220	-
1407122	1407438	-
111661190	111661456	-
4774926	4775174	-
140547248	140547766	-
18024500	18024955	-
18024016	18024214	-
10105782	10106310	-
135661266	135661830	-
135661842	135663169	-
66628502	66628666	-
50300087	50300469	-
50299154	50299326	-
43436232	43436803	-
54715416	54715918	-
34072941	34073292	-
51982811	51983192	-
51982483	51982756	51982628:51982639:+:CCTGCAGGGGGC
51984045	51984182	-
20661302	20661842	-
28357577	28357705	-
28357844	28358041	-
111631032	111631273	-
136791330	136791601	-
186109332	186109482	-
35664830	35665271	-
20438311	20439154	-
20437113	20437504	-
50115689	50116232	-
22589102	22589308	22589197:22589208:-:GCCCCCTGGTGT

66616519	66616932	-
160449877	160450342	-
160450441	160450833	-
160449305	160449606	-
166954975	166955959	-
33931599	33931728	-
58451349	58451711	-
271402	271610	-
45226649	45226893	-
46541820	46542100	-
31706528	31707261	-
46545423	46545614	-
10429860	10430637	10430368:10430379:+:CCGCCAGGGGGC
169479254	169480137	169479646:169479657:+:CCTGTAGAGGGC
90985677	90985880	-
90985914	90986142	-
90984603	90984821	90984732:90984743:-:GCCACCGGGTGG
106659817	106660242	-
36528904	36529252	-
10840037	10840337	-
144526589	144526728	-
111911814	111912142	-
71927903	71928342	-
57742428	57742716	-
62573970	62574285	-
62574445	62574765	-
66551295	66552295	-
45362662	45363067	45362965:45362976:+:CCTCTAGAGGGC
18193950	18194164	-
31546521	31546955	-
19192055	19192713	-
57424505	57424958	-
140400991	140401774	-
140402069	140402345	-
140402353	140402570	-
10115296	10115612	-
58579549	58580081	58579726:58579737:-:GCCCCCTGCCGG
31739188	31739832	-
31739839	31740550	-
58306153	58306375	58306159:58306170:+:ACACCAGAGGGC
112086676	112086907	-
112086325	112086647	-
48877701	48878512	-
62665357	62665929	-
62664678	62664878	-
57706354	57706644	-
114400253	114400560	-
41349850	41349982	-
225923404	225923666	-
63974611	63974797	-
63974886	63975045	-
133130542	133130898	-
119085620	119085942	-
67374273	67374732	67374313:67374324:-:GCCCCCTGCGGG

2508865	2509359	-
42931878	42932147	-
45998695	45998827	-
133130542	133130898	-
122266166	122266727	-
121888178	121889016	-
72053892	72054007	-
566848	567119	-
120446211	120446485	-
101390754	101391177	-
47273607	47273900	-
56316247	56316563	-
63657520	63657708	-
63658156	63658404	-
63658447	63658787	-
32154209	32154908	-
111879265	111880057	-
111878954	111879245	-
19516618	19516805	-
10840037	10840337	-
35122051	35122369	-
64506678	64507000	-
89921916	89922155	-
8064483	8064753	-
78080753	78080895	-
78079560	78079956	-
78079246	78079552	-
47254116	47254406	-
47254116	47254406	-
45586781	45586986	-
87316810	87317355	-
72375894	72376249	-
66517092	66517362	-
30559115	30559441	-
30558163	30558300	30558318:30558329:+:GCACTAGAGGGC
31968357	31968740	-
55407484	55407686	-
30534660	30534846	-
75241330	75241476	-
75241656	75241844	75241778:75241789:-:GCCCCCTGCTGG
55407484	55407686	-
67935123	67935710	-
49422543	49423116	-
4426583	4426799	-
4425320	4425906	-
46922788	46923157	-
206470395	206470612	206470500:206470511:+:CCACAAGGAGGC
145824253	145824630	-
32612281	32612829	-
32612967	32613146	-
49492382	49492534	-
120176176	120176801	-
150293483	150293880	-
150293968	150294256	-

42964350	42964598	-
2739308	2739540	-
42980418	42980579	-
42980957	42981060	-
35497705	35498125	-
35497026	35497671	-
76732961	76733507	-
5903610	5904279	-
5905027	5905229	-
4539719	4540322	-
10232074	10232208	-
5903610	5904279	-
4788771	4788901	-
35748294	35748713	-
8321681	8321945	-
57477565	57477784	-
57819572	57819798	-
16660135	16660311	-
16660521	16660828	-
17266916	17267243	-
154540936	154541099	-
154541362	154541732	-
154541784	154542008	-
57819572	57819798	-
12670025	12670364	-
12484962	12485196	-
40798345	40798726	-
40798103	40798308	40798241:40798252:-:GCCCTCAGGTGG
139340154	139342541	-
9324755	9324884	-
231528276	231528909	-
19596361	19597099	-
196942366	196942680	-
196943192	196943403	-
102853957	102854175	-
34426005	34426449	-
34425651	34425834	-
34425279	34425487	-
653595	654346	-
35031579	35031837	-
89632659	89632877	-
89633099	89633458	-
158168330	158168802	-
34392613	34393262	-
34391586	34391979	-
10248817	10249202	-
52686	53557	-
69339930	69340098	-
69339521	69339661	-
134226263	134226601	-
112860767	112861072	-
112861076	112861392	-
53347562	53347807	-
136791330	136791601	-

4604711	4604863	-
65013902	65014056	-
65013279	65013461	-
65014246	65014476	-
99154828	99155147	-
99155152	99155522	-
131796855	131797298	-
47176662	47177390	-
47176197	47176645	-
196337836	196338792	-
196337461	196337745	-
38825578	38825775	-
38825301	38825564	-
69369582	69369973	-
108963000	108963247	-
75073928	75074168	-
27893053	27893559	-
27146802	27147301	-
27147470	27147835	-
55618764	55619079	-
55618133	55618656	-
37609709	37609884	-
22109594	22110023	-
95165335	95165805	-
27139063	27139424	-
37406156	37406299	-
26188780	26189194	-
36535005	36535162	-
36535426	36535613	-
247078721	247078886	-
53052714	53052902	-
29663379	29663815	-
37406156	37406299	-
62665357	62665929	-
62664678	62664878	-
27893053	27893559	-
26027057	26027482	-
149718129	149718755	-
38728538	38728705	-
69902490	69902606	-
138773362	138773917	-
11284040	11284550	-
88686925	88687142	-
88686644	88686865	-
45499372	45500589	-
29815346	29815462	-
29815915	29816335	-
139273417	139273714	-
139273722	139274012	-
25160426	25160839	-
29817167	29817348	-
23565434	23565837	-
131994364	131994944	-
53378078	53378331	-

99734331	99734441	-
114400253	114400560	-
26169392	26169701	-
121281891	121282120	-
42767254	42767486	-

:oding genes from ChIP-seq data. Nucleic Acids Res. 2017 Jan 04;45(D1):D43-D50. Ep
om ChIP-Seq data. Nucleic Acids Res. 2013 Jan;41(Database issue):D177-87.

pub 2016 Oct 23
