# Supplementary material for: HDAC8 Promotes Liver Metastasis of Colorectal Cancer via Inhibition of IRF1 and Upregulation of SUCNR1
Source: Oxid Med Cell Longev. 2022 Aug 16;2022:2815187. doi: 10.1155/2022/2815187 (PMC9400431; doi:10.1155/2022/2815187)
Supplement: Supplementary 3 — Table S3: multiple binding sites between IRF1 and the promoter region of SUCNR1 gene. [file 2815187.f3.pdf]

Matrix II	Name	Score	Relative	Sequence	Start	End	Strand	Predicted
MA0050.1	IRF1	12.4031	0.891287	SUCNR1	1536	1547	-	aataacgaa
MA0050.1	IRF1	12.132	0.885245	SUCNR1	1833	1844	+	gaaaataaa
MA0050.2	IRF1	15.3143	0.858548	SUCNR1	1822	1842	-	ttttatttt
MA0050.1	IRF1	10.6525	0.852264	SUCNR1	1482	1493	-	taaagggaa
MA0050.1	IRF1	10.2148	0.842508	SUCNR1	1495	1506	-	aaaactgaa
MA0050.2	IRF1	13.7635	0.840798	SUCNR1	1828	1848	-	tgaggcttt
MA0050.1	IRF1	10.0175	0.83811	SUCNR1	36	47	-	caaaatgat
MA0050.2	IRF1	13.3312	0.835849	SUCNR1	1816	1836	-	tttcacatt
MA0050.1	IRF1	9.09761	0.817605	SUCNR1	1827	1838	+	gaatgtgaa
MA0050.1	IRF1	9.03716	0.816258	SUCNR1	1664	1675	+	taaaatgaa
MA0050.1	IRF1	8.73889	0.809609	SUCNR1	282	293	+	gaaagagga
MA0050.2	IRF1	10.9076	0.808106	SUCNR1	1413	1433	-	agttatttt
MA0050.2	IRF1	10.2678	0.800783	SUCNR1	1491	1511	+	ttaaaattt

sequence
acc
agc
cacattcacatt
acc
att
tattttcacatt
agc
cacatttatttt
aat
aat
agc
ccattttatttt
cagttttatagt
