# Supplementary figures and images for: HDAC8 Promotes Liver Metastasis of Colorectal Cancer via Inhibition of IRF1 and Upregulation of SUCNR1
Source: Oxid Med Cell Longev. 2022 Aug 16;2022:2815187. doi: 10.1155/2022/2815187 (PMC9400431; doi:10.1155/2022/2815187)

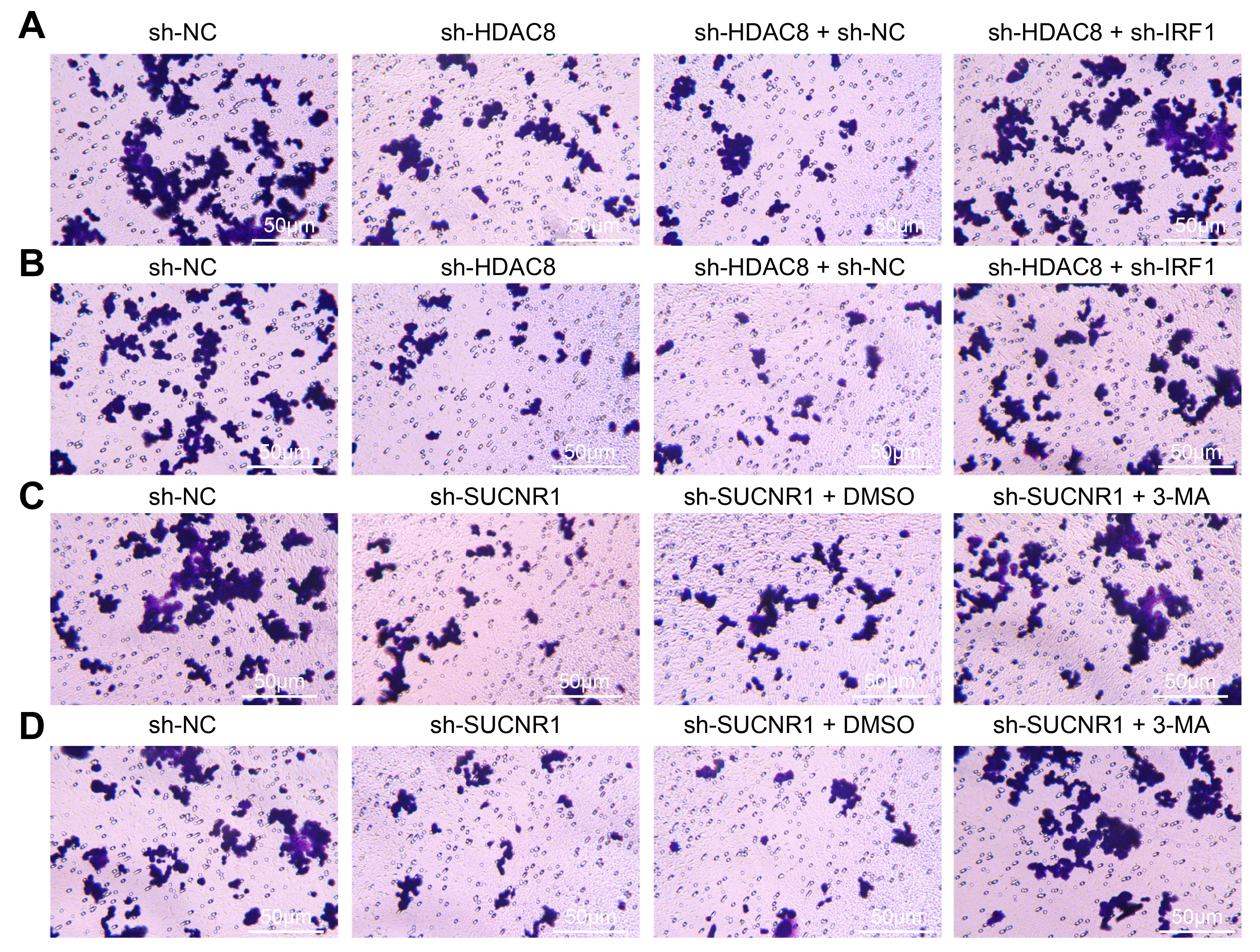

Supplement: Supplementary 4 — Figure S1: representative images of Transwell assay. [file 2815187.f4.jpg]
